# Investigating the OXA Variants of ESKAPE Pathogens

**DOI:** 10.3390/antibiotics10121539

**Published:** 2021-12-15

**Authors:** Deeksha Pandey, Neelja Singhal, Manish Kumar

**Affiliations:** Department of Biophysics, University of Delhi South Campus, New Delhi 110021, India; deeksha.pandey.biophysics@south.du.ac.in

**Keywords:** ESKAPE pathogens, OXA, β-lactamase, plasmid, chromosome, class D, carbapenemase, multidrug resistance, nosocomial infections, antimicrobial resistance

## Abstract

ESKAPE pathogens are the leading cause of nosocomial infections. The Global Priority List of WHO has categorized ESKAPE as priority 1 and 2 pathogens. Even though several mechanisms contribute to antimicrobial resistance, OXA β-lactamase has emerged as a new threat in combating nosocomial infections. In the present study we have investigated the presence of OXA and their variants, copy number, distribution on chromosomes/plasmids, subfamilies, phylogenetic relationships, amino acid identities and variabilities in ESKAPE pathogens. Our results revealed that a total of 929 OXA were present in 2258 completely assembled genomes, which could be further subdivided into 16 sub-families. Among all the ESKAPE pathogens, OXA were highly prevalent in *A. baumannii*, followed by *P. aeruginosa* and *K. pneumoniae* but completely absent in *E. faecium* and *S. aureus* while, only a few copies were found in *Enterobacter* spp. Most of the OXA variants belonged to the OXA-51-like subfamily (200 proteins), followed by OXA-50-like subfamily (189 proteins), OXA-23-like subfamily (156 proteins) and OXA-1-like subfamily (154 proteins). OXA-51-like, OXA-213-like, OXA-134-like, OXA-58-like, OXA-24-like and OXA-20-like subfamilies were present exclusively in *A. baumannii.* Phylogenetic tree of the subfamilies revealed that OXA-1-like and OXA-33-like, OXA-51-like and OXA-213-like and, OXA-5-like and OXA-10-like belonged to the same branches with amino acid identities as 100%, 97.10% and 80.90% respectively. This indicates that the members of these subfamily-pairs might have evolved from the same ancestor or have recently diverged. Thus, a judicious use of carbapenems is warranted to curtail the rise of new OXA enzymes and preserve them. This is the first detailed report about the OXA of ESKAPE pathogens.

## 1. Introduction

Indiscriminate use, misuse and overuse of antibiotics has escalated into antimicrobial resistance (AMR) and multidrug resistance (MDR) which are regarded as the greatest scourges of the 21st century [1,2]. AMR and MDR can evolve in bacteria by gene mutations and/or resistance determinants can be acquired through horizontal gene transfer from other bacteria [3,4]. ESKAPE pathogens (*Enterococcus faecium, Staphylococcus aureus, Klebsiella pneumoniae, Acinetobacter baumannii, Pseudomonas aeruginosa,* and *Enterobacter* species) are of global concern due to their involvement in several hospital-acquired infections (nosocomial infections). Hence, WHO has included them in the ‘Global Priority List of Antibiotic-resistant Bacteria to Guide Research, Discovery and Development of New Antibiotics’ [5]. Four members of this group viz. *K. pneumoniae*, *A. baumannii*, *P. aeruginosa* and *Enterobacter* spp. are included in priority 1 category (critical) while two members *E. faecium* and *S. aureus* come in priority 2 (high) category.

ESKAPE pathogens employ multiple approaches to neutralize the effect of antibiotics. These include (i) inactivation or alteration of antimicrobial molecules, (ii) bacterial target site modifications, (iii) reduced antibiotic penetration/accumulation and (iv) formation of bacterial biofilms [4,6,7]. Of these, the most common mechanism of AMR is production of enzymes that can hydrolyze or covalently modify the drugs which hinder their interaction with the drug-target site [8]. Bacterial β-lactamases and aminoglycoside-modifying enzymes are responsible for inactivating many β-lactam antibiotics and aminoglycosides respectively [9,10]. Bacterial β-lactamases hydrolyze the β-lactam ring of the β-lactam antibiotics and are a very diverse group of enzymes. As of now, nearly a thousand variants of β-lactamase have been reported and newer variants are consistently being reported [11,12]. On the basis of similarity in the primary amino acid sequence, β-lactamases are divided into four classes named as A, B, C and D. Enzymes of class A, C and D are active site serine β-lactamases, while Class B enzymes are metallo-β-lactamases [13,14]. Even though class A, C and D serine β-lactamases usually show a determined pattern of functions/activities, class B metallo-β-lactamases do not [15]. Even though class D β-lactamases are non-metallo enzymes, their activity might be influenced by addition of cofactors such as divalent metal ions which augments the enzyme stability [16]. Several variants of class A and D β-lactamases have emerged in recent years with enhanced ability to hydrolyze oxyimino-cephalosporins and carbapenems. This raises the alarm bell because oxyimino-cephalosporins and carbapenems are considered as last resorts in the drug arsenal against MDR infections [17,18,19,20].

Class D β-lactamases are known as oxacillinases or OXA. These were the earliest detected β-lactamases which were found to be widely distributed among Gram-negative bacteria [21]. Class D β-lactamases are intrinsic to many Gram-negative bacteria including *A. baumannii* and *P. aeruginosa* and play an important role in natural resistance phenotypes. However, during the past few years, the spectrum of class D OXA β-lactamases (*blaOXA*) has expanded greatly, with addition of several new variants [20,21]. The genes encoding *blaOXA* are found on both chromosomes and plasmids of diverse bacterial species [22,23,24] reflecting their potential for widespread dissemination among clinically relevant pathogens [25,26]. Several OXA variants were found to be responsible for pathogen-associated outbreaks in the non-endemic regions of the world due to their association with mobile genetic elements [27]. Moreover, detection of OXA variants such as OXA-48-like enzymes is challenging for clinical laboratories and can be confirmed only by gene sequencing. The diversity and the potential of these enzymes necessitate a detailed study of their characteristics so as to enable efficient strategies to combat OXA positive ESKAPE pathogens. Thus, in the present study we have investigated the OXA and their variants, copy number, distribution on chromosomes/plasmids, subfamilies, phylogenetic relationships, amino acid sequence identities/variabilities among OXA of ESKAPE pathogens. This is the first detailed report on the OXA of ESKAPE pathogens.

## 2. Results and Discussion

### 2.1. Data Statistics and Classification of ESKAPE Pathogens

Of the 2256, 12,397, 10,383, 5057, 5711 and 2680 entries enlisted in the NCBI genome database corresponding to *E. faecium*, *S. aureus*, *K. pneumoniae*, *A. baumannii*, *P. aeruginosa* and *Enterobacter* spp. only; 187, 590, 732, 233, 291 and 225 entries corresponded to the complete genomes of *E. faecium*, *S. aureus*, *K. pneumoniae*, *A. baumannii*, *P. aeruginosa* and *Enterobacter* spp., respectively (Table 1). Among these, 12 entries corresponded to only chromosomes and 175 corresponded to both chromosomes and plasmids of *E. faecium*. Furthermore, 285 entries corresponded to only chromosomes and 305 corresponded to both chromosomes and plasmids of *S. aureus*. In this case, 63 entries corresponded to chromosomes and 669 corresponded to both chromosomes and plasmids of *K. pneumoniae.* Furthermore, 50 entries corresponded to only chromosomes and 183 corresponded to both chromosomes and plasmids of *A. baumannii.* In *P. aeruginosa* 254 entries corresponded to only chromosomes and 37 corresponded to both chromosome and plasmids. Genomes of the majority of the ESKAPE pathogens showed the presence of plasmid(s). An interesting pattern was observed in the number of plasmids in the ESKAPE pathogens. The average number of plasmids per genome was approximately four in the case of *E. faecium, K. pneumoniae* and *Enterobacter* spp. In *A. baumannii*, the number of plasmids per genome was nearly 2. However, in the case of *S. aureus* and *P. aeruginosa*, the average number of plasmids per genome was 1.

### 2.2. Construction of Profile HMM of BlaOXA

A total of 363 protein sequences corresponding to the OXA family of class D β-lactamases were discerned using CBMAR, UniProtKB and NCBI NR databases. The MSA of these 363 protein sequences was manually analyzed and after removing the fragmented sequences, remaining 329 sequences were used to build a profile HMM (pHMM). Re-validation of the HMMSCAN outputs with the GenBank annotations of the corresponding genomes did not reveal any unannotated OXA or extra OXA. This indicates that the pHMM developed by us showed 100% specificity for the OXA family of class D β-lactamases.

### 2.3. Distribution and Localization of BlaOXA in ESKAPE Pathogens

A total of 929 OXA proteins were discerned in the 2258 completely assembled genomes of ESKAPE pathogens (Table 1). Interestingly, OXA were found to be completely absent in *E. faecium* and *S. aureus*. In *S. aureus*, during pHMM search, one hit corresponding to chromosomal and 174 hits corresponding to plasmidic OXA were discerned. However, during re-validation it was observed that these were regulatory proteins *BlaR1*; hence they were removed from further analyses. Several earlier studies have also reported that the sensor domains of *BlaR1* are similar in sequence and in three-dimensional structures with the class D OXA β-lactamases [28,29,30,31].

In *K. pneumoniae*, six OXA were discerned in 63 complete chromosomal genomes assemblies and in the 669 complete genomes assemblies representing both chromosomes and plasmids, 39 OXA were discerned on chromosomes and 218 OXA were discerned on 2362 plasmids. This indicated that OXA were more frequent on plasmids (9%) than on chromosomes (6%) in *K. pneumoniae*.

In *A. baumannii,* 82 OXA were discerned in the 50 complete chromosomal genome assemblies. In addition, 283 OXA were discerned on chromosomes and 43 OXA were discerned on 384 plasmids in the 183 complete genomes assemblies containing both chromosomes and plasmids. This suggests that a greater number of OXA were present on chromosomes (15%) than on plasmids (12%) and more than one copy of OXA might be present on chromosomes of *A. baumannii* strains.

In *P. aeruginosa*, 199 OXA were present on the 254 complete chromosomal genomes assemblies, while 37 OXA were present on chromosomes and 11 OXA were present on 50 plasmids in the 37 complete genomes assemblies representing both chromosomes and plasmids. This suggests that OXA were highly frequent on chromosomes (80%) than on plasmids (22%) in *P. aeruginosa*.

In *Enterobacter* spp., OXA were discerned in only a few genome assemblies. It was observed that OXA were not present in any of the 53 complete chromosomal genomes assemblies. However, four OXA were present on chromosomes and 19 OXA were present on 593 plasmids in the 172 complete genomes assemblies containing both chromosomes and plasmids. This suggests that *blaOXA* were less frequent on chromosomes (2%) and plasmids (3%) of *Enterobacter* spp.

Based on our findings, it can be inferred that the distribution and localization of OXA varied in the ESKAPE pathogens. OXA was found to be completely absent from the reported genomes of *E. faecium* and *S. aureus* while only a few copies could be discerned in the genomes of *Enterobacter* spp. Among all the ESKAPE pathogens, OXA was highly prevalent on the chromosomes of *A. baumannii* (~1.5 copies) per genome, followed by *P. aeruginosa* and *K. pneumoniae*. Our results exhibited complete concordance with a recent study, which also reported presence of a large number of *blaOXAs* in *A. baumannii, K. pneumoniae and P. aeruginosa* [32].

### 2.4. Classification of BlaOXA Variants in Subfamilies

The 929 OXA proteins included in this study formed 16 clusters hence, the OXA variants discerned in the ESKAPE pathogens were classified in 16 subfamilies using the information provided in the BLDB database developed by Naas et al. [33]. The detailed information about these proteins, OXA variants, subfamily classification and the associated ESKAPE pathogen is given in Supplementary Materials Table S1.

The maximum number of *blaOXA* belonged to the OXA-51-like subfamily (200 proteins), followed by OXA-50-like subfamily (189 proteins), OXA-23-like subfamily (156 proteins), OXA-1-like subfamily (154 proteins), OXA-48-like subfamily (86 proteins), OXA-10-like subfamily (42 proteins), OXA-9-like subfamily (32 proteins), OXA-134-like subfamily (24 proteins), OXA-58-like subfamily (14 proteins), OXA-2-like subfamily (12 proteins), OXA-24-like subfamily (11 proteins), OXA-5-like subfamily (4 proteins), OXA-33-like subfamily (2 proteins), OXA-198-like, OXA-213-like and OXA-20-like subfamilies (1 protein each). Interestingly, the bigger subfamilies, OXA-51-like and OXA-213-like were present exclusively in *A. baumannii* as were small subfamilies OXA-134-like, OXA-58-like, OXA-24-like and OXA-20-like (Table 2). In addition, enzymes of the subfamilies OXA-51-like and OXA-134-like were present on both plasmids and chromosomes while, enzymes of the subfamilies OXA-58-like and OXA-24-like were present on plasmids and enzymes of the subfamilies OXA-20-like and OXA-213-like were present on chromosomes of *A. baumannii* strains. Similar to the earlier reports, our results also revealed that OXA-20-like enzymes were present only on the chromosomes of *A. baumannii* [34]. An earlier study had reported that OXA-51-like enzymes were present exclusively in *A. baumannii* [34] while, OXA-24-like enzymes were exclusive to *Acinetobacter* spp. Similar to an earlier report, in this study too, OXA-58-like enzymes were found to be specifically present on the plasmids of *A. baumannii* [34].

The OXA-50-like (189 proteins), OXA-33-like (2 proteins) and OXA-198-like (1 proteins) subfamilies were present exclusively in *P. aeruginosa*. An earlier study also reported that OXA-198-like enzymes were present only in *P. aeruginosa* [33]. The OXA-48-like subfamily (86 proteins) was found exclusively on the chromosomes and plasmids of *K. pneumoniae*. The OXA-48 subfamily has been reportedly present only in the *Enterobacteriaceae* family [35]. Other subfamilies, OXA-1-like (154 proteins), OXA-10-like (42 proteins), OXA-9-like (32 proteins) and OXA-2-like (12 proteins) were found to be present in *K. pneumoniae, P. aeruginosa* and *Enterobacter* spp. Earlier studies also reported that enzymes of the OXA-2-like subfamily were predominantly present in *P. aeruginosa* [34] but can also be found in some members of *K. pneumoniae* [36] and *Acinetobacter* spp. [37]. The OXA-23-like subfamily (156 proteins) was exclusively present in *A. baumannii* and *P. aeruginosa* while, OXA-5-like subfamily (4 proteins) was exclusive to *P. aeruginosa* and *Enterobacter* spp.

### 2.5. Identification of Relatedness among BlaOXA

**Figure 1:**
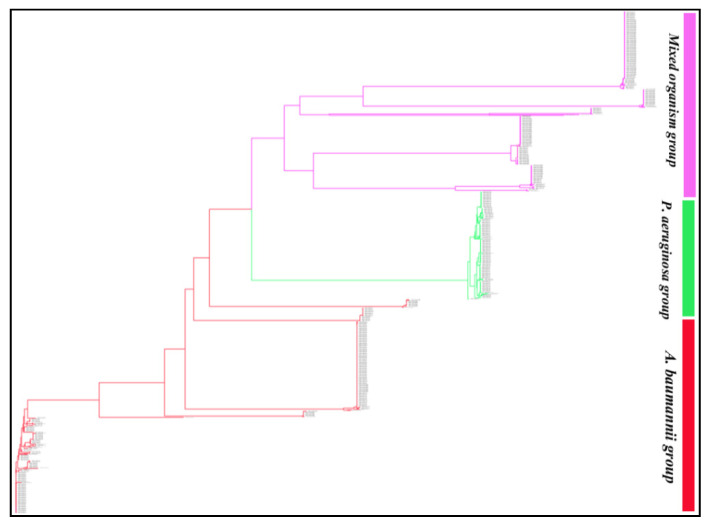
Phylogram of the 929 *blaOXA* sequences of *Klebsiella pneumoniae*, *Acinetobacter baumannii*, *Pseudomonas aeruginosa*, and *Enterobacter* spp. The 929 sequences formed three main clusters with 927 leaves and 1856 nodes. The tree session is also shared for a broader view (https://phylogeny.io/share/08bcde3be7fa2fb9c87e77fff2968967b5fa0f3d, accessed on 15 September 2021). In the shared tree session, the zoom in and out, scrolling (up, down, left and right) option is available which can be used to discern the interest of branches and clades of each enzyme group and their sequences.

Phylogenetic analysis of the 929 *blaOXA* sequences revealed that OXA variants of the ESKAPE pathogens formed three distinct groups (Figure 1). *BlaOXA* of *A. baumannii* and *P. aeruginosa* formed one separate branch each, while the third branch consisted of OXA of *K. pneumoniae*, *P. aeruginosa*, *A. baumannii* and *Enterobacter* spp. This group mostly included the sequences of *K. pneumoniae* and a few sequences of *P. aeruginosa*, *A. baumannii* and *Enterobacter* spp. A detailed analysis of the phylogenetic tree revealed that similar OXA variants clustered together within their subfamily, irrespective of their location (chromosomal/plasmidic) or the pathogen (Figure S1). OXA subfamilies such as, OXA-48-like, OXA-10-like, OXA-9-like, OXA-2-like, OXA-23-like, OXA-1-like and OXA-5-like which were present in all the ESKAPE pathogens formed separate clusters but, these clusters could be sub-grouped on the basis of the pathogen, also. However, OXA variants of the subfamilies OXA-50-like, OXA-33-like and OXA-198-like present only in *P. aeruginosa* formed three independent clusters.

### 2.6. Analysis of Amino Acid Variations in OXA Subfamilies

Analysis of the amino acid variations within the 16 OXA subfamilies revealed that amino acid sequences of OXA variants of subfamilies such as, OXA-9-like, OXA-23-like and OXA-33-like were fully conserved. The maximum variations were observed in members of the OXA-51-like subfamily (largest subfamily), followed by OXA-50-like, OXA-1-like, OXA-48-like and OXA-10-like subfamilies. Since, OXA-51-like subfamily consists of the maximum number of *blaOXA* proteins reported till date, it is natural that the maximum number of variations was observed in this subfamily, followed by other subfamilies (Table 2 and Figure S2).

### 2.7. Pairwise Identity between the Subfamilies

Since, OXA is a highly diverse group of β-lactamases; hence we analyzed the relatedness among different *blaOXA* subfamilies by carrying out pairwise sequence alignment among the representative sequences of the 16 subfamilies. It was observed that the pairwise identity between different subfamilies of OXA ranged from 19.90–100% (Table 3). For example, OXA-23-like and OXA-9-like subfamilies showed only 19.90% amino acid identity, whereas OXA-10-like and OXA-5-like subfamilies showed 80.90% identity. The OXA subfamily pair OXA-33-like and OXA-1-like showed 100% similarity, OXA-213-like and OXA-51-like 97.10% similarity, OXA-10-like and OXA-5-like 80.90% similarity and OXA-2-like and OXA-20-like subfamily showed 72.27% similarity. This suggests that these subfamily pairs either belonged to the same parent family or might have diverged recently.

### 2.8. Amino Acid Analysis of Representatives of OXA Subfamilies and Their Phylogenetic Study

**Figure 2:**
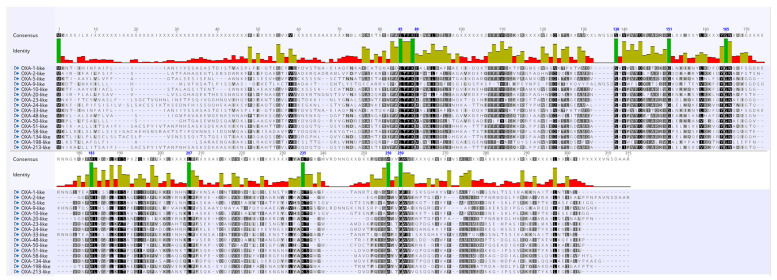
Multiple sequence alignment of a representative sequence from sequences of the 16 OXA subfamilies. In the conservation plot, identical amino acid residues are highlighted in dark green color.

Despite the fact that OXA is a very heterogeneous group of β-lactamases and different subfamilies share a low sequence identity, they all belong to Ambler class D. Hence, to discern a few characteristics that might be common to all the subfamilies, amino acid sequences of representative sequences of the 16 *blaOXA* subfamilies were analyzed. The STFK motif characteristic of serine class β-lactamases was found to be conserved in each representative of the 16 OXA subfamilies. Amino acid sequences at position 85–89, S at position 138, G at position 151, 165 and 259, W at position 183, L at position 207, K at position 235, W at position 256 and G at position 259 showed 100% conservation (shown in dark green color in Figure 2).

**Figure 3:**
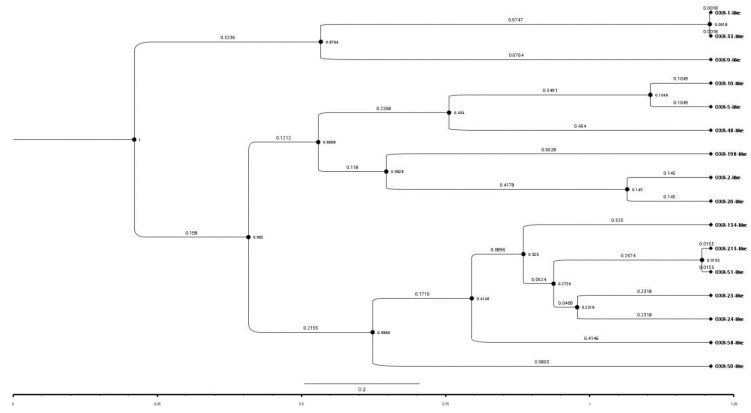
A time-stamped phylogenetic tree of a representative sequence from sequences of the 16 OXA subfamilies. The tree session is also shared for a broader view (https://raw.githubusercontent.com/University-of-Delhi-south-campus/OXA-beta-lactamase/main/Tree%20session.svg, accessed on 15 September 2021).

The time-stamped phylogenetic tree of representatives of the OXA subfamilies revealed that initially two main branches had evolved from a common ancestor. The first branch further split into two subbranches representing OXA-1-like/OXA-33-like and OXA-9-like subfamilies. However, the second main branch further split into two subbranches which during the course of time further split into 13 subbranches representing the 13 different subfamilies of OXA (Figure 3). Further, OXA-1-like and OXA-33-like, OXA-51-like and OXA-213-like and OXA-5-like and OXA-10-like subfamilies belonged to the same branches; hence, the members of these subfamily pairs might have evolved from the same ancestors. This is also supported by the pairwise similarity between OXA-1-like and OXA-33-like, OXA-51-like and OXA-213-like and, OXA-5-like and OXA-10-like subfamilies which were 100%, 97.1%, and 90.9%, respectively. The similarities between these subfamilies were also obvious by their branch-lengths and the time that had elapsed since their divergence from a common ancestor. This indicated a close relationship between them or a recent divergence, as also evident from their sequence identities. Overall, pairwise sequence identity and phylogenetic analysis showed a complete agreement with each other. This implied that more was the pairwise sequence identity between two sequences, greater was the divergence between them and, more was the time that had elapsed since they diverged from each other.

### 2.9. Structural Variations in the 3D Protein Models of the OXA Subfamilies

**Figure 4:**
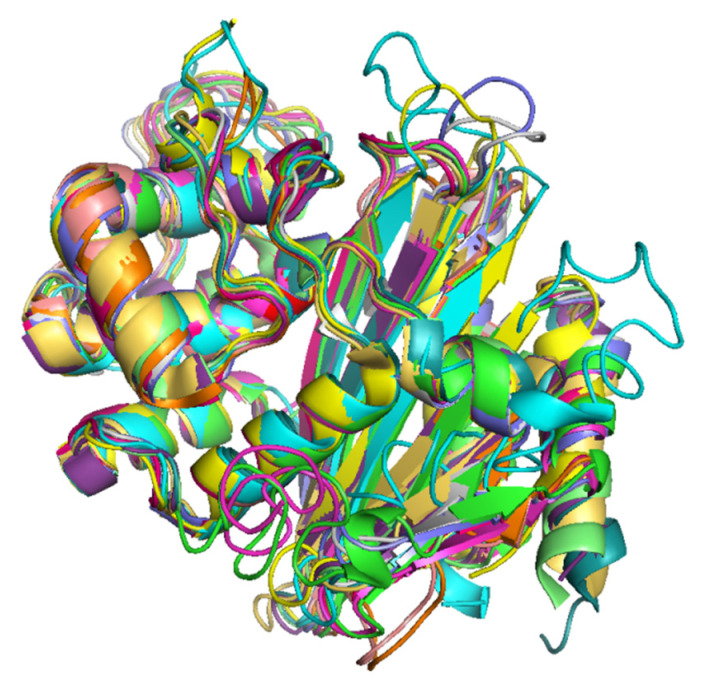
Superimposed 3D protein models of representatives of the 16 OXA subfamilies. (Color Key, Green: OXA-50-like, Cyan: OXA-9-like, Magenta: OXA-198-like, Yellow: OXA-1-like and OXA-33-like, Wheat: OXA-2-like, Grey: OXA-5-like, Blue: OXA-10-like, Orange: OXA-20-like, Light Green: OXA-23-like and OXA-134-like, Dark Green: OXA-24-like, Pink: OXA-48-like, Mustard: OXA-58-like, Purple: OXA-213-like and OXA-51-like.

The superimposition of 3D protein models of the representatives of each OXA-subfamily revealed that the protein models were highly superimposable (Figure 4). The details of RMSD values of pairwise structure alignments of the subfamilies are shown in Table 4. The maximum RMSD value was observed in OXA-9-like and OXA-198-like subfamilies, which showed a pairwise sequence identity of 22.50%. In the phylogenetic tree also, they represented two separate branches. Interestingly, the RMSD values of pairwise structural alignments of two OXA subfamily pairs namely, OXA-1-like and OXA-33-like and, OXA-51-like and OXA-213-like showed RMSD value as 0. The former protein pair showed 100% while the latter showed 97.10% pairwise sequence identity. In the phylogenetic tree OXA-1-like and OXA-33-like and, OXA-51-like and OXA-213-like were present on the same branches without any divergence. Thus, it could be inferred that when the RMSD value of pairwise alignment was low, the pairwise sequence identity was high and the degree of divergence was low. The multiple sequence alignment of the OXA sequences revealed that the primary and secondary amino acid sequences were highly conserved within a subfamily (Figure S2).

## 3. Materials and Methods

### 3.1. Data Collection

Complete genomes of *E. faecium, S. aureus, K. pneumoniae, A. baumannii, P. aeruginosa* and *Enterobacter* spp. (ESKAPE pathogens) were retrieved from the NCBI genome database (14 February 2021). The database showed 2256, 12,397, 10,383, 5057, 5711 and 2680 entries corresponding to the genomes of *E. faecium, S. aureus, K. pneumoniae, A. baumannii, P. aeruginosa,* and *Enterobacter* spp. respectively. To minimize exclusion of any OXA variant from our study, incomplete genome assemblies were strictly not included in analysis. The statistics at different stages of data collection is summarized in Table 1. On the basis of the types of replicons present in the genome, each genome was divided into two categories: (a) those that contained only chromosomes and (b) those that contained both chromosome and plasmid(s). All the replicons were subsequently translated as proteins in the six reading frames.

### 3.2. Building Profile HMM and OXA Search in the Genomes of ESKAPE Pathogens

The OXA sequences of class D β-lactamases were retrieved from UniProtKB, NCBI NR and Comprehensive Beta-lactamase Molecular Annotation Resource (CBMAR), a database of β-lactamases earlier developed in our laboratory [38]. The OXA sequences were aligned using Muscle 3.8 [39] at default parameters. Alignment was manually examined and fragmented sequences were removed. From the multiple sequence alignment (MSA), a profile HMM (pHMM) was built using the hmmbuild program of HMMER package (version 3.1) [40]. The proteomes of all the ESKAPE pathogens obtained using the six-frame translation of each replicon was searched for the pHMM of OXA using the HMMSCAN program of HMMER package (version 3.1) with E-value threshold 1e-06. The HMMSCAN search results were cross-checked using the GenBank annotations of the corresponding genomes to remove non-OXA sequences.

### 3.3. Multiple Sequence Alignment and Phylogenetic Tree

The conservation pattern and evolutionary pattern of OXA in each ESKAPE pathogen was analyzed by MSA and a phylogenetic tree was built using the clustalw 2.1 program [41]. For phylogenetic analysis, the neighbor joining (NJ) method was used with 1000 bootstrap values. The MSA and tree were visualized together using jalview [42] and Phylogeny IO [43], respectively.

### 3.4. Determination of Pairwise Sequence Identity in Representatives of OXA Subfamilies and Phylogenetic Analysis

The first/topmost sequence of the MSA was considered as the representative of that subfamily. The pairwise identity in the 16 OXA subfamilies was calculated using the representative sequence of each subfamily. A representative protein sequence from each subfamily was used for comparison of amino acid sequences and construction of the phylogenetic tree. Bayesian evolutionary analysis and time stamped phylogeny was created using BEAST [44]. Prior to using BEAST, the tree file in nexus format of OXA subfamilies and representatives’ sequences were created using the ClustalW program. The nexus format tree file was used to load the parameters, which were further used for BEAST analysis. This was carried out using BEAUti program with substitution models and clock models by default priors. The MCMC was run for 10,000 iterations with a default burn-in period and samples saved every 10,000 iterations. The loaded parameters file obtained from BEAUti was saved as an .xml extension. The .xml file was used to run BEAST at default settings. The BEAST output was analyzed using tracer programs. The posterior sample of phylogenetic time-tree along with its parameter estimates was summarized using the program TreeAnnotator. The burnin and posterior probability limit was set to 50 and 0.0, respectively, maximum clade credibility tree with default options and mean heights for node heights was chosen which sets the heights (ages) of each node in the tree to the mean height across the entire sample of trees for that clade. The tree was visualized using the FigTree program which displayed node Bars, node age error bars, branch labels and posterior probability for each node.

### 3.5. 3D Modeling and Assessment of Structural Variations in the OXA Subfamilies

The structural variations among the 3D protein models of representative sequences of each OXA-subfamily were performed using BLAST search against the PDB. The OXA protein structures obtained using BLAST search were selected on the basis of % alignment identity and % query coverage. For 13 subfamilies namely, OXA-10-like, OXA-2-like, OXA-48-like, OXA-24-like, OXA-58-like, OXA-1-like, OXA-213-like, OXA-33-like, OXA-51-like, OXA-23-like, OXA-5-like, OXA-20-like and OXA-134-like protein structures were found in the PDB database which showed more than 60% identity and 85% query coverage. Hence, these structures were used as representatives of their OXA subfamily. The PDB id, query coverage and identity of the subfamily members and best hits/templates are shown in Table 5. For the remaining three subfamilies, OXA-9-like, OXA-198-like and OXA-50-like, the protein models were created using the I-TASSER server [45]. To model a representative 3D structure of OXA-9-like, OXA-198-like and OXA-50-like subfamilies we used the top five structures as template (Table S2a). The best 3D model was selected on the basis of confidence score (C-score). Presence of 97%, 99%, 100% phi/psi angles of modeled structure of OXA-9-like, OXA-198-like and OXA-50-like subfamilies in the allowed regions of Ramachandran map (Table S2b) indicated absence of spatial constrains in the models. The nonbonded atomic interactions in the modeled protein structures were calculated using the ERRAT program. An average ERRAT score of 79.33, 86.50 and 95.27 for representative structures of modeled OXA-9-like, OXA-198-like and OXA-50-like proteins, respectively indicated that the models were highly accurate. Assessment of accuracy of the modeled structures of OXA-9-like, OXA-198-like and OXA-50–like subfamilies using Verify-3D program gave scores of 84.59%, 91.54% and 91.60%, respectively. Overall, all three structure model assessment tools categorized them as accurate models.

The structural alignment of the 3D models of the 16 OXA subfamilies was assessed by superimposing the models using PyMOL. The pairwise structural comparison of the 3D models was performed by calculating their RMSD values.

**Figure 5:**
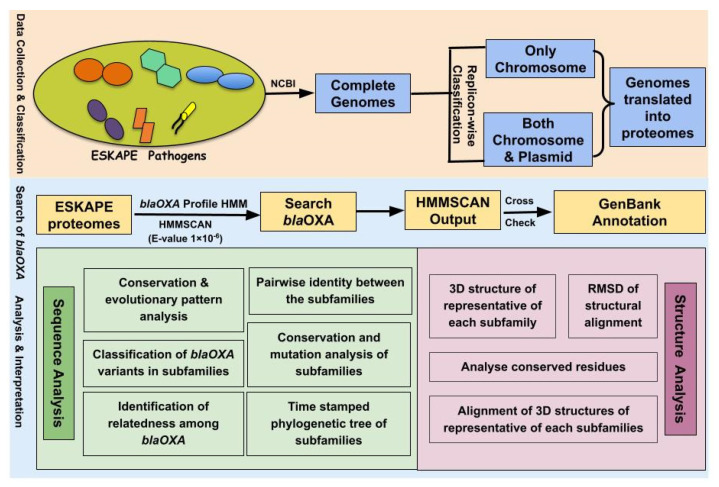
The overall workflow depicting the methodology used for investigating the OXA variants of ESKAPE pathogens.

The overall workflow depicting the methodology adopted in this study has been illustrated in Figure 5.

## 4. Conclusions

To summarize, a total of 929 OXA were discerned in 2258 completely assembled genomes of the ESKAPE pathogens, which could be further subdivided into 16 subfamilies. The distribution and localization of OXA varied in the ESKAPE pathogens. OXA were found to be completely absent in *E. faecium* and *S. aureus* while only a few copies were found in *Enterobacter* spp. Among all the ESKAPE pathogens, OXA were highly prevalent in *A. baumannii*, followed by *P. aeruginosa* and *K. pneumoniae.* Most of the OXA variants belonged to the OXA-51-like subfamily (200 proteins), followed by OXA-50-like subfamily (189 proteins), OXA-23-like subfamily (156 proteins) and OXA-1-like subfamily (154 proteins). OXA-51-like, OXA-23-like, OXA-213-like, OXA-134-like, OXA-58-like, OXA-24-like and OXA-20-like subfamilies were present exclusively in *A. baumannii.* Phylogenetic tree of the subfamilies revealed that OXA-1-like and OXA-33-like, OXA-51-like and OXA-213-like and OXA-5-like and OXA-10-like belonged to the same branches with amino acid identities as 100%, 97.10% and 80.90%, respectively. This indicates that the members of these subfamily-pairs might have evolved from the same ancestor or diverged recently. Thus, our results suggest a very judicious use of carbapenems to curtail the rise of new OXA enzymes and fence the diversity of current carbapenemases.

## Figures and Tables

**Table 1:** Number of genomes of ESKAPE pathogens found in NCBI Genome Database and statistics of *blaOXA* found therein.

Organism	Total No. of Genomes (NCBI)	No. of Complete Genomes/No. of *blaOXA*	Replicons			
			No. of Chromosomal Genomes/No. of *blaOXA*	Both Chromosome and Plasmid		
				No. of Genomes Containing Both Chromosomes and Plasmids/No. of *blaOXA* (Total)	No. of Chromosomes/No. of *blaOXA*	No. of Plasmids/No. of *blaOXA*
*E. faecium*	2256	187/0	12/0	175/0	175/0	851/0
*S. aureus*	12,397	590/0	285/0	305/0	305/0	467/0
*K. pneumoniae*	10,383	732/263	63/6	669/257	669/39	2362/218
*A. baumannii*	5057	233/408	50/82	183/326	183/283	384/43
*P. aeruginosa*	5711	291/235	254/199	37/36	37/25	50/11
*Enterobacter* spp.	2680	225/23	53/0	172/23	172/4	593/19
**Total**		**2258/929**	**717/287**	**1541/642**	**1541/351**	**4707/291**

Organism and replicon-wise statistics of data downloaded from NCBI and found number of hits of *blaOXA* with regards to ESKAPE pathogens.

**Table 2:** List of variations identified each of 16 OXA subfamilies.

S. No.	Enzyme Group	Organism and Gene Location(s)	Number of Sequences	Variations
1	OXA-48-like	*K. pneumoniae* (B: chromosome, B: plasmid, O: chromosome) *Enterobacter* spp. (B: plasmid)	86	T104A, N110D, E168Q, S171A, R214S
2	OXA-10-like	*K. pneumoniae* (B: plasmid) *P. aeruginosa* (B: chromosome, B: plasmid, O: chromosome) *Enterobacter* spp. (B: plasmid) *A. baumannii* (O: chromosome)	42	I10T, G20S, S27F, D55N, T107S, Y174F, E229G, S245N, E259A
3	OXA-9-like	*K. pneumoniae* (B: plasmid) *P. aeruginosa* (O: chromosome) *Enterobacter* spp. (B: plasmid)	32	Not found
4	OXA-2-like	*Enterobacter* spp. (B: chromosome) *K. pneumoniae* (B: plasmid) *P. aeruginosa* (B: chromosome, O: chromosome)	12	D321V
5	OXA-51-like	*A. baumannii* (B: chromosome, B: plasmid, O: chromosome)	200	A5T, T24S, E36V, E36D, E36K, A38G, A48V, Q57H, A96T, D105N, K107Q, K107E, D117, V129I, P130Q, K146N, L167V, K177Q, Q194P, K195E, D198H, L222W, D225N
6	OXA-20-like	*A. baumannii* (B: chromosome)	1	NA
7	OXA-23-like	*A. baumannii* (B: chromosome, B: plasmid, O: chromosome) *P. aeruginosa* (B: chromosome)	156	Not found
8	OXA-213-like	*A. baumannii* (B: chromosome)	1	NA
9	OXA-134-like	*A. baumannii* (B: chromosome, B: plasmid, O: chromosome)	24	D208G
10	OXA-58-like	*A. baumannii* (B: plasmid)	14	F114L, I161M, A256D
11	OXA-24-like		11	D224G
12	OXA-1-like	*K. pneumoniae* (B: chromosome, B: plasmid, O: chromosome) *P. aeruginosa* (B: chromosome, B: plasmid, O: chromosome) *Enterobacter* spp. (B: chromosome, B: plasmid)	154	L28F, V64A, I102M, D103N, N2111E E223D, N268S
13	OXA-50-like	*P. aeruginosa* (B: chromosome, O: chromosome)	189	F6L, A8T, T17A, Q26R, R50C, R84K, D110E, R168H, K113E
14	OXA-33-like	*P. aeruginosa* (B: chromosome)	2	Not found
15	OXA-198-like	*P. aeruginosa* (O: chromosome)	1	NA
16	OXA-5-like	*Enterobacter* spp. (B: plasmid) *P. aeruginosa* (O: chromosome)	4	V117L

NA: No variation found due to presence of one sequence in the subfamily.

**Table 3:** Pair-wise amino acid identities (%) between OXA subfamilies.

	OXA- 1-like	OXA- 2-like	OXA- 5-like	OXA- 9-like	OXA- 10-like	OXA- 20-like	OXA- 23-like	OXA- 24-like	OXA- 33-like	OXA- 48-like	OXA- 50-like	OXA- 51-like	OXA- 58-like	OXA- 134-like	OXA- 198-like	OXA- 213-like
OXA- 1-like	100.00															
OXA- 2-like	26.00	100.00														
OXA- 5-like	27.50	32.30	100.00													
OXA- 9-like	31.00	21.20	26.40	100.00												
OXA- 10-like	27.10	34.00	80.90	26.30	100.00											
OXA- 20-like	26.90	72.70	35.60	21.70	33.90	100.00										
OXA- 23-like	24.10	27.00	33.90	19.90	33.20	28.80	100.00									
OXA- 24-like	26.90	25.20	33.10	22.10	36.30	28.90	59.90	100.00								
OXA- 33-like	100.00	26.00	27.60	31.00	27.10	26.90	24.10	26.90	100.00							
OXA- 48-like	27.20	39.20	44.10	22.50	45.40	40.90	35.60	31.80	27.20	100.00						
OXA- 50-like	25.00	32.90	35.80	25.60	33.30	35.80	40.90	36.20	25.00	36.10	100.00					
OXA- 51-like	26.40	26.40	34.50	25.60	32.60	31.00	56.90	63.40	26.40	36.30	40.40	100.00				
OXA- 58-like	21.80	28.40	35.40	25.30	36.00	29.60	47.70	48.60	21.80	34.00	39.40	48.30	100.00			
OXA- 134-like	25.00	28.70	34.50	23.70	35.20	28.50	55.90	55.90	25.00	32.10	38.10	54.50	52.70	100.00		
OXA- 198-like	26.40	34.80	30.70	22.50	31.30	36.30	27.20	31.00	26.40	35.30	33.00	28.40	32.50	32.60	100.00	
OXA- 213-like	24.70	26.70	33.80	24.70	33.00	30.00	56.50	62.00	24.70	36.60	39.60	97.10	47.20	53.00	28.10	100.00

The color scale is from highest (green) to lowest (red).

**Table 4:** Root Mean Square Deviation values of pairwise structure alignments of 3D protein models of the 16 OXA subfamilies.

Super-Imposition	OXA-1-like	OXA-2-like	OXA-5-like	OXA-9-like	OXA-10-like	OXA-20-like	OXA-23-like	OXA-24-like	OXA-33-like	OXA-48-like	OXA-50-like	OXA-51-like	OXA-58-like	OXA-134-like	OXA-198-like	OXA-213-like
OXA-1-like	0															
OXA-2-like	0.972	0														
OXA-5-like	1.112	0.704	0													
OXA-9-like	0.784	1.099	1.756	0												
OXA-10-like	1.058	0.762	0.268	1.738	0											
OXA-20-like	0.96	0.226	0.715	1.174	0.759	0										
OXA-23-like	1.143	0.902	1.108	1.768	1.118	0.898	0									
OXA-24-like	1.023	1.007	1.027	1.052	1.07	0.946	0.426	0								
OXA-33-like	0	0.972	1.112	0.784	1.058	0.96	1.143	1.023	0							
OXA-48-like	0.817	0.613	0.659	0.946	0.805	0.645	0.823	0.848	0.817	0						
OXA-50-like	0.811	0.623	0.51	0.699	0.637	0.636	0.798	0.798	0.811	0.375	0					
OXA-51-like	0.963	0.893	1.081	1.364	1.131	0.912	0.41	0.478	0.963	0.817	0.759	0				
OXA-58-like	0.987	1.007	0.848	2.324	0.881	1.054	0.623	0.639	0.987	0.926	0.882	0.616	0			
OXA-134-like	1.143	0.902	1.108	1.768	1.118	0.898	0	0.426	1.143	0.823	0.798	0.41	0.623	0		
OXA-198-like	0.78	0.581	0.548	2.466	0.705	0.556	0.727	0.784	0.78	0.381	0.277	0.701	0.915	0.727	0	
OXA-213-like	0.963	0.893	1.081	1.364	1.131	0.912	0.41	0.478	0.963	0.817	0.759	0	0.616	0.41	0.701	0

**Table 5:** PDB IDs of the structural representatives of the 16 OXA subfamilies with query coverage and identity percentage.

S. No.	Protein Name	PDB ID	Query Coverage (%)	Identity (%)
1	OXA-10-like	1K6R	92	100
2	OXA-2-like	1K38	92	99.60
3	OXA-48-like	5OE0	100	99.60
4	OXA-24-like	4WM9	89	99.50
5	OXA-58-like	4OH0	100	99.29
6	OXA-1-like	1M6K	90	98.8
7	OXA-213-like	4ZDX	100	97
8	OXA-33-like	1M6K	90	98.80
9	OXA-51-like	4ZDX	100	97
10	OXA-23-like	4JF4	89	100
11	OXA-5-like	1FOF	91	84.01
12	OXA-20-like	6XJ3	92	77.30
13	OXA-134-like	4JF4	85	63.90
14	OXA-9-like	6NHU	89	46.06
15	OXA-198-like	6NLW	88	40.60
16	OXA-50-like	4JF4	90	44.30

## Data Availability

Data is contained within the article or supplementary material.

## Supplementary Materials

The following are available online at https://www.mdpi.com/article/10.3390/antibiotics10121539/s1, Figure S1: The phylogram of 929 OXA sequences of *Klebsiella pneumoniae, Acinetobacter baumannii, Pseudomonas aeruginosa, and Enterobacter* species, Figure S2: Subfamily wise structure based multiple sequence alignment of the OXA proteins present in OXA discerned in ESKAPE pathogens, Table S1: Distribution of OXA variants in ESKAPE pathogens, Table S2: (a) Target-Template details of selected modeled structures, (b) Model validation.

## Abbreviations

OXA	Oxacillinases
ESKAPE	*Enterococcus faecium, Staphylococcus aureus, Klebsiella pneumoniae, Acinetobacter baumannii, Pseudomonas aeruginosa,* and *Enterobacter* species
WHO	World Health Organization
AMR	Antimicrobial Resistance
MDR	Multidrug Resistance
*blaOXA*	OXA β-lactamase
ARG	Antibiotic Resistance Gene
MSA	Multiple Sequence Alignment
HMM	Hidden Markov Model
RMSD	Root Mean Square Deviation
CBMAR	Comprehensive Beta-lactamase Molecular Annotation Resource
NCBI	National Center for Biotechnology Information
NR	Non Redundant
BLDB	Beta-Lactamase Database
NJ	Neighbor joining
BLAST	Basic Local Alignment Search Tool
BEAST	Bayesian evolutionary analysis through a simple tutorial
BEAUti	Bayesian Evolutionary Analysis Utility
MCMC	Markov chain Monte Carlo
