# Supplementary material for: Investigating the OXA Variants of ESKAPE Pathogens
Source: Antibiotics (Basel). 2021 Dec 15;10(12):1539. doi: 10.3390/antibiotics10121539 (PMC8699015; doi:10.3390/antibiotics10121539)
Supplement: Supplementary file 1 [file antibiotics-10-01539-s001.zip › antibiotics-1401819-supplementary.pdf]

antibiotics

MDPI

Article

Investigating the OXA variants of ESKAPE pathogens

Deeksha Pandey [1], Neelja Singhal [1,*] and Manish Kumar[1,*]

Department of Biophysics, University of Delhi South Campus, New Delhi, 110021, India ; deeksha.pandey.bi physics@south.du.ac.in(D.P.);
* Correspondence: neelja@south.du.ac.in (N.S.); manish@south.du.ac.in (M.K.)

Supplementary Materials

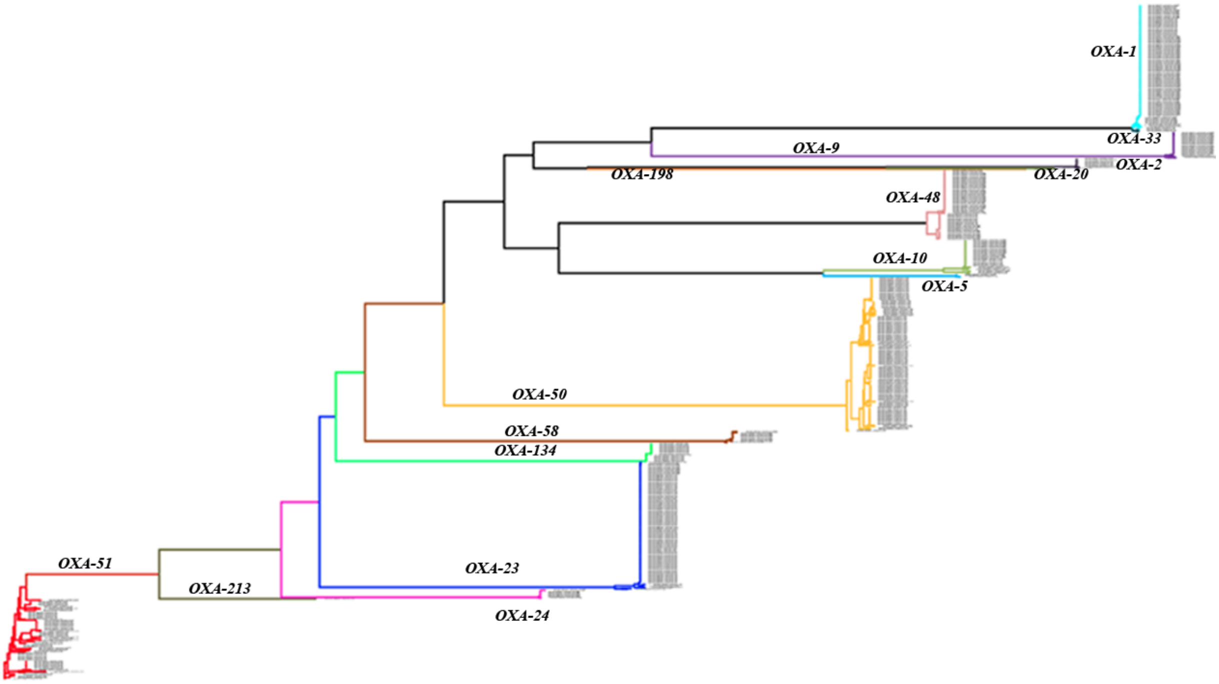

Figure S1. The phylogram of 929 OXA sequences of *Klebsiella pneumoniae, Acinetobacter baumannii, Pseudomonas aeruginosa, and Enterobacter* species. Each enzyme group was colored with a different shade. A total of 929 sequences formed 927 leaves and 1856 nodes. The tree session is also shared for a broader view (https://phylogeny.io/share/97725f9b8259229493b0147bee59aedde7277a6f).). In the shared tree session, the zoom in and out, scrolling (up, down, left & right) option is available which can be used to discern the branches and clades of each enzyme group and their sequences.

Figure S2 Please see the PDF: Figure S2.

Table S1. Distribution of OXA variants in ESKAPE pathogens.

Organism	Variants/subfamilies of OXA found in chromosome where both chromosome and plasmid are genetic material	Variants/subfamilies of OXA found in plasmid where both chromosome and plasmid are genetic material	Variants/subfamilies of OXA found in chromosome where only chromosome is genetic material
E. faecium	0	0	0
S. aureus	BlaR1	BlaR1	0
K. pneumoniae	OXA-1/OXA-1-like<br>OXA-48/OXA-48-like<br>OXA-181/OXA-48-like	OXA-1/ OXA-1-like<br>OXA-2/OXA-2-like<br>OXA-204/OXA-48-like<br>OXA-244/OXA-48-like	OXA-181/ OXA-48-like<br>OXA-1/OXA-1-like<br>OXA-48/ OXA-48-like

		OXA-181/OXA-48-like<br>OXA-232/OXA-48-like<br>OXA-10/OXA-10-like<br>OXA-9/OXA-9-like<br>OXA-48/OXA-48-like	
A. baumannii	OXA-106/ OXA-51-like<br>OXA-109/ OXA-51-like<br>OXA-113/ OXA-51-like<br>OXA-126/ OXA-51-like<br>OXA-132/ OXA-51-like<br>OXA-144/ OXA-51-like<br>OXA-173/ OXA-51-like<br>OXA-180/ OXA-51-like<br>OXA-2/ OXA-2-like<br>OXA-20/ OXA-20-like<br>OXA-239/ OXA-23-like<br>OXA-340/ OXA-51-like<br>OXA-371/ OXA-51-like<br>OXA-402/ OXA-51-like<br>OXA-500/ OXA-213-like<br>OXA-735/ OXA-51-like<br>OXA-834/ OXA-51-like<br>OXA-94/ OXA-51-like<br>OXA-67/ OXA-51-like<br>OXA-70/ OXA-51-like<br>OXA-78/ OXA-51-like<br>OXA-92/ OXA-51-like<br>OXA-104/ OXA-51-like<br>OXA-51/ OXA-51-like<br>OXA-90/ OXA-51-like<br>OXA-82/ OXA-51-like<br>OXA-235/ OXA-134-like<br>OXA-64/ OXA-51-like<br>OXA-98 / OXA-51-like<br>OXA-259/ OXA-51-like<br>OXA-69/ OXA-51-like<br>OXA-65/ OXA-51-like<br>OXA-68/ OXA-51-like<br>OXA-66/ OXA-51-like<br>OXA-23/ OXA-23-like	OXA-164/ OXA-58-like<br>OXA-23/ OXA-23-like<br>OXA-24/ OXA-24-like<br>OXA-235/ OXA-134-like<br>OXA-237/ OXA-134-like<br>OXA-420/ OXA-58-like<br>OXA-437/ OXA-24-like<br>OXA-51/ OXA-51-like<br>OXA-58/ OXA-58-like<br>OXA-72/ OXA-24-like<br>OXA-96/ OXA-58-like	OXA-113/ OXA-51-like<br>OXA-23/ OXA-23-like<br>OXA-235/ OXA-134-like<br>OXA-237/ OXA-134-like OXA-<br>256/ OXA-10-like<br>OXA-259/ OXA-51-like<br>OXA-343/ OXA-51-like<br>OXA-51/ OXA-51-like<br>OXA-66/ OXA-51-like<br>OXA-68/ OXA-51-like<br>OXA-684/ OXA-51-like<br>OXA-69/ OXA-51-like<br>OXA-90/ OXA-51-like
P. aeruginosa	OXA-1/OXA-1-like<br>OXA-10/ OXA-10-like<br>OXA-133/ OXA-23-like<br>OXA-2/ OXA-2-like<br>OXA-395/ OXA-50-like<br>OXA-396/ OXA-10-like<br>OXA-4/ OXA-1-like<br>OXA-486/ OXA-50-like<br>OXA-488/ OXA-50-like<br>OXA-494/ OXA-50-like<br>OXA-50/ OXA-50-like<br>OXA-847/ OXA-50-like<br>OXA-848/ OXA-50-like	OXA-1/OXA-1-like<br>OXA-10/ OXA-10-like<br>OXA-101/ OXA-10-like	OXA-1/OXA-1-like<br>OXA-101/ OXA-10-like<br>OXA-198/ OXA-198-like<br>OXA-21/ OXA-2-like<br>OXA-677/ OXA-10-like<br>OXA-796/OXA-1-like<br>OXA-9/OXA-9-like<br>OXA-901/OXA-50-like<br>OXA-908/OXA-50-like<br>OXA-914/OXA-50-like<br>OXA-129/OXA-5-like<br>OXA-17/ OXA-10-like<br>OXA-35/ OXA-10-like<br>OXA-4/ OXA-1-like<br>OXA-905/OXA-50-like<br>OXA-902/OXA-50-like<br>OXA-904/OXA-50-like

			OXA-2/ OXA-2-like<br>OXA-847/OXA-50-like<br>OXA-851/OXA-50-like<br>OXA-10/OXA-10-like<br>OXA-396/OXA-50-like<br>OXA-846/OXA-50-like<br>OXA-486/OXA-50-like<br>OXA-395/OXA-50-like<br>OXA-494/OXA-50-like<br>OXA-50/OXA-50-like<br>OXA-488/OXA-50-like
Enterobacter spp.	OXA-1/OXA-1-like<br>OXA-2/ OXA-2-like	OXA-48/ OXA-48-like<br>OXA-1/OXA-1-like<br>OXA-129/OXA-5-like<br>OXA-9/OXA-9-like<br>OXA-181/ OXA-48-like<br>OXA-10/OXA-10-like<br>OXA-17/OXA-10-like	**0**

Table S2. a: Target-Template details of selected modeled structures.

Variant Name	C-score	Amino Acid	Template Used
OXA-9-like	-0.96	279 a.a.	6nhu:A, 7oda:A, 6huh, 7kis, 6nio:A
OXA-198-like	-0.02	260 a.a.	6nlw:A, 1nrf:A, 3qnb:A, 5dtk:A, 3hbr:B,
OXA-50-like	-0.49	262 a.a.	4JF4:A, 3hbr:B, 3qnb:A, 7oda:A, 6nlw:A

Table S2. b: Model validation.

Variant Name	Ramachandran Plot Statistics					ERRAT Statistics	Verify 3D Statistics
	Residues in most favorable region	Residues in additional allowed region	Residues in generously allowed region	Residues in disallowed region	Number of non-glycine and non-proline residues	Score	Score
OXA-9-like	158 (65.6%)	64 (26.6%)	12 (5%)	7 (2.9%)	241 (100%)	79.33	84.59%
OXA-198-like	185 (78.7%)	44 (18.7%)	4 (1.7%)	2 (0.9%)	235 (100%)	86.50	91.54%
OXA-50-like	181 (81.9%)	33 (14.9%)	7 (3.2%)	0 (0%)	221 (100%)	95.27	91.60%
