# Supplementary material for: Investigating the OXA Variants of ESKAPE Pathogens
Source: Antibiotics (Basel). 2021 Dec 15;10(12):1539. doi: 10.3390/antibiotics10121539 (PMC8699015; doi:10.3390/antibiotics10121539)
Supplement: Supplementary file 1 [file antibiotics-10-01539-s001.zip › Figure S2.pdf]

OXA-1-like subfamily

```
                                                                         α1         β1
OXA-1-like_BBE55408.1_K.p.
                                  1        10        20        30*       40*
OXA-1-like_BBE55408.1_K.p.   ...............MKNTIHINFAIFLIANIIYSSASASTDISTVASPLFEGTEGCFL
1M6K_beta-lactamase_OXA-1    .......................................STDISTVASPLFEGTEGCFL
OXA-1-like_AVK27274.1_P.a.   ...............MKNTIHINFAIFLIANIIYSSASASTDISTVASPLFEGTEGCFL
OXA-1-like_AWE78522.1_P.a.   ...............MKNTIHINFAIFLIANIIYSSASASTDISTVASPLFEGTEGCFL
OXA-1-like_QLA55054.1_P.a.   ...............MKNTIHINFAIFLIANIIYSSASASTDISTVASPLFEGTEGCFL
OXA-1-like_QKG33210.1_P.a.   ...............MKNTIHINFAIFLIANIIYSSASASTDISTVASPLFEGTEGCFL
OXA-1-like_QOC40829.1_K.p.   ...............MKNTIHINFAIFFIANIIYSSASASTDISTVASPLFEGTEGCFL
OXA-1-like_QLF61683.1_K.p.   ...............MKNTIHINFAIFLIANIIYSSASASTDISTVASPLFEGTEGCFL
OXA-1-like_ANP63432.1_P.a.   ...............MKNTIHINFAIFLIANIIYSSASASTDISTVASPLFEGTEGCFL
OXA-1-like_QII98525.1_P.a.   ...............MKNTIHINFAIFLIANIIYSSASASTDISTVASPLFEGTEGCFL
OXA-1-like_AXL78701.1_P.a.   ...............MKNTIHINFAIFLIANIIYSSASASTDISTVASPLFEGTEGCFL
OXA-1-like_QEL33624.1_E.spp. ...............MKNTIHINFAIFLIANIIYSSASASTDISTVASPLFEGTEGCFL
OXA-1-like_QCZ41047.1_E.spp. ...............MKNTIHINFAIFLIANIIYSSASASTDISTVASPLFEGTEGCFL
OXA-1-like_QPO60228.1_E.spp. ...............MKNTIHINFAIFLIANIIYSSASASTDISTVASPLFEGTEGCFL
OXA-1-like_ATW95033.1_E.spp. ...............MKNTIHINFAIFLIANIIYSSASASTDISTVASPLFEGTEGCFL
OXA-1-like_QBB08468.1_E.spp. ...............MKNTIHINFAIFLIANIIYSSASASTDISTVASPLFEGTEGCFL
OXA-1-like_QFH57730.1_E.spp. ...............MKNTIHINFAIFLIANIIYSSASASTDISTVASPLFEGTEGCFL
OXA-1-like_QFI25362.1_E.spp. ...............MKNTIHINFAIFLIANIIYSSASASTDISTVASPLFEGTEGCFL
OXA-1-like_QHC80210.1_E.spp. ...............MKNTIHINFAIFLIANIIYSSASASTDISTVASPLFEGTEGCFL
OXA-1-like_QMT04262.1_E.spp. ...............MKNTIHINFAIFLIANIIYSSASASTDISTVASPLFEGTEGCFL
OXA-1-like_QPD73921.1_E.spp. ...............MKNTIHINFAIFLIANIIYSSASASTDISTVASPLFEGTEGCFL
OXA-1-like_QPO69359.1_E.spp. ...............MKNTIHINFAIFLIANIIYSSASASTDISTVASPLFEGTEGCFL
OXA-1-like_QPO94996.1_E.spp. ...............MKNTIHINFAIFLIANIIYSSASASTDISTVASPLFEGTEGCFL
OXA-1-like_QQA69926.1_E.spp. ...............MKNTIHINFAIFLIANIIYSSASASTDISTVASPLFEGTEGCFL
OXA-1-like_ASP02997.1_E.spp. ...............MKNTIHINFAIFLIANIIYSSASASTDISTVASPLFEGTEGCFL
OXA-1-like_AOF07832.1_K.p.   ...............MKNTIHINFAIFLIANIIYSSASASTDISTVASPLFEGTEGCFL
OXA-1-like_APD44928.1_K.p.   ...............MKNTIHINFAIFLIANIIYSSASASTDISTVASPLFEGTEGCFL
OXA-1-like_AWF45003.1_K.p.   ...............MKNTIHINFAIFLIANIIYSSASASTDISTVASPLFEGTEGCFL
OXA-1-like_AWX26315.1_K.p.   ...............MKNTIHINFAIFLIANIIYSSASASTDISTVASPLFEGTEGCFL
OXA-1-like_BBE66685.1_K.p.   ...............MKNTIHINFAIFLIANIIYSSASASTDISTVASPLFEGTEGCFL
OXA-1-like_ARA49869.1_K.p.   ...............MKNTIHINFAIFLIANIIYSSASASTDISTVASPLFEGTEGCFL
OXA-1-like_ART03029.1_K.p.   ...............MKNTIHINFAIFLIANIIYSSASASTDISTVASPLFEGTEGCFL
OXA-1-like_ART14043.1_K.p.   ...............MKNTIHINFAIFLIANIIYSSASASTDISTVASPLFEGTEGCFL
OXA-1-like_ATO08041.1_K.p.   ...............MKNTIHINFAIFLIANIIYSSASASTDISTVASPLFEGTEGCFL
OXA-1-like_ATQ97012.1_K.p.   ...............MKNTIHINFAIFLIANIIYSSASASTDISTVASPLFEGTEGCFL
OXA-1-like_AUD20317.1_K.p.   ...............MKNTIHINFAIFLIANIIYSSASASTDISTVASPLFEGTEGCFL
OXA-1-like_AUD31186.1_K.p.   ...............MKNTIHINFAIFLIANIIYSSASASTDISTVASPLFEGTEGCFL
OXA-1-like_AUD36737.1_K.p.   ...............MKNTIHINFAIFLIANIIYSSASASTDISTVASPLFEGTEGCFL
OXA-1-like_AVF14577.1_K.p.   ...............MKNTIHINFAIFLIANIIYSSASASTDISTVASPLFEGTEGCFL
OXA-1-like_AWC05483.1_K.p.   ...............MKNTIHINFAIFLIANIIYSSASASTDISTVASPLFEGTEGCFL
OXA-1-like_QAZ99397.1_K.p.   ...............MKNTIHINFAIFLIANIIYSSASASTDISTVASPLFEGTEGCFL
OXA-1-like_QIF88052.1_K.p.   ...............MKNTIHINFAIFLIANIIYSSASASTDISTVASPLFEGTEGCFL
OXA-1-like_QLF61156.1_K.p.   ...............MKNTIHINFAIFLIANIIYSSASASTDISTVASPLFEGTEGCFL
OXA-1-like_QLF66737.1_K.p.   ...............MKNTIHINFAIFLIANIIYSSASASTDISTVASPLFEGTEGCFL
OXA-1-like_QLG18431.1_K.p.   ...............MKNTIHINFAIFLIANIIYSSASASTDISTVASPLFEGTEGCFL
OXA-1-like_AIT04829.1_K.p.   ...............MKNTIHINFAIFLIANIIYSSASASTDISTVASPLFEGTEGCFL
OXA-1-like_AKE78741.1_K.p.   ...............MKNTIHINFAIFLIANIIYSSASASTDISTVASPLFEGTEGCFL
OXA-1-like_ANK19483.1_K.p.   ...............MKNTIHINFAIFLIANIIYSSASASTDISTVASPLFEGTEGCFL
OXA-1-like_AOF02731.1_K.p.   ...............MKNTIHINFAIFLIANIIYSSASASTDISTVASPLFEGTEGCFL
OXA-1-like_APB53840.1_K.p.   ...............MKNTIHINFAIFLIANIIYSSASASTDISTVASPLFEGTEGCFL
OXA-1-like_APM47234.1_K.p.   ...............MKNTIHINFAIFLIANIIYSSASASTDISTVASPLFEGTEGCFL
OXA-1-like_APM56693.1_K.p.   ...............MKNTIHINFAIFLIANIIYSSASASTDISTVASPLFEGTEGCFL
OXA-1-like_APM62285.1_K.p.   ...............MKNTIHINFAIFLIANIIYSSASASTDISTVASPLFEGTEGCFL
OXA-1-like_APM67903.1_K.p.   ...............MKNTIHINFAIFLIANIIYSSASASTDISTVASPLFEGTEGCFL
OXA-1-like_APP44482.1_K.p.   ...............MKNTIHINFAIFLIANIIYSSASASTDISTVASPLFEGTEGCFL
OXA-1-like_APQ29072.1_K.p.   ...............MKNTIHINFAIFLIANIIYSSASASTDISTVASPLFEGTEGCFL
OXA-1-like_APR51020.1_K.p.   ...............MKNTIHINFAIFLIANIIYSSASASTDISTVASPLFEGTEGCFL
OXA-1-like_ARM26101.1_K.p.   ...............MKNTIHINFAIFLIANIIYSSASASTDISTVASPLFEGTEGCFL
OXA-1-like_ARM26250.1_K.p.   ...............MKNTIHINFAIFLIANIIYSSASASTDISTVASPLFEGTEGCFL
OXA-1-like_ART14424.1_K.p.   ...............MKNTIHINFAIFLIANIIYSSASASTDISTVASPLFEGTEGCFL
OXA-1-like_ARX22868.1_K.p.   ...............MKNTIHINFAIFLIANIIYSSASASTDISTVASPLFEGTEGCFL
OXA-1-like_ARZ96561.1_K.p.   ...............MKNTIHINFAIFLIANIIYSSASASTDISTVASPLFEGTEGCFL
OXA-1-like_ASC31826.1_K.p.   ...............MKNTIHINFAIFLIANIIYSSASASTDISTVASPLFEGTEGCFL
OXA-1-like_QIF55803.1_K.p.   ...............MKNTIHINFAIFLIANIIYSSASASTDISTVASPLFEGTEGCFL
OXA-1-like_QIF55813.1_K.p.   ...............MKNTIHINFAIFLIANIIYSSASASTDISTVASPLFEGTEGCFL
OXA-1-like_VAX91792.1_K.p.   ...............MKNTIHINFAIFLIANIIYSSASASTDISTVASPLFEGTEGCFL
OXA-1-like_VAX91482.1_K.p.   ...............MKNTIHINFAIFLIANIIYSSASASTDISTVASPLFEGTEGCFL
OXA-1-like_QQL36550.1_K.p.   ...............MKNTIHINFAIFLIANIIYSSASASTDISTVASPLFEGTEGCFL
OXA-1-like_QPP73193.1_K.p.   ...............MKNTIHINFAIFLIANIIYSSASASTDISTVASPLFEGTEGCFL
OXA-1-like_QPJ20091.1_K.p.   ...............MKNTIHINFAIFLIANIIYSSASASTDISTVASPLFEGTEGCFL
OXA-1-like_QPB99042.1_K.p.   ...............MKNTIHINFAIFLIANIIYSSASASTDISTVASPLFEGTEGCFL
OXA-1-like_QPA13355.1_K.p.   ...............MKNTIHINFAIFLIANIIYSSASASTDISTVASPLFEGTEGCFL
OXA-1-like_QOU70610.1_K.p.   ...............MKNTIHINFAIFLIANIIYSSASASTDISTVASPLFEGTEGCFL
OXA-1-like_QOU65181.1_K.p.   ...............MKNTIHINFAIFLIANIIYSSASASTDISTVASPLFEGTEGCFL
OXA-1-like_QOU39182.1_K.p.   ...............MKNTIHINFAIFLIANIIYSSASASTDISTVASPLFEGTEGCFL
OXA-1-like_QOT97178.1_K.p.   ...............MKNTIHINFAIFLIANIIYSSASASTDISTVASPLFEGTEGCFL
OXA-1-like_QOD80635.1_K.p.   ...............MKNTIHINFAIFLIANIIYSSASASTDISTVASPLFEGTEGCFL
OXA-1-like_QOD80603.1_K.p.   ...............MKNTIHINFAIFLIANIIYSSASASTDISTVASPLFEGTEGCFL
OXA-1-like_QNB32204.1_K.p.   ...............MKNTIHINFAIFLIANIIYSSASASTDISTVASPLFEGTEGCFL
OXA-1-like_QMS74616.1_K.p.   ...............MKNTIHINFAIFLIANIIYSSASASTDISTVASPLFEGTEGCFL
OXA-1-like_QLQ67472.1_K.p.   ...............MKNTIHINFAIFLIANIIYSSASASTDISTVASPLFEGTEGCFL
OXA-1-like_QLI33436.1_K.p.   ...............MKNTIHINFAIFLIANIIYSSASASTDISTVASPLFEGTEGCFL
OXA-1-like_QLI28063.1_K.p.   ...............MKNTIHINFAIFLIANIIYSSASASTDISTVASPLFEGTEGCFL
OXA-1-like_QLI22583.1_K.p.   ...............MKNTIHINFAIFLIANIIYSSASASTDISTVASPLFEGTEGCFL
OXA-1-like_QLI17177.1_K.p.   ...............MKNTIHINFAIFLIANIIYSSASASTDISTVASPLFEGTEGCFL
OXA-1-like_QLI11731.1_K.p.   ...............MKNTIHINFAIFLIANIIYSSASASTDISTVASPLFEGTEGCFL
OXA-1-like_QLC16052.1_K.p.   ...............MKNTIHINFAIFLIANIIYSSASASTDISTVASPLFEGTEGCFL
OXA-1-like_QLC05373.1_K.p.   ...............MKNTIHINFAIFLIANIIYSSASASTDISTVASPLFEGTEGCFL
OXA-1-like_QLC00088.1_K.p.   ...............MKNTIHINFAIFLIANIIYSSASASTDISTVASPLFEGTEGCFL
OXA-1-like_QLB94808.1_K.p.   ...............MKNTIHINFAIFLIANIIYSSASASTDISTVASPLFEGTEGCFL
OXA-1-like_QLB89468.1_K.p.   ...............MKNTIHINFAIFLIANIIYSSASASTDISTVASPLFEGTEGCFL
OXA-1-like_QLB84130.1_K.p.   ...............MKNTIHINFAIFLIANIIYSSASASTDISTVASPLFEGTEGCFL
OXA-1-like_QLB78789.1_K.p.   ...............MKNTIHINFAIFLIANIIYSSASASTDISTVASPLFEGTEGCFL
OXA-1-like_QLB73465.1_K.p.   ...............MKNTIHINFAIFLIANIIYSSASASTDISTVASPLFEGTEGCFL
OXA-1-like_QIR58461.1_K.p.   ...............MKNTIHINFAIFLIANIIYSSASASTDISTVASPLFEGTEGCFL
OXA-1-like_QHG08101.1_K.p.   ...............MKNTIHINFAIFLIANIIYSSASASTDISTVASPLFEGTEGCFL
OXA-1-like_QGZ85920.1_K.p.   ...............MKNTIHINFAIFLIANIIYSSASASTDISTVASPLFEGTEGCFL
OXA-1-like_QGZ80560.1_K.p.   ...............MKNTIHINFAIFLIANIIYSSASASTDISTVASPLFEGTEGCFL
OXA-1-like_QGT67290.1_K.p.   ...............MKNTIHINFAIFLIANIIYSSASASTDISTVASPLFEGTEGCFL
OXA-1-like_QFY31451.1_K.p.   ...............MKNTIHINFAIFLIANIIYSSASASTDISTVASPLFEGTEGCFL
OXA-1-like_QFH43884.1_K.p.   ...............MKNTIHINFAIFLIANIIYSSASASTDISTVASPLFEGTEGCFL
OXA-1-like_QER51630.1_K.p.   ...............MKNTIHINFAIFLIANIIYSSASASTDISTVASPLFEGTEGCFL
OXA-1-like_QEG84620.1_K.p.   ...............MKNTIHINFAIFLIANIIYSSASASTDISTVASPLFEGTEGCFL
```

OXA-1-like subfamily

```
OXA-1-like_QDZ35954.1_K.p.     ...............MKNTIHINFAIFLIIANIIYSSASASTDISTVASPLFEGTEGCFL
OXA-1-like_QCX07927.1_K.p.     ...............MKNTIHINFAIFLIIANIIYSSASASTDISTVASPLFEGTEGCFL
OXA-1-like_QBH11436.1_K.p.     ...............MKNTIHINFAIFLIIANIIYSSASASTDISTVASPLFEGTEGCFL
OXA-1-like_QBF99632.1_K.p.     ...............MKNTIHINFAIFLIIANIIYSSASASTDISTVASPLFEGTEGCFL
OXA-1-like_QBD88840.1_K.p.     ...............MKNTIHINFAIFLIIANIIYSSASASTDISTVASPLFEGTEGCFL
OXA-1-like_QBA36467.1_K.p.     ...............MKNTIHINFAIFLIIANIIYSSASASTDISTVASPLFEGTEGCFL
OXA-1-like_QAZ94541.1_K.p.     ...............MKNTIHINFAIFLIIANIIYSSASASTDISTVASPLFEGTEGCFL
OXA-1-like_BBE58940.1_K.p.     ...............MKNTIHINFAIFLIIANIIYSSASASTDISTVASPLFEGTEGCFL
OXA-1-like_BBE06733.1_K.p.     ...............MKNTIHINFAIFLIIANIIYSSASASTDISTVASPLFEGTEGCFL
OXA-1-like_AZI07357.1_K.p.     ...............MKNTIHINFAIFLIIANIIYSSASASTDISTVASPLFEGTEGCFL
OXA-1-like_AZH52602.1_K.p.     ...............MKNTIHINFAIFLIIANIIYSSASASTDISTVASPLFEGTEGCFL
OXA-1-like_AZG68296.1_K.p.     ...............MKNTIHINFAIFLIIANIIYSSASASTDISTVASPLFEGTEGCFL
OXA-1-like_AZG62714.1_K.p.     ...............MKNTIHINFAIFLIIANIIYSSASASTDISTVASPLFEGTEGCFL
OXA-1-like_AYD37605.1_K.p.     ...............MKNTIHINFAIFLIIANIIYSSASASTDISTVASPLFEGTEGCFL
OXA-1-like_AXZ62257.1_K.p.     ...............MKNTIHINFAIFLIIANIIYSSASASTDISTVASPLFEGTEGCFL
OXA-1-like_AXZ56762.1_K.p.     ...............MKNTIHINFAIFLIIANIIYSSASASTDISTVASPLFEGTEGCFL
OXA-1-like_AXZ10749.1_K.p.     ...............MKNTIHINFAIFLIIANIIYSSASASTDISTVASPLFEGTEGCFL
OXA-1-like_AWR57732.1_K.p.     ...............MKNTIHINFAIFLIIANIIYSSASASTDISTVASPLFEGTEGCFL
OXA-1-like_AWQ55663.1_K.p.     ...............MKNTIHINFAIFLIIANIIYSSASASTDISTVASPLFEGTEGCFL
OXA-1-like_AWQ55646.1_K.p.     ...............MKNTIHINFAIFLIIANIIYSSASASTDISTVASPLFEGTEGCFL
OXA-1-like_AWQ55645.1_K.p.     ...............MKNTIHINFAIFLIIANIIYSSASASTDISTVASPLFEGTEGCFL
OXA-1-like_AWO49662.1_K.p.     ...............MKNTIHINFAIFLIIANIIYSSASASTDISTVASPLFEGTEGCFL
OXA-1-like_AWO44383.1_K.p.     ...............MKNTIHINFAIFLIIANIIYSSASASTDISTVASPLFEGTEGCFL
OXA-1-like_AWO39120.1_K.p.     ...............MKNTIHINFAIFLIIANIIYSSASASTDISTVASPLFEGTEGCFL
OXA-1-like_AWC00236.1_K.p.     ...............MKNTIHINFAIFLIIANIIYSSASASTDISTVASPLFEGTEGCFL
OXA-1-like_AWA71813.1_K.p.     ...............MKNTIHINFAIFLIIANIIYSSASASTDISTVASPLFEGTEGCFL
OXA-1-like_AVO80920.1_K.p.     ...............MKNTIHINFAIFLIIANIIYSSASASTDISTVASPLFEGTEGCFL
OXA-1-like_AUX60365.1_K.p.     ...............MKNTIHINFAIFLIIANIIYSSASASTDISTVASPLFEGTEGCFL
OXA-1-like_ATR77794.1_K.p.     ...............MKNTIHINFAIFLIIANIIYSSASASTDISTVASPLFEGTEGCFL
OXA-1-like_ATR72263.1_K.p.     ...............MKNTIHINFAIFLIIANIIYSSASASTDISTVASPLFEGTEGCFL
OXA-1-like_ATR66734.1_K.p.     ...............MKNTIHINFAIFLIIANIIYSSASASTDISTVASPLFEGTEGCFL
OXA-1-like_ATR61171.1_K.p.     ...............MKNTIHINFAIFLIIANIIYSSASASTDISTVASPLFEGTEGCFL
OXA-1-like_ATR39902.1_K.p.     ...............MKNTIHINFAIFLIIANIIYSSASASTDISTVASPLFEGTEGCFL
OXA-1-like_ATR34406.1_K.p.     ...............MKNTIHINFAIFLIIANIIYSSASASTDISTVASPLFEGTEGCFL
OXA-1-like_ATR28842.1_K.p.     ...............MKNTIHINFAIFLIIANIIYSSASASTDISTVASPLFEGTEGCFL
OXA-1-like_ATR07462.1_K.p.     ...............MKNTIHINFAIFLIIANIIYSSASASTDISTVASPLFEGTEGCFL
OXA-1-like_ATQ91613.1_K.p.     ...............MKNTIHINFAIFLIIANIIYSSASASTDISTVASPLFEGTEGCFL
OXA-1-like_ATQ27236.1_K.p.     ...............MKNTIHINFAIFLIIANIIYSSASASTDISTVASPLFEGTEGCFL
OXA-1-like_ASC42867.1_K.p.     ...............MKNTIHINFAIFLIIANIIYSSASASTDISTVASPLFEGTEGCFL
OXA-1-like_QKK74875.1_K.p.     MLAVKIKPFTKPILIMKNTIHINFAIFLIIANIIYSSASASTDISTVASPLFEGTEGCFL
OXA-1-like_ATM40508.1_K.p.     ...............MKNTIHINFAIFLIIANIIYSSASASTDISTVASPLFEGTEGCFL
OXA-1-like_ATM68467.1_K.p.     ...............MKNTIHINFAIFLIIANIIYSSASASTDISTVASPLFEGTEGCFL
OXA-1-like_ASC37513.1_K.p.     ...............MKNTIHINFAIFLIIANIIYSSASASTDISTVASPLFEGTEGCFL
OXA-1-like_AQU64164.1_K.p.     MLAVKIKPFTKPILIMKNTIHINFAIFLIIANIIYSSASASTDISTVASPLFEGTEGCFL
OXA-1-like_AQU64317.1_K.p.     MLAVKIKPFTKPILIMKNTIHINFAIFLIIANIIYSSASASTDISTVASPLFEGTEGCFL
OXA-1-like_APV28290.1_K.p.     MLAVKIKPFTKPILIMKNTIHINFAIFLIIANIIYSSASASTDISTVASPLFEGTEGCFL
OXA-1-like_APV22515.1_K.p.     MLAVKIKPFTKPILIMKNTIHINFAIFLIIANIIYSSASASTDISTVASPLFEGTEGCFL
OXA-1-like_ANF44129.1_K.p.     MLAVKIKPFTKPILIMKNTIHINFAIFLIIANIIYSSASASTDISTVASPLFEGTEGCFL
OXA-1-like_AIG86468.1_K.p.     MLAVKIKPFTKPILIMKNTIHINFAIFLIIANIIYSSASASTDISTVASPLFEGTEGCFL
OXA-1-like_AHI38983.1_K.p.     MLAVKIKPFTKPILIMKNTIHINFAIFLIIANIIYSSASASTDISTVASPLFEGTEGCFL
OXA-1-like_QCP74266.1_P.a.     ...............MKNTIHINFAIFLIIANIIYSSASASTDISTVASPLFEGTEGCFL
OXA-1-like_QDE47052.1_E.spp.   ...............MKNTIHINFAIFLIIANIIYSSASASTDISTVASPLFEGTEGCFL
                          acc
```

OXA-1-like subfamily

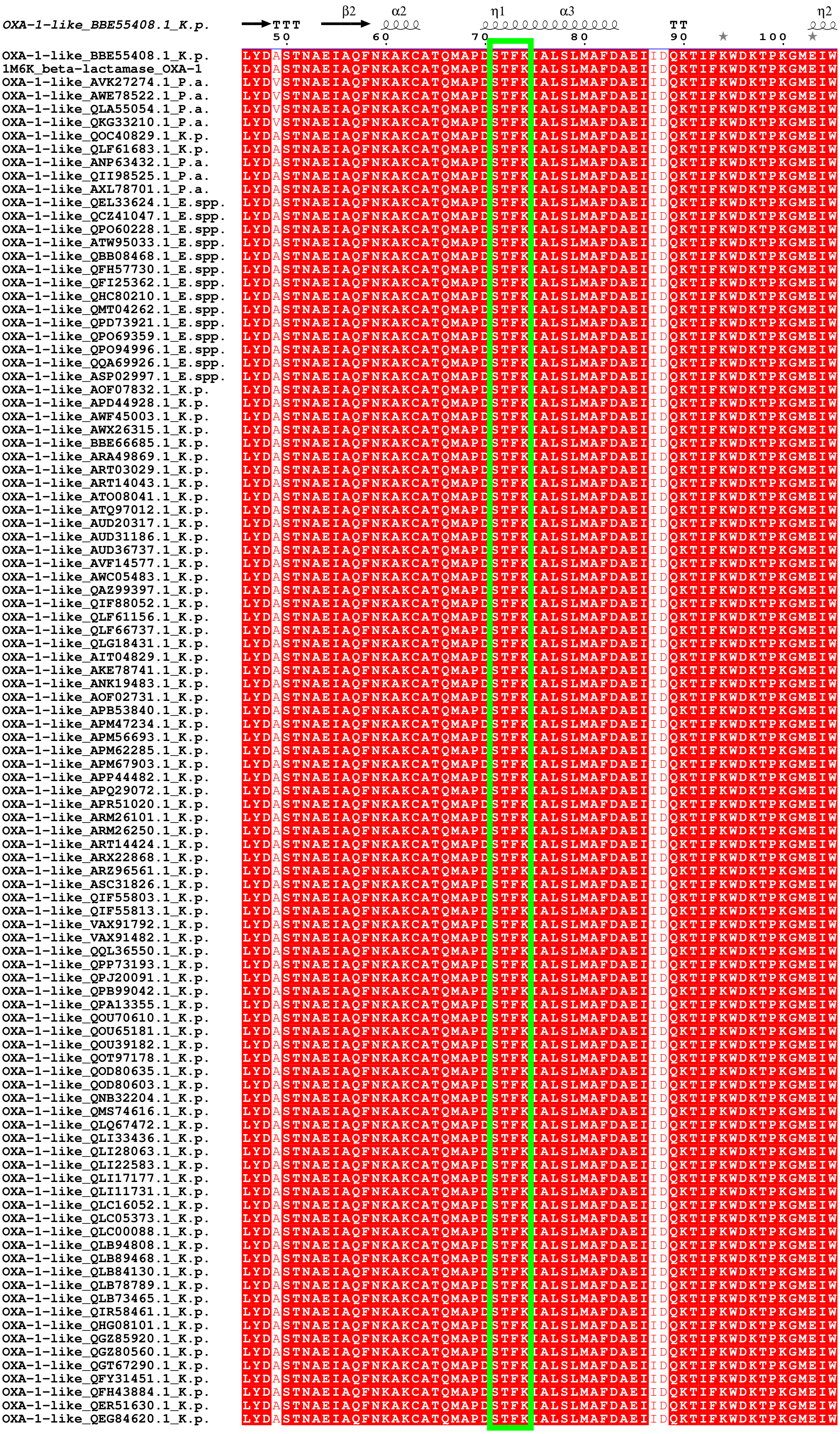

OXA-1-like subfamily

```
OXA-1-like_QDZ35954.1_K.p.    LYDASTNAEIAQFNKAKCATQMAPDSTFKIALSLMAFDAEIIDQKTIFKWDKTPKGMEIW
OXA-1-like_QCX07927.1_K.p.    LYDASTNAEIAQFNKAKCATQMAPDSTFKIALSLMAFDAEIIDQKTIFKWDKTPKGMEIW
OXA-1-like_QBH11436.1_K.p.    LYDASTNAEIAQFNKAKCATQMAPDSTFKIALSLMAFDAEIIDQKTIFKWDKTPKGMEIW
OXA-1-like_QBF99632.1_K.p.    LYDASTNAEIAQFNKAKCATQMAPDSTFKIALSLMAFDAEIIDQKTIFKWDKTPKGMEIW
OXA-1-like_QBD88840.1_K.p.    LYDASTNAEIAQFNKAKCATQMAPDSTFKIALSLMAFDAEIIDQKTIFKWDKTPKGMEIW
OXA-1-like_QBA36467.1_K.p.    LYDASTNAEIAQFNKAKCATQMAPDSTFKIALSLMAFDAEIIDQKTIFKWDKTPKGMEIW
OXA-1-like_QAZ94541.1_K.p.    LYDASTNAEIAQFNKAKCATQMAPDSTFKIALSLMAFDAEIIDQKTIFKWDKTPKGMEIW
OXA-1-like_BBE58940.1_K.p.    LYDASTNAEIAQFNKAKCATQMAPDSTFKIALSLMAFDAEIIDQKTIFKWDKTPKGMEIW
OXA-1-like_BBE06733.1_K.p.    LYDASTNAEIAQFNKAKCATQMAPDSTFKIALSLMAFDAEIIDQKTIFKWDKTPKGMEIW
OXA-1-like_AZI07357.1_K.p.    LYDASTNAEIAQFNKAKCATQMAPDSTFKIALSLMAFDAEIIDQKTIFKWDKTPKGMEIW
OXA-1-like_AZH52602.1_K.p.    LYDASTNAEIAQFNKAKCATQMAPDSTFKIALSLMAFDAEIIDQKTIFKWDKTPKGMEIW
OXA-1-like_AZG68296.1_K.p.    LYDASTNAEIAQFNKAKCATQMAPDSTFKIALSLMAFDAEIIDQKTIFKWDKTPKGMEIW
OXA-1-like_AZG62714.1_K.p.    LYDASTNAEIAQFNKAKCATQMAPDSTFKIALSLMAFDAEIIDQKTIFKWDKTPKGMEIW
OXA-1-like_AYD37605.1_K.p.    LYDASTNAEIAQFNKAKCATQMAPDSTFKIALSLMAFDAEIIDQKTIFKWDKTPKGMEIW
OXA-1-like_AXZ62257.1_K.p.    LYDASTNAEIAQFNKAKCATQMAPDSTFKIALSLMAFDAEIIDQKTIFKWDKTPKGMEIW
OXA-1-like_AXZ56762.1_K.p.    LYDASTNAEIAQFNKAKCATQMAPDSTFKIALSLMAFDAEIIDQKTIFKWDKTPKGMEIW
OXA-1-like_AXZ10749.1_K.p.    LYDASTNAEIAQFNKAKCATQMAPDSTFKIALSLMAFDAEIIDQKTIFKWDKTPKGMEIW
OXA-1-like_AWR57732.1_K.p.    LYDASTNAEIAQFNKAKCATQMAPDSTFKIALSLMAFDAEIIDQKTIFKWDKTPKGMEIW
OXA-1-like_AWQ55663.1_K.p.    LYDASTNAEIAQFNKAKCATQMAPDSTFKIALSLMAFDAEIIDQKTIFKWDKTPKGMEIW
OXA-1-like_AWQ55646.1_K.p.    LYDASTNAEIAQFNKAKCATQMAPDSTFKIALSLMAFDAEIIDQKTIFKWDKTPKGMEIW
OXA-1-like_AWQ55645.1_K.p.    LYDASTNAEIAQFNKAKCATQMAPDSTFKIALSLMAFDAEIIDQKTIFKWDKTPKGMEIW
OXA-1-like_AWO49662.1_K.p.    LYDASTNAEIAQFNKAKCATQMAPDSTFKIALSLMAFDAEIIDQKTIFKWDKTPKGMEIW
OXA-1-like_AWO44383.1_K.p.    LYDASTNAEIAQFNKAKCATQMAPDSTFKIALSLMAFDAEIIDQKTIFKWDKTPKGMEIW
OXA-1-like_AWO39120.1_K.p.    LYDASTNAEIAQFNKAKCATQMAPDSTFKIALSLMAFDAEIIDQKTIFKWDKTPKGMEIW
OXA-1-like_AWC00236.1_K.p.    LYDASTNAEIAQFNKAKCATQMAPDSTFKIALSLMAFDAEIIDQKTIFKWDKTPKGMEIW
OXA-1-like_AWA71813.1_K.p.    LYDASTNAEIAQFNKAKCATQMAPDSTFKIALSLMAFDAEIIDQKTIFKWDKTPKGMEIW
OXA-1-like_AVO80920.1_K.p.    LYDASTNAEIAQFNKAKCATQMAPDSTFKIALSLMAFDAEIIDQKTIFKWDKTPKGMEIW
OXA-1-like_AUX60365.1_K.p.    LYDASTNAEIAQFNKAKCATQMAPDSTFKIALSLMAFDAEIIDQKTIFKWDKTPKGMEIW
OXA-1-like_ATR77794.1_K.p.    LYDASTNAEIAQFNKAKCATQMAPDSTFKIALSLMAFDAEIIDQKTIFKWDKTPKGMEIW
OXA-1-like_ATR72263.1_K.p.    LYDASTNAEIAQFNKAKCATQMAPDSTFKIALSLMAFDAEIIDQKTIFKWDKTPKGMEIW
OXA-1-like_ATR66734.1_K.p.    LYDASTNAEIAQFNKAKCATQMAPDSTFKIALSLMAFDAEIIDQKTIFKWDKTPKGMEIW
OXA-1-like_ATR61171.1_K.p.    LYDASTNAEIAQFNKAKCATQMAPDSTFKIALSLMAFDAEIIDQKTIFKWDKTPKGMEIW
OXA-1-like_ATR39902.1_K.p.    LYDASTNAEIAQFNKAKCATQMAPDSTFKIALSLMAFDAEIIDQKTIFKWDKTPKGMEIW
OXA-1-like_ATR34406.1_K.p.    LYDASTNAEIAQFNKAKCATQMAPDSTFKIALSLMAFDAEIIDQKTIFKWDKTPKGMEIW
OXA-1-like_ATR28842.1_K.p.    LYDASTNAEIAQFNKAKCATQMAPDSTFKIALSLMAFDAEIIDQKTIFKWDKTPKGMEIW
OXA-1-like_ATR07462.1_K.p.    LYDASTNAEIAQFNKAKCATQMAPDSTFKIALSLMAFDAEIIDQKTIFKWDKTPKGMEIW
OXA-1-like_ATQ91613.1_K.p.    LYDASTNAEIAQFNKAKCATQMAPDSTFKIALSLMAFDAEIIDQKTIFKWDKTPKGMEIW
OXA-1-like_ATQ27236.1_K.p.    LYDASTNAEIAQFNKAKCATQMAPDSTFKIALSLMAFDAEIIDQKTIFKWDKTPKGMEIW
OXA-1-like_ASC42867.1_K.p.    LYDASTNAEIAQFNKAKCATQMAPDSTFKIALSLMAFDAEIIDQKTIFKWDKTPKGMEIW
OXA-1-like_QKK74875.1_K.p.    LYDASTNAEIAQFNKAKCATQMAPDSTFKIALSLMAFDAEIIDQKTIFKWDKTPKGMEIW
OXA-1-like_ATM40508.1_K.p.    LYDASTNAEIAQFNKAKCATQMAPDSTFKIALSLMAFDAEIIDQKTIFKWDKTPKGMEIW
OXA-1-like_ATM68467.1_K.p.    LYDASTNAEIAQFNKAKCATQMAPDSTFKIALSLMAFDAEIIDQKTIFKWDKTPKGMEIW
OXA-1-like_ASC37513.1_K.p.    LYDASTNAEIAQFNKAKCATQMAPDSTFKIALSLMAFDAEIIDQKTIFKWDKTPKGMEIW
OXA-1-like_AQU64164.1_K.p.    LYDASTNAEIAQFNKAKCATQMAPDSTFKIALSLMAFDAEIIDQKTIFKWDKTPKGMEIW
OXA-1-like_AQU64317.1_K.p.    LYDASTNAEIAQFNKAKCATQMAPDSTFKIALSLMAFDAEIIDQKTIFKWDKTPKGMEIW
OXA-1-like_APV28290.1_K.p.    LYDASTNAEIAQFNKAKCATQMAPDSTFKIALSLMAFDAEIIDQKTIFKWDKTPKGMEIW
OXA-1-like_APV22515.1_K.p.    LYDASTNAEIAQFNKAKCATQMAPDSTFKIALSLMAFDAEIIDQKTIFKWDKTPKGMEIW
OXA-1-like_ANF44129.1_K.p.    LYDASTNAEIAQFNKAKCATQMAPDSTFKIALSLMAFDAEIIDQKTIFKWDKTPKGMEIW
OXA-1-like_AIG86468.1_K.p.    LYDASTNAEIAQFNKAKCATQMAPDSTFKIALSLMAFDAEIIDQKTIFKWDKTPKGMEIW
OXA-1-like_AHI38983.1_K.p.    LYDASTNAEIAQFNKAKCATQMAPDSTFKIALSLMAFDAEIIDQKTIFKWDKTPKGMEIW
OXA-1-like_QCP74266.1_P.a.    LYDASTNAEIAQFNKAKCATQMAPDSTFKIALSLMAFDAEIIDQKTIFKWDKTPKGMEIW
OXA-1-like_QDE47052.1_E.spp.  LYDASTNAEIAQFNKAKCATQMAPDSTFKIALSLMAFDAEIMNQKTIFKWDKTPKGMEIW
                         acc
```

OXA-1-like subfamily

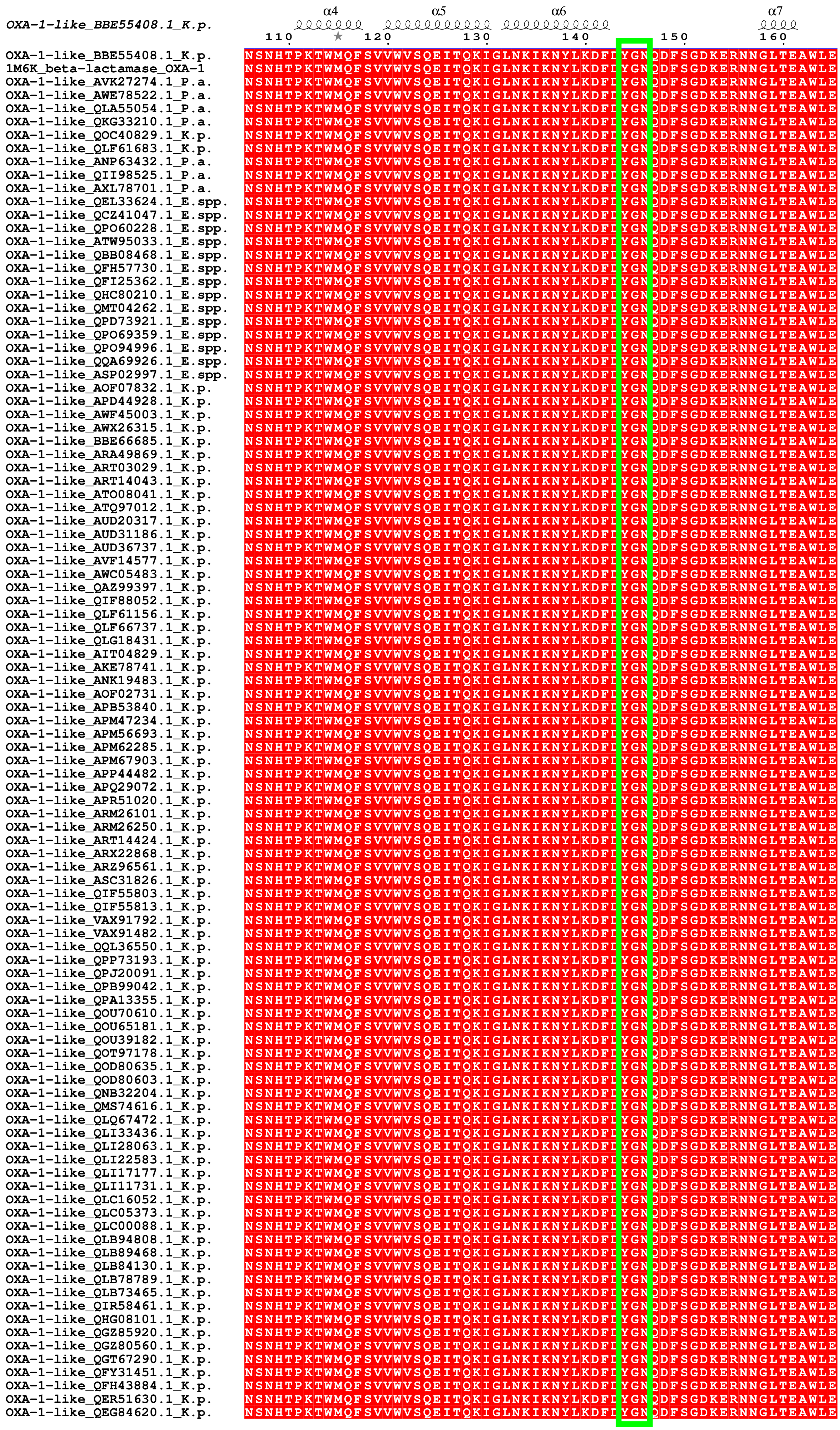

OXA-1-like subfamily

```
OXA-1-like_QDZ35954.1_K.p.    NSNHTPKTWMQFSVVWVSQEITQKIGLNKIKNYLKDFDYGNQDFSGDKERNNGLTEAWLE
OXA-1-like_QCX07927.1_K.p.    NSNHTPKTWMQFSVVWVSQEITQKIGLNKIKNYLKDFDYGNQDFSGDKERNNGLTEAWLE
OXA-1-like_QBH11436.1_K.p.    NSNHTPKTWMQFSVVWVSQEITQKIGLNKIKNYLKDFDYGNQDFSGDKERNNGLTEAWLE
OXA-1-like_QBF99632.1_K.p.    NSNHTPKTWMQFSVVWVSQEITQKIGLNKIKNYLKDFDYGNQDFSGDKERNNGLTEAWLE
OXA-1-like_QBD88840.1_K.p.    NSNHTPKTWMQFSVVWVSQEITQKIGLNKIKNYLKDFDYGNQDFSGDKERNNGLTEAWLE
OXA-1-like_QBA36467.1_K.p.    NSNHTPKTWMQFSVVWVSQEITQKIGLNKIKNYLKDFDYGNQDFSGDKERNNGLTEAWLE
OXA-1-like_QAZ94541.1_K.p.    NSNHTPKTWMQFSVVWVSQEITQKIGLNKIKNYLKDFDYGNQDFSGDKERNNGLTEAWLE
OXA-1-like_BBE58940.1_K.p.    NSNHTPKTWMQFSVVWVSQEITQKIGLNKIKNYLKDFDYGNQDFSGDKERNNGLTEAWLE
OXA-1-like_BBE06733.1_K.p.    NSNHTPKTWMQFSVVWVSQEITQKIGLNKIKNYLKDFDYGNQDFSGDKERNNGLTEAWLE
OXA-1-like_AZI07357.1_K.p.    NSNHTPKTWMQFSVVWVSQEITQKIGLNKIKNYLKDFDYGNQDFSGDKERNNGLTEAWLE
OXA-1-like_AZH52602.1_K.p.    NSNHTPKTWMQFSVVWVSQEITQKIGLNKIKNYLKDFDYGNQDFSGDKERNNGLTEAWLE
OXA-1-like_AZG68296.1_K.p.    NSNHTPKTWMQFSVVWVSQEITQKIGLNKIKNYLKDFDYGNQDFSGDKERNNGLTEAWLE
OXA-1-like_AZG62714.1_K.p.    NSNHTPKTWMQFSVVWVSQEITQKIGLNKIKNYLKDFDYGNQDFSGDKERNNGLTEAWLE
OXA-1-like_AYD37605.1_K.p.    NSNHTPKTWMQFSVVWVSQEITQKIGLNKIKNYLKDFDYGNQDFSGDKERNNGLTEAWLE
OXA-1-like_AXZ62257.1_K.p.    NSNHTPKTWMQFSVVWVSQEITQKIGLNKIKNYLKDFDYGNQDFSGDKERNNGLTEAWLE
OXA-1-like_AXZ56762.1_K.p.    NSNHTPKTWMQFSVVWVSQEITQKIGLNKIKNYLKDFDYGNQDFSGDKERNNGLTEAWLE
OXA-1-like_AXZ10749.1_K.p.    NSNHTPKTWMQFSVVWVSQEITQKIGLNKIKNYLKDFDYGNQDFSGDKERNNGLTEAWLE
OXA-1-like_AWR57732.1_K.p.    NSNHTPKTWMQFSVVWVSQEITQKIGLNKIKNYLKDFDYGNQDFSGDKERNNGLTEAWLE
OXA-1-like_AWQ55663.1_K.p.    NSNHTPKTWMQFSVVWVSQEITQKIGLNKIKNYLKDFDYGNQDFSGDKERNNGLTEAWLE
OXA-1-like_AWQ55646.1_K.p.    NSNHTPKTWMQFSVVWVSQEITQKIGLNKIKNYLKDFDYGNQDFSGDKERNNGLTEAWLE
OXA-1-like_AWQ55645.1_K.p.    NSNHTPKTWMQFSVVWVSQEITQKIGLNKIKNYLKDFDYGNQDFSGDKERNNGLTEAWLE
OXA-1-like_AWO49662.1_K.p.    NSNHTPKTWMQFSVVWVSQEITQKIGLNKIKNYLKDFDYGNQDFSGDKERNNGLTEAWLE
OXA-1-like_AWO44383.1_K.p.    NSNHTPKTWMQFSVVWVSQEITQKIGLNKIKNYLKDFDYGNQDFSGDKERNNGLTEAWLE
OXA-1-like_AWO39120.1_K.p.    NSNHTPKTWMQFSVVWVSQEITQKIGLNKIKNYLKDFDYGNQDFSGDKERNNGLTEAWLE
OXA-1-like_AWC00236.1_K.p.    NSNHTPKTWMQFSVVWVSQEITQKIGLNKIKNYLKDFDYGNQDFSGDKERNNGLTEAWLE
OXA-1-like_AWA71813.1_K.p.    NSNHTPKTWMQFSVVWVSQEITQKIGLNKIKNYLKDFDYGNQDFSGDKERNNGLTEAWLE
OXA-1-like_AVO80920.1_K.p.    NSNHTPKTWMQFSVVWVSQEITQKIGLNKIKNYLKDFDYGNQDFSGDKERNNGLTEAWLE
OXA-1-like_AUX60365.1_K.p.    NSNHTPKTWMQFSVVWVSQEITQKIGLNKIKNYLKDFDYGNQDFSGDKERNNGLTEAWLE
OXA-1-like_ATR77794.1_K.p.    NSNHTPKTWMQFSVVWVSQEITQKIGLNKIKNYLKDFDYGNQDFSGDKERNNGLTEAWLE
OXA-1-like_ATR72263.1_K.p.    NSNHTPKTWMQFSVVWVSQEITQKIGLNKIKNYLKDFDYGNQDFSGDKERNNGLTEAWLE
OXA-1-like_ATR66734.1_K.p.    NSNHTPKTWMQFSVVWVSQEITQKIGLNKIKNYLKDFDYGNQDFSGDKERNNGLTEAWLE
OXA-1-like_ATR61171.1_K.p.    NSNHTPKTWMQFSVVWVSQEITQKIGLNKIKNYLKDFDYGNQDFSGDKERNNGLTEAWLE
OXA-1-like_ATR39902.1_K.p.    NSNHTPKTWMQFSVVWVSQEITQKIGLNKIKNYLKDFDYGNQDFSGDKERNNGLTEAWLE
OXA-1-like_ATR34406.1_K.p.    NSNHTPKTWMQFSVVWVSQEITQKIGLNKIKNYLKDFDYGNQDFSGDKERNNGLTEAWLE
OXA-1-like_ATR28842.1_K.p.    NSNHTPKTWMQFSVVWVSQEITQKIGLNKIKNYLKDFDYGNQDFSGDKERNNGLTEAWLE
OXA-1-like_ATR07462.1_K.p.    NSNHTPKTWMQFSVVWVSQEITQKIGLNKIKNYLKDFDYGNQDFSGDKERNNGLTEAWLE
OXA-1-like_ATQ91613.1_K.p.    NSNHTPKTWMQFSVVWVSQEITQKIGLNKIKNYLKDFDYGNQDFSGDKERNNGLTEAWLE
OXA-1-like_ATQ27236.1_K.p.    NSNHTPKTWMQFSVVWVSQEITQKIGLNKIKNYLKDFDYGNQDFSGDKERNNGLTEAWLE
OXA-1-like_ASC42867.1_K.p.    NSNHTPKTWMQFSVVWVSQEITQKIGLNKIKNYLKDFDYGNQDFSGDKERNNGLTEAWLE
OXA-1-like_QKK74875.1_K.p.    NSNHTPKTWMQFSVVWVSQEITQKIGLNKIKNYLKDFDYGNQDFSGDKERNNGLTEAWLE
OXA-1-like_ATM40508.1_K.p.    NSNHTPKTWMQFSVVWVSQEITQKIGLNKIKNYLKDFDYGNQDFSGDKERNNGLTEAWLE
OXA-1-like_ATM68467.1_K.p.    NSNHTPKTWMQFSVVWVSQEITQKIGLNKIKNYLKDFDYGNQDFSGDKERNNGLTEAWLE
OXA-1-like_ASC37513.1_K.p.    NSNHTPKTWMQFSVVWVSQEITQKIGLNKIKNYLKDFDYGNQDFSGDKERNNGLTEAWLE
OXA-1-like_AQU64164.1_K.p.    NSNHTPKTWMQFSVVWVSQEITQKIGLNKIKNYLKDFDYGNQDFSGDKERNNGLTEAWLE
OXA-1-like_AQU64317.1_K.p.    NSNHTPKTWMQFSVVWVSQEITQKIGLNKIKNYLKDFDYGNQDFSGDKERNNGLTEAWLE
OXA-1-like_APV28290.1_K.p.    NSNHTPKTWMQFSVVWVSQEITQKIGLNKIKNYLKDFDYGNQDFSGDKERNNGLTEAWLE
OXA-1-like_APV22515.1_K.p.    NSNHTPKTWMQFSVVWVSQEITQKIGLNKIKNYLKDFDYGNQDFSGDKERNNGLTEAWLE
OXA-1-like_ANF44129.1_K.p.    NSNHTPKTWMQFSVVWVSQEITQKIGLNKIKNYLKDFDYGNQDFSGDKERNNGLTEAWLE
OXA-1-like_AIG86468.1_K.p.    NSNHTPKTWMQFSVVWVSQEITQKIGLNKIKNYLKDFDYGNQDFSGDKERNNGLTEAWLE
OXA-1-like_AHI38983.1_K.p.    NSNHTPKTWMQFSVVWVSQEITQKIGLNKIKNYLKDFDYGNQDFSGDKERNNGLTEAWLE
OXA-1-like_QCP74266.1_P.a.    NSNHTPKTWMQFSVVWVSQEITQKIGLNKIKNYLKDFDYGNQDFSGDKERNNGLTEAWLE
OXA-1-like_QDE47052.1_E.spp.  NSNHTPKTWMQFSVVWVSQEITQKIGLNKIKNYLKDFDYGNQDFSGDKERNNGLTEAWLE
                         acc
```

OXA-1-like subfamily

```
OXA-1-like_BBE55408.1_K.p.   α8 (170–180), α9 (190–200), β3, TT, β4 (210–220), TT

OXA-1-like_BBE55408.1_K.p.    SSLKISPEEQIQFLRKIINHNLPVKNSAIEN.TIENMYLQDLDNSTKLYGKTGAGFTANR
1M6K_beta-lactamase_OXA-1     SSLKISPEEQIQFLRKIINHNLPVKNSAIEN.TIENMYLQDLDNSTKLYGKTGAGFTANR
OXA-1-like_AVK27274.1_P.a.    SSLKISPEEQIQFLRKIINHNLPVKNSAIEN.TIENMYLQDLENSTKLYGKTGAGFTANR
OXA-1-like_AWE78522.1_P.a.    SSLKISPEEQIQFLRKIINHNLPVKNSAIEN.TIENMYLQDLENSTKLYGKTGAGFTANR
OXA-1-like_QLA55054.1_P.a.    SSLKISPEEQIQFLRKIINHNLPVKNSAIEN.TIENMYLQDLENSTKLYGKTGAGFTANR
OXA-1-like_QKG33210.1_P.a.    SSLKISPEEQIQFLRKIINHNLPVKNSAIEN.TIENMYLQDLDNSTKLYGKTGAGFTANR
OXA-1-like_QOC40829.1_K.p.    SSLKISPEEQIQFLRKIINHNLPVKNSAIEN.TIENMYLQDLDNSTKLYGKTGAGFTANR
OXA-1-like_QLF61683.1_K.p.    SSLKISPEEQIQFLRKIINHNLPVKNSAIEENTIENMYLQDLDNSTKLYGKTGAGFTANR
OXA-1-like_ANP63432.1_P.a.    SSLKISPEEQIQFLRKIINHNLPVKNSAIEN.TIENMYLQDLDNSTKLYGKTGAGFTANR
OXA-1-like_QII98525.1_P.a.    SSLKISPEEQIQFLRKIINHNLPVKNSAIEN.TIENMYLQDLDNSTKLYGKTGAGFTANR
OXA-1-like_AXL78701.1_P.a.    SSLKISPEEQIQFLRKIINHNLPVKNSAIEN.TIENMYLQDLDNSTKLYGKTGAGFTANR
OXA-1-like_QEL33624.1_E.spp.  SSLKISPEEQIQFLRKIINHNLPVKNSAIEN.TIENMYLQDLDNSTKLYGKTGAGFTANR
OXA-1-like_QCZ41047.1_E.spp.  SSLKISPEEQIQFLRKIINHNLPVKNSAIEN.TIENMYLQDLDNSTKLYGKTGAGFTANR
OXA-1-like_QPO60228.1_E.spp.  SSLKISPEEQIQFLRKIINHNLPVKNSAIEN.TIENMYLQDLDNSTKLYGKTGAGFTANR
OXA-1-like_ATW95033.1_E.spp.  SSLKISPEEQIQFLRKIINHNLPVKNSAIEN.TIENMYLQDLDNSTKLYGKTGAGFTANR
OXA-1-like_QBB08468.1_E.spp.  SSLKISPEEQIQFLRKIINHNLPVKNSAIEN.TIENMYLQDLDNSTKLYGKTGAGFTANR
OXA-1-like_QFH57730.1_E.spp.  SSLKISPEEQIQFLRKIINHNLPVKNSAIEN.TIENMYLQDLDNSTKLYGKTGAGFTANR
OXA-1-like_QFI25362.1_E.spp.  SSLKISPEEQIQFLRKIINHNLPVKNSAIEN.TIENMYLQDLDNSTKLYGKTGAGFTANR
OXA-1-like_QHC80210.1_E.spp.  SSLKISPEEQIQFLRKIINHNLPVKNSAIEN.TIENMYLQDLDNSTKLYGKTGAGFTANR
OXA-1-like_QMT04262.1_E.spp.  SSLKISPEEQIQFLRKIINHNLPVKNSAIEN.TIENMYLQDLDNSTKLYGKTGAGFTANR
OXA-1-like_QPD73921.1_E.spp.  SSLKISPEEQIQFLRKIINHNLPVKNSAIEN.TIENMYLQDLDNSTKLYGKTGAGFTANR
OXA-1-like_QPO69359.1_E.spp.  SSLKISPEEQIQFLRKIINHNLPVKNSAIEN.TIENMYLQDLDNSTKLYGKTGAGFTANR
OXA-1-like_QPO94996.1_E.spp.  SSLKISPEEQIQFLRKIINHNLPVKNSAIEN.TIENMYLQDLDNSTKLYGKTGAGFTANR
OXA-1-like_QQA69926.1_E.spp.  SSLKISPEEQIQFLRKIINHNLPVKNSAIEN.TIENMYLQDLDNSTKLYGKTGAGFTANR
OXA-1-like_ASP02997.1_E.spp.  SSLKISPEEQIQFLRKIINHNLPVKNSAIEN.TIENMYLQDLDNSTKLYGKTGAGFTANR
OXA-1-like_AOF07832.1_K.p.    SSLKISPEEQIQFLRKIINHNLPVKNSAIEN.TIENMYLQDLDNSTKLYGKTGAGFTANR
OXA-1-like_APD44928.1_K.p.    SSLKISPEEQIQFLRKIINHNLPVKNSAIEN.TIENMYLQDLDNSTKLYGKTGAGFTANR
OXA-1-like_AWF45003.1_K.p.    SSLKISPEEQIQFLRKIINHNLPVKNSAIEN.TIENMYLQDLDNSTKLYGKTGAGFTANR
OXA-1-like_AWX26315.1_K.p.    SSLKISPEEQIQFLRKIINHNLPVKNSAIEN.TIENMYLQDLDNSTKLYGKTGAGFTANR
OXA-1-like_BBE66685.1_K.p.    SSLKISPEEQIQFLRKIINHNLPVKNSAIEN.TIENMYLQDLDNSTKLYGKTGAGFTANR
OXA-1-like_ARA49869.1_K.p.    SSLKISPEEQIQFLRKIINHNLPVKNSAIEN.TIENMYLQDLDNSTKLYGKTGAGFTANR
OXA-1-like_ART03029.1_K.p.    SSLKISPEEQIQFLRKIINHNLPVKNSAIEN.TIENMYLQDLDNSTKLYGKTGAGFTANR
OXA-1-like_ART14043.1_K.p.    SSLKISPEEQIQFLRKIINHNLPVKNSAIEN.TIENMYLQDLDNSTKLYGKTGAGFTANR
OXA-1-like_ATO08041.1_K.p.    SSLKISPEEQIQFLRKIINHNLPVKNSAIEN.TIENMYLQDLDNSTKLYGKTGAGFTANR
OXA-1-like_ATQ97012.1_K.p.    SSLKISPEEQIQFLRKIINHNLPVKNSAIEN.TIENMYLQDLDNSTKLYGKTGAGFTANR
OXA-1-like_AUD20317.1_K.p.    SSLKISPEEQIQFLRKIINHNLPVKNSAIEN.TIENMYLQDLDNSTKLYGKTGAGFTANR
OXA-1-like_AUD31186.1_K.p.    SSLKISPEEQIQFLRKIINHNLPVKNSAIEN.TIENMYLQDLDNSTKLYGKTGAGFTANR
OXA-1-like_AUD36737.1_K.p.    SSLKISPEEQIQFLRKIINHNLPVKNSAIEN.TIENMYLQDLDNSTKLYGKTGAGFTANR
OXA-1-like_AVF14577.1_K.p.    SSLKISPEEQIQFLRKIINHNLPVKNSAIEN.TIENMYLQDLDNSTKLYGKTGAGFTANR
OXA-1-like_AWC05483.1_K.p.    SSLKISPEEQIQFLRKIINHNLPVKNSAIEN.TIENMYLQDLDNSTKLYGKTGAGFTANR
OXA-1-like_QAZ99397.1_K.p.    SSLKISPEEQIQFLRKIINHNLPVKNSAIEN.TIENMYLQDLDNSTKLYGKTGAGFTANR
OXA-1-like_QIF88052.1_K.p.    SSLKISPEEQIQFLRKIINHNLPVKNSAIEN.TIENMYLQDLDNSTKLYGKTGAGFTANR
OXA-1-like_QLF61156.1_K.p.    SSLKISPEEQIQFLRKIINHNLPVKNSAIEN.TIENMYLQDLDNSTKLYGKTGAGFTANR
OXA-1-like_QLF66737.1_K.p.    SSLKISPEEQIQFLRKIINHNLPVKNSAIEN.TIENMYLQDLDNSTKLYGKTGAGFTANR
OXA-1-like_QLG18431.1_K.p.    SSLKISPEEQIQFLRKIINHNLPVKNSAIEN.TIENMYLQDLDNSTKLYGKTGAGFTANR
OXA-1-like_AIT04829.1_K.p.    SSLKISPEEQIQFLRKIINHNLPVKNSAIEN.TIENMYLQDLDNSTKLYGKTGAGFTANR
OXA-1-like_AKE78741.1_K.p.    SSLKISPEEQIQFLRKIINHNLPVKNSAIEN.TIENMYLQDLDNSTKLYGKTGAGFTANR
OXA-1-like_ANK19483.1_K.p.    SSLKISPEEQIQFLRKIINHNLPVKNSAIEN.TIENMYLQDLDNSTKLYGKTGAGFTANR
OXA-1-like_AOF02731.1_K.p.    SSLKISPEEQIQFLRKIINHNLPVKNSAIEN.TIENMYLQDLDNSTKLYGKTGAGFTANR
OXA-1-like_APB53840.1_K.p.    SSLKISPEEQIQFLRKIINHNLPVKNSAIEN.TIENMYLQDLDNSTKLYGKTGAGFTANR
OXA-1-like_APM47234.1_K.p.    SSLKISPEEQIQFLRKIINHNLPVKNSAIEN.TIENMYLQDLDNSTKLYGKTGAGFTANR
OXA-1-like_APM56693.1_K.p.    SSLKISPEEQIQFLRKIINHNLPVKNSAIEN.TIENMYLQDLDNSTKLYGKTGAGFTANR
OXA-1-like_APM62285.1_K.p.    SSLKISPEEQIQFLRKIINHNLPVKNSAIEN.TIENMYLQDLDNSTKLYGKTGAGFTANR
OXA-1-like_APM67903.1_K.p.    SSLKISPEEQIQFLRKIINHNLPVKNSAIEN.TIENMYLQDLDNSTKLYGKTGAGFTANR
OXA-1-like_APP44482.1_K.p.    SSLKISPEEQIQFLRKIINHNLPVKNSAIEN.TIENMYLQDLDNSTKLYGKTGAGFTANR
OXA-1-like_APQ29072.1_K.p.    SSLKISPEEQIQFLRKIINHNLPVKNSAIEN.TIENMYLQDLDNSTKLYGKTGAGFTANR
OXA-1-like_APR51020.1_K.p.    SSLKISPEEQIQFLRKIINHNLPVKNSAIEN.TIENMYLQDLDNSTKLYGKTGAGFTANR
OXA-1-like_ARM26101.1_K.p.    SSLKISPEEQIQFLRKIINHNLPVKNSAIEN.TIENMYLQDLDNSTKLYGKTGAGFTANR
OXA-1-like_ARM26250.1_K.p.    SSLKISPEEQIQFLRKIINHNLPVKNSAIEN.TIENMYLQDLDNSTKLYGKTGAGFTANR
OXA-1-like_ART14424.1_K.p.    SSLKISPEEQIQFLRKIINHNLPVKNSAIEN.TIENMYLQDLDNSTKLYGKTGAGFTANR
OXA-1-like_ARX22868.1_K.p.    SSLKISPEEQIQFLRKIINHNLPVKNSAIEN.TIENMYLQDLDNSTKLYGKTGAGFTANR
OXA-1-like_ARZ96561.1_K.p.    SSLKISPEEQIQFLRKIINHNLPVKNSAIEN.TIENMYLQDLDNSTKLYGKTGAGFTANR
OXA-1-like_ASC31826.1_K.p.    SSLKISPEEQIQFLRKIINHNLPVKNSAIEN.TIENMYLQDLDNSTKLYGKTGAGFTANR
OXA-1-like_QIF55803.1_K.p.    SSLKISPEEQIQFLRKIINHNLPVKNSAIEN.TIENMYLQDLDNSTKLYGKTGAGFTANR
OXA-1-like_QIF55813.1_K.p.    SSLKISPEEQIQFLRKIINHNLPVKNSAIEN.TIENMYLQDLDNSTKLYGKTGAGFTANR
OXA-1-like_VAX91792.1_K.p.    SSLKISPEEQIQFLRKIINHNLPVKNSAIEN.TIENMYLQDLDNSTKLYGKTGAGFTANR
OXA-1-like_VAX91482.1_K.p.    SSLKISPEEQIQFLRKIINHNLPVKNSAIEN.TIENMYLQDLDNSTKLYGKTGAGFTANR
OXA-1-like_QQL36550.1_K.p.    SSLKISPEEQIQFLRKIINHNLPVKNSAIEN.TIENMYLQDLDNSTKLYGKTGAGFTANR
OXA-1-like_QPP73193.1_K.p.    SSLKISPEEQIQFLRKIINHNLPVKNSAIEN.TIENMYLQDLDNSTKLYGKTGAGFTANR
OXA-1-like_QPJ20091.1_K.p.    SSLKISPEEQIQFLRKIINHNLPVKNSAIEN.TIENMYLQDLDNSTKLYGKTGAGFTANR
OXA-1-like_QPB99042.1_K.p.    SSLKISPEEQIQFLRKIINHNLPVKNSAIEN.TIENMYLQDLDNSTKLYGKTGAGFTANR
OXA-1-like_QPA13355.1_K.p.    SSLKISPEEQIQFLRKIINHNLPVKNSAIEN.TIENMYLQDLDNSTKLYGKTGAGFTANR
OXA-1-like_QOU70610.1_K.p.    SSLKISPEEQIQFLRKIINHNLPVKNSAIEN.TIENMYLQDLDNSTKLYGKTGAGFTANR
OXA-1-like_QOU65181.1_K.p.    SSLKISPEEQIQFLRKIINHNLPVKNSAIEN.TIENMYLQDLDNSTKLYGKTGAGFTANR
OXA-1-like_QOU39182.1_K.p.    SSLKISPEEQIQFLRKIINHNLPVKNSAIEN.TIENMYLQDLDNSTKLYGKTGAGFTANR
OXA-1-like_QOT97178.1_K.p.    SSLKISPEEQIQFLRKIINHNLPVKNSAIEN.TIENMYLQDLDNSTKLYGKTGAGFTANR
OXA-1-like_QOD80635.1_K.p.    SSLKISPEEQIQFLRKIINHNLPVKNSAIEN.TIENMYLQDLDNSTKLYGKTGAGFTANR
OXA-1-like_QOD80603.1_K.p.    SSLKISPEEQIQFLRKIINHNLPVKNSAIEN.TIENMYLQDLDNSTKLYGKTGAGFTANR
OXA-1-like_QNB32204.1_K.p.    SSLKISPEEQIQFLRKIINHNLPVKNSAIEN.TIENMYLQDLDNSTKLYGKTGAGFTANR
OXA-1-like_QMS74616.1_K.p.    SSLKISPEEQIQFLRKIINHNLPVKNSAIEN.TIENMYLQDLDNSTKLYGKTGAGFTANR
OXA-1-like_QLQ67472.1_K.p.    SSLKISPEEQIQFLRKIINHNLPVKNSAIEN.TIENMYLQDLDNSTKLYGKTGAGFTANR
OXA-1-like_QLI33436.1_K.p.    SSLKISPEEQIQFLRKIINHNLPVKNSAIEN.TIENMYLQDLDNSTKLYGKTGAGFTANR
OXA-1-like_QLI28063.1_K.p.    SSLKISPEEQIQFLRKIINHNLPVKNSAIEN.TIENMYLQDLDNSTKLYGKTGAGFTANR
OXA-1-like_QLI22583.1_K.p.    SSLKISPEEQIQFLRKIINHNLPVKNSAIEN.TIENMYLQDLDNSTKLYGKTGAGFTANR
OXA-1-like_QLI17177.1_K.p.    SSLKISPEEQIQFLRKIINHNLPVKNSAIEN.TIENMYLQDLDNSTKLYGKTGAGFTANR
OXA-1-like_QLI11731.1_K.p.    SSLKISPEEQIQFLRKIINHNLPVKNSAIEN.TIENMYLQDLDNSTKLYGKTGAGFTANR
OXA-1-like_QLC16052.1_K.p.    SSLKISPEEQIQFLRKIINHNLPVKNSAIEN.TIENMYLQDLDNSTKLYGKTGAGFTANR
OXA-1-like_QLC05373.1_K.p.    SSLKISPEEQIQFLRKIINHNLPVKNSAIEN.TIENMYLQDLDNSTKLYGKTGAGFTANR
OXA-1-like_QLC00088.1_K.p.    SSLKISPEEQIQFLRKIINHNLPVKNSAIEN.TIENMYLQDLDNSTKLYGKTGAGFTANR
OXA-1-like_QLB94808.1_K.p.    SSLKISPEEQIQFLRKIINHNLPVKNSAIEN.TIENMYLQDLDNSTKLYGKTGAGFTANR
OXA-1-like_QLB89468.1_K.p.    SSLKISPEEQIQFLRKIINHNLPVKNSAIEN.TIENMYLQDLDNSTKLYGKTGAGFTANR
OXA-1-like_QLB84130.1_K.p.    SSLKISPEEQIQFLRKIINHNLPVKNSAIEN.TIENMYLQDLDNSTKLYGKTGAGFTANR
OXA-1-like_QLB78789.1_K.p.    SSLKISPEEQIQFLRKIINHNLPVKNSAIEN.TIENMYLQDLDNSTKLYGKTGAGFTANR
OXA-1-like_QLB73465.1_K.p.    SSLKISPEEQIQFLRKIINHNLPVKNSAIEN.TIENMYLQDLDNSTKLYGKTGAGFTANR
OXA-1-like_QIR58461.1_K.p.    SSLKISPEEQIQFLRKIINHNLPVKNSAIEN.TIENMYLQDLDNSTKLYGKTGAGFTANR
OXA-1-like_QHG08101.1_K.p.    SSLKISPEEQIQFLRKIINHNLPVKNSAIEN.TIENMYLQDLDNSTKLYGKTGAGFTANR
OXA-1-like_QGZ85920.1_K.p.    SSLKISPEEQIQFLRKIINHNLPVKNSAIEN.TIENMYLQDLDNSTKLYGKTGAGFTANR
OXA-1-like_QGZ80560.1_K.p.    SSLKISPEEQIQFLRKIINHNLPVKNSAIEN.TIENMYLQDLDNSTKLYGKTGAGFTANR
OXA-1-like_QGT67290.1_K.p.    SSLKISPEEQIQFLRKIINHNLPVKNSAIEN.TIENMYLQDLDNSTKLYGKTGAGFTANR
OXA-1-like_QFY31451.1_K.p.    SSLKISPEEQIQFLRKIINHNLPVKNSAIEN.TIENMYLQDLDNSTKLYGKTGAGFTANR
OXA-1-like_QFH43884.1_K.p.    SSLKISPEEQIQFLRKIINHNLPVKNSAIEN.TIENMYLQDLDNSTKLYGKTGAGFTANR
OXA-1-like_QER51630.1_K.p.    SSLKISPEEQIQFLRKIINHNLPVKNSAIEN.TIENMYLQDLDNSTKLYGKTGAGFTANR
OXA-1-like_QEG84620.1_K.p.    SSLKISPEEQIQFLRKIINHNLPVKNSAIEN.TIENMYLQDLDNSTKLYGKTGAGFTANR
```

OXA-1-like subfamily

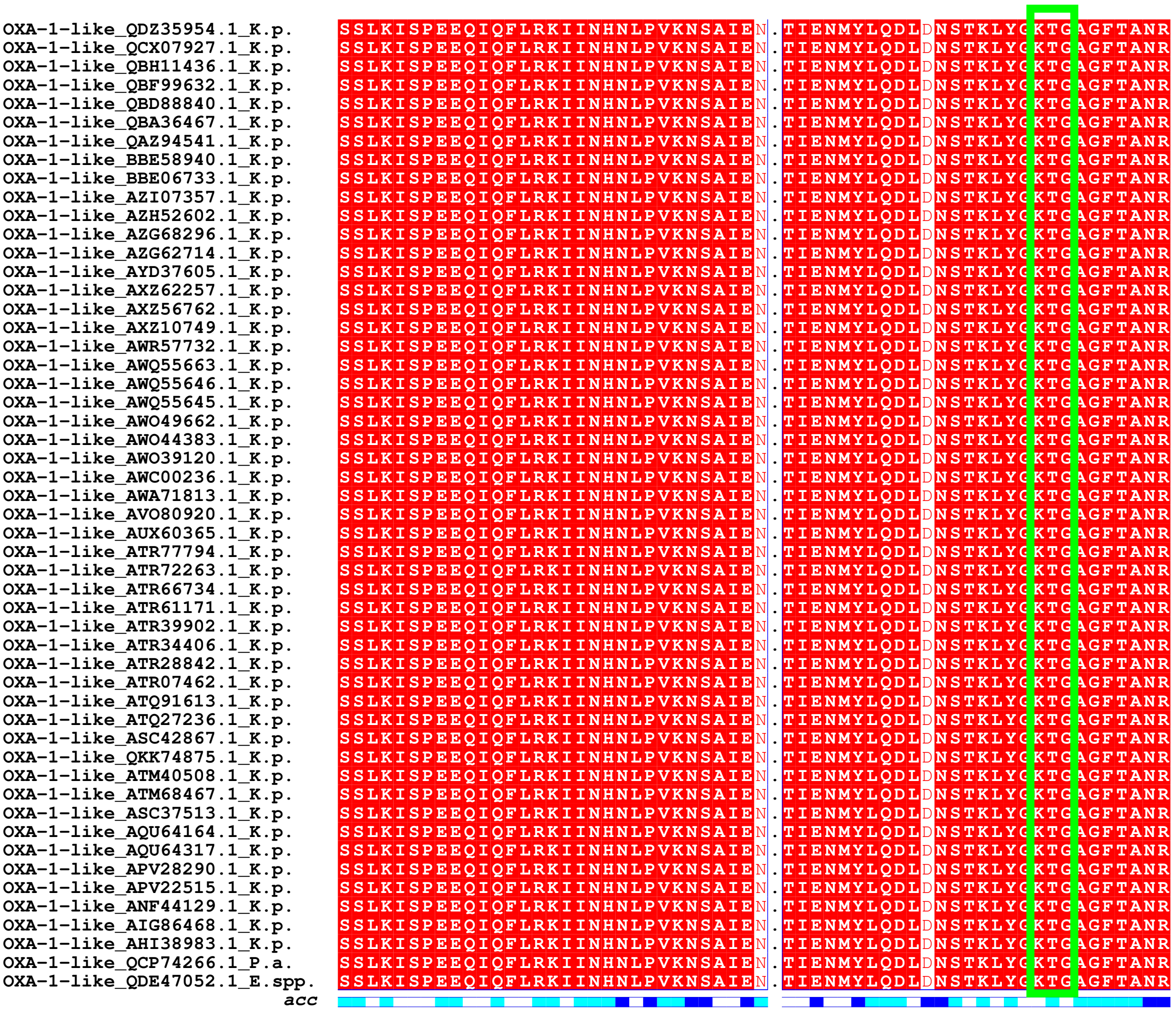

OXA-1-like subfamily

```
                                    β5                β6                    α10
OXA-1-like_BBE55408.1_K.p.    ──────────►   TT  ──────────►          QQQQQQQQQQQQQQQQ
                                230          240★         250       ★  260          270
OXA-1-like_BBE55408.1_K.p.    TLQNGWFEGFIISKSGHKYVFVSALTGNLGSNLTSSIKAKKNAITILNTLNL
1M6K_beta-lactamase_OXA-1     TLQNGWFEGFIISKSGHKYVFVSALTGNLGSNLTSSIKAKKNAITILNTLNL
OXA-1-like_AVK27274.1_P.a.    TLQNGWFEGFIISKSGHKYVFVSALTGNLGSNLTSSIKAKKNAITILNTLNL
OXA-1-like_AWE78522.1_P.a.    TLQNGWFEGFIISKSGHKYVFVSALTGNLGSNLTSSIKAKKNAITILNTLNL
OXA-1-like_QLA55054.1_P.a.    TLQNGWFEGFIISKSGHKYVFVSALTGNLGSNLTSSIKAKKNAITILNTLNL
OXA-1-like_QKG33210.1_P.a.    TLQNGWFEGFIISKSGHKYVFVSALTGNLGSNLTSSIKAKKNAITILNTLNL
OXA-1-like_QOC40829.1_K.p.    TLQNGWFEGFIISKSGHKYVFVSALTGNLGSNLTSSIKAKKNAITILNTLNL
OXA-1-like_QLF61683.1_K.p.    TLQNGWFEGFIISKSGHKYVFVSALTGNLGSNLTSSIKAKKNAITILNTLNL
OXA-1-like_ANP63432.1_P.a.    TLQNGWFEGFIISKSGHKYVFVSALTGNLGSNLTSSIKAKKNAITILNTLNL
OXA-1-like_QII98525.1_P.a.    TLQNGWFEGFIISKSGHKYVFVSALTGNLGSNLTSSIKAKKNAITILNTLNL
OXA-1-like_AXL78701.1_P.a.    TLQNGWFEGFIISKSGHKYVFVSALTGNLGSNLTSSIKAKKNAITILNTLNL
OXA-1-like_QEL33624.1_E.spp.  TLQNGWFEGFIISKSGHKYVFVSALTGNLGSNLTSSIKAKKNAITILNTLNL
OXA-1-like_QCZ41047.1_E.spp.  TLQNGWFEGFIISKSGHKYVFVSALTGNLGSNLTSSIKAKKNAITILNTLNL
OXA-1-like_QPO60228.1_E.spp.  TLQNGWFEGFIISKSGHKYVFVSALTGNLGSNLTSSIKAKKNAITILNTLNL
OXA-1-like_ATW95033.1_E.spp.  TLQNGWFEGFIISKSGHKYVFVSALTGNLGSNLTSSIKAKKNAITILNTLNL
OXA-1-like_QBB08468.1_E.spp.  TLQNGWFEGFIISKSGHKYVFVSALTGNLGSNLTSSIKAKKNAITILNTLNL
OXA-1-like_QFH57730.1_E.spp.  TLQNGWFEGFIISKSGHKYVFVSALTGNLGSNLTSSIKAKKNAITILNTLNL
OXA-1-like_QFI25362.1_E.spp.  TLQNGWFEGFIISKSGHKYVFVSALTGNLGSNLTSSIKAKKNAITILNTLNL
OXA-1-like_QHC80210.1_E.spp.  TLQNGWFEGFIISKSGHKYVFVSALTGNLGSNLTSSIKAKKNAITILNTLNL
OXA-1-like_QMT04262.1_E.spp.  TLQNGWFEGFIISKSGHKYVFVSALTGNLGSNLTSSIKAKKNAITILNTLNL
OXA-1-like_QPD73921.1_E.spp.  TLQNGWFEGFIISKSGHKYVFVSALTGNLGSNLTSSIKAKKNAITILNTLNL
OXA-1-like_QPO69359.1_E.spp.  TLQNGWFEGFIISKSGHKYVFVSALTGNLGSNLTSSIKAKKNAITILNTLNL
OXA-1-like_QPO94996.1_E.spp.  TLQNGWFEGFIISKSGHKYVFVSALTGNLGSNLTSSIKAKKNAITILNTLNL
OXA-1-like_QQA69926.1_E.spp.  TLQNGWFEGFIISKSGHKYVFVSALTGNLGSNLTSSIKAKKNAITILNTLNL
OXA-1-like_ASP02997.1_E.spp.  TLQNGWFEGFIISKSGHKYVFVSALTGNLGSNLTSSIKAKKNAITILNTLNL
OXA-1-like_AOF07832.1_K.p.    TLQNGWFEGFIISKSGHKYVFVSALTGNLGSNLTSSIKAKKNAITILNTLNL
OXA-1-like_APD44928.1_K.p.    TLQNGWFEGFIISKSGHKYVFVSALTGNLGSNLTSSIKAKKNAITILNTLNL
OXA-1-like_AWF45003.1_K.p.    TLQNGWFEGFIISKSGHKYVFVSALTGNLGSNLTSSIKAKKNAITILNTLNL
OXA-1-like_AWX26315.1_K.p.    TLQNGWFEGFIISKSGHKYVFVSALTGNLGSNLTSSIKAKKNAITILNTLNL
OXA-1-like_BBE66685.1_K.p.    TLQNGWFEGFIISKSGHKYVFVSALTGNLGSNLTSSIKAKKNAITILNTLNL
OXA-1-like_ARA49869.1_K.p.    TLQNGWFEGFIISKSGHKYVFVSALTGNLGSNLTSSIKAKKNAITILNTLNL
OXA-1-like_ART03029.1_K.p.    TLQNGWFEGFIISKSGHKYVFVSALTGNLGSNLTSSIKAKKNAITILNTLNL
OXA-1-like_ART14043.1_K.p.    TLQNGWFEGFIISKSGHKYVFVSALTGNLGSNLTSSIKAKKNAITILNTLNL
OXA-1-like_ATO08041.1_K.p.    TLQNGWFEGFIISKSGHKYVFVSALTGNLGSNLTSSIKAKKNAITILNTLNL
OXA-1-like_ATQ97012.1_K.p.    TLQNGWFEGFIISKSGHKYVFVSALTGNLGSNLTSSIKAKKNAITILNTLNL
OXA-1-like_AUD20317.1_K.p.    TLQNGWFEGFIISKSGHKYVFVSALTGNLGSNLTSSIKAKKNAITILNTLNL
OXA-1-like_AUD31186.1_K.p.    TLQNGWFEGFIISKSGHKYVFVSALTGNLGSNLTSSIKAKKNAITILNTLNL
OXA-1-like_AUD36737.1_K.p.    TLQNGWFEGFIISKSGHKYVFVSALTGNLGSNLTSSIKAKKNAITILNTLNL
OXA-1-like_AVF14577.1_K.p.    TLQNGWFEGFIISKSGHKYVFVSALTGNLGSNLTSSIKAKKNAITILNTLNL
OXA-1-like_AWC05483.1_K.p.    TLQNGWFEGFIISKSGHKYVFVSALTGNLGSNLTSSIKAKKNAITILNTLNL
OXA-1-like_QAZ99397.1_K.p.    TLQNGWFEGFIISKSGHKYVFVSALTGNLGSNLTSSIKAKKNAITILNTLNL
OXA-1-like_QIF88052.1_K.p.    TLQNGWFEGFIISKSGHKYVFVSALTGNLGSNLTSSIKAKKNAITILNTLNL
OXA-1-like_QLF61156.1_K.p.    TLQNGWFEGFIISKSGHKYVFVSALTGNLGSNLTSSIKAKKNAITILNTLNL
OXA-1-like_QLF66737.1_K.p.    TLQNGWFEGFIISKSGHKYVFVSALTGNLGSNLTSSIKAKKNAITILNTLNL
OXA-1-like_QLG18431.1_K.p.    TLQNGWFEGFIISKSGHKYVFVSALTGNLGSNLTSSIKAKKNAITILNTLNL
OXA-1-like_AIT04829.1_K.p.    TLQNGWFEGFIISKSGHKYVFVSALTGNLGSNLTSSIKAKKNAITILNTLNL
OXA-1-like_AKE78741.1_K.p.    TLQNGWFEGFIISKSGHKYVFVSALTGNLGSNLTSSIKAKKNAITILNTLNL
OXA-1-like_ANK19483.1_K.p.    TLQNGWFEGFIISKSGHKYVFVSALTGNLGSNLTSSIKAKKNAITILNTLNL
OXA-1-like_AOF02731.1_K.p.    TLQNGWFEGFIISKSGHKYVFVSALTGNLGSNLTSSIKAKKNAITILNTLNL
OXA-1-like_APB53840.1_K.p.    TLQNGWFEGFIISKSGHKYVFVSALTGNLGSNLTSSIKAKKNAITILNTLNL
OXA-1-like_APM47234.1_K.p.    TLQNGWFEGFIISKSGHKYVFVSALTGNLGSNLTSSIKAKKNAITILNTLNL
OXA-1-like_APM56693.1_K.p.    TLQNGWFEGFIISKSGHKYVFVSALTGNLGSNLTSSIKAKKNAITILNTLNL
OXA-1-like_APM62285.1_K.p.    TLQNGWFEGFIISKSGHKYVFVSALTGNLGSNLTSSIKAKKNAITILNTLNL
OXA-1-like_APM67903.1_K.p.    TLQNGWFEGFIISKSGHKYVFVSALTGNLGSNLTSSIKAKKNAITILNTLNL
OXA-1-like_APP44482.1_K.p.    TLQNGWFEGFIISKSGHKYVFVSALTGNLGSNLTSSIKAKKNAITILNTLNL
OXA-1-like_APQ29072.1_K.p.    TLQNGWFEGFIISKSGHKYVFVSALTGNLGSNLTSSIKAKKNAITILNTLNL
OXA-1-like_APR51020.1_K.p.    TLQNGWFEGFIISKSGHKYVFVSALTGNLGSNLTSSIKAKKNAITILNTLNL
OXA-1-like_ARM26101.1_K.p.    TLQNGWFEGFIISKSGHKYVFVSALTGNLGSNLTSSIKAKKNAITILNTLNL
OXA-1-like_ARM26250.1_K.p.    TLQNGWFEGFIISKSGHKYVFVSALTGNLGSNLTSSIKAKKNAITILNTLNL
OXA-1-like_ART14424.1_K.p.    TLQNGWFEGFIISKSGHKYVFVSALTGNLGSNLTSSIKAKKNAITILNTLNL
OXA-1-like_ARX22868.1_K.p.    TLQNGWFEGFIISKSGHKYVFVSALTGNLGSNLTSSIKAKKNAITILNTLNL
OXA-1-like_ARZ96561.1_K.p.    TLQNGWFEGFIISKSGHKYVFVSALTGNLGSNLTSSIKAKKNAITILNTLNL
OXA-1-like_ASC31826.1_K.p.    TLQNGWFEGFIISKSGHKYVFVSALTGNLGSNLTSSIKAKKNAITILNTLNL
OXA-1-like_QIF55803.1_K.p.    TLQNGWFEGFIISKSGHKYVFVSALTGNLGSNLTSSIKAKKNAITILNTLNL
OXA-1-like_QIF55813.1_K.p.    TLQNGWFEGFIISKSGHKYVFVSALTGNLGSNLTSSIKAKKNAITILNTLNL
OXA-1-like_VAX91792.1_K.p.    TLQNGWFEGFIISKSGHKYVFVSALTGNLGSNLTSSIKAKKNAITILNTLNL
OXA-1-like_VAX91482.1_K.p.    TLQNGWFEGFIISKSGHKYVFVSALTGNLGSNLTSSIKAKKNAITILNTLNL
OXA-1-like_QQL36550.1_K.p.    TLQNGWFEGFIISKSGHKYVFVSALTGNLGSNLTSSIKAKKNAITILNTLNL
OXA-1-like_QPP73193.1_K.p.    TLQNGWFEGFIISKSGHKYVFVSALTGNLGSNLTSSIKAKKNAITILNTLNL
OXA-1-like_QPJ20091.1_K.p.    TLQNGWFEGFIISKSGHKYVFVSALTGNLGSNLTSSIKAKKNAITILNTLNL
OXA-1-like_QPB99042.1_K.p.    TLQNGWFEGFIISKSGHKYVFVSALTGNLGSNLTSSIKAKKNAITILNTLNL
OXA-1-like_QPA13355.1_K.p.    TLQNGWFEGFIISKSGHKYVFVSALTGNLGSNLTSSIKAKKNAITILNTLNL
OXA-1-like_QOU70610.1_K.p.    TLQNGWFEGFIISKSGHKYVFVSALTGNLGSNLTSSIKAKKNAITILNTLNL
OXA-1-like_QOU65181.1_K.p.    TLQNGWFEGFIISKSGHKYVFVSALTGNLGSNLTSSIKAKKNAITILNTLNL
OXA-1-like_QOU39182.1_K.p.    TLQNGWFEGFIISKSGHKYVFVSALTGNLGSNLTSSIKAKKNAITILNTLNL
OXA-1-like_QOT97178.1_K.p.    TLQNGWFEGFIISKSGHKYVFVSALTGNLGSNLTSSIKAKKNAITILNTLNL
OXA-1-like_QOD80635.1_K.p.    TLQNGWFEGFIISKSGHKYVFVSALTGNLGSNLTSSIKAKKNAITILNTLNL
OXA-1-like_QOD80603.1_K.p.    TLQNGWFEGFIISKSGHKYVFVSALTGNLGSNLTSSIKAKKNAITILNTLNL
OXA-1-like_QNB32204.1_K.p.    TLQNGWFEGFIISKSGHKYVFVSALTGNLGSNLTSSIKAKKNAITILNTLNL
OXA-1-like_QMS74616.1_K.p.    TLQNGWFEGFIISKSGHKYVFVSALTGNLGSNLTSSIKAKKNAITILNTLNL
OXA-1-like_QLQ67472.1_K.p.    TLQNGWFEGFIISKSGHKYVFVSALTGNLGSNLTSSIKAKKNAITILNTLNL
OXA-1-like_QLI33436.1_K.p.    TLQNGWFEGFIISKSGHKYVFVSALTGNLGSNLTSSIKAKKNAITILNTLNL
OXA-1-like_QLI28063.1_K.p.    TLQNGWFEGFIISKSGHKYVFVSALTGNLGSNLTSSIKAKKNAITILNTLNL
OXA-1-like_QLI22583.1_K.p.    TLQNGWFEGFIISKSGHKYVFVSALTGNLGSNLTSSIKAKKNAITILNTLNL
OXA-1-like_QLI17177.1_K.p.    TLQNGWFEGFIISKSGHKYVFVSALTGNLGSNLTSSIKAKKNAITILNTLNL
OXA-1-like_QLI11731.1_K.p.    TLQNGWFEGFIISKSGHKYVFVSALTGNLGSNLTSSIKAKKNAITILNTLNL
OXA-1-like_QLC16052.1_K.p.    TLQNGWFEGFIISKSGHKYVFVSALTGNLGSNLTSSIKAKKNAITILNTLNL
OXA-1-like_QLC05373.1_K.p.    TLQNGWFEGFIISKSGHKYVFVSALTGNLGSNLTSSIKAKKNAITILNTLNL
OXA-1-like_QLC00088.1_K.p.    TLQNGWFEGFIISKSGHKYVFVSALTGNLGSNLTSSIKAKKNAITILNTLNL
OXA-1-like_QLB94808.1_K.p.    TLQNGWFEGFIISKSGHKYVFVSALTGNLGSNLTSSIKAKKNAITILNTLNL
OXA-1-like_QLB89468.1_K.p.    TLQNGWFEGFIISKSGHKYVFVSALTGNLGSNLTSSIKAKKNAITILNTLNL
OXA-1-like_QLB84130.1_K.p.    TLQNGWFEGFIISKSGHKYVFVSALTGNLGSNLTSSIKAKKNAITILNTLNL
OXA-1-like_QLB78789.1_K.p.    TLQNGWFEGFIISKSGHKYVFVSALTGNLGSNLTSSIKAKKNAITILNTLNL
OXA-1-like_QLB73465.1_K.p.    TLQNGWFEGFIISKSGHKYVFVSALTGNLGSNLTSSIKAKKNAITILNTLNL
OXA-1-like_QIR58461.1_K.p.    TLQNGWFEGFIISKSGHKYVFVSALTGNLGSNLTSSIKAKKNAITILNTLNL
OXA-1-like_QHG08101.1_K.p.    TLQNGWFEGFIISKSGHKYVFVSALTGNLGSNLTSSIKAKKNAITILNTLNL
OXA-1-like_QGZ85920.1_K.p.    TLQNGWFEGFIISKSGHKYVFVSALTGNLGSNLTSSIKAKKNAITILNTLNL
OXA-1-like_QGZ80560.1_K.p.    TLQNGWFEGFIISKSGHKYVFVSALTGNLGSNLTSSIKAKKNAITILNTLNL
OXA-1-like_QGT67290.1_K.p.    TLQNGWFEGFIISKSGHKYVFVSALTGNLGSNLTSSIKAKKNAITILNTLNL
OXA-1-like_QFY31451.1_K.p.    TLQNGWFEGFIISKSGHKYVFVSALTGNLGSNLTSSIKAKKNAITILNTLNL
OXA-1-like_QFH43884.1_K.p.    TLQNGWFEGFIISKSGHKYVFVSALTGNLGSNLTSSIKAKKNAITILNTLNL
OXA-1-like_QER51630.1_K.p.    TLQNGWFEGFIISKSGHKYVFVSALTGNLGSNLTSSIKAKKNAITILNTLNL
OXA-1-like_QEG84620.1_K.p.    TLQNGWFEGFIISKSGHKYVFVSALTGNLGSNLTSSIKAKKNAITILNTLNL
```

OXA-1-like subfamily

```
OXA-1-like_QDZ35954.1_K.p.    TLQNGWFEGFIISKSGHKYVFVSALTGNLGSNLTSSIKAKKNAITILNTLNL
OXA-1-like_QCX07927.1_K.p.    TLQNGWFEGFIISKSGHKYVFVSALTGNLGSNLTSSIKAKKNAITILNTLNL
OXA-1-like_QBH11436.1_K.p.    TLQNGWFEGFIISKSGHKYVFVSALTGNLGSNLTSSIKAKKNAITILNTLNL
OXA-1-like_QBF99632.1_K.p.    TLQNGWFEGFIISKSGHKYVFVSALTGNLGSNLTSSIKAKKNAITILNTLNL
OXA-1-like_QBD88840.1_K.p.    TLQNGWFEGFIISKSGHKYVFVSALTGNLGSNLTSSIKAKKNAITILNTLNL
OXA-1-like_QBA36467.1_K.p.    TLQNGWFEGFIISKSGHKYVFVSALTGNLGSNLTSSIKAKKNAITILNTLNL
OXA-1-like_QAZ94541.1_K.p.    TLQNGWFEGFIISKSGHKYVFVSALTGNLGSNLTSSIKAKKNAITILNTLNL
OXA-1-like_BBE58940.1_K.p.    TLQNGWFEGFIISKSGHKYVFVSALTGNLGSNLTSSIKAKKNAITILNTLNL
OXA-1-like_BBE06733.1_K.p.    TLQNGWFEGFIISKSGHKYVFVSALTGNLGSNLTSSIKAKKNAITILNTLNL
OXA-1-like_AZI07357.1_K.p.    TLQNGWFEGFIISKSGHKYVFVSALTGNLGSNLTSSIKAKKNAITILNTLNL
OXA-1-like_AZH52602.1_K.p.    TLQNGWFEGFIISKSGHKYVFVSALTGNLGSNLTSSIKAKKNAITILNTLNL
OXA-1-like_AZG68296.1_K.p.    TLQNGWFEGFIISKSGHKYVFVSALTGNLGSNLTSSIKAKKNAITILNTLNL
OXA-1-like_AZG62714.1_K.p.    TLQNGWFEGFIISKSGHKYVFVSALTGNLGSNLTSSIKAKKNAITILNTLNL
OXA-1-like_AYD37605.1_K.p.    TLQNGWFEGFIISKSGHKYVFVSALTGNLGSNLTSSIKAKKNAITILNTLNL
OXA-1-like_AXZ62257.1_K.p.    TLQNGWFEGFIISKSGHKYVFVSALTGNLGSNLTSSIKAKKNAITILNTLNL
OXA-1-like_AXZ56762.1_K.p.    TLQNGWFEGFIISKSGHKYVFVSALTGNLGSNLTSSIKAKKNAITILNTLNL
OXA-1-like_AXZ10749.1_K.p.    TLQNGWFEGFIISKSGHKYVFVSALTGNLGSNLTSSIKAKKNAITILNTLNL
OXA-1-like_AWR57732.1_K.p.    TLQNGWFEGFIISKSGHKYVFVSALTGNLGSNLTSSIKAKKNAITILNTLNL
OXA-1-like_AWQ55663.1_K.p.    TLQNGWFEGFIISKSGHKYVFVSALTGNLGSNLTSSIKAKKNAITILNTLNL
OXA-1-like_AWQ55646.1_K.p.    TLQNGWFEGFIISKSGHKYVFVSALTGNLGSNLTSSIKAKKNAITILNTLNL
OXA-1-like_AWQ55645.1_K.p.    TLQNGWFEGFIISKSGHKYVFVSALTGNLGSNLTSSIKAKKNAITILNTLNL
OXA-1-like_AWO49662.1_K.p.    TLQNGWFEGFIISKSGHKYVFVSALTGNLGSNLTSSIKAKKNAITILNTLNL
OXA-1-like_AWO44383.1_K.p.    TLQNGWFEGFIISKSGHKYVFVSALTGNLGSNLTSSIKAKKNAITILNTLNL
OXA-1-like_AWO39120.1_K.p.    TLQNGWFEGFIISKSGHKYVFVSALTGNLGSNLTSSIKAKKNAITILNTLNL
OXA-1-like_AWC00236.1_K.p.    TLQNGWFEGFIISKSGHKYVFVSALTGNLGSNLTSSIKAKKNAITILNTLNL
OXA-1-like_AWA71813.1_K.p.    TLQNGWFEGFIISKSGHKYVFVSALTGNLGSNLTSSIKAKKNAITILNTLNL
OXA-1-like_AVO80920.1_K.p.    TLQNGWFEGFIISKSGHKYVFVSALTGNLGSNLTSSIKAKKNAITILNTLNL
OXA-1-like_AUX60365.1_K.p.    TLQNGWFEGFIISKSGHKYVFVSALTGNLGSNLTSSIKAKKNAITILNTLNL
OXA-1-like_ATR77794.1_K.p.    TLQNGWFEGFIISKSGHKYVFVSALTGNLGSNLTSSIKAKKNAITILNTLNL
OXA-1-like_ATR72263.1_K.p.    TLQNGWFEGFIISKSGHKYVFVSALTGNLGSNLTSSIKAKKNAITILNTLNL
OXA-1-like_ATR66734.1_K.p.    TLQNGWFEGFIISKSGHKYVFVSALTGNLGSNLTSSIKAKKNAITILNTLNL
OXA-1-like_ATR61171.1_K.p.    TLQNGWFEGFIISKSGHKYVFVSALTGNLGSNLTSSIKAKKNAITILNTLNL
OXA-1-like_ATR39902.1_K.p.    TLQNGWFEGFIISKSGHKYVFVSALTGNLGSNLTSSIKAKKNAITILNTLNL
OXA-1-like_ATR34406.1_K.p.    TLQNGWFEGFIISKSGHKYVFVSALTGNLGSNLTSSIKAKKNAITILNTLNL
OXA-1-like_ATR28842.1_K.p.    TLQNGWFEGFIISKSGHKYVFVSALTGNLGSNLTSSIKAKKNAITILNTLNL
OXA-1-like_ATR07462.1_K.p.    TLQNGWFEGFIISKSGHKYVFVSALTGNLGSNLTSSIKAKKNAITILNTLNL
OXA-1-like_ATQ91613.1_K.p.    TLQNGWFEGFIISKSGHKYVFVSALTGNLGSNLTSSIKAKKNAITILNTLNL
OXA-1-like_ATQ27236.1_K.p.    TLQNGWFEGFIISKSGHKYVFVSALTGNLGSNLTSSIKAKKNAITILNTLNL
OXA-1-like_ASC42867.1_K.p.    TLQNGWFEGFIISKSGHKYVFVSALTGNLGSNLTSSIKAKKNAITILNTLNL
OXA-1-like_QKK74875.1_K.p.    TLQNGWFEGFIISKSGHKYVFVSALTGNLGSNLTSSIKAKKNAITILNTLNL
OXA-1-like_ATM40508.1_K.p.    TLQNGWFEGFIISKSGHKYVFVSALTGNLGSNLTSSIKAKKNAITILNTLNL
OXA-1-like_ATM68467.1_K.p.    TLQNGWFEGFIISKSGHKYVFVSALTGNLGSNLTSSIKAKKNAITILNTLNL
OXA-1-like_ASC37513.1_K.p.    TLQNGWFEGFIISKSGHKYVFVSALTGNLGSNLTSSIKAKKNAITILNTLNL
OXA-1-like_AQU64164.1_K.p.    TLQNGWFEGFIISKSGHKYVFVSALTGNLGSNLTSSIKAKKNAITILNTLNL
OXA-1-like_AQU64317.1_K.p.    TLQNGWFEGFIISKSGHKYVFVSALTGNLGSNLTSSIKAKKNAITILNTLNL
OXA-1-like_APV28290.1_K.p.    TLQNGWFEGFIISKSGHKYVFVSALTGNLGSNLTSSIKAKKNAITILNTLNL
OXA-1-like_APV22515.1_K.p.    TLQNGWFEGFIISKSGHKYVFVSALTGNLGSNLTSSIKAKKNAITILNTLNL
OXA-1-like_ANF44129.1_K.p.    TLQNGWFEGFIISKSGHKYVFVSALTGNLGSNLTSSIKAKKNAITILNTLNL
OXA-1-like_AIG86468.1_K.p.    TLQNGWFEGFIISKSGHKYVFVSALTGNLGSNLTSSIKAKKNAITILNTLNL
OXA-1-like_AHI38983.1_K.p.    TLQNGWFEGFIISKSGHKYVFVSALTGNLGSNLTSSIKAKKNAITILNTLNL
OXA-1-like_QCP74266.1_P.a.    TLQNGWFEGFIISKSGHKYVFVSALTGSLGSNLTSSIKAKKNAITILNTLNL
OXA-1-like_QDE47052.1_E.spp.  TLQNGWFEGFIISKSGHKYVFVSALTGNLGSNLTSSIKAKKNAITILNTLNL
                        acc
```

OXA-2-like subfamily

```
1K38_1_Beta-lactamase

1K38_1_Beta-lactamase            ............................................................
OXA-2-like_VAV79013.1_E.spp.     ............................................................
OXA-2-like_AXX42030.1_A.b.       ............................................................
OXA-2-like_APV34059.1_K.p.       ............................................................
OXA-2-like_ARG86543.1_P.a.       ............................................................
OXA-2-like_AVK22308.1_P.a.       ............................................................
OXA-2-like_SZD41030.1_P.a.       ............................................................
OXA-2-like_ASA15328.1_P.a.       ............................................................
OXA-2-like_ASA17138.1_P.a.       ............................................................
OXA-2-like_ASD12027.1_P.a.       ............................................................
OXA-2-like_AYZ57805.1_P.a.       ............................................................
OXA-2-like_QPV55945.1_P.a.       ............................................................
OXA-2-like_AXS74056.1_P.a.       MSRAPVRDRRISVRKSRPACCCPHCCSTRRRSTRVCRAFDPPVCGGVLLRQRCVLGGLVR
                             acc
```

```
1K38_1_Beta-lactamase

1K38_1_Beta-lactamase            ............................................................
OXA-2-like_VAV79013.1_E.spp.     ............................................................
OXA-2-like_AXX42030.1_A.b.       ............................................................
OXA-2-like_APV34059.1_K.p.       ............................................................
OXA-2-like_ARG86543.1_P.a.       ............................................................
OXA-2-like_AVK22308.1_P.a.       ............................................................
OXA-2-like_SZD41030.1_P.a.       ............................................................
OXA-2-like_ASA15328.1_P.a.       ............................................................
OXA-2-like_ASA17138.1_P.a.       ............................................................
OXA-2-like_ASD12027.1_P.a.       ............................................................
OXA-2-like_AYZ57805.1_P.a.       ............................................................
OXA-2-like_QPV55945.1_P.a.       ............................................................
OXA-2-like_AXS74056.1_P.a.       CAKCAASGRRCSSGKSRRALGSWSRMHRIRLRHSTYQQRKHLGAPGGWIHGGCSSTLLPE
                             acc
```

```
1K38_1_Beta-lactamase

1K38_1_Beta-lactamase            ............................................................
OXA-2-like_VAV79013.1_E.spp.     ...................................................MAIRIFAIL
OXA-2-like_AXX42030.1_A.b.       ...................................................MAIRIFAIL
OXA-2-like_APV34059.1_K.p.       ...................................................MAIRIFAIL
OXA-2-like_ARG86543.1_P.a.       ...................................................MAIRIFAIL
OXA-2-like_AVK22308.1_P.a.       ...................................................MAIRIFAIL
OXA-2-like_SZD41030.1_P.a.       ...................................................MAIRIFAIL
OXA-2-like_ASA15328.1_P.a.       ...................................................MAIRIFAIL
OXA-2-like_ASA17138.1_P.a.       ...................................................MAIRIFAIL
OXA-2-like_ASD12027.1_P.a.       ...................................................MAIRIFAIL
OXA-2-like_AYZ57805.1_P.a.       ...................................................MAIRIFAIL
OXA-2-like_QPV55945.1_P.a.       ...................................................MAIRIFAIL
OXA-2-like_AXS74056.1_P.a.       TVVRGAALVPNNSFKPNLLRYTKAMAERACHGFGSTTQVGLTQQLGIKEKLMAIRIFAIL
                             acc
```

```
                                               β1   η1   α1            β2            β3  α2
1K38_1_Beta-lactamase                        1        10        20        30      *  40
1K38_1_Beta-lactamase            ............QEGTLERSDWRKFFSEFQAKGTIVVADERQADRAMLVFDPVRSKKRYS
OXA-2-like_VAV79013.1_E.spp.     FSIFSLATFAHAQEGTLERSDWRKFFSEFQAKGTIVVADERQADRAMLVFDPVRSKKRYS
OXA-2-like_AXX42030.1_A.b.       FSIFSLATFAHAQEGTLERSDWRKFFSEFQAKGTIVVADERQADRAMLVFDPVRSKKRYS
OXA-2-like_APV34059.1_K.p.       FSIFSLATFAHAQEGTLERSDWRKFFSEFQAKGTIVVADERQADRAMLVFDPVRSKKRYS
OXA-2-like_ARG86543.1_P.a.       FSIFSLATFAHAQEGTLERSDWRKFFSEFQAKGTIVVADERQADRAMLVFDPVRSKKRYS
OXA-2-like_AVK22308.1_P.a.       FSIFSLATFAHAQEGTLERSDWRKFFSEFQAKGTIVVADERQADRAMLVFDPVRSKKRYS
OXA-2-like_SZD41030.1_P.a.       FSIFSLATFAHAQEGTLERSDWRKFFSEFQAKGTIVVADERQADRAMLVFDPVRSKKRYS
OXA-2-like_ASA15328.1_P.a.       FSIFSLATFAHAQEGTLERSDWRKFFSEFQAKGTIVVADERQADRAMLVFDPVRSKKRYS
OXA-2-like_ASA17138.1_P.a.       FSIFSLATFAHAQEGTLERSDWRKFFSEFQAKGTIVVADERQADRAMLVFDPVRSKKRYS
OXA-2-like_ASD12027.1_P.a.       FSIFSLATFAHAQEGTLERSDWRKFFSEFQAKGTIVVADERQADRAMLVFDPVRSKKRYS
OXA-2-like_AYZ57805.1_P.a.       FSIFSLATFAHAQEGTLERSDWRKFFSEFQAKGTIVVADERQADRAMLVFDPVRSKKRYS
OXA-2-like_QPV55945.1_P.a.       FSIFSLATFAHAQEGTLERSDWRKFFSEFQAKGTIVVADERQADRAMLVFDPVRSKKRYS
OXA-2-like_AXS74056.1_P.a.       FSIFSLATFAHAQEGTLERSDWRKFFSEFQAKGTIVVADERQADRAMLVFDPVRSKKRYS
                             acc
```

```
                                  η2       α3                                     α4       α5
1K38_1_Beta-lactamase                                   TT
                                 50        60        70        80        90     * 100
1K38_1_Beta-lactamase            PASTFKIPHTLFALDAGAVRDEFQIFRWDGVNRGFAGHNQDQDLRSAMRNSTVWVYELFA
OXA-2-like_VAV79013.1_E.spp.     PASTFKIPHTLFALDAGAVRDEFQIFRWDGVNRGFAGHNQDQDLRSAMRNSTVWVYELFA
OXA-2-like_AXX42030.1_A.b.       PASTFKIPHTLFALDAGAVRDEFQIFRWDGVNRGFAGHNQDQDLRSAMRNSTVWVYELFA
OXA-2-like_APV34059.1_K.p.       PASTFKIPHTLFALDAGAVRDEFQIFRWDGVNRGFAGHNQDQDLRSAMRNSTVWVYELFA
OXA-2-like_ARG86543.1_P.a.       PASTFKIPHTLFALDAGAVRDEFQIFRWDGVNRGFAGHNQDQDLRSAMRNSTVWVYELFA
OXA-2-like_AVK22308.1_P.a.       PASTFKIPHTLFALDAGAVRDEFQIFRWDGVNRGFAGHNQDQDLRSAMRNSTVWVYELFA
OXA-2-like_SZD41030.1_P.a.       PASTFKIPHTLFALDAGAVRDEFQIFRWDGVNRGFAGHNQDQDLRSAMRNSTVWVYELFA
OXA-2-like_ASA15328.1_P.a.       PASTFKIPHTLFALDAGAVRDEFQIFRWDGVNRGFAGHNQDQDLRSAMRNSTVWVYELFA
OXA-2-like_ASA17138.1_P.a.       PASTFKIPHTLFALDAGAVRDEFQIFRWDGVNRGFAGHNQDQDLRSAMRNSTVWVYELFA
OXA-2-like_ASD12027.1_P.a.       PASTFKIPHTLFALDAGAVRDEFQIFRWDGVNRGFAGHNQDQDLRSAMRNSTVWVYELFA
OXA-2-like_AYZ57805.1_P.a.       PASTFKIPHTLFALDAGAVRDEFQIFRWDGVNRGFAGHNQDQDLRSAMRNSTVWVYELFA
OXA-2-like_QPV55945.1_P.a.       PASTFKIPHTLFALDAGAVRDEFQIFRWDGVNRGFAGHNQDQDLRSAMRNSTVWVYELFA
OXA-2-like_AXS74056.1_P.a.       PASTFKIPHTLFALDAGAVRDEFQIFRWDGVNRGFAGHNQDQDLRSAMRNSTVWVYELFA
                             acc
```

OXA-2-like subfamily

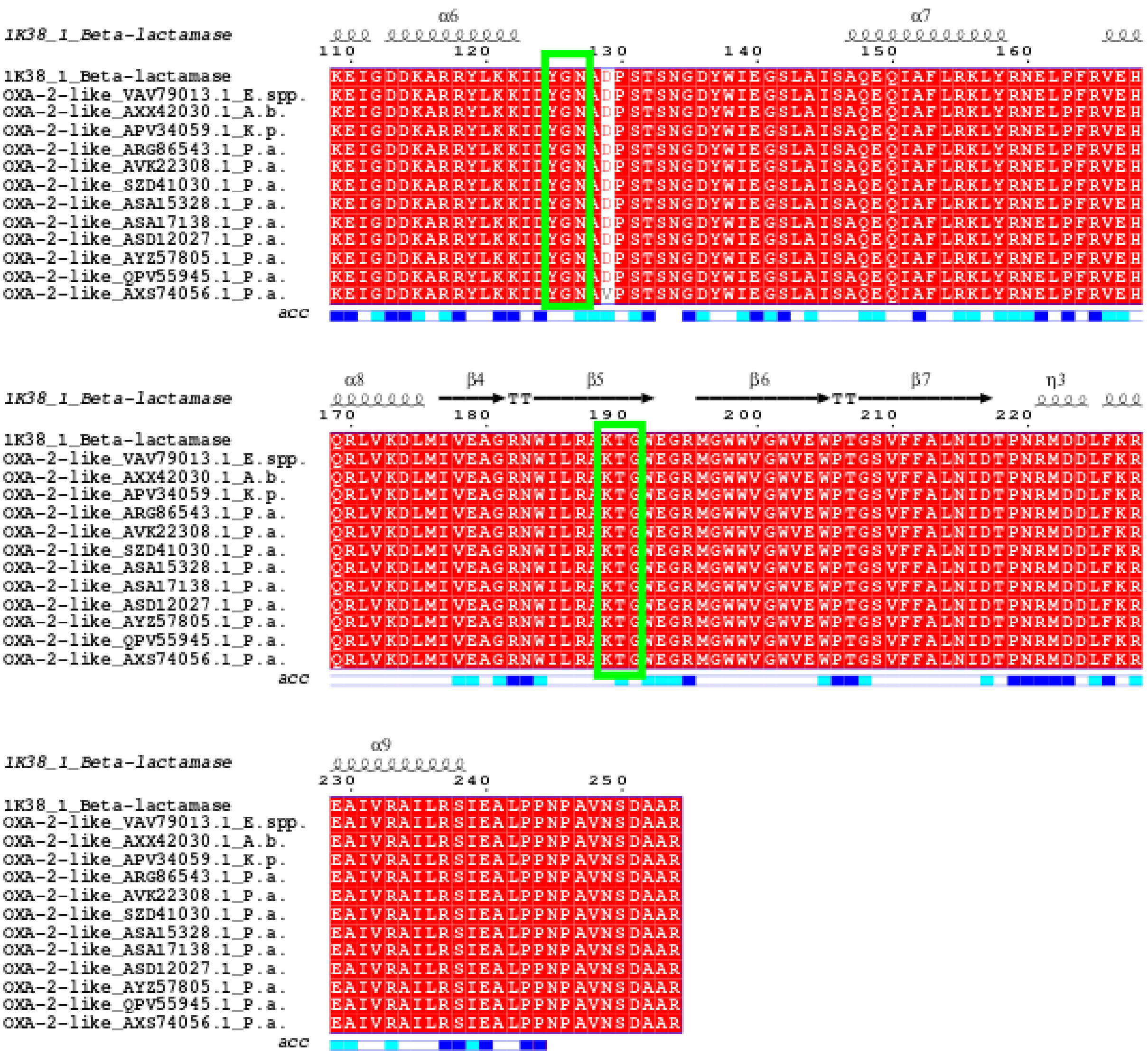

OXA-5-like subfamily

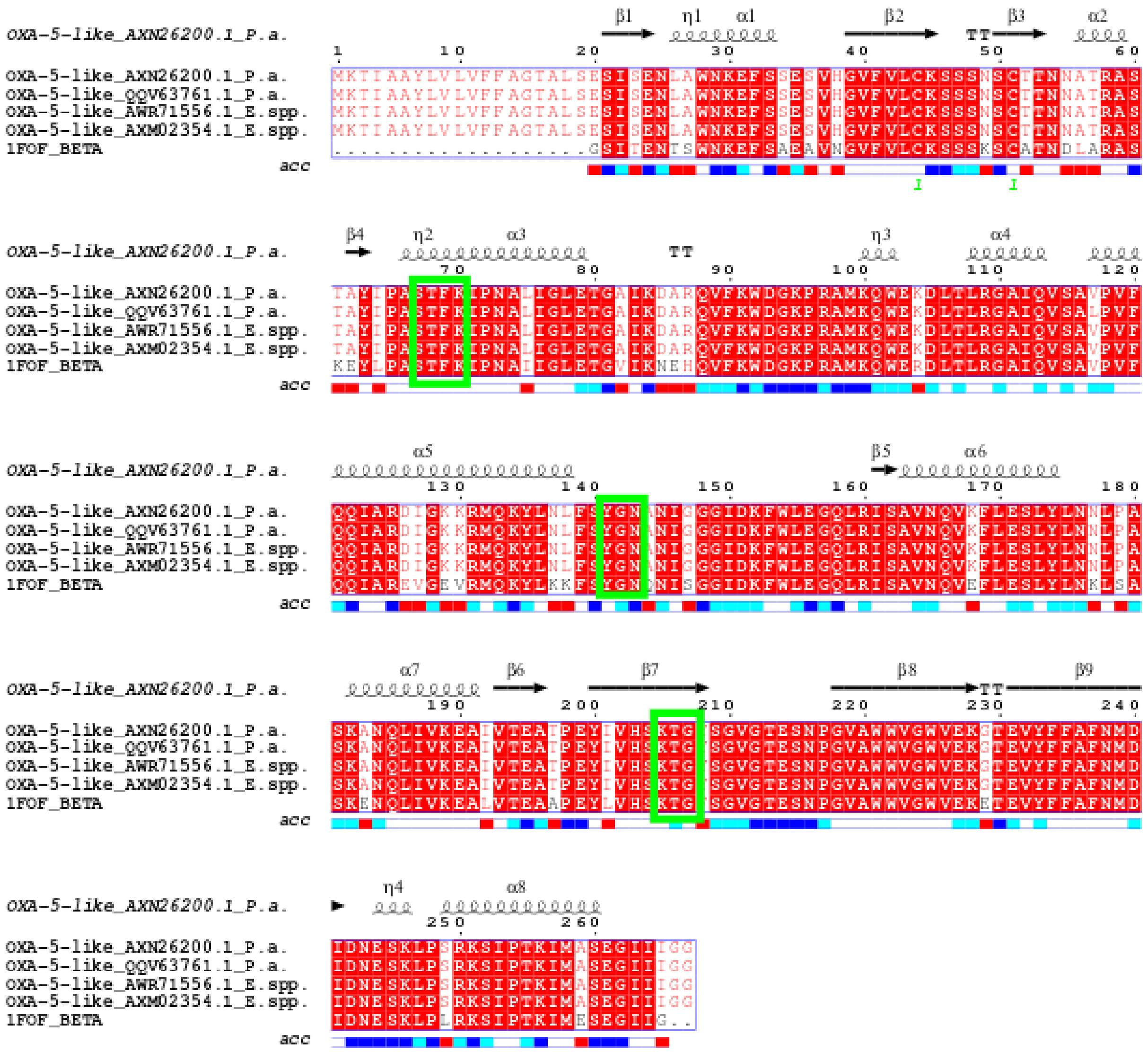

OXA-9-like subfamily

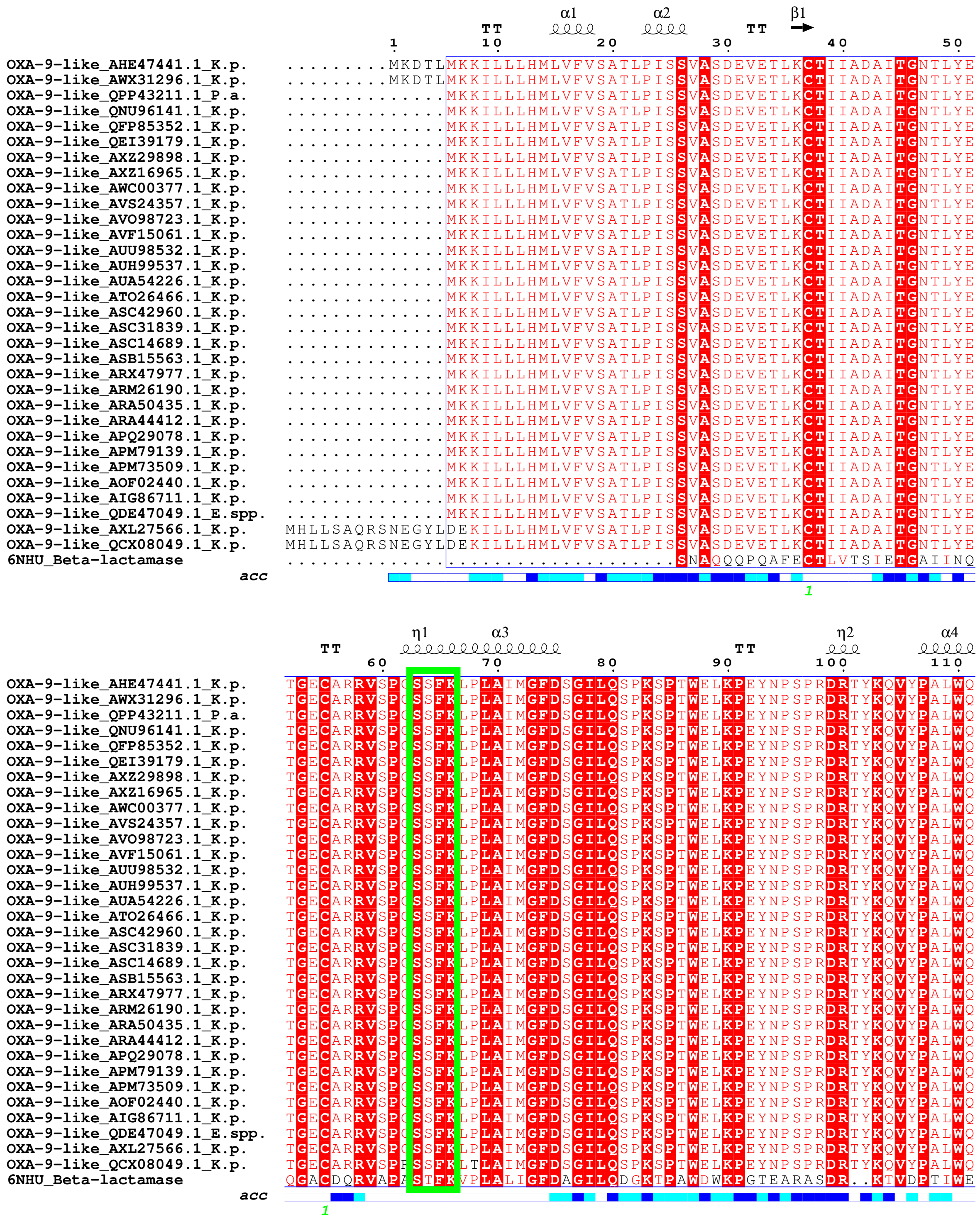

OXA-9-like subfamily

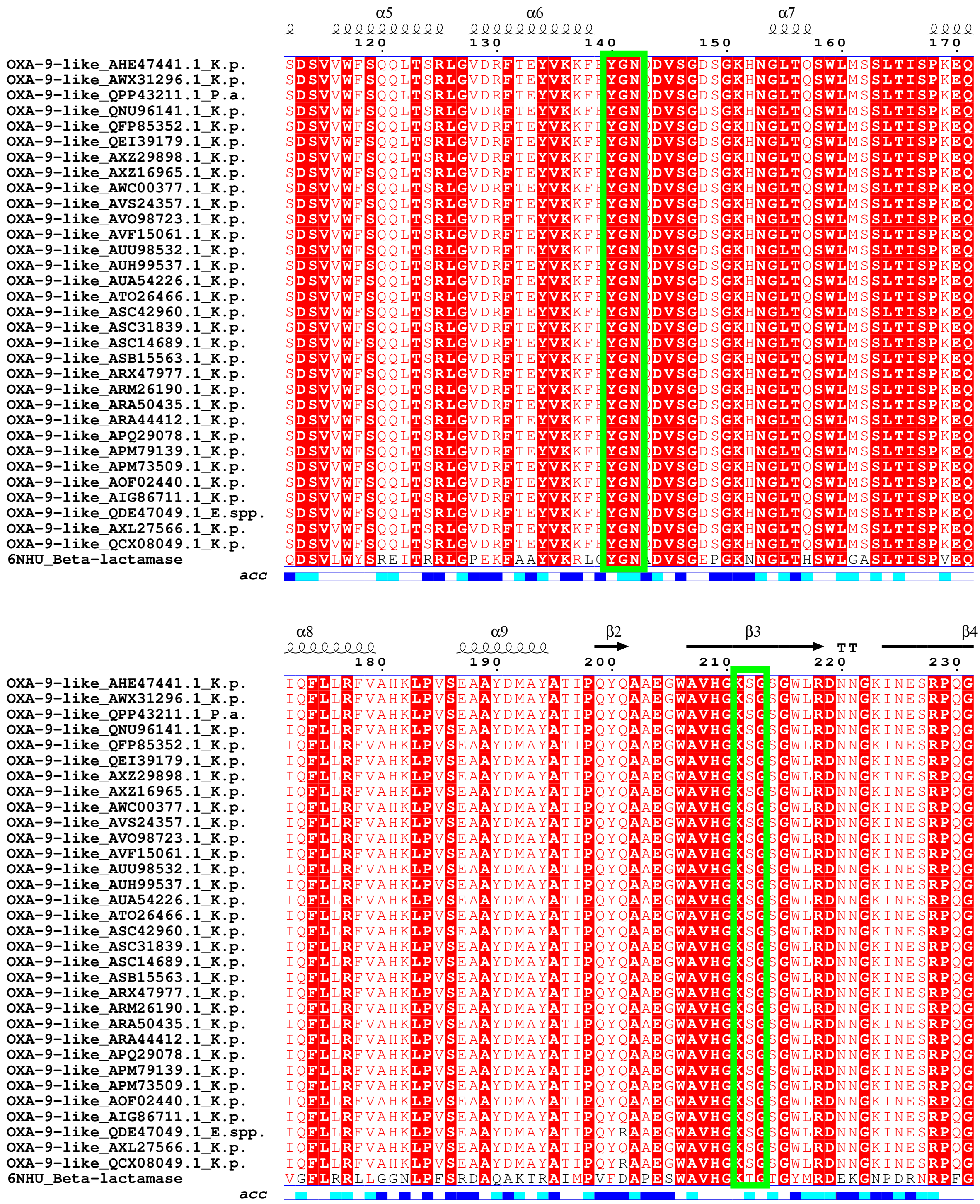

OXA-9-like subfamily

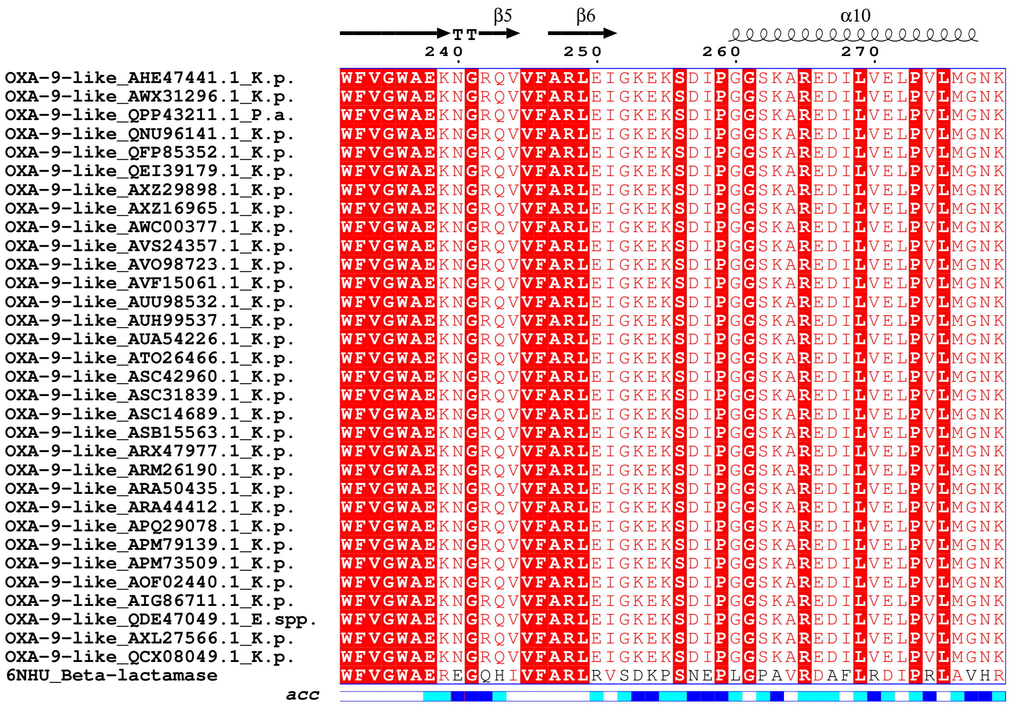

OXA-10-like subfamily

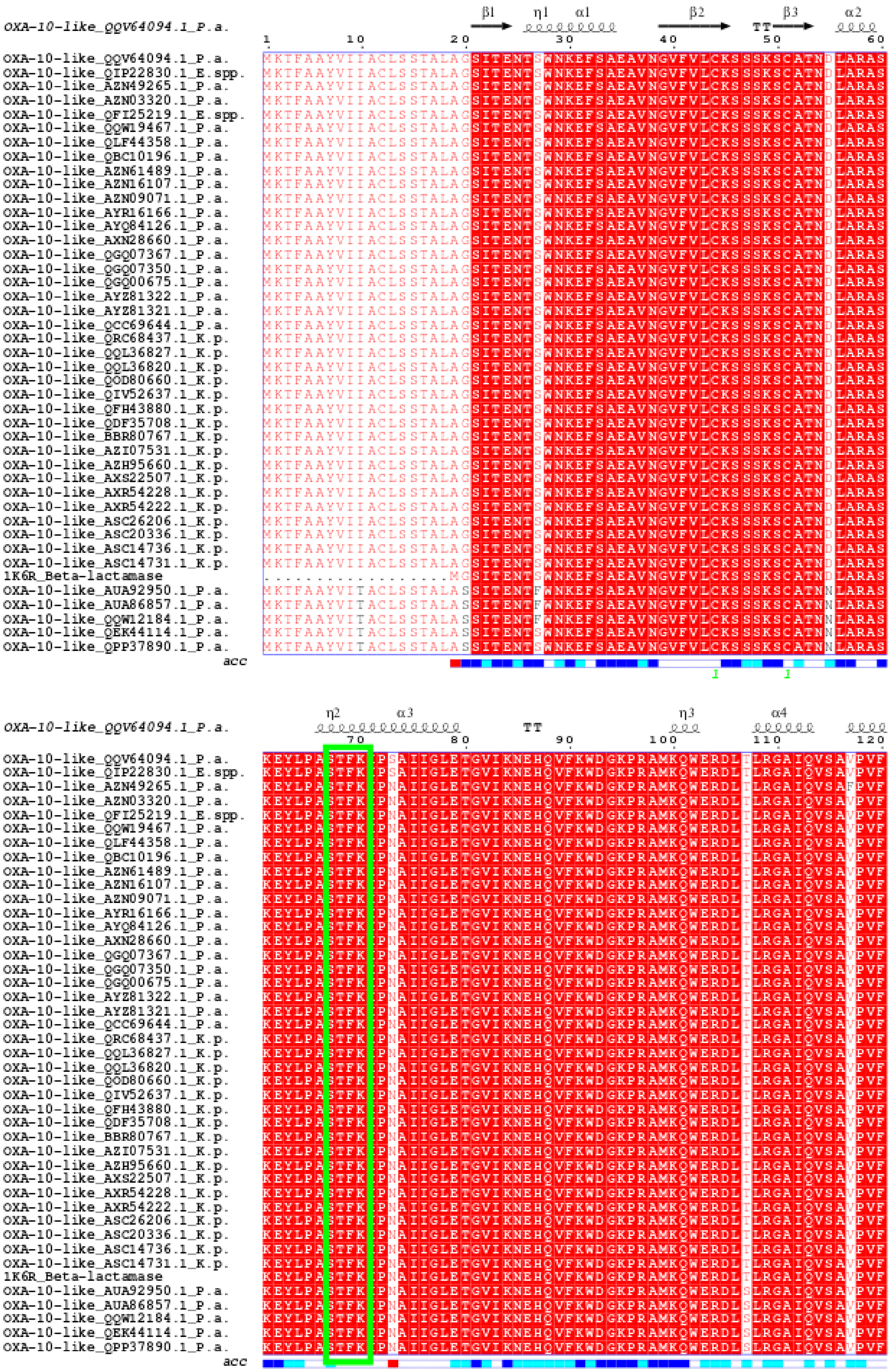

OXA-10-like subfamily

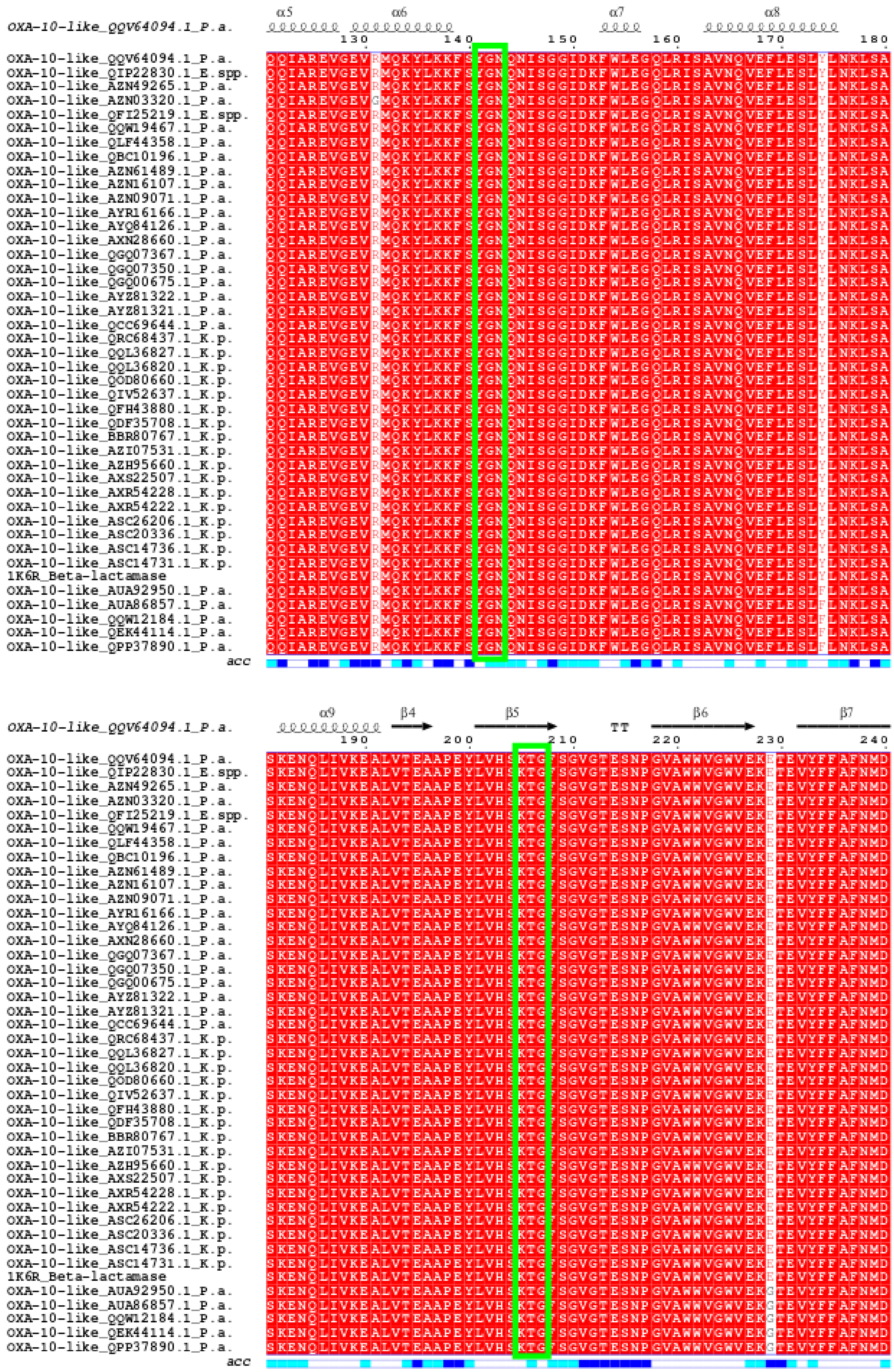

OXA-10-like subfamily

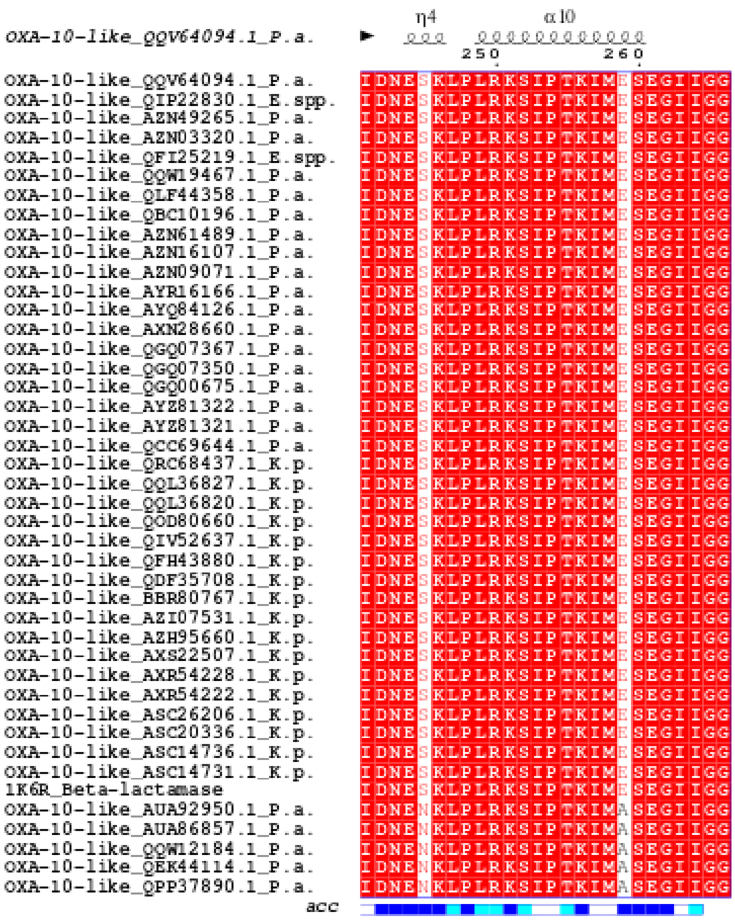

OXA-20-like subfamily

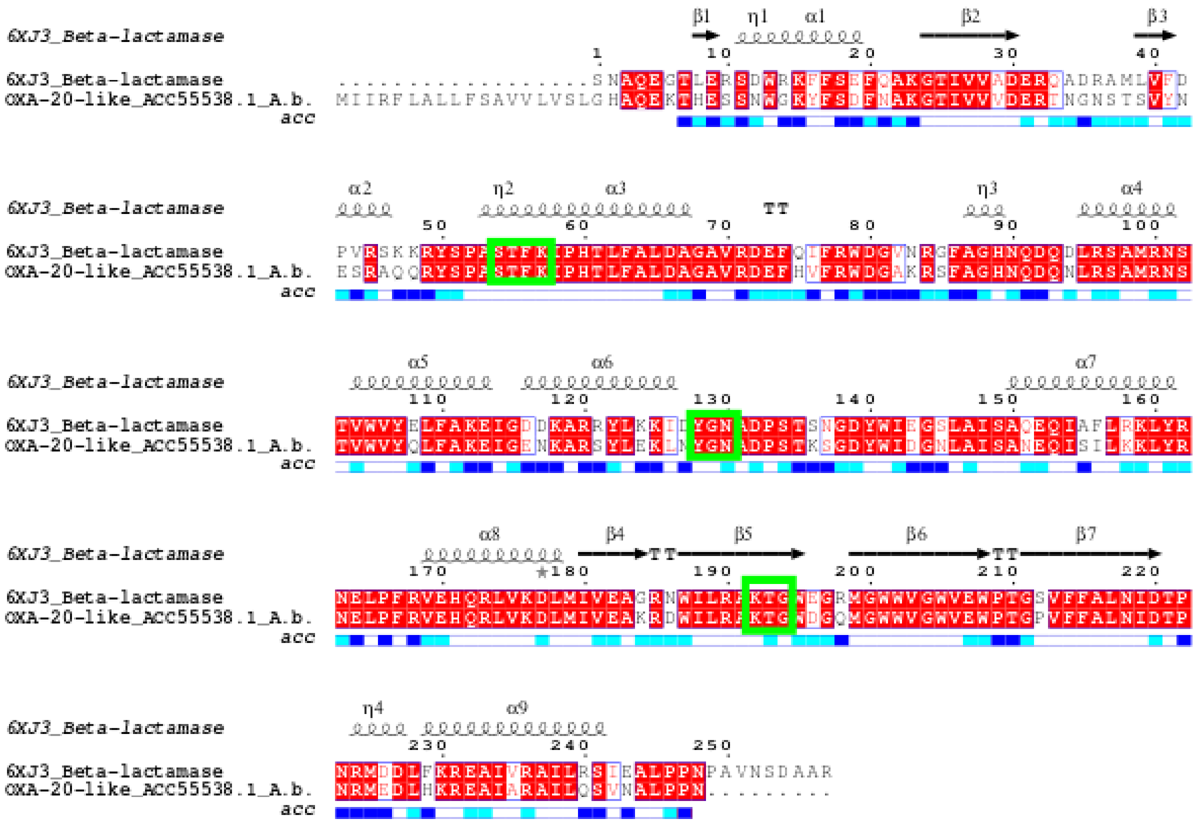

OXA-23-like subfamily

```
                                                                                   α1
OXA-23-like_QQE03160.1_A.b.
                                                 1        10        20        30        40
OXA-23-like_QQE03160.1_A.b.  ...................MNKYFTCYVVASLFLSGCTVQHNLINETPSQIVQGHNQVIH
OXA-23-like_QQE03411.1_A.b.  ...................MNKYFTCYVVASLFLSGCTVQHNLINETPSQIVQGHNQVIH
OXA-23-like_QQD99609.1_A.b.  ...................MNKYFTCYVVASLFLSGCTVQHNLINETPSQIVQGHNQVIH
OXA-23-like_QQD99362.1_A.b.  ...................MNKYFTCYVVASLFLSGCTVQHNLINETPSQIVQGHNQVIH
OXA-23-like_QQD95815.1_A.b.  ...................MNKYFTCYVVASLFLSGCTVQHNLINETPSQIVQGHNQVIH
OXA-23-like_QQD95566.1_A.b.  ...................MNKYFTCYVVASLFLSGCTVQHNLINETPSQIVQGHNQVIH
OXA-23-like_QQD93073.1_A.b.  ...................MNKYFTCYVVASLFLSGCTVQHNLINETPSQIVQGHNQVIH
OXA-23-like_QQD92824.1_A.b.  ...................MNKYFTCYVVASLFLSGCTVQHNLINETPSQIVQGHNQVIH
OXA-23-like_QQB67579.1_A.b.  ...................MNKYFTCYVVASLFLSGCTVQHNLINETPSQIVQGHNQVIH
OXA-23-like_QQB65684.1_A.b.  ...................MNKYFTCYVVASLFLSGCTVQHNLINETPSQIVQGHNQVIH
OXA-23-like_QPO03745.1_A.b.  ...................MNKYFTCYVVASLFLSGCTVQHNLINETPSQIVQGHNQVIH
OXA-23-like_QPO00239.1_A.b.  ...................MNKYFTCYVVASLFLSGCTVQHNLINETPSQIVQGHNQVIH
OXA-23-like_QLG80230.1_A.b.  ...................MNKYFTCYVVASLFLSGCTVQHNLINETPSQIVQGHNQVIH
OXA-23-like_QLA71954.1_A.b.  ...................MNKYFTCYVVASLFLSGCTVQHNLINETPSQIVQGHNQVIH
OXA-23-like_QJP32450.1_A.b.  ...................MNKYFTCYVVASLFLSGCTVQHNLINETPSQIVQGHNQVIH
OXA-23-like_QJH23301.1_A.b.  ...................MNKYFTCYVVASLFLSGCTVQHNLINETPSQIVQGHNQVIH
OXA-23-like_QJH19267.1_A.b.  ...................MNKYFTCYVVASLFLSGCTVQHNLINETPSQIVQGHNQVIH
OXA-23-like_QJH16482.1_A.b.  ...................MNKYFTCYVVASLFLSGCTVQHNLINETPSQIVQGHNQVIH
OXA-23-like_QJH15745.1_A.b.  ...................MNKYFTCYVVASLFLSGCTVQHNLINETPSQIVQGHNQVIH
OXA-23-like_QJH15717.1_A.b.  ...................MNKYFTCYVVASLFLSGCTVQHNLINETPSQIVQGHNQVIH
OXA-23-like_QJH08361.1_A.b.  ...................MNKYFTCYVVASLFLSGCTVQHNLINETPSQIVQGHNQVIH
OXA-23-like_QJH12331.1_A.b.  ...................MNKYFTCYVVASLFLSGCTVQHNLINETPSQIVQGHNQVIH
OXA-23-like_QJH05456.1_A.b.  ...................MNKYFTCYVVASLFLSGCTVQHNLINETPSQIVQGHNQVIH
OXA-23-like_QJH00715.1_A.b.  ...................MNKYFTCYVVASLFLSGCTVQHNLINETPSQIVQGHNQVIH
OXA-23-like_QJG95773.1_A.b.  ...................MNKYFTCYVVASLFLSGCTVQHNLINETPSQIVQGHNQVIH
OXA-23-like_QJG96932.1_A.b.  ...................MNKYFTCYVVASLFLSGCTVQHNLINETPSQIVQGHNQVIH
OXA-23-like_QJG96957.1_A.b.  ...................MNKYFTCYVVASLFLSGCTVQHNLINETPSQIVQGHNQVIH
OXA-23-like_QJH00689.1_A.b.  ...................MNKYFTCYVVASLFLSGCTVQHNLINETPSQIVQGHNQVIH
OXA-23-like_AOX88011.1_A.b.  ...................MNKYFTCYVVASLFLSGCTVQHNLINETPSQIVQGHNQVIH
OXA-23-like_AOX91849.1_A.b.  ...................MNKYFTCYVVASLFLSGCTVQHNLINETPSQIVQGHNQVIH
OXA-23-like_AOX95708.1_A.b.  ...................MNKYFTCYVVASLFLSGCTVQHNLINETPSQIVQGHNQVIH
OXA-23-like_APM50633.1_A.b.  ...................MNKYFTCYVVASLFLSGCTVQHNLINETPSQIVQGHNQVIH
OXA-23-like_APM50651.1_A.b.  ...................MNKYFTCYVVASLFLSGCTVQHNLINETPSQIVQGHNQVIH
OXA-23-like_AQU56756.1_A.b.  ...................MNKYFTCYVVASLFLSGCTVQHNLINETPSQIVQGHNQVIH
OXA-23-like_AQU56788.1_A.b.  ...................MNKYFTCYVVASLFLSGCTVQHNLINETPSQIVQGHNQVIH
OXA-23-like_ARF92486.1_A.b.  ...................MNKYFTCYVVASLFLSGCTVQHNLINETPSQIVQGHNQVIH
OXA-23-like_ARF92497.1_A.b.  ...................MNKYFTCYVVASLFLSGCTVQHNLINETPSQIVQGHNQVIH
OXA-23-like_ARF95290.1_A.b.  ...................MNKYFTCYVVASLFLSGCTVQHNLINETPSQIVQGHNQVIH
OXA-23-like_ARF96125.1_A.b.  ...................MNKYFTCYVVASLFLSGCTVQHNLINETPSQIVQGHNQVIH
OXA-23-like_ARG05487.1_A.b.  ...................MNKYFTCYVVASLFLSGCTVQHNLINETPSQIVQGHNQVIH
OXA-23-like_ARG10150.1_A.b.  ...................MNKYFTCYVVASLFLSGCTVQHNLINETPSQIVQGHNQVIH
OXA-23-like_ARG12999.1_A.b.  ...................MNKYFTCYVVASLFLSGCTVQHNLINETPSQIVQGHNQVIH
OXA-23-like_ARG19192.1_A.b.  ...................MNKYFTCYVVASLFLSGCTVQHNLINETPSQIVQGHNQVIH
OXA-23-like_ARG19219.1_A.b.  ...................MNKYFTCYVVASLFLSGCTVQHNLINETPSQIVQGHNQVIH
OXA-23-like_ARG28873.1_A.b.  ...................MNKYFTCYVVASLFLSGCTVQHNLINETPSQIVQGHNQVIH
OXA-23-like_ARG28898.1_A.b.  ...................MNKYFTCYVVASLFLSGCTVQHNLINETPSQIVQGHNQVIH
OXA-23-like_ARG30155.1_A.b.  ...................MNKYFTCYVVASLFLSGCTVQHNLINETPSQIVQGHNQVIH
OXA-23-like_ARG35250.1_A.b.  ...................MNKYFTCYVVASLFLSGCTVQHNLINETPSQIVQGHNQVIH
OXA-23-like_ASF49900.1_A.b.  ...................MNKYFTCYVVASLFLSGCTVQHNLINETPSQIVQGHNQVIH
OXA-23-like_AUG13479.1_A.b.  ...................MNKYFTCYVVASLFLSGCTVQHNLINETPSQIVQGHNQVIH
OXA-23-like_AVN25815.1_A.b.  ...................MNKYFTCYVVASLFLSGCTVQHNLINETPSQIVQGHNQVIH
OXA-23-like_AVN25876.1_A.b.  ...................MNKYFTCYVVASLFLSGCTVQHNLINETPSQIVQGHNQVIH
OXA-23-like_AVN25910.1_A.b.  ...................MNKYFTCYVVASLFLSGCTVQHNLINETPSQIVQGHNQVIH
OXA-23-like_AWW79127.1_A.b.  ...................MNKYFTCYVVASLFLSGCTVQHNLINETPSQIVQGHNQVIH
OXA-23-like_AWW83877.1_A.b.  ...................MNKYFTCYVVASLFLSGCTVQHNLINETPSQIVQGHNQVIH
OXA-23-like_AWW86703.1_A.b.  ...................MNKYFTCYVVASLFLSGCTVQHNLINETPSQIVQGHNQVIH
OXA-23-like_AYY88859.1_A.b.  ...................MNKYFTCYVVASLFLSGCTVQHNLINETPSQIVQGHNQVIH
OXA-23-like_BAP68327.1_A.b.  ...................MNKYFTCYVVASLFLSGCTVQHNLINETPSQIVQGHNQVIH
OXA-23-like_QAS95332.1_A.b.  ...................MNKYFTCYVVASLFLSGCTVQHNLINETPSQIVQGHNQVIH
OXA-23-like_QAT02983.1_A.b.  ...................MNKYFTCYVVASLFLSGCTVQHNLINETPSQIVQGHNQVIH
OXA-23-like_QBY15339.1_A.b.  ...................MNKYFTCYVVASLFLSGCTVQHNLINETPSQIVQGHNQVIH
OXA-23-like_QCH32733.1_A.b.  ...................MNKYFTCYVVASLFLSGCTVQHNLINETPSQIVQGHNQVIH
OXA-23-like_QCP17255.1_A.b.  ...................MNKYFTCYVVASLFLSGCTVQHNLINETPSQIVQGHNQVIH
OXA-23-like_QCP24705.1_A.b.  ...................MNKYFTCYVVASLFLSGCTVQHNLINETPSQIVQGHNQVIH
OXA-23-like_QCP27215.1_A.b.  ...................MNKYFTCYVVASLFLSGCTVQHNLINETPSQIVQGHNQVIH
OXA-23-like_QCP29558.1_A.b.  ...................MNKYFTCYVVASLFLSGCTVQHNLINETPSQIVQGHNQVIH
OXA-23-like_QCP40778.1_A.b.  ...................MNKYFTCYVVASLFLSGCTVQHNLINETPSQIVQGHNQVIH
OXA-23-like_QCP43779.1_A.b.  ...................MNKYFTCYVVASLFLSGCTVQHNLINETPSQIVQGHNQVIH
OXA-23-like_QCP44095.1_A.b.  ...................MNKYFTCYVVASLFLSGCTVQHNLINETPSQIVQGHNQVIH
OXA-23-like_QCP47140.1_A.b.  ...................MNKYFTCYVVASLFLSGCTVQHNLINETPSQIVQGHNQVIH
OXA-23-like_QCR88532.1_A.b.  ...................MNKYFTCYVVASLFLSGCTVQHNLINETPSQIVQGHNQVIH
OXA-23-like_QDR92294.1_A.b.  ...................MNKYFTCYVVASLFLSGCTVQHNLINETPSQIVQGHNQVIH
OXA-23-like_QDR95367.1_A.b.  ...................MNKYFTCYVVASLFLSGCTVQHNLINETPSQIVQGHNQVIH
OXA-23-like_QEE57736.1_A.b.  ...................MNKYFTCYVVASLFLSGCTVQHNLINETPSQIVQGHNQVIH
OXA-23-like_QEE57740.1_A.b.  ...................MNKYFTCYVVASLFLSGCTVQHNLINETPSQIVQGHNQVIH
OXA-23-like_QEE57744.1_A.b.  ...................MNKYFTCYVVASLFLSGCTVQHNLINETPSQIVQGHNQVIH
OXA-23-like_QEY29291.1_A.b.  ...................MNKYFTCYVVASLFLSGCTVQHNLINETPSQIVQGHNQVIH
OXA-23-like_QEY29320.1_A.b.  ...................MNKYFTCYVVASLFLSGCTVQHNLINETPSQIVQGHNQVIH
OXA-23-like_QEY29496.1_A.b.  ...................MNKYFTCYVVASLFLSGCTVQHNLINETPSQIVQGHNQVIH
OXA-23-like_QIX29121.1_A.b.  ...................MNKYFTCYVVASLFLSGCTVQHNLINETPSQIVQGHNQVIH
OXA-23-like_QIX32951.1_A.b.  ...................MNKYFTCYVVASLFLSGCTVQHNLINETPSQIVQGHNQVIH
OXA-23-like_QIX36912.1_A.b.  ...................MNKYFTCYVVASLFLSGCTVQHNLINETPSQIVQGHNQVIH
OXA-23-like_QIX40711.1_A.b.  ...................MNKYFTCYVVASLFLSGCTVQHNLINETPSQIVQGHNQVIH
OXA-23-like_QIX44509.1_A.b.  ...................MNKYFTCYVVASLFLSGCTVQHNLINETPSQIVQGHNQVIH
OXA-23-like_QJG69304.1_A.b.  ...................MNKYFTCYVVASLFLSGCTVQHNLINETPSQIVQGHNQVIH
OXA-23-like_QJG70467.1_A.b.  ...................MNKYFTCYVVASLFLSGCTVQHNLINETPSQIVQGHNQVIH
OXA-23-like_QJG70485.1_A.b.  ...................MNKYFTCYVVASLFLSGCTVQHNLINETPSQIVQGHNQVIH
OXA-23-like_QJG71265.1_A.b.  ...................MNKYFTCYVVASLFLSGCTVQHNLINETPSQIVQGHNQVIH
OXA-23-like_QJG74213.1_A.b.  ...................MNKYFTCYVVASLFLSGCTVQHNLINETPSQIVQGHNQVIH
OXA-23-like_QJG81833.1_A.b.  ...................MNKYFTCYVVASLFLSGCTVQHNLINETPSQIVQGHNQVIH
OXA-23-like_QJG84680.1_A.b.  ...................MNKYFTCYVVASLFLSGCTVQHNLINETPSQIVQGHNQVIH
OXA-23-like_QJG89564.1_A.b.  ...................MNKYFTCYVVASLFLSGCTVQHNLINETPSQIVQGHNQVIH
OXA-23-like_QJG89584.1_A.b.  ...................MNKYFTCYVVASLFLSGCTVQHNLINETPSQIVQGHNQVIH
OXA-23-like_QJG90447.1_A.b.  ...................MNKYFTCYVVASLFLSGCTVQHNLINETPSQIVQGHNQVIH
OXA-23-like_QJG93290.1_A.b.  ...................MNKYFTCYVVASLFLSGCTVQHNLINETPSQIVQGHNQVIH
OXA-23-like_ADX93021.1_A.b.  ................MFKMNKYFTCYVVASLFLSGCTVQHNLINETPSQIVQGHNQVIH
OXA-23-like_AXG83856.1_A.b.  ...................MNKYFTCYVVASLFLSGCTVQHNLINETPSQIVQGHNQVIH
OXA-23-like_AML68547.1_A.b.  ...................MNKYFTCYVVASLFLSGCTVQHNLINETPSQIVQGHNQVIH
OXA-23-like_ARG03241.1_A.b.  ...................MNKYFTCYVVASLFLSGCTVQHNLINETPSQIVQGHNQVIH
OXA-23-like_AOX84133.1_A.b.  ...................MNKYFTCYVVASLFLSGCTVQHNLINETPSQIVQGHNQVIH
OXA-23-like_AOX82595.1_A.b.  ...................MNKYFTCYVVASLFLSGCTVQHNLINETPSQIVQGHNQVIH
OXA-23-like_AOX75920.1_A.b.  ...................MNKYFTCYVVASLFLSGCTVQHNLINETPSQIVQGHNQVIH
OXA-23-like_AOX75888.1_A.b.  ...................MNKYFTCYVVASLFLSGCTVQHNLINETPSQIVQGHNQVIH
```

OXA-20-like subfamily

```
OXA-23-like_AOX72443.1_A.b.  ...................MNKYFTCYVVASLFLSGCTVQHNLINETPSQIVQGHNQVIH
OXA-23-like_AOX68171.1_A.b.  ...................MNKYFTCYVVASLFLSGCTVQHNLINETPSQIVQGHNQVIH
OXA-23-like_AMC14310.1_A.b.  ...................MNKYFTCYVVASLFLSGCTVQHNLINETPSQIVQGHNQVIH
OXA-23-like_AMC14301.1_A.b.  ...................MNKYFTCYVVASLFLSGCTVQHNLINETPSQIVQGHNQVIH
OXA-23-like_ALY00722.1_A.b.  ...................MNKYFTCYVVASLFLSGCTVQHNLINETPSQIVQGHNQVIH
OXA-23-like_ALX98871.1_A.b.  ...................MNKYFTCYVVASLFLSGCTVQHNLINETPSQIVQGHNQVIH
OXA-23-like_AKA30333.1_A.b.  ...................MNKYFTCYVVASLFLSGCTVQHNLINETPSQIVQGHNQVIH
OXA-23-like_AGQ10499.1_A.b.  ...................MNKYFTCYVVASLFLSGCTVQHNLINETPSQIVQGHNQVIH
OXA-23-like_AGQ05635.1_A.b.  ...................MNKYFTCYVVASLFLSGCTVQHNLINETPSQIVQGHNQVIH
OXA-23-like_ACJ39972.1_A.b.  ...................MNKYFTCYVVASLFLSGCTVQHNLINETPSQIVQGHNQVIH
OXA-23-like_QKK76748.1_A.b.  ...................MNKYFTCYVVASLFLSGCTVQHNLINETPSQIVQGHNQVIH
OXA-23-like_QKK76707.1_A.b.  ...................MNKYFTCYVVASLFLSGCTVQHNLINETPSQIVQGHNQVIH
OXA-23-like_QKK76393.1_A.b.  ...................MNKYFTCYVVASLFLSGCTVQHNLINETPSQIVQGHNQVIH
OXA-23-like_QFX72557.1_A.b.  ...................MNKYFTCYVVASLFLSGCTVQHNLINETPSQIVQGHNQVIH
OXA-23-like_QFZ56693.1_A.b.  ...................MNKYFTCYVVASLFLSGCTVQHNLINETPSQIVQGHNQVIH
OXA-23-like_AYC00513.1_A.b.  ...................MNKYFTCYVVASLFLSGCTVQHNLINETPSQIVQGHNQVIH
OXA-23-like_AYC00505.1_A.b.  ...................MNKYFTCYVVASLFLSGCTVQHNLINETPSQIVQGHNQVIH
OXA-23-like_ARG39357.1_A.b.  ...................MNKYFTCYVVASLFLSGCTVQHNLINETPSQIVQGHNQVIH
OXA-23-like_ARG23739.1_A.b.  ...................MNKYFTCYVVASLFLSGCTVQHNLINETPSQIVQGHNQVIH
OXA-23-like_ARG23713.1_A.b.  ...................MNKYFTCYVVASLFLSGCTVQHNLINETPSQIVQGHNQVIH
OXA-23-like_ARG03186.1_A.b.  ...................MNKYFTCYVVASLFLSGCTVQHNLINETPSQIVQGHNQVIH
OXA-23-like_AEP06450.2_A.b.  ...................MNKYFTCYVVASLFLSGCTVQHNLINETPSQIVQGHNQVIH
OXA-23-like_AML75286.1_A.b.  ...................MNKYFTCYVVASLFLSGCTVQHNLINETPSQIVQGHNQVIH
OXA-23-like_AML75277.1_A.b.  ...................MNKYFTCYVVASLFLSGCTVQHNLINETPSQIVQGHNQVIH
OXA-23-like_AML71526.1_A.b.  ...................MNKYFTCYVVASLFLSGCTVQHNLINETPSQIVQGHNQVIH
OXA-23-like_AML69469.1_A.b.  ...................MNKYFTCYVVASLFLSGCTVQHNLINETPSQIVQGHNQVIH
OXA-23-like_AML66436.1_A.b.  ...................MNKYFTCYVVASLFLSGCTVQHNLINETPSQIVQGHNQVIH
OXA-23-like_AML65160.1_A.b.  ...................MNKYFTCYVVASLFLSGCTVQHNLINETPSQIVQGHNQVIH
OXA-23-like_AML65128.1_A.b.  ...................MNKYFTCYVVASLFLSGCTVQHNLINETPSQIVQGHNQVIH
OXA-23-like_AMN00602.1_A.b.  ...................MNKYFTCYVVASLFLSGCTVQHNLINETPSQIVQGHNQVIH
OXA-23-like_AMN00587.1_A.b.  ...................MNKYFTCYVVASLFLSGCTVQHNLINETPSQIVQGHNQVIH
OXA-23-like_AMN00578.1_A.b.  ...................MNKYFTCYVVASLFLSGCTVQHNLINETPSQIVQGHNQVIH
OXA-23-like_AMN00569.1_A.b.  ...................MNKYFTCYVVASLFLSGCTVQHNLINETPSQIVQGHNQVIH
OXA-23-like_QJF37605.1_A.b.  ...................MNKYFTCYVVASLFLSGCTVQHNLINETPSQIVQGHNQVIH
OXA-23-like_QJF33717.1_A.b.  ...................MNKYFTCYVVASLFLSGCTVQHNLINETPSQIVQGHNQVIH
OXA-23-like_QJH05102.1_A.b.  ...................MNKYFTCYVVASLFLSGCTVQHNLINETPSQIVQGHNQVIH
OXA-23-like_QJH23846.1_A.b.  ...................MNKYFTCYVVASLFLSGCTVQHNLINETPSQIVQGHNQVIH
OXA-23-like_QCH35083.1_A.b.  ...................MNKYFTCYVVASLFLSGCTVQHNLINETPSQIVQGHNQVIH
OXA-23-like_QAT07029.1_A.b.  ...................MNKYFTCYVVASLFLSGCTVQHNLINETPSQIVQGHNQVIH
OXA-23-like_QAS99734.1_A.b.  ...................MNKYFTCYVVASLFLSGCTVQHNLINETPSQIVQGHNQVIH
OXA-23-like_AVE56824.1_A.b.  ...................MNKYFTCYVVASLFLSGCTVQHNLINETPSQIVQGHNQVIH
OXA-23-like_AVE44374.1_A.b.  ...................MNKYFTCYVVASLFLSGCTVQHNLINETPSQIVQGHNQVIH
OXA-23-like_ATR89604.1_A.b.  ...................MNKYFTCYVVASLFLSGCTVQHNLINETPSQIVQGHNQVIH
OXA-23-like_ARG29802.1_A.b.  ...................MNKYFTCYVVASLFLSGCTVQHNLINETPSQIVQGHNQVIH
OXA-23-like_APF45746.1_A.b.  ...................MNKYFTCYVVASLFLSGCTVQHNLINETPSQIVQGHNQVIH
OXA-23-like_APQ95067.1_A.b.  ...................MNKYFTCYVVASLFLSGCTVQHNLINETPSQIVQGHNQVIH
OXA-23-like_APQ87413.1_A.b.  ...................MNKYFTCYVVASLFLSGCTVQHNLINETPSQIVQGHNQVIH
OXA-23-like_ATR88802.1_A.b.  MSYLFLSCTE...........KYFTCYVVASLFLSGCTVQHNLINETPSQIVQGHNQVIH
OXA-23-like_ATR88943.1_A.b.  MSYLFLSCTE...........KYFTCYVVASLFLSGCTVQHNLINETPSQIVQGHNQVIH
OXA-23-like_SBS23848.1_A.b.  MSYLFLSCTELFSIDLVFKMNKYFTCYVVASLFLSGCTVQHNLINETPSQIVQGHNQVIH
OXA-23-like_AOP62462.1_A.b.  MSYLFLSCTELFSIDLVFKMNKYFTCYVVASLFLSGCTVQHNLINETPSQIVQGHNQVIH
4JF4_Beta-lactamase          .................................................QIVQGHNQVIH
OXA-23-like_QCP18237.1_A.b.  .........................MWLLLFFFLSGCTVQHNLINETPSQIVQGHNQVIH
OXA-23-like_QRN19109.1_A.b.  .........................MWLLLFF.LSGCTVQHNLINETPSQIVQGHNQVIH
                        acc
```

OXA-23-like subfamily

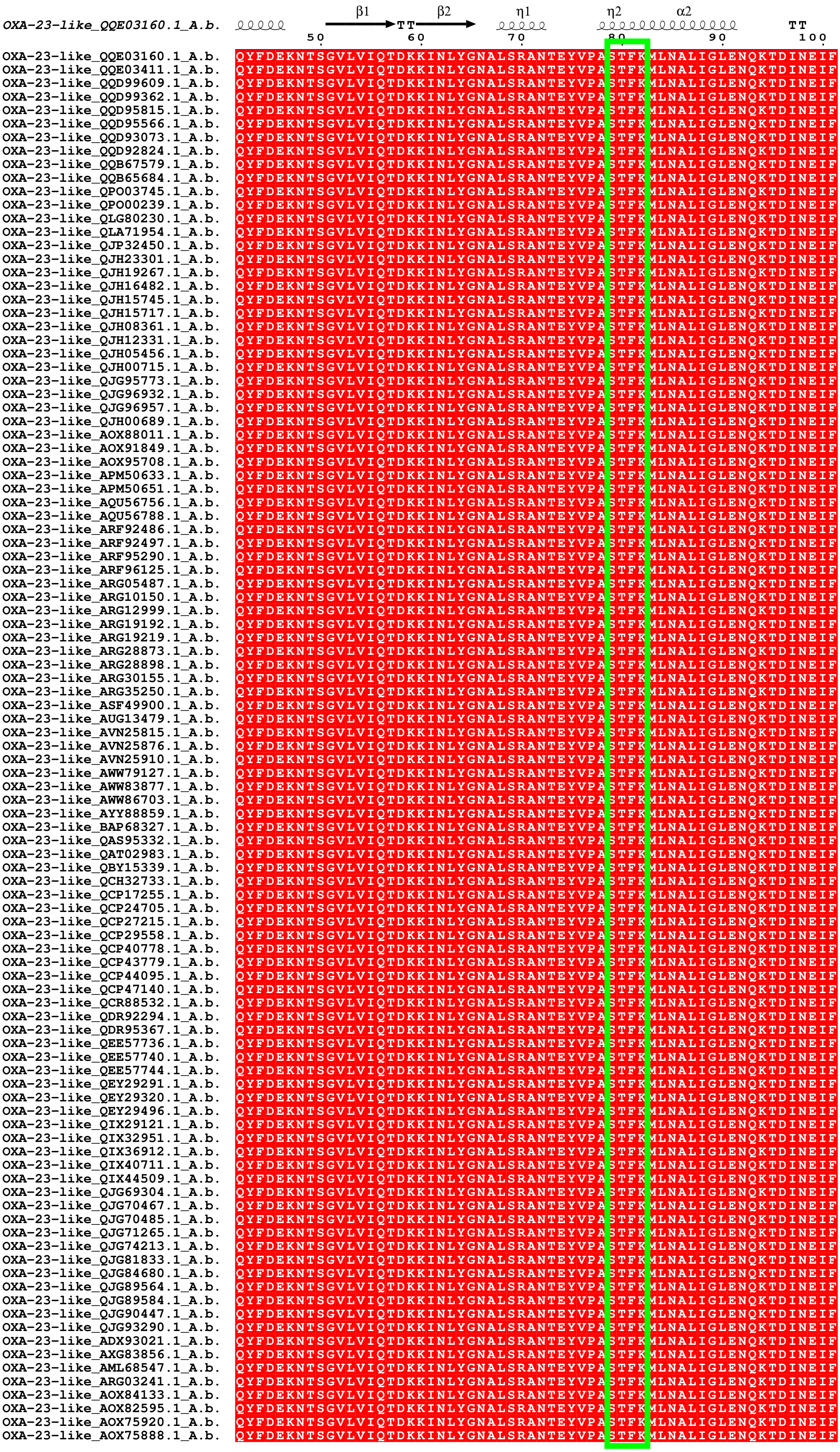

OXA-23-like subfamily

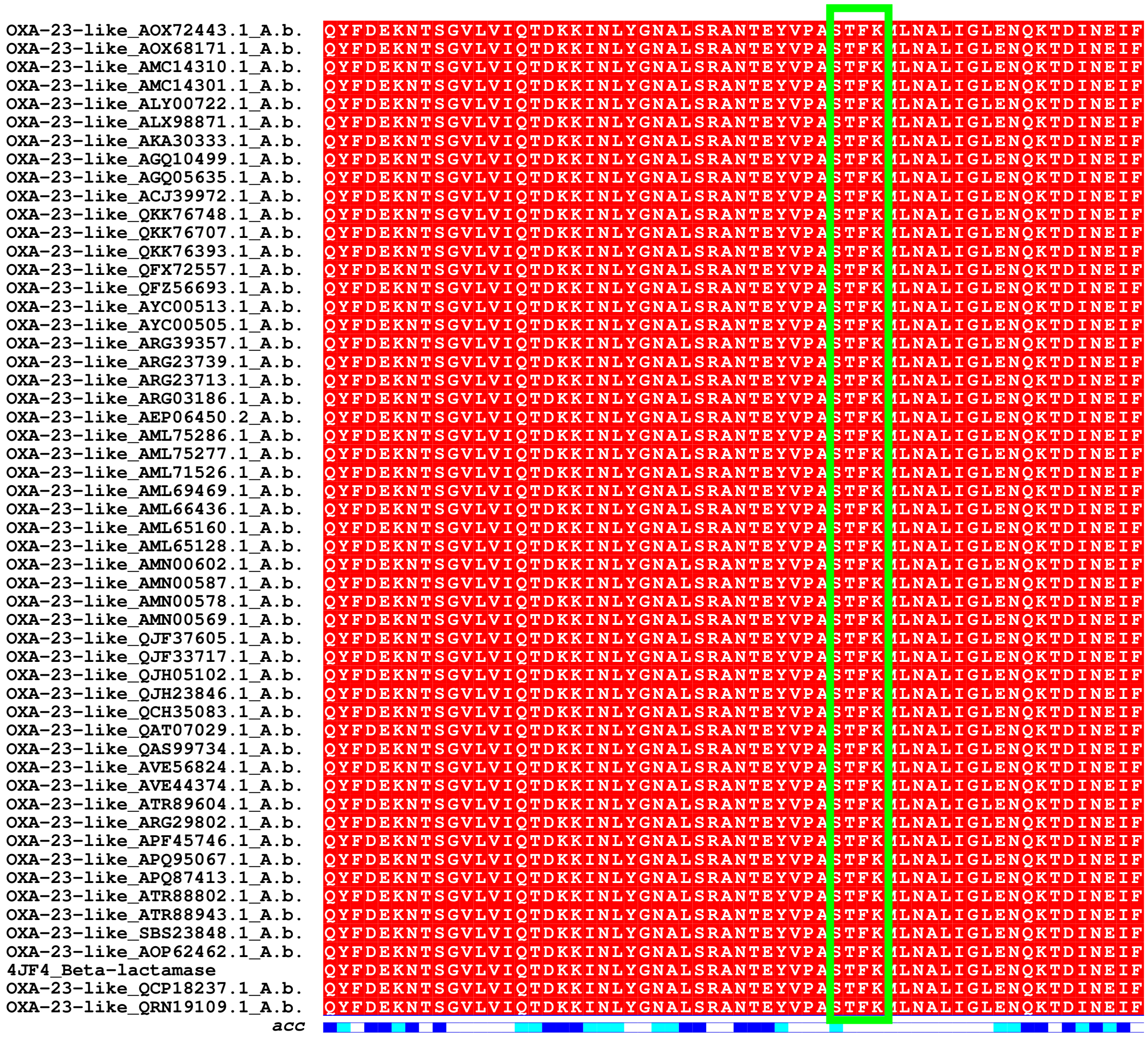

OXA-23-like subfamily

```
                                      η3        α3           α4           α5
OXA-23-like_QQE03160.1_A.b.
                                 110       120       130  ★    140       150       160
OXA-23-like_QQE03160.1_A.b.  KWKGEKRSFTAWEKDMTLGEAMKLSAVPVYQELARRIGLDLMQKEVKRIGFGNAEIGQQV
OXA-23-like_QQE03411.1_A.b.  KWKGEKRSFTAWEKDMTLGEAMKLSAVPVYQELARRIGLDLMQKEVKRIGFGNAEIGQQV
OXA-23-like_QQD99609.1_A.b.  KWKGEKRSFTAWEKDMTLGEAMKLSAVPVYQELARRIGLDLMQKEVKRIGFGNAEIGQQV
OXA-23-like_QQD99362.1_A.b.  KWKGEKRSFTAWEKDMTLGEAMKLSAVPVYQELARRIGLDLMQKEVKRIGFGNAEIGQQV
OXA-23-like_QQD95815.1_A.b.  KWKGEKRSFTAWEKDMTLGEAMKLSAVPVYQELARRIGLDLMQKEVKRIGFGNAEIGQQV
OXA-23-like_QQD95566.1_A.b.  KWKGEKRSFTAWEKDMTLGEAMKLSAVPVYQELARRIGLDLMQKEVKRIGFGNAEIGQQV
OXA-23-like_QQD93073.1_A.b.  KWKGEKRSFTAWEKDMTLGEAMKLSAVPVYQELARRIGLDLMQKEVKRIGFGNAEIGQQV
OXA-23-like_QQD92824.1_A.b.  KWKGEKRSFTAWEKDMTLGEAMKLSAVPVYQELARRIGLDLMQKEVKRIGFGNAEIGQQV
OXA-23-like_QQB67579.1_A.b.  KWKGEKRSFTAWEKDMTLGEAMKLSAVPVYQELARRIGLDLMQKEVKRIGFGNAEIGQQV
OXA-23-like_QQB65684.1_A.b.  KWKGEKRSFTAWEKDMTLGEAMKLSAVPVYQELARRIGLDLMQKEVKRIGFGNAEIGQQV
OXA-23-like_QPO03745.1_A.b.  KWKGEKRSFTAWEKDMTLGEAMKLSAVPVYQELARRIGLDLMQKEVKRIGFGNAEIGQQV
OXA-23-like_QPO00239.1_A.b.  KWKGEKRSFTAWEKDMTLGEAMKLSAVPVYQELARRIGLDLMQKEVKRIGFGNAEIGQQV
OXA-23-like_QLG80230.1_A.b.  KWKGEKRSFTAWEKDMTLGEAMKLSAVPVYQELARRIGLDLMQKEVKRIGFGNAEIGQQV
OXA-23-like_QLA71954.1_A.b.  KWKGEKRSFTAWEKDMTLGEAMKLSAVPVYQELARRIGLDLMQKEVKRIGFGNAEIGQQV
OXA-23-like_QJP32450.1_A.b.  KWKGEKRSFTAWEKDMTLGEAMKLSAVPVYQELARRIGLDLMQKEVKRIGFGNAEIGQQV
OXA-23-like_QJH23301.1_A.b.  KWKGEKRSFTAWEKDMTLGEAMKLSAVPVYQELARRIGLDLMQKEVKRIGFGNAEIGQQV
OXA-23-like_QJH19267.1_A.b.  KWKGEKRSFTAWEKDMTLGEAMKLSAVPVYQELARRIGLDLMQKEVKRIGFGNAEIGQQV
OXA-23-like_QJH16482.1_A.b.  KWKGEKRSFTAWEKDMTLGEAMKLSAVPVYQELARRIGLDLMQKEVKRIGFGNAEIGQQV
OXA-23-like_QJH15745.1_A.b.  KWKGEKRSFTAWEKDMTLGEAMKLSAVPVYQELARRIGLDLMQKEVKRIGFGNAEIGQQV
OXA-23-like_QJH15717.1_A.b.  KWKGEKRSFTAWEKDMTLGEAMKLSAVPVYQELARRIGLDLMQKEVKRIGFGNAEIGQQV
OXA-23-like_QJH08361.1_A.b.  KWKGEKRSFTAWEKDMTLGEAMKLSAVPVYQELARRIGLDLMQKEVKRIGFGNAEIGQQV
OXA-23-like_QJH12331.1_A.b.  KWKGEKRSFTAWEKDMTLGEAMKLSAVPVYQELARRIGLDLMQKEVKRIGFGNAEIGQQV
OXA-23-like_QJH05456.1_A.b.  KWKGEKRSFTAWEKDMTLGEAMKLSAVPVYQELARRIGLDLMQKEVKRIGFGNAEIGQQV
OXA-23-like_QJH00715.1_A.b.  KWKGEKRSFTAWEKDMTLGEAMKLSAVPVYQELARRIGLDLMQKEVKRIGFGNAEIGQQV
OXA-23-like_QJG95773.1_A.b.  KWKGEKRSFTAWEKDMTLGEAMKLSAVPVYQELARRIGLDLMQKEVKRIGFGNAEIGQQV
OXA-23-like_QJG96932.1_A.b.  KWKGEKRSFTAWEKDMTLGEAMKLSAVPVYQELARRIGLDLMQKEVKRIGFGNAEIGQQV
OXA-23-like_QJG96957.1_A.b.  KWKGEKRSFTAWEKDMTLGEAMKLSAVPVYQELARRIGLDLMQKEVKRIGFGNAEIGQQV
OXA-23-like_QJH00689.1_A.b.  KWKGEKRSFTAWEKDMTLGEAMKLSAVPVYQELARRIGLDLMQKEVKRIGFGNAEIGQQV
OXA-23-like_AOX88011.1_A.b.  KWKGEKRSFTAWEKDMTLGEAMKLSAVPVYQELARRIGLDLMQKEVKRIGFGNAEIGQQV
OXA-23-like_AOX91849.1_A.b.  KWKGEKRSFTAWEKDMTLGEAMKLSAVPVYQELARRIGLDLMQKEVKRIGFGNAEIGQQV
OXA-23-like_AOX95708.1_A.b.  KWKGEKRSFTAWEKDMTLGEAMKLSAVPVYQELARRIGLDLMQKEVKRIGFGNAEIGQQV
OXA-23-like_APM50633.1_A.b.  KWKGEKRSFTAWEKDMTLGEAMKLSAVPVYQELARRIGLDLMQKEVKRIGFGNAEIGQQV
OXA-23-like_APM50651.1_A.b.  KWKGEKRSFTAWEKDMTLGEAMKLSAVPVYQELARRIGLDLMQKEVKRIGFGNAEIGQQV
OXA-23-like_AQU56756.1_A.b.  KWKGEKRSFTAWEKDMTLGEAMKLSAVPVYQELARRIGLDLMQKEVKRIGFGNAEIGQQV
OXA-23-like_AQU56788.1_A.b.  KWKGEKRSFTAWEKDMTLGEAMKLSAVPVYQELARRIGLDLMQKEVKRIGFGNAEIGQQV
OXA-23-like_ARF92486.1_A.b.  KWKGEKRSFTAWEKDMTLGEAMKLSAVPVYQELARRIGLDLMQKEVKRIGFGNAEIGQQV
OXA-23-like_ARF92497.1_A.b.  KWKGEKRSFTAWEKDMTLGEAMKLSAVPVYQELARRIGLDLMQKEVKRIGFGNAEIGQQV
OXA-23-like_ARF95290.1_A.b.  KWKGEKRSFTAWEKDMTLGEAMKLSAVPVYQELARRIGLDLMQKEVKRIGFGNAEIGQQV
OXA-23-like_ARF96125.1_A.b.  KWKGEKRSFTAWEKDMTLGEAMKLSAVPVYQELARRIGLDLMQKEVKRIGFGNAEIGQQV
OXA-23-like_ARG05487.1_A.b.  KWKGEKRSFTAWEKDMTLGEAMKLSAVPVYQELARRIGLDLMQKEVKRIGFGNAEIGQQV
OXA-23-like_ARG10150.1_A.b.  KWKGEKRSFTAWEKDMTLGEAMKLSAVPVYQELARRIGLDLMQKEVKRIGFGNAEIGQQV
OXA-23-like_ARG12999.1_A.b.  KWKGEKRSFTAWEKDMTLGEAMKLSAVPVYQELARRIGLDLMQKEVKRIGFGNAEIGQQV
OXA-23-like_ARG19192.1_A.b.  KWKGEKRSFTAWEKDMTLGEAMKLSAVPVYQELARRIGLDLMQKEVKRIGFGNAEIGQQV
OXA-23-like_ARG19219.1_A.b.  KWKGEKRSFTAWEKDMTLGEAMKLSAVPVYQELARRIGLDLMQKEVKRIGFGNAEIGQQV
OXA-23-like_ARG28873.1_A.b.  KWKGEKRSFTAWEKDMTLGEAMKLSAVPVYQELARRIGLDLMQKEVKRIGFGNAEIGQQV
OXA-23-like_ARG28898.1_A.b.  KWKGEKRSFTAWEKDMTLGEAMKLSAVPVYQELARRIGLDLMQKEVKRIGFGNAEIGQQV
OXA-23-like_ARG30155.1_A.b.  KWKGEKRSFTAWEKDMTLGEAMKLSAVPVYQELARRIGLDLMQKEVKRIGFGNAEIGQQV
OXA-23-like_ARG35250.1_A.b.  KWKGEKRSFTAWEKDMTLGEAMKLSAVPVYQELARRIGLDLMQKEVKRIGFGNAEIGQQV
OXA-23-like_ASF49900.1_A.b.  KWKGEKRSFTAWEKDMTLGEAMKLSAVPVYQELARRIGLDLMQKEVKRIGFGNAEIGQQV
OXA-23-like_AUG13479.1_A.b.  KWKGEKRSFTAWEKDMTLGEAMKLSAVPVYQELARRIGLDLMQKEVKRIGFGNAEIGQQV
OXA-23-like_AVN25815.1_A.b.  KWKGEKRSFTAWEKDMTLGEAMKLSAVPVYQELARRIGLDLMQKEVKRIGFGNAEIGQQV
OXA-23-like_AVN25876.1_A.b.  KWKGEKRSFTAWEKDMTLGEAMKLSAVPVYQELARRIGLDLMQKEVKRIGFGNAEIGQQV
OXA-23-like_AVN25910.1_A.b.  KWKGEKRSFTAWEKDMTLGEAMKLSAVPVYQELARRIGLDLMQKEVKRIGFGNAEIGQQV
OXA-23-like_AWW79127.1_A.b.  KWKGEKRSFTAWEKDMTLGEAMKLSAVPVYQELARRIGLDLMQKEVKRIGFGNAEIGQQV
OXA-23-like_AWW83877.1_A.b.  KWKGEKRSFTAWEKDMTLGEAMKLSAVPVYQELARRIGLDLMQKEVKRIGFGNAEIGQQV
OXA-23-like_AWW86703.1_A.b.  KWKGEKRSFTAWEKDMTLGEAMKLSAVPVYQELARRIGLDLMQKEVKRIGFGNAEIGQQV
OXA-23-like_AYY88859.1_A.b.  KWKGEKRSFTAWEKDMTLGEAMKLSAVPVYQELARRIGLDLMQKEVKRIGFGNAEIGQQV
OXA-23-like_BAP68327.1_A.b.  KWKGEKRSFTAWEKDMTLGEAMKLSAVPVYQELARRIGLDLMQKEVKRIGFGNAEIGQQV
OXA-23-like_QAS95332.1_A.b.  KWKGEKRSFTAWEKDMTLGEAMKLSAVPVYQELARRIGLDLMQKEVKRIGFGNAEIGQQV
OXA-23-like_QAT02983.1_A.b.  KWKGEKRSFTAWEKDMTLGEAMKLSAVPVYQELARRIGLDLMQKEVKRIGFGNAEIGQQV
OXA-23-like_QBY15339.1_A.b.  KWKGEKRSFTAWEKDMTLGEAMKLSAVPVYQELARRIGLDLMQKEVKRIGFGNAEIGQQV
OXA-23-like_QCH32733.1_A.b.  KWKGEKRSFTAWEKDMTLGEAMKLSAVPVYQELARRIGLDLMQKEVKRIGFGNAEIGQQV
OXA-23-like_QCP17255.1_A.b.  KWKGEKRSFTAWEKDMTLGEAMKLSAVPVYQELARRIGLDLMQKEVKRIGFGNAEIGQQV
OXA-23-like_QCP24705.1_A.b.  KWKGEKRSFTAWEKDMTLGEAMKLSAVPVYQELARRIGLDLMQKEVKRIGFGNAEIGQQV
OXA-23-like_QCP27215.1_A.b.  KWKGEKRSFTAWEKDMTLGEAMKLSAVPVYQELARRIGLDLMQKEVKRIGFGNAEIGQQV
OXA-23-like_QCP29558.1_A.b.  KWKGEKRSFTAWEKDMTLGEAMKLSAVPVYQELARRIGLDLMQKEVKRIGFGNAEIGQQV
OXA-23-like_QCP40778.1_A.b.  KWKGEKRSFTAWEKDMTLGEAMKLSAVPVYQELARRIGLDLMQKEVKRIGFGNAEIGQQV
OXA-23-like_QCP43779.1_A.b.  KWKGEKRSFTAWEKDMTLGEAMKLSAVPVYQELARRIGLDLMQKEVKRIGFGNAEIGQQV
OXA-23-like_QCP44095.1_A.b.  KWKGEKRSFTAWEKDMTLGEAMKLSAVPVYQELARRIGLDLMQKEVKRIGFGNAEIGQQV
OXA-23-like_QCP47140.1_A.b.  KWKGEKRSFTAWEKDMTLGEAMKLSAVPVYQELARRIGLDLMQKEVKRIGFGNAEIGQQV
OXA-23-like_QCR88532.1_A.b.  KWKGEKRSFTAWEKDMTLGEAMKLSAVPVYQELARRIGLDLMQKEVKRIGFGNAEIGQQV
OXA-23-like_QDR92294.1_A.b.  KWKGEKRSFTAWEKDMTLGEAMKLSAVPVYQELARRIGLDLMQKEVKRIGFGNAEIGQQV
OXA-23-like_QDR95367.1_A.b.  KWKGEKRSFTAWEKDMTLGEAMKLSAVPVYQELARRIGLDLMQKEVKRIGFGNAEIGQQV
OXA-23-like_QEE57736.1_A.b.  KWKGEKRSFTAWEKDMTLGEAMKLSAVPVYQELARRIGLDLMQKEVKRIGFGNAEIGQQV
OXA-23-like_QEE57740.1_A.b.  KWKGEKRSFTAWEKDMTLGEAMKLSAVPVYQELARRIGLDLMQKEVKRIGFGNAEIGQQV
OXA-23-like_QEE57744.1_A.b.  KWKGEKRSFTAWEKDMTLGEAMKLSAVPVYQELARRIGLDLMQKEVKRIGFGNAEIGQQV
OXA-23-like_QEY29291.1_A.b.  KWKGEKRSFTAWEKDMTLGEAMKLSAVPVYQELARRIGLDLMQKEVKRIGFGNAEIGQQV
OXA-23-like_QEY29320.1_A.b.  KWKGEKRSFTAWEKDMTLGEAMKLSAVPVYQELARRIGLDLMQKEVKRIGFGNAEIGQQV
OXA-23-like_QEY29496.1_A.b.  KWKGEKRSFTAWEKDMTLGEAMKLSAVPVYQELARRIGLDLMQKEVKRIGFGNAEIGQQV
OXA-23-like_QIX29121.1_A.b.  KWKGEKRSFTAWEKDMTLGEAMKLSAVPVYQELARRIGLDLMQKEVKRIGFGNAEIGQQV
OXA-23-like_QIX32951.1_A.b.  KWKGEKRSFTAWEKDMTLGEAMKLSAVPVYQELARRIGLDLMQKEVKRIGFGNAEIGQQV
OXA-23-like_QIX36912.1_A.b.  KWKGEKRSFTAWEKDMTLGEAMKLSAVPVYQELARRIGLDLMQKEVKRIGFGNAEIGQQV
OXA-23-like_QIX40711.1_A.b.  KWKGEKRSFTAWEKDMTLGEAMKLSAVPVYQELARRIGLDLMQKEVKRIGFGNAEIGQQV
OXA-23-like_QIX44509.1_A.b.  KWKGEKRSFTAWEKDMTLGEAMKLSAVPVYQELARRIGLDLMQKEVKRIGFGNAEIGQQV
OXA-23-like_QJG69304.1_A.b.  KWKGEKRSFTAWEKDMTLGEAMKLSAVPVYQELARRIGLDLMQKEVKRIGFGNAEIGQQV
OXA-23-like_QJG70467.1_A.b.  KWKGEKRSFTAWEKDMTLGEAMKLSAVPVYQELARRIGLDLMQKEVKRIGFGNAEIGQQV
OXA-23-like_QJG70485.1_A.b.  KWKGEKRSFTAWEKDMTLGEAMKLSAVPVYQELARRIGLDLMQKEVKRIGFGNAEIGQQV
OXA-23-like_QJG71265.1_A.b.  KWKGEKRSFTAWEKDMTLGEAMKLSAVPVYQELARRIGLDLMQKEVKRIGFGNAEIGQQV
OXA-23-like_QJG74213.1_A.b.  KWKGEKRSFTAWEKDMTLGEAMKLSAVPVYQELARRIGLDLMQKEVKRIGFGNAEIGQQV
OXA-23-like_QJG81833.1_A.b.  KWKGEKRSFTAWEKDMTLGEAMKLSAVPVYQELARRIGLDLMQKEVKRIGFGNAEIGQQV
OXA-23-like_QJG84680.1_A.b.  KWKGEKRSFTAWEKDMTLGEAMKLSAVPVYQELARRIGLDLMQKEVKRIGFGNAEIGQQV
OXA-23-like_QJG89564.1_A.b.  KWKGEKRSFTAWEKDMTLGEAMKLSAVPVYQELARRIGLDLMQKEVKRIGFGNAEIGQQV
OXA-23-like_QJG89584.1_A.b.  KWKGEKRSFTAWEKDMTLGEAMKLSAVPVYQELARRIGLDLMQKEVKRIGFGNAEIGQQV
OXA-23-like_QJG90447.1_A.b.  KWKGEKRSFTAWEKDMTLGEAMKLSAVPVYQELARRIGLDLMQKEVKRIGFGNAEIGQQV
OXA-23-like_QJG93290.1_A.b.  KWKGEKRSFTAWEKDMTLGEAMKLSAVPVYQELARRIGLDLMQKEVKRIGFGNAEIGQQV
OXA-23-like_ADX93021.1_A.b.  KWKGEKRSFTAWEKDMTLGEAMKLSAVPVYQELARRIGLDLMQKEVKRIGFGNAEIGQQV
OXA-23-like_AXG83856.1_A.b.  KWKGEKRLFTAWEKDMTLGEAMKLSAVPVYQELARRIGLDLMQKEVKRIGFGNAEIGQQV
OXA-23-like_AML68547.1_A.b.  KWKGEKRSFTAWEKDMTLGEAMKLSAVPVYQELARRIGLDLMQKEVKRIGFGNAEIGQQV
OXA-23-like_ARG03241.1_A.b.  KWKGEKRSFTAWEKDMTLGEAMKLSAVPVYQELARRIGLDLMQKEVKRIGFGNAEIGQQV
OXA-23-like_AOX84133.1_A.b.  KWKGEKRSFTAWEKDMTLGEAMKLSAVPVYQELARRIGLDLMQKEVKRIGFGNAEIGQQV
OXA-23-like_AOX82595.1_A.b.  KWKGEKRSFTAWEKDMTLGEAMKLSAVPVYQELARRIGLDLMQKEVKRIGFGNAEIGQQV
OXA-23-like_AOX75920.1_A.b.  KWKGEKRSFTAWEKDMTLGEAMKLSAVPVYQELARRIGLDLMQKEVKRIGFGNAEIGQQV
OXA-23-like_AOX75888.1_A.b.  KWKGEKRSFTAWEKDMTLGEAMKLSAVPVYQELARRIGLDLMQKEVKRIGFGNAEIGQQV
```

OXA-23-like subfamily

```
OXA-23-like_AOX72443.1_A.b.  KWKGEKRSFTAWEKDMTLGEAMKLSAVPVYQELARRIGLDLMQKEVKRIGFGNAEIGQQV
OXA-23-like_AOX68171.1_A.b.  KWKGEKRSFTAWEKDMTLGEAMKLSAVPVYQELARRIGLDLMQKEVKRIGFGNAEIGQQV
OXA-23-like_AMC14310.1_A.b.  KWKGEKRSFTAWEKDMTLGEAMKLSAVPVYQELARRIGLDLMQKEVKRIGFGNAEIGQQV
OXA-23-like_AMC14301.1_A.b.  KWKGEKRSFTAWEKDMTLGEAMKLSAVPVYQELARRIGLDLMQKEVKRIGFGNAEIGQQV
OXA-23-like_ALY00722.1_A.b.  KWKGEKRSFTAWEKDMTLGEAMKLSAVPVYQELARRIGLDLMQKEVKRIGFGNAEIGQQV
OXA-23-like_ALX98871.1_A.b.  KWKGEKRSFTAWEKDMTLGEAMKLSAVPVYQELARRIGLDLMQKEVKRIGFGNAEIGQQV
OXA-23-like_AKA30333.1_A.b.  KWKGEKRSFTAWEKDMTLGEAMKLSAVPVYQELARRIGLDLMQKEVKRIGFGNAEIGQQV
OXA-23-like_AGQ10499.1_A.b.  KWKGEKRSFTAWEKDMTLGEAMKLSAVPVYQELARRIGLDLMQKEVKRIGFGNAEIGQQV
OXA-23-like_AGQ05635.1_A.b.  KWKGEKRSFTAWEKDMTLGEAMKLSAVPVYQELARRIGLDLMQKEVKRIGFGNAEIGQQV
OXA-23-like_ACJ39972.1_A.b.  KWKGEKRSFTAWEKDMTLGEAMKLSAVPVYQELARRIGLDLMQKEVKRIGFGNAEIGQQV
OXA-23-like_QKK76748.1_A.b.  KWKGEKRSFTAWEKDMTLGEAMKLSAVPVYQELARRIGLDLMQKEVKRIGFGNAEIGQQV
OXA-23-like_QKK76707.1_A.b.  KWKGEKRSFTAWEKDMTLGEAMKLSAVPVYQELARRIGLDLMQKEVKRIGFGNAEIGQQV
OXA-23-like_QKK76393.1_A.b.  KWKGEKRSFTAWEKDMTLGEAMKLSAVPVYQELARRIGLDLMQKEVKRIGFGNAEIGQQV
OXA-23-like_QFX72557.1_A.b.  KWKGEKRSFTAWEKDMTLGEAMKLSAVPVYQELARRIGLDLMQKEVKRIGFGNAEIGQQV
OXA-23-like_QFZ56693.1_A.b.  KWKGEKRSFTAWEKDMTLGEAMKLSAVPVYQELARRIGLDLMQKEVKRIGFGNAEIGQQV
OXA-23-like_AYC00513.1_A.b.  KWKGEKRSFTAWEKDMTLGEAMKLSAVPVYQELARRIGLDLMQKEVKRIGFGNAEIGQQV
OXA-23-like_AYC00505.1_A.b.  KWKGEKRSFTAWEKDMTLGEAMKLSAVPVYQELARRIGLDLMQKEVKRIGFGNAEIGQQV
OXA-23-like_ARG39357.1_A.b.  KWKGEKRSFTAWEKDMTLGEAMKLSAVPVYQELARRIGLDLMQKEVKRIGFGNAEIGQQV
OXA-23-like_ARG23739.1_A.b.  KWKGEKRSFTAWEKDMTLGEAMKLSAVPVYQELARRIGLDLMQKEVKRIGFGNAEIGQQV
OXA-23-like_ARG23713.1_A.b.  KWKGEKRSFTAWEKDMTLGEAMKLSAVPVYQELARRIGLDLMQKEVKRIGFGNAEIGQQV
OXA-23-like_ARG03186.1_A.b.  KWKGEKRSFTAWEKDMTLGEAMKLSAVPVYQELARRIGLDLMQKEVKRIGFGNAEIGQQV
OXA-23-like_AEP06450.2_A.b.  KWKGEKRSFTAWEKDMTLGEAMKLSAVPVYQELARRIGLDLMQKEVKRIGFGNAEIGQQV
OXA-23-like_AML75286.1_A.b.  KWKGEKRSFTAWEKDMTLGEAMKLSAVPVYQELARRIGLDLMQKEVKRIGFGNAEIGQQV
OXA-23-like_AML75277.1_A.b.  KWKGEKRSFTAWEKDMTLGEAMKLSAVPVYQELARRIGLDLMQKEVKRIGFGNAEIGQQV
OXA-23-like_AML71526.1_A.b.  KWKGEKRSFTAWEKDMTLGEAMKLSAVPVYQELARRIGLDLMQKEVKRIGFGNAEIGQQV
OXA-23-like_AML69469.1_A.b.  KWKGEKRSFTAWEKDMTLGEAMKLSAVPVYQELARRIGLDLMQKEVKRIGFGNAEIGQQV
OXA-23-like_AML66436.1_A.b.  KWKGEKRSFTAWEKDMTLGEAMKLSAVPVYQELARRIGLDLMQKEVKRIGFGNAEIGQQV
OXA-23-like_AML65160.1_A.b.  KWKGEKRSFTAWEKDMTLGEAMKLSAVPVYQELARRIGLDLMQKEVKRIGFGNAEIGQQV
OXA-23-like_AML65128.1_A.b.  KWKGEKRSFTAWEKDMTLGEAMKLSAVPVYQELARRIGLDLMQKEVKRIGFGNAEIGQQV
OXA-23-like_AMN00602.1_A.b.  KWKGEKRSFTAWEKDMTLGEAMKLSAVPVYQELARRIGLDLMQKEVKRIGFGNAEIGQQV
OXA-23-like_AMN00587.1_A.b.  KWKGEKRSFTAWEKDMTLGEAMKLSAVPVYQELARRIGLDLMQKEVKRIGFGNAEIGQQV
OXA-23-like_AMN00578.1_A.b.  KWKGEKRSFTAWEKDMTLGEAMKLSAVPVYQELARRIGLDLMQKEVKRIGFGNAEIGQQV
OXA-23-like_AMN00569.1_A.b.  KWKGEKRSFTAWEKDMTLGEAMKLSAVPVYQELARRIGLDLMQKEVKRIGFGNAEIGQQV
OXA-23-like_QJF37605.1_A.b.  KWKGEKRSFTAWEKDMTLGEAMKLSAVPVYQELARRIGLDLMQKEVKRIGFGNAEIGQQV
OXA-23-like_QJF33717.1_A.b.  KWKGEKRSFTAWEKDMTLGEAMKLSAVPVYQELARRIGLDLMQKEVKRIGFGNAEIGQQV
OXA-23-like_QJH05102.1_A.b.  KWKGEKRSFTAWEKDMTLGEAMKLSAVPVYQELARRIGLDLMQKEVKRIGFGNAEIGQQV
OXA-23-like_QJH23846.1_A.b.  KWKGEKRSFTAWEKDMTLGEAMKLSAVPVYQELARRIGLDLMQKEVKRIGFGNAEIGQQV
OXA-23-like_QCH35083.1_A.b.  KWKGEKRSFTAWEKDMTLGEAMKLSAVPVYQELARRIGLDLMQKEVKRIGFGNAEIGQQV
OXA-23-like_QAT07029.1_A.b.  KWKGEKRSFTAWEKDMTLGEAMKLSAVPVYQELARRIGLDLMQKEVKRIGFGNAEIGQQV
OXA-23-like_QAS99734.1_A.b.  KWKGEKRSFTAWEKDMTLGEAMKLSAVPVYQELARRIGLDLMQKEVKRIGFGNAEIGQQV
OXA-23-like_AVE56824.1_A.b.  KWKGEKRSFTAWEKDMTLGEAMKLSAVPVYQELARRIGLDLMQKEVKRIGFGNAEIGQQV
OXA-23-like_AVE44374.1_A.b.  KWKGEKRSFTAWEKDMTLGEAMKLSAVPVYQELARRIGLDLMQKEVKRIGFGNAEIGQQV
OXA-23-like_ATR89604.1_A.b.  KWKGEKRSFTAWEKDMTLGEAMKLSAVPVYQELARRIGLDLMQKEVKRIGFGNAEIGQQV
OXA-23-like_ARG29802.1_A.b.  KWKGEKRSFTAWEKDMTLGEAMKLSAVPVYQELARRIGLDLMQKEVKRIGFGNAEIGQQV
OXA-23-like_APF45746.1_A.b.  KWKGEKRSFTAWEKDMTLGEAMKLSAVPVYQELARRIGLDLMQKEVKRIGFGNAEIGQQV
OXA-23-like_APQ95067.1_A.b.  KWKGEKRSFTAWEKDMTLGEAMKLSAVPVYQELARRIGLDLMQKEVKRIGFGNAEIGQQV
OXA-23-like_APQ87413.1_A.b.  KWKGEKRSFTAWEKDMTLGEAMKLSAVPVYQELARRIGLDLMQKEVKRIGFGNAEIGQQV
OXA-23-like_ATR88802.1_A.b.  KWKGEKRSFTAWEKDMTLGEAMKLSAVPVYQELARRIGLDLMQKEVKRIGFGNAEIGQQV
OXA-23-like_ATR88943.1_A.b.  KWKGEKRSFTAWEKDMTLGEAMKLSAVPVYQELARRIGLDLMQKEVKRIGFGNAEIGQQV
OXA-23-like_SBS23848.1_A.b.  KWKGEKRSFTAWEKDMTLGEAMKVSAVPVYQELARRIGLDLMQKEVKRIGFGNAEIGQQV
OXA-23-like_AOP62462.1_A.b.  KWKGEKRSFTAWEKDMTLGEAMKLSAVPVYQELARRIGLDLMQKEVKRIGFGNAEIGQQV
4JF4_Beta-lactamase          KWKGEKRSFTAWEKDMTLGEAMKLSAVPVYQELARRIGLDLMQKEVKRIGFGNAEIGQQV
OXA-23-like_QCP18237.1_A.b.  KWKGEKRSFTAWEKDMTLGEAMKLSAVPVYQELARRIGLDLMQKEVKRIGFGNAEIGQQV
OXA-23-like_QRN19109.1_A.b.  KWKGEKRSFTAWEKDMTLGEAMKLSAVPVYQELARRIGLDLMQKEVKRIGFGNAEIGQQV
                        acc
```

OXA-23-like subfamily

```
                                      α6              α7          β3      β4
OXA-23-like_QQE03160.1_A.b.     QQQQQQQQQQQQ      QQQQQQQQQ   ──────►TT──────────►T
                         170       180       190       200       210       220
OXA-23-like_QQE03160.1_A.b. DNFWLVGPLKVTPIQEVEFVSQLAHTQLPFSEKVQANVKNMLLLEESNGYKIFGKTGWAM
OXA-23-like_QQE03411.1_A.b. DNFWLVGPLKVTPIQEVEFVSQLAHTQLPFSEKVQANVKNMLLLEESNGYKIFGKTGWAM
OXA-23-like_QQD99609.1_A.b. DNFWLVGPLKVTPIQEVEFVSQLAHTQLPFSEKVQANVKNMLLLEESNGYKIFGKTGWAM
OXA-23-like_QQD99362.1_A.b. DNFWLVGPLKVTPIQEVEFVSQLAHTQLPFSEKVQANVKNMLLLEESNGYKIFGKTGWAM
OXA-23-like_QQD95815.1_A.b. DNFWLVGPLKVTPIQEVEFVSQLAHTQLPFSEKVQANVKNMLLLEESNGYKIFGKTGWAM
OXA-23-like_QQD95566.1_A.b. DNFWLVGPLKVTPIQEVEFVSQLAHTQLPFSEKVQANVKNMLLLEESNGYKIFGKTGWAM
OXA-23-like_QQD93073.1_A.b. DNFWLVGPLKVTPIQEVEFVSQLAHTQLPFSEKVQANVKNMLLLEESNGYKIFGKTGWAM
OXA-23-like_QQD92824.1_A.b. DNFWLVGPLKVTPIQEVEFVSQLAHTQLPFSEKVQANVKNMLLLEESNGYKIFGKTGWAM
OXA-23-like_QQB67579.1_A.b. DNFWLVGPLKVTPIQEVEFVSQLAHTQLPFSEKVQANVKNMLLLEESNGYKIFGKTGWAM
OXA-23-like_QQB65684.1_A.b. DNFWLVGPLKVTPIQEVEFVSQLAHTQLPFSEKVQANVKNMLLLEESNGYKIFGKTGWAM
OXA-23-like_QPO03745.1_A.b. DNFWLVGPLKVTPIQEVEFVSQLAHTQLPFSEKVQANVKNMLLLEESNGYKIFGKTGWAM
OXA-23-like_QPO00239.1_A.b. DNFWLVGPLKVTPIQEVEFVSQLAHTQLPFSEKVQANVKNMLLLEESNGYKIFGKTGWAM
OXA-23-like_QLG80230.1_A.b. DNFWLVGPLKVTPIQEVEFVSQLAHTQLPFSEKVQANVKNMLLLEESNGYKIFGKTGWAM
OXA-23-like_QLA71954.1_A.b. DNFWLVGPLKVTPIQEVEFVSQLAHTQLPFSEKVQANVKNMLLLEESNGYKIFGKTGWAM
OXA-23-like_QJP32450.1_A.b. DNFWLVGPLKVTPIQEVEFVSQLAHTQLPFSEKVQANVKNMLLLEESNGYKIFGKTGWAM
OXA-23-like_QJH23301.1_A.b. DNFWLVGPLKVTPIQEVEFVSQLAHTQLPFSEKVQANVKNMLLLEESNGYKIFGKTGWAM
OXA-23-like_QJH19267.1_A.b. DNFWLVGPLKVTPIQEVEFVSQLAHTQLPFSEKVQANVKNMLLLEESNGYKIFGKTGWAM
OXA-23-like_QJH16482.1_A.b. DNFWLVGPLKVTPIQEVEFVSQLAHTQLPFSEKVQANVKNMLLLEESNGYKIFGKTGWAM
OXA-23-like_QJH15745.1_A.b. DNFWLVGPLKVTPIQEVEFVSQLAHTQLPFSEKVQANVKNMLLLEESNGYKIFGKTGWAM
OXA-23-like_QJH15717.1_A.b. DNFWLVGPLKVTPIQEVEFVSQLAHTQLPFSEKVQANVKNMLLLEESNGYKIFGKTGWAM
OXA-23-like_QJH08361.1_A.b. DNFWLVGPLKVTPIQEVEFVSQLAHTQLPFSEKVQANVKNMLLLEESNGYKIFGKTGWAM
OXA-23-like_QJH12331.1_A.b. DNFWLVGPLKVTPIQEVEFVSQLAHTQLPFSEKVQANVKNMLLLEESNGYKIFGKTGWAM
OXA-23-like_QJH05456.1_A.b. DNFWLVGPLKVTPIQEVEFVSQLAHTQLPFSEKVQANVKNMLLLEESNGYKIFGKTGWAM
OXA-23-like_QJH00715.1_A.b. DNFWLVGPLKVTPIQEVEFVSQLAHTQLPFSEKVQANVKNMLLLEESNGYKIFGKTGWAM
OXA-23-like_QJG95773.1_A.b. DNFWLVGPLKVTPIQEVEFVSQLAHTQLPFSEKVQANVKNMLLLEESNGYKIFGKTGWAM
OXA-23-like_QJG96932.1_A.b. DNFWLVGPLKVTPIQEVEFVSQLAHTQLPFSEKVQANVKNMLLLEESNGYKIFGKTGWAM
OXA-23-like_QJG96957.1_A.b. DNFWLVGPLKVTPIQEVEFVSQLAHTQLPFSEKVQANVKNMLLLEESNGYKIFGKTGWAM
OXA-23-like_QJH00689.1_A.b. DNFWLVGPLKVTPIQEVEFVSQLAHTQLPFSEKVQANVKNMLLLEESNGYKIFGKTGWAM
OXA-23-like_AOX88011.1_A.b. DNFWLVGPLKVTPIQEVEFVSQLAHTQLPFSEKVQANVKNMLLLEESNGYKIFGKTGWAM
OXA-23-like_AOX91849.1_A.b. DNFWLVGPLKVTPIQEVEFVSQLAHTQLPFSEKVQANVKNMLLLEESNGYKIFGKTGWAM
OXA-23-like_AOX95708.1_A.b. DNFWLVGPLKVTPIQEVEFVSQLAHTQLPFSEKVQANVKNMLLLEESNGYKIFGKTGWAM
OXA-23-like_APM50633.1_A.b. DNFWLVGPLKVTPIQEVEFVSQLAHTQLPFSEKVQANVKNMLLLEESNGYKIFGKTGWAM
OXA-23-like_APM50651.1_A.b. DNFWLVGPLKVTPIQEVEFVSQLAHTQLPFSEKVQANVKNMLLLEESNGYKIFGKTGWAM
OXA-23-like_AQU56756.1_A.b. DNFWLVGPLKVTPIQEVEFVSQLAHTQLPFSEKVQANVKNMLLLEESNGYKIFGKTGWAM
OXA-23-like_AQU56788.1_A.b. DNFWLVGPLKVTPIQEVEFVSQLAHTQLPFSEKVQANVKNMLLLEESNGYKIFGKTGWAM
OXA-23-like_ARF92486.1_A.b. DNFWLVGPLKVTPIQEVEFVSQLAHTQLPFSEKVQANVKNMLLLEESNGYKIFGKTGWAM
OXA-23-like_ARF92497.1_A.b. DNFWLVGPLKVTPIQEVEFVSQLAHTQLPFSEKVQANVKNMLLLEESNGYKIFGKTGWAM
OXA-23-like_ARF95290.1_A.b. DNFWLVGPLKVTPIQEVEFVSQLAHTQLPFSEKVQANVKNMLLLEESNGYKIFGKTGWAM
OXA-23-like_ARF96125.1_A.b. DNFWLVGPLKVTPIQEVEFVSQLAHTQLPFSEKVQANVKNMLLLEESNGYKIFGKTGWAM
OXA-23-like_ARG05487.1_A.b. DNFWLVGPLKVTPIQEVEFVSQLAHTQLPFSEKVQANVKNMLLLEESNGYKIFGKTGWAM
OXA-23-like_ARG10150.1_A.b. DNFWLVGPLKVTPIQEVEFVSQLAHTQLPFSEKVQANVKNMLLLEESNGYKIFGKTGWAM
OXA-23-like_ARG12999.1_A.b. DNFWLVGPLKVTPIQEVEFVSQLAHTQLPFSEKVQANVKNMLLLEESNGYKIFGKTGWAM
OXA-23-like_ARG19192.1_A.b. DNFWLVGPLKVTPIQEVEFVSQLAHTQLPFSEKVQANVKNMLLLEESNGYKIFGKTGWAM
OXA-23-like_ARG19219.1_A.b. DNFWLVGPLKVTPIQEVEFVSQLAHTQLPFSEKVQANVKNMLLLEESNGYKIFGKTGWAM
OXA-23-like_ARG28873.1_A.b. DNFWLVGPLKVTPIQEVEFVSQLAHTQLPFSEKVQANVKNMLLLEESNGYKIFGKTGWAM
OXA-23-like_ARG28898.1_A.b. DNFWLVGPLKVTPIQEVEFVSQLAHTQLPFSEKVQANVKNMLLLEESNGYKIFGKTGWAM
OXA-23-like_ARG30155.1_A.b. DNFWLVGPLKVTPIQEVEFVSQLAHTQLPFSEKVQANVKNMLLLEESNGYKIFGKTGWAM
OXA-23-like_ARG35250.1_A.b. DNFWLVGPLKVTPIQEVEFVSQLAHTQLPFSEKVQANVKNMLLLEESNGYKIFGKTGWAM
OXA-23-like_ASF49900.1_A.b. DNFWLVGPLKVTPIQEVEFVSQLAHTQLPFSEKVQANVKNMLLLEESNGYKIFGKTGWAM
OXA-23-like_AUG13479.1_A.b. DNFWLVGPLKVTPIQEVEFVSQLAHTQLPFSEKVQANVKNMLLLEESNGYKIFGKTGWAM
OXA-23-like_AVN25815.1_A.b. DNFWLVGPLKVTPIQEVEFVSQLAHTQLPFSEKVQANVKNMLLLEESNGYKIFGKTGWAM
OXA-23-like_AVN25876.1_A.b. DNFWLVGPLKVTPIQEVEFVSQLAHTQLPFSEKVQANVKNMLLLEESNGYKIFGKTGWAM
OXA-23-like_AVN25910.1_A.b. DNFWLVGPLKVTPIQEVEFVSQLAHTQLPFSEKVQANVKNMLLLEESNGYKIFGKTGWAM
OXA-23-like_AWW79127.1_A.b. DNFWLVGPLKVTPIQEVEFVSQLAHTQLPFSEKVQANVKNMLLLEESNGYKIFGKTGWAM
OXA-23-like_AWW83877.1_A.b. DNFWLVGPLKVTPIQEVEFVSQLAHTQLPFSEKVQANVKNMLLLEESNGYKIFGKTGWAM
OXA-23-like_AWW86703.1_A.b. DNFWLVGPLKVTPIQEVEFVSQLAHTQLPFSEKVQANVKNMLLLEESNGYKIFGKTGWAM
OXA-23-like_AYY88859.1_A.b. DNFWLVGPLKVTPIQEVEFVSQLAHTQLPFSEKVQANVKNMLLLEESNGYKIFGKTGWAM
OXA-23-like_BAP68327.1_A.b. DNFWLVGPLKVTPIQEVEFVSQLAHTQLPFSEKVQANVKNMLLLEESNGYKIFGKTGWAM
OXA-23-like_QAS95332.1_A.b. DNFWLVGPLKVTPIQEVEFVSQLAHTQLPFSEKVQANVKNMLLLEESNGYKIFGKTGWAM
OXA-23-like_QAT02983.1_A.b. DNFWLVGPLKVTPIQEVEFVSQLAHTQLPFSEKVQANVKNMLLLEESNGYKIFGKTGWAM
OXA-23-like_QBY15339.1_A.b. DNFWLVGPLKVTPIQEVEFVSQLAHTQLPFSEKVQANVKNMLLLEESNGYKIFGKTGWAM
OXA-23-like_QCH32733.1_A.b. DNFWLVGPLKVTPIQEVEFVSQLAHTQLPFSEKVQANVKNMLLLEESNGYKIFGKTGWAM
OXA-23-like_QCP17255.1_A.b. DNFWLVGPLKVTPIQEVEFVSQLAHTQLPFSEKVQANVKNMLLLEESNGYKIFGKTGWAM
OXA-23-like_QCP24705.1_A.b. DNFWLVGPLKVTPIQEVEFVSQLAHTQLPFSEKVQANVKNMLLLEESNGYKIFGKTGWAM
OXA-23-like_QCP27215.1_A.b. DNFWLVGPLKVTPIQEVEFVSQLAHTQLPFSEKVQANVKNMLLLEESNGYKIFGKTGWAM
OXA-23-like_QCP29558.1_A.b. DNFWLVGPLKVTPIQEVEFVSQLAHTQLPFSEKVQANVKNMLLLEESNGYKIFGKTGWAM
OXA-23-like_QCP40778.1_A.b. DNFWLVGPLKVTPIQEVEFVSQLAHTQLPFSEKVQANVKNMLLLEESNGYKIFGKTGWAM
OXA-23-like_QCP43779.1_A.b. DNFWLVGPLKVTPIQEVEFVSQLAHTQLPFSEKVQANVKNMLLLEESNGYKIFGKTGWAM
OXA-23-like_QCP44095.1_A.b. DNFWLVGPLKVTPIQEVEFVSQLAHTQLPFSEKVQANVKNMLLLEESNGYKIFGKTGWAM
OXA-23-like_QCP47140.1_A.b. DNFWLVGPLKVTPIQEVEFVSQLAHTQLPFSEKVQANVKNMLLLEESNGYKIFGKTGWAM
OXA-23-like_QCR88532.1_A.b. DNFWLVGPLKVTPIQEVEFVSQLAHTQLPFSEKVQANVKNMLLLEESNGYKIFGKTGWAM
OXA-23-like_QDR92294.1_A.b. DNFWLVGPLKVTPIQEVEFVSQLAHTQLPFSEKVQANVKNMLLLEESNGYKIFGKTGWAM
OXA-23-like_QDR95367.1_A.b. DNFWLVGPLKVTPIQEVEFVSQLAHTQLPFSEKVQANVKNMLLLEESNGYKIFGKTGWAM
OXA-23-like_QEE57736.1_A.b. DNFWLVGPLKVTPIQEVEFVSQLAHTQLPFSEKVQANVKNMLLLEESNGYKIFGKTGWAM
OXA-23-like_QEE57740.1_A.b. DNFWLVGPLKVTPIQEVEFVSQLAHTQLPFSEKVQANVKNMLLLEESNGYKIFGKTGWAM
OXA-23-like_QEE57744.1_A.b. DNFWLVGPLKVTPIQEVEFVSQLAHTQLPFSEKVQANVKNMLLLEESNGYKIFGKTGWAM
OXA-23-like_QEY29291.1_A.b. DNFWLVGPLKVTPIQEVEFVSQLAHTQLPFSEKVQANVKNMLLLEESNGYKIFGKTGWAM
OXA-23-like_QEY29320.1_A.b. DNFWLVGPLKVTPIQEVEFVSQLAHTQLPFSEKVQANVKNMLLLEESNGYKIFGKTGWAM
OXA-23-like_QEY29496.1_A.b. DNFWLVGPLKVTPIQEVEFVSQLAHTQLPFSEKVQANVKNMLLLEESNGYKIFGKTGWAM
OXA-23-like_QIX29121.1_A.b. DNFWLVGPLKVTPIQEVEFVSQLAHTQLPFSEKVQANVKNMLLLEESNGYKIFGKTGWAM
OXA-23-like_QIX32951.1_A.b. DNFWLVGPLKVTPIQEVEFVSQLAHTQLPFSEKVQANVKNMLLLEESNGYKIFGKTGWAM
OXA-23-like_QIX36912.1_A.b. DNFWLVGPLKVTPIQEVEFVSQLAHTQLPFSEKVQANVKNMLLLEESNGYKIFGKTGWAM
OXA-23-like_QIX40711.1_A.b. DNFWLVGPLKVTPIQEVEFVSQLAHTQLPFSEKVQANVKNMLLLEESNGYKIFGKTGWAM
OXA-23-like_QIX44509.1_A.b. DNFWLVGPLKVTPIQEVEFVSQLAHTQLPFSEKVQANVKNMLLLEESNGYKIFGKTGWAM
OXA-23-like_QJG69304.1_A.b. DNFWLVGPLKVTPIQEVEFVSQLAHTQLPFSEKVQANVKNMLLLEESNGYKIFGKTGWAM
OXA-23-like_QJG70467.1_A.b. DNFWLVGPLKVTPIQEVEFVSQLAHTQLPFSEKVQANVKNMLLLEESNGYKIFGKTGWAM
OXA-23-like_QJG70485.1_A.b. DNFWLVGPLKVTPIQEVEFVSQLAHTQLPFSEKVQANVKNMLLLEESNGYKIFGKTGWAM
OXA-23-like_QJG71265.1_A.b. DNFWLVGPLKVTPIQEVEFVSQLAHTQLPFSEKVQANVKNMLLLEESNGYKIFGKTGWAM
OXA-23-like_QJG74213.1_A.b. DNFWLVGPLKVTPIQEVEFVSQLAHTQLPFSEKVQANVKNMLLLEESNGYKIFGKTGWAM
OXA-23-like_QJG81833.1_A.b. DNFWLVGPLKVTPIQEVEFVSQLAHTQLPFSEKVQANVKNMLLLEESNGYKIFGKTGWAM
OXA-23-like_QJG84680.1_A.b. DNFWLVGPLKVTPIQEVEFVSQLAHTQLPFSEKVQANVKNMLLLEESNGYKIFGKTGWAM
OXA-23-like_QJG89564.1_A.b. DNFWLVGPLKVTPIQEVEFVSQLAHTQLPFSEKVQANVKNMLLLEESNGYKIFGKTGWAM
OXA-23-like_QJG89584.1_A.b. DNFWLVGPLKVTPIQEVEFVSQLAHTQLPFSEKVQANVKNMLLLEESNGYKIFGKTGWAM
OXA-23-like_QJG90447.1_A.b. DNFWLVGPLKVTPIQEVEFVSQLAHTQLPFSEKVQANVKNMLLLEESNGYKIFGKTGWAM
OXA-23-like_QJG93290.1_A.b. DNFWLVGPLKVTPIQEVEFVSQLAHTQLPFSEKVQANVKNMLLLEESNGYKIFGKTGWAM
OXA-23-like_ADX93021.1_A.b. DNFWLVGPLKVTPIQEVEFVSQLAHTQLPFSEKVQANVKNMLLLEESNGYKIFGKTGWAM
OXA-23-like_AXG83856.1_A.b. DNFWLVGPLKVTPIQEVEFVSQLAHTQLPFSEKVQANVKNMLLLEESNGYKIFGKTGWAM
OXA-23-like_AML68547.1_A.b. DNFWLVGPLKVTPIQEVEFVSQLAHTQLPFSEKVQANVKNMLLLEESNGYKIFGKTGWAM
OXA-23-like_ARG03241.1_A.b. DNFWLVGPLKVTPIQEVEFVSQLAHTQLPFSEKVQANVKNMLLLEESNGYKIFGKTGWAM
OXA-23-like_AOX84133.1_A.b. DNFWLVGPLKVTPIQEVEFVSQLAHTQLPFSEKVQANVKNMLLLEESNGYKIFGKTGWAM
OXA-23-like_AOX82595.1_A.b. DNFWLVGPLKVTPIQEVEFVSQLAHTQLPFSEKVQANVKNMLLLEESNGYKIFGKTGWAM
OXA-23-like_AOX75920.1_A.b. DNFWLVGPLKVTPIQEVEFVSQLAHTQLPFSEKVQANVKNMLLLEESNGYKIFGKTGWAM
OXA-23-like_AOX75888.1_A.b. DNFWLVGPLKVTPIQEVEFVSQLAHTQLPFSEKVQANVKNMLLLEESNGYKIFGKTGWAM
```

OXA-23-like subfamily

```
OXA-23-like_AOX72443.1_A.b.  DNFWLVGPLKVTPIQEVEFVSQLAHTQLPFSEKVQANVKNMLLLEESNGYKIFGKTGWAM
OXA-23-like_AOX68171.1_A.b.  DNFWLVGPLKVTPIQEVEFVSQLAHTQLPFSEKVQANVKNMLLLEESNGYKIFGKTGWAM
OXA-23-like_AMC14310.1_A.b.  DNFWLVGPLKVTPIQEVEFVSQLAHTQLPFSEKVQANVKNMLLLEESNGYKIFGKTGWAM
OXA-23-like_AMC14301.1_A.b.  DNFWLVGPLKVTPIQEVEFVSQLAHTQLPFSEKVQANVKNMLLLEESNGYKIFGKTGWAM
OXA-23-like_ALY00722.1_A.b.  DNFWLVGPLKVTPIQEVEFVSQLAHTQLPFSEKVQANVKNMLLLEESNGYKIFGKTGWAM
OXA-23-like_ALX98871.1_A.b.  DNFWLVGPLKVTPIQEVEFVSQLAHTQLPFSEKVQANVKNMLLLEESNGYKIFGKTGWAM
OXA-23-like_AKA30333.1_A.b.  DNFWLVGPLKVTPIQEVEFVSQLAHTQLPFSEKVQANVKNMLLLEESNGYKIFGKTGWAM
OXA-23-like_AGQ10499.1_A.b.  DNFWLVGPLKVTPIQEVEFVSQLAHTQLPFSEKVQANVKNMLLLEESNGYKIFGKTGWAM
OXA-23-like_AGQ05635.1_A.b.  DNFWLVGPLKVTPIQEVEFVSQLAHTQLPFSEKVQANVKNMLLLEESNGYKIFGKTGWAM
OXA-23-like_ACJ39972.1_A.b.  DNFWLVGPLKVTPIQEVEFVSQLAHTQLPFSEKVQANVKNMLLLEESNGYKIFGKTGWAM
OXA-23-like_QKK76748.1_A.b.  DNFWLVGPLKVTPIQEVEFVSQLAHTQLPFSEKVQANVKNMLLLEESNGYKIFGKTGWAM
OXA-23-like_QKK76707.1_A.b.  DNFWLVGPLKVTPIQEVEFVSQLAHTQLPFSEKVQANVKNMLLLEESNGYKIFGKTGWAM
OXA-23-like_QKK76393.1_A.b.  DNFWLVGPLKVTPIQEVEFVSQLAHTQLPFSEKVQANVKNMLLLEESNGYKIFGKTGWAM
OXA-23-like_QFX72557.1_A.b.  DNFWLVGPLKVTPIQEVEFVSQLAHTQLPFSEKVQANVKNMLLLEESNGYKIFGKTGWAM
OXA-23-like_QFZ56693.1_A.b.  DNFWLVGPLKVTPIQEVEFVSQLAHTQLPFSEKVQANVKNMLLLEESNGYKIFGKTGWAM
OXA-23-like_AYC00513.1_A.b.  DNFWLVGPLKVTPIQEVEFVSQLAHTQLPFSEKVQANVKNMLLLEESNGYKIFGKTGWAM
OXA-23-like_AYC00505.1_A.b.  DNFWLVGPLKVTPIQEVEFVSQLAHTQLPFSEKVQANVKNMLLLEESNGYKIFGKTGWAM
OXA-23-like_ARG39357.1_A.b.  DNFWLVGPLKVTPIQEVEFVSQLAHTQLPFSEKVQANVKNMLLLEESNGYKIFGKTGWAM
OXA-23-like_ARG23739.1_A.b.  DNFWLVGPLKVTPIQEVEFVSQLAHTQLPFSEKVQANVKNMLLLEESNGYKIFGKTGWAM
OXA-23-like_ARG23713.1_A.b.  DNFWLVGPLKVTPIQEVEFVSQLAHTQLPFSEKVQANVKNMLLLEESNGYKIFGKTGWAM
OXA-23-like_ARG03186.1_A.b.  DNFWLVGPLKVTPIQEVEFVSQLAHTQLPFSEKVQANVKNMLLLEESNGYKIFGKTGWAM
OXA-23-like_AEP06450.2_A.b.  DNFWLVGPLKVTPIQEVEFVSQLAHTQLPFSEKVQANVKNMLLLEESNGYKIFGKTGWAM
OXA-23-like_AML75286.1_A.b.  DNFWLVGPLKVTPIQEVEFVSQLAHTQLPFSEKVQANVKNMLLLEESNGYKIFGKTGWAM
OXA-23-like_AML75277.1_A.b.  DNFWLVGPLKVTPIQEVEFVSQLAHTQLPFSEKVQANVKNMLLLEESNGYKIFGKTGWAM
OXA-23-like_AML71526.1_A.b.  DNFWLVGPLKVTPIQEVEFVSQLAHTQLPFSEKVQANVKNMLLLEESNGYKIFGKTGWAM
OXA-23-like_AML69469.1_A.b.  DNFWLVGPLKVTPIQEVEFVSQLAHTQLPFSEKVQANVKNMLLLEESNGYKIFGKTGWAM
OXA-23-like_AML66436.1_A.b.  DNFWLVGPLKVTPIQEVEFVSQLAHTQLPFSEKVQANVKNMLLLEESNGYKIFGKTGWAM
OXA-23-like_AML65160.1_A.b.  DNFWLVGPLKVTPIQEVEFVSQLAHTQLPFSEKVQANVKNMLLLEESNGYKIFGKTGWAM
OXA-23-like_AML65128.1_A.b.  DNFWLVGPLKVTPIQEVEFVSQLAHTQLPFSEKVQANVKNMLLLEESNGYKIFGKTGWAM
OXA-23-like_AMN00602.1_A.b.  DNFWLVGPLKVTPIQEVEFVSQLAHTQLPFSEKVQANVKNMLLLEESNGYKIFGKTGWAM
OXA-23-like_AMN00587.1_A.b.  DNFWLVGPLKVTPIQEVEFVSQLAHTQLPFSEKVQANVKNMLLLEESNGYKIFGKTGWAM
OXA-23-like_AMN00578.1_A.b.  DNFWLVGPLKVTPIQEVEFVSQLAHTQLPFSEKVQANVKNMLLLEESNGYKIFGKTGWAM
OXA-23-like_AMN00569.1_A.b.  DNFWLVGPLKVTPIQEVEFVSQLAHTQLPFSEKVQANVKNMLLLEESNGYKIFGKTGWAM
OXA-23-like_QJF37605.1_A.b.  DNFWLVGPLKVTPIQEVEFVSQLAHTQLPFSEKVQANVKNMLLLEESNGYKIFGKTGWAM
OXA-23-like_QJF33717.1_A.b.  DNFWLVGPLKVTPIQEVEFVSQLAHTQLPFSEKVQANVKNMLLLEESNGYKIFGKTGWAM
OXA-23-like_QJH05102.1_A.b.  DNFWLVGPLKVTPIQEVEFVSQLAHTQLPFSEKVQANVKNMLLLEESNGYKIFGKTGWAM
OXA-23-like_QJH23846.1_A.b.  DNFWLVGPLKVTPIQEVEFVSQLAHTQLPFSEKVQANVKNMLLLEESNGYKIFGKTGWAM
OXA-23-like_QCH35083.1_A.b.  DNFWLVGPLKVTPIQEVEFVSQLAHTQLPFSEKVQANVKNMLLLEESNGYKIFGKTGWAM
OXA-23-like_QAT07029.1_A.b.  DNFWLVGPLKVTPIQEVEFVSQLAHTQLPFSEKVQANVKNMLLLEESNGYKIFGKTGWAM
OXA-23-like_QAS99734.1_A.b.  DNFWLVGPLKVTPIQEVEFVSQLAHTQLPFSEKVQANVKNMLLLEESNGYKIFGKTGWAM
OXA-23-like_AVE56824.1_A.b.  DNFWLVGPLKVTPIQEVEFVSQLAHTQLPFSEKVQANVKNMLLLEESNGYKIFGKTGWAM
OXA-23-like_AVE44374.1_A.b.  DNFWLVGPLKVTPIQEVEFVSQLAHTQLPFSEKVQANVKNMLLLEESNGYKIFGKTGWAM
OXA-23-like_ATR89604.1_A.b.  DNFWLVGPLKVTPIQEVEFVSQLAHTQLPFSEKVQANVKNMLLLEESNGYKIFGKTGWAM
OXA-23-like_ARG29802.1_A.b.  DNFWLVGPLKVTPIQEVEFVSQLAHTQLPFSEKVQANVKNMLLLEESNGYKIFGKTGWAM
OXA-23-like_APF45746.1_A.b.  DNFWLVGPLKVTPIQEVEFVSQLAHTQLPFSEKVQANVKNMLLLEESNGYKIFGKTGWAM
OXA-23-like_APQ95067.1_A.b.  DNFWLVGPLKVTPIQEVEFVSQLAHTQLPFSEKVQANVKNMLLLEESNGYKIFGKTGWAM
OXA-23-like_APQ87413.1_A.b.  DNFWLVGPLKVTPIQEVEFVSQLAHTQLPFSEKVQANVKNMLLLEESNGYKIFGKTGWAM
OXA-23-like_ATR88802.1_A.b.  DNFWLVGPLKVTPIQEVEFVSQLAHTQLPFSEKVQANVKNMLLLEESNGYKIFGKTGWAM
OXA-23-like_ATR88943.1_A.b.  DNFWLVGPLKVTPIQEVEFVSQLAHTQLPFSEKVQANVKNMLLLEESNGYKIFGKTGWAM
OXA-23-like_SBS23848.1_A.b.  DNFWLVGPLKVTPIQEVEFVSQLAHTQLPFSEKVQANVKNMLLLEESNGYKIFGKTGWAM
OXA-23-like_AOP62462.1_A.b.  DNFWLVGPLKVTPIQEVEFVSQLAHTQLPFSEKVQANVKNMLLLEESNGYKIFGKTGWAM
4JF4_Beta-lactamase          DNFWLVGPLKVTPIQEVEFVSQLAHTQLPFSEKVQANVKNMLLLEESNGYKIFGKTGWAM
OXA-23-like_QCP18237.1_A.b.  DNFWLVGPLKVTPIQEVEFVSQLAHTQLPFSEKVQANVKNMLLLEESNGYKIFGKTGWAM
OXA-23-like_QRN19109.1_A.b.  DNFWLVGPLKVTPIQEVEFVSQLAHTQLPFSEKVQANVKNMLLLEESNGYKIFGKTGWAM
                        acc
```

OXA-23-like subfamily

```
                                      β5         β6          α8
OXA-23-like_QQE03160.1_A.b.  T  ────────→ TT ────────→ TT  ℓℓℓℓℓℓℓℓℓℓℓℓℓ
                                 ★230          240            250          260★         270

OXA-23-like_QQE03160.1_A.b.  DIKPQVGWLTGWVEQPDGKIVAFALNMEMRSEMPASIRNELLMKSLKQLNII
OXA-23-like_QQE03411.1_A.b.  DIKPQVGWLTGWVEQPDGKIVAFALNMEMRSEMPASIRNELLMKSLKQLNII
OXA-23-like_QQD99609.1_A.b.  DIKPQVGWLTGWVEQPDGKIVAFALNMEMRSEMPASIRNELLMKSLKQLNII
OXA-23-like_QQD99362.1_A.b.  DIKPQVGWLTGWVEQPDGKIVAFALNMEMRSEMPASIRNELLMKSLKQLNII
OXA-23-like_QQD95815.1_A.b.  DIKPQVGWLTGWVEQPDGKIVAFALNMEMRSEMPASIRNELLMKSLKQLNII
OXA-23-like_QQD95566.1_A.b.  DIKPQVGWLTGWVEQPDGKIVAFALNMEMRSEMPASIRNELLMKSLKQLNII
OXA-23-like_QQD93073.1_A.b.  DIKPQVGWLTGWVEQPDGKIVAFALNMEMRSEMPASIRNELLMKSLKQLNII
OXA-23-like_QQD92824.1_A.b.  DIKPQVGWLTGWVEQPDGKIVAFALNMEMRSEMPASIRNELLMKSLKQLNII
OXA-23-like_QQB67579.1_A.b.  DIKPQVGWLTGWVEQPDGKIVAFALNMEMRSEMPASIRNELLMKSLKQLNII
OXA-23-like_QQB65684.1_A.b.  DIKPQVGWLTGWVEQPDGKIVAFALNMEMRSEMPASIRNELLMKSLKQLNII
OXA-23-like_QPO03745.1_A.b.  DIKPQVGWLTGWVEQPDGKIVAFALNMEMRSEMPASIRNELLMKSLKQLNII
OXA-23-like_QPO00239.1_A.b.  DIKPQVGWLTGWVEQPDGKIVAFALNMEMRSEMPASIRNELLMKSLKQLNII
OXA-23-like_QLG80230.1_A.b.  DIKPQVGWLTGWVEQPDGKIVAFALNMEMRSEMPASIRNELLMKSLKQLNII
OXA-23-like_QLA71954.1_A.b.  DIKPQVGWLTGWVEQPDGKIVAFALNMEMRSEMPASIRNELLMKSLKQLNII
OXA-23-like_QJP32450.1_A.b.  DIKPQVGWLTGWVEQPDGKIVAFALNMEMRSEMPASIRNELLMKSLKQLNII
OXA-23-like_QJH23301.1_A.b.  DIKPQVGWLTGWVEQPDGKIVAFALNMEMRSEMPASIRNELLMKSLKQLNII
OXA-23-like_QJH19267.1_A.b.  DIKPQVGWLTGWVEQPDGKIVAFALNMEMRSEMPASIRNELLMKSLKQLNII
OXA-23-like_QJH16482.1_A.b.  DIKPQVGWLTGWVEQPDGKIVAFALNMEMRSEMPASIRNELLMKSLKQLNII
OXA-23-like_QJH15745.1_A.b.  DIKPQVGWLTGWVEQPDGKIVAFALNMEMRSEMPASIRNELLMKSLKQLNII
OXA-23-like_QJH15717.1_A.b.  DIKPQVGWLTGWVEQPDGKIVAFALNMEMRSEMPASIRNELLMKSLKQLNII
OXA-23-like_QJH08361.1_A.b.  DIKPQVGWLTGWVEQPDGKIVAFALNMEMRSEMPASIRNELLMKSLKQLNII
OXA-23-like_QJH12331.1_A.b.  DIKPQVGWLTGWVEQPDGKIVAFALNMEMRSEMPASIRNELLMKSLKQLNII
OXA-23-like_QJH05456.1_A.b.  DIKPQVGWLTGWVEQPDGKIVAFALNMEMRSEMPASIRNELLMKSLKQLNII
OXA-23-like_QJH00715.1_A.b.  DIKPQVGWLTGWVEQPDGKIVAFALNMEMRSEMPASIRNELLMKSLKQLNII
OXA-23-like_QJG95773.1_A.b.  DIKPQVGWLTGWVEQPDGKIVAFALNMEMRSEMPASIRNELLMKSLKQLNII
OXA-23-like_QJG96932.1_A.b.  DIKPQVGWLTGWVEQPDGKIVAFALNMEMRSEMPASIRNELLMKSLKQLNII
OXA-23-like_QJG96957.1_A.b.  DIKPQVGWLTGWVEQPDGKIVAFALNMEMRSEMPASIRNELLMKSLKQLNII
OXA-23-like_QJH00689.1_A.b.  DIKPQVGWLTGWVEQPDGKIVAFALNMEMRSEMPASIRNELLMKSLKQLNII
OXA-23-like_AOX88011.1_A.b.  DIKPQVGWLTGWVEQPDGKIVAFALNMEMRSEMPASIRNELLMKSLKQLNII
OXA-23-like_AOX91849.1_A.b.  DIKPQVGWLTGWVEQPDGKIVAFALNMEMRSEMPASIRNELLMKSLKQLNII
OXA-23-like_AOX95708.1_A.b.  DIKPQVGWLTGWVEQPDGKIVAFALNMEMRSEMPASIRNELLMKSLKQLNII
OXA-23-like_APM50633.1_A.b.  DIKPQVGWLTGWVEQPDGKIVAFALNMEMRSEMPASIRNELLMKSLKQLNII
OXA-23-like_APM50651.1_A.b.  DIKPQVGWLTGWVEQPDGKIVAFALNMEMRSEMPASIRNELLMKSLKQLNII
OXA-23-like_AQU56756.1_A.b.  DIKPQVGWLTGWVEQPDGKIVAFALNMEMRSEMPASIRNELLMKSLKQLNII
OXA-23-like_AQU56788.1_A.b.  DIKPQVGWLTGWVEQPDGKIVAFALNMEMRSEMPASIRNELLMKSLKQLNII
OXA-23-like_ARF92486.1_A.b.  DIKPQVGWLTGWVEQPDGKIVAFALNMEMRSEMPASIRNELLMKSLKQLNII
OXA-23-like_ARF92497.1_A.b.  DIKPQVGWLTGWVEQPDGKIVAFALNMEMRSEMPASIRNELLMKSLKQLNII
OXA-23-like_ARF95290.1_A.b.  DIKPQVGWLTGWVEQPDGKIVAFALNMEMRSEMPASIRNELLMKSLKQLNII
OXA-23-like_ARF96125.1_A.b.  DIKPQVGWLTGWVEQPDGKIVAFALNMEMRSEMPASIRNELLMKSLKQLNII
OXA-23-like_ARG05487.1_A.b.  DIKPQVGWLTGWVEQPDGKIVAFALNMEMRSEMPASIRNELLMKSLKQLNII
OXA-23-like_ARG10150.1_A.b.  DIKPQVGWLTGWVEQPDGKIVAFALNMEMRSEMPASIRNELLMKSLKQLNII
OXA-23-like_ARG12999.1_A.b.  DIKPQVGWLTGWVEQPDGKIVAFALNMEMRSEMPASIRNELLMKSLKQLNII
OXA-23-like_ARG19192.1_A.b.  DIKPQVGWLTGWVEQPDGKIVAFALNMEMRSEMPASIRNELLMKSLKQLNII
OXA-23-like_ARG19219.1_A.b.  DIKPQVGWLTGWVEQPDGKIVAFALNMEMRSEMPASIRNELLMKSLKQLNII
OXA-23-like_ARG28873.1_A.b.  DIKPQVGWLTGWVEQPDGKIVAFALNMEMRSEMPASIRNELLMKSLKQLNII
OXA-23-like_ARG28898.1_A.b.  DIKPQVGWLTGWVEQPDGKIVAFALNMEMRSEMPASIRNELLMKSLKQLNII
OXA-23-like_ARG30155.1_A.b.  DIKPQVGWLTGWVEQPDGKIVAFALNMEMRSEMPASIRNELLMKSLKQLNII
OXA-23-like_ARG35250.1_A.b.  DIKPQVGWLTGWVEQPDGKIVAFALNMEMRSEMPASIRNELLMKSLKQLNII
OXA-23-like_ASF49900.1_A.b.  DIKPQVGWLTGWVEQPDGKIVAFALNMEMRSEMPASIRNELLMKSLKQLNII
OXA-23-like_AUG13479.1_A.b.  DIKPQVGWLTGWVEQPDGKIVAFALNMEMRSEMPASIRNELLMKSLKQLNII
OXA-23-like_AVN25815.1_A.b.  DIKPQVGWLTGWVEQPDGKIVAFALNMEMRSEMPASIRNELLMKSLKQLNII
OXA-23-like_AVN25876.1_A.b.  DIKPQVGWLTGWVEQPDGKIVAFALNMEMRSEMPASIRNELLMKSLKQLNII
OXA-23-like_AVN25910.1_A.b.  DIKPQVGWLTGWVEQPDGKIVAFALNMEMRSEMPASIRNELLMKSLKQLNII
OXA-23-like_AWW79127.1_A.b.  DIKPQVGWLTGWVEQPDGKIVAFALNMEMRSEMPASIRNELLMKSLKQLNII
OXA-23-like_AWW83877.1_A.b.  DIKPQVGWLTGWVEQPDGKIVAFALNMEMRSEMPASIRNELLMKSLKQLNII
OXA-23-like_AWW86703.1_A.b.  DIKPQVGWLTGWVEQPDGKIVAFALNMEMRSEMPASIRNELLMKSLKQLNII
OXA-23-like_AYY88859.1_A.b.  DIKPQVGWLTGWVEQPDGKIVAFALNMEMRSEMPASIRNELLMKSLKQLNII
OXA-23-like_BAP68327.1_A.b.  DIKPQVGWLTGWVEQPDGKIVAFALNMEMRSEMPASIRNELLMKSLKQLNII
OXA-23-like_QAS95332.1_A.b.  DIKPQVGWLTGWVEQPDGKIVAFALNMEMRSEMPASIRNELLMKSLKQLNII
OXA-23-like_QAT02983.1_A.b.  DIKPQVGWLTGWVEQPDGKIVAFALNMEMRSEMPASIRNELLMKSLKQLNII
OXA-23-like_QBY15339.1_A.b.  DIKPQVGWLTGWVEQPDGKIVAFALNMEMRSEMPASIRNELLMKSLKQLNII
OXA-23-like_QCH32733.1_A.b.  DIKPQVGWLTGWVEQPDGKIVAFALNMEMRSEMPASIRNELLMKSLKQLNII
OXA-23-like_QCP17255.1_A.b.  DIKPQVGWLTGWVEQPDGKIVAFALNMEMRSEMPASIRNELLMKSLKQLNII
OXA-23-like_QCP24705.1_A.b.  DIKPQVGWLTGWVEQPDGKIVAFALNMEMRSEMPASIRNELLMKSLKQLNII
OXA-23-like_QCP27215.1_A.b.  DIKPQVGWLTGWVEQPDGKIVAFALNMEMRSEMPASIRNELLMKSLKQLNII
OXA-23-like_QCP29558.1_A.b.  DIKPQVGWLTGWVEQPDGKIVAFALNMEMRSEMPASIRNELLMKSLKQLNII
OXA-23-like_QCP40778.1_A.b.  DIKPQVGWLTGWVEQPDGKIVAFALNMEMRSEMPASIRNELLMKSLKQLNII
OXA-23-like_QCP43779.1_A.b.  DIKPQVGWLTGWVEQPDGKIVAFALNMEMRSEMPASIRNELLMKSLKQLNII
OXA-23-like_QCP44095.1_A.b.  DIKPQVGWLTGWVEQPDGKIVAFALNMEMRSEMPASIRNELLMKSLKQLNII
OXA-23-like_QCP47140.1_A.b.  DIKPQVGWLTGWVEQPDGKIVAFALNMEMRSEMPASIRNELLMKSLKQLNII
OXA-23-like_QCR88532.1_A.b.  DIKPQVGWLTGWVEQPDGKIVAFALNMEMRSEMPASIRNELLMKSLKQLNII
OXA-23-like_QDR92294.1_A.b.  DIKPQVGWLTGWVEQPDGKIVAFALNMEMRSEMPASIRNELLMKSLKQLNII
OXA-23-like_QDR95367.1_A.b.  DIKPQVGWLTGWVEQPDGKIVAFALNMEMRSEMPASIRNELLMKSLKQLNII
OXA-23-like_QEE57736.1_A.b.  DIKPQVGWLTGWVEQPDGKIVAFALNMEMRSEMPASIRNELLMKSLKQLNII
OXA-23-like_QEE57740.1_A.b.  DIKPQVGWLTGWVEQPDGKIVAFALNMEMRSEMPASIRNELLMKSLKQLNII
OXA-23-like_QEE57744.1_A.b.  DIKPQVGWLTGWVEQPDGKIVAFALNMEMRSEMPASIRNELLMKSLKQLNII
OXA-23-like_QEY29291.1_A.b.  DIKPQVGWLTGWVEQPDGKIVAFALNMEMRSEMPASIRNELLMKSLKQLNII
OXA-23-like_QEY29320.1_A.b.  DIKPQVGWLTGWVEQPDGKIVAFALNMEMRSEMPASIRNELLMKSLKQLNII
OXA-23-like_QEY29496.1_A.b.  DIKPQVGWLTGWVEQPDGKIVAFALNMEMRSEMPASIRNELLMKSLKQLNII
OXA-23-like_QIX29121.1_A.b.  DIKPQVGWLTGWVEQPDGKIVAFALNMEMRSEMPASIRNELLMKSLKQLNII
OXA-23-like_QIX32951.1_A.b.  DIKPQVGWLTGWVEQPDGKIVAFALNMEMRSEMPASIRNELLMKSLKQLNII
OXA-23-like_QIX36912.1_A.b.  DIKPQVGWLTGWVEQPDGKIVAFALNMEMRSEMPASIRNELLMKSLKQLNII
OXA-23-like_QIX40711.1_A.b.  DIKPQVGWLTGWVEQPDGKIVAFALNMEMRSEMPASIRNELLMKSLKQLNII
OXA-23-like_QIX44509.1_A.b.  DIKPQVGWLTGWVEQPDGKIVAFALNMEMRSEMPASIRNELLMKSLKQLNII
OXA-23-like_QJG69304.1_A.b.  DIKPQVGWLTGWVEQPDGKIVAFALNMEMRSEMPASIRNELLMKSLKQLNII
OXA-23-like_QJG70467.1_A.b.  DIKPQVGWLTGWVEQPDGKIVAFALNMEMRSEMPASIRNELLMKSLKQLNII
OXA-23-like_QJG70485.1_A.b.  DIKPQVGWLTGWVEQPDGKIVAFALNMEMRSEMPASIRNELLMKSLKQLNII
OXA-23-like_QJG71265.1_A.b.  DIKPQVGWLTGWVEQPDGKIVAFALNMEMRSEMPASIRNELLMKSLKQLNII
OXA-23-like_QJG74213.1_A.b.  DIKPQVGWLTGWVEQPDGKIVAFALNMEMRSEMPASIRNELLMKSLKQLNII
OXA-23-like_QJG81833.1_A.b.  DIKPQVGWLTGWVEQPDGKIVAFALNMEMRSEMPASIRNELLMKSLKQLNII
OXA-23-like_QJG84680.1_A.b.  DIKPQVGWLTGWVEQPDGKIVAFALNMEMRSEMPASIRNELLMKSLKQLNII
OXA-23-like_QJG89564.1_A.b.  DIKPQVGWLTGWVEQPDGKIVAFALNMEMRSEMPASIRNELLMKSLKQLNII
OXA-23-like_QJG89584.1_A.b.  DIKPQVGWLTGWVEQPDGKIVAFALNMEMRSEMPASIRNELLMKSLKQLNII
OXA-23-like_QJG90447.1_A.b.  DIKPQVGWLTGWVEQPDGKIVAFALNMEMRSEMPASIRNELLMKSLKQLNII
OXA-23-like_QJG93290.1_A.b.  DIKPQVGWLTGWVEQPDGKIVAFALNMEMRSEMPASIRNELLMKSLKQLNII
OXA-23-like_ADX93021.1_A.b.  DIKPQVGWLTGWVEQPDGKIVAFALNMEMRSEMPASIRNELLMKSLKQLNII
OXA-23-like_AXG83856.1_A.b.  NIKSQVGWLTGWVEQPDGKIVAFALNMEMRSEMPASIRNELLMKSLKQLNII
OXA-23-like_AML68547.1_A.b.  DIKPQVGWLTGWVEQPDGKIVAFALNMEMRSEMPASIRNELLMKSLKQLNII
OXA-23-like_ARG03241.1_A.b.  DIKPQVGWLTGWVEQPDGKIVAFALNMEMRSEMPASIRNELLMKSLKQLNII
OXA-23-like_AOX84133.1_A.b.  DIKPQVGWLTGWVEQPDGKIVAFALNMEMRSEMPASIRNELLMKSLKQLNII
OXA-23-like_AOX82595.1_A.b.  DIKPQVGWLTGWVEQPDGKIVAFALNMEMRSEMPASIRNELLMKSLKQLNII
OXA-23-like_AOX75920.1_A.b.  DIKPQVGWLTGWVEQPDGKIVAFALNMEMRSEMPASIRNELLMKSLKQLNII
OXA-23-like_AOX75888.1_A.b.  DIKPQVGWLTGWVEQPDGKIVAFALNMEMRSEMPASIRNELLMKSLKQLNII
```

```
OXA-23-like_AOX72443.1_A.b.  DIKPQVGWLTGWVEQPDGKIVAFALNMEMRSEMPASIRNELLMKSLKQLNII
OXA-23-like_AOX68171.1_A.b.  DIKPQVGWLTGWVEQPDGKIVAFALNMEMRSEMPASIRNELLMKSLKQLNII
OXA-23-like_AMC14310.1_A.b.  DIKPQVGWLTGWVEQPDGKIVAFALNMEMRSEMPASIRNELLMKSLKQLNII
OXA-23-like_AMC14301.1_A.b.  DIKPQVGWLTGWVEQPDGKIVAFALNMEMRSEMPASIRNELLMKSLKQLNII
OXA-23-like_ALY00722.1_A.b.  DIKPQVGWLTGWVEQPDGKIVAFALNMEMRSEMPASIRNELLMKSLKQLNII
OXA-23-like_ALX98871.1_A.b.  DIKPQVGWLTGWVEQPDGKIVAFALNMEMRSEMPASIRNELLMKSLKQLNII
OXA-23-like_AKA30333.1_A.b.  DIKPQVGWLTGWVEQPDGKIVAFALNMEMRSEMPASIRNELLMKSLKQLNII
OXA-23-like_AGQ10499.1_A.b.  DIKPQVGWLTGWVEQPDGKIVAFALNMEMRSEMPASIRNELLMKSLKQLNII
OXA-23-like_AGQ05635.1_A.b.  DIKPQVGWLTGWVEQPDGKIVAFALNMEMRSEMPASIRNELLMKSLKQLNII
OXA-23-like_ACJ39972.1_A.b.  DIKPQVGWLTGWVEQPDGKIVAFALNMEMRSEMPASIRNELLMKSLKQLNII
OXA-23-like_QKK76748.1_A.b.  DIKPQVGWLTGWVEQPDGKIVAFALNMEMRSEMPASIRNELLMKSLKQLNII
OXA-23-like_QKK76707.1_A.b.  DIKPQVGWLTGWVEQPDGKIVAFALNMEMRSEMPASIRNELLMKSLKQLNII
OXA-23-like_QKK76393.1_A.b.  DIKPQVGWLTGWVEQPDGKIVAFALNMEMRSEMPASIRNELLMKSLKQLNII
OXA-23-like_QFX72557.1_A.b.  DIKPQVGWLTGWVEQPDGKIVAFALNMEMRSEMPASIRNELLMKSLKQLNII
OXA-23-like_QFZ56693.1_A.b.  DIKPQVGWLTGWVEQPDGKIVAFALNMEMRSEMPASIRNELLMKSLKQLNII
OXA-23-like_AYC00513.1_A.b.  DIKPQVGWLTGWVEQPDGKIVAFALNMEMRSEMPASIRNELLMKSLKQLNII
OXA-23-like_AYC00505.1_A.b.  DIKPQVGWLTGWVEQPDGKIVAFALNMEMRSEMPASIRNELLMKSLKQLNII
OXA-23-like_ARG39357.1_A.b.  DIKPQVGWLTGWVEQPDGKIVAFALNMEMRSEMPASIRNELLMKSLKQLNII
OXA-23-like_ARG23739.1_A.b.  DIKPQVGWLTGWVEQPDGKIVAFALNMEMRSEMPASIRNELLMKSLKQLNII
OXA-23-like_ARG23713.1_A.b.  DIKPQVGWLTGWVEQPDGKIVAFALNMEMRSEMPASIRNELLMKSLKQLNII
OXA-23-like_ARG03186.1_A.b.  DIKPQVGWLTGWVEQPDGKIVAFALNMEMRSEMPASIRNELLMKSLKQLNII
OXA-23-like_AEP06450.2_A.b.  DIKPQVGWLTGWVEQPDGKIVAFALNMEMRSEMPASIRNELLMKSLKQLNII
OXA-23-like_AML75286.1_A.b.  DIKPQVGWLTGWVEQPDGKIVAFALNMEMRSEMPASIRNELLMKSLKQLNII
OXA-23-like_AML75277.1_A.b.  DIKPQVGWLTGWVEQPDGKIVAFALNMEMRSEMPASIRNELLMKSLKQLNII
OXA-23-like_AML71526.1_A.b.  DIKPQVGWLTGWVEQPDGKIVAFALNMEMRSEMPASIRNELLMKSLKQLNII
OXA-23-like_AML69469.1_A.b.  DIKPQVGWLTGWVEQPDGKIVAFALNMEMRSEMPASIRNELLMKSLKQLNII
OXA-23-like_AML66436.1_A.b.  DIKPQVGWLTGWVEQPDGKIVAFALNMEMRSEMPASIRNELLMKSLKQLNII
OXA-23-like_AML65160.1_A.b.  DIKPQVGWLTGWVEQPDGKIVAFALNMEMRSEMPASIRNELLMKSLKQLNII
OXA-23-like_AML65128.1_A.b.  DIKPQVGWLTGWVEQPDGKIVAFALNMEMRSEMPASIRNELLMKSLKQLNII
OXA-23-like_AMN00602.1_A.b.  DIKPQVGWLTGWVEQPDGKIVAFALNMEMRSEMPASIRNELLMKSLKQLNII
OXA-23-like_AMN00587.1_A.b.  DIKPQVGWLTGWVEQPDGKIVAFALNMEMRSEMPASIRNELLMKSLKQLNII
OXA-23-like_AMN00578.1_A.b.  DIKPQVGWLTGWVEQPDGKIVAFALNMEMRSEMPASIRNELLMKSLKQLNII
OXA-23-like_AMN00569.1_A.b.  DIKPQVGWLTGWVEQPDGKIVAFALNMEMRSEMPASIRNELLMKSLKQLNII
OXA-23-like_QJF37605.1_A.b.  DIKPQVGWLTGWVEQPDGKIVAFALNMEMRSEMPASIRNELLMKSLKQLNII
OXA-23-like_QJF33717.1_A.b.  DIKPQVGWLTGWVEQPDGKIVAFALNMEMRSEMPASIRNELLMKSLKQLNII
OXA-23-like_QJH05102.1_A.b.  DIKPQVGWLTGWVEQPDGKIVAFALNMEMRSEMPASIRNELLMKSLKQLNII
OXA-23-like_QJH23846.1_A.b.  DIKPQVGWLTGWVEQPDGKIVAFALNMEMRSEMPASIRNELLMKSLKQLNII
OXA-23-like_QCH35083.1_A.b.  DIKPQVGWLTGWVEQPDGKIVAFALNMEMRSEMPASIRNELLMKSLKQLNII
OXA-23-like_QAT07029.1_A.b.  DIKPQVGWLTGWVEQPDGKIVAFALNMEMRSEMPASIRNELLMKSLKQLNII
OXA-23-like_QAS99734.1_A.b.  DIKPQVGWLTGWVEQPDGKIVAFALNMEMRSEMPASIRNELLMKSLKQLNII
OXA-23-like_AVE56824.1_A.b.  DIKPQVGWLTGWVEQPDGKIVAFALNMEMRSEMPASIRNELLMKSLKQLNII
OXA-23-like_AVE44374.1_A.b.  DIKPQVGWLTGWVEQPDGKIVAFALNMEMRSEMPASIRNELLMKSLKQLNII
OXA-23-like_ATR89604.1_A.b.  DIKPQVGWLTGWVEQPDGKIVAFALNMEMRSEMPASIRNELLMKSLKQLNII
OXA-23-like_ARG29802.1_A.b.  DIKPQVGWLTGWVEQPDGKIVAFALNMEMRSEMPASIRNELLMKSLKQLNII
OXA-23-like_APF45746.1_A.b.  DIKPQVGWLTGWVEQPDGKIVAFALNMEMRSEMPASIRNELLMKSLKQLNII
OXA-23-like_APQ95067.1_A.b.  DIKPQVGWLTGWVEQPDGKIVAFALNMEMRSEMPASIRNELLMKSLKQLNII
OXA-23-like_APQ87413.1_A.b.  DIKPQVGWLTGWVEQPDGKIVAFALNMEMRSEMPASIRNELLMKSLKQLNII
OXA-23-like_ATR88802.1_A.b.  DIKPQVGWLTGWVEQPDGKIVAFALNMEMRSEMPASIRNELLMKSLKQLNII
OXA-23-like_ATR88943.1_A.b.  DIKPQVGWLTGWVEQPDGKIVAFALNMEMRSEMPASIRNELLMKSLKQLNII
OXA-23-like_SBS23848.1_A.b.  DIKPQVGWLTGWVEQPDGKIVAFALNMEMRSEMPASIRNELLMKSLKQLNII
OXA-23-like_AOP62462.1_A.b.  DIKPQVGWLTGWVEQPDGKIVAFALNMEMRSEMPASIRNELLMKSLKQLNII
4JF4_Beta-lactamase          DIKPQVGWLTGWVEQPDGKIVAFALNMEMRSEMPASIRNELLMKSLKQLNII
OXA-23-like_QCP18237.1_A.b.  DIKPQVGWLTGWVEQPDGKIVAFALNMEMRSEMPASIRNELLMKSLKQLNII
OXA-23-like_QRN19109.1_A.b.  DIKPQVGWLTGWVEQPDGKIVAFALNMEMRSEMPASIRNELLMKSLKQLNII
                        acc
```

OXA-24-like subfamily

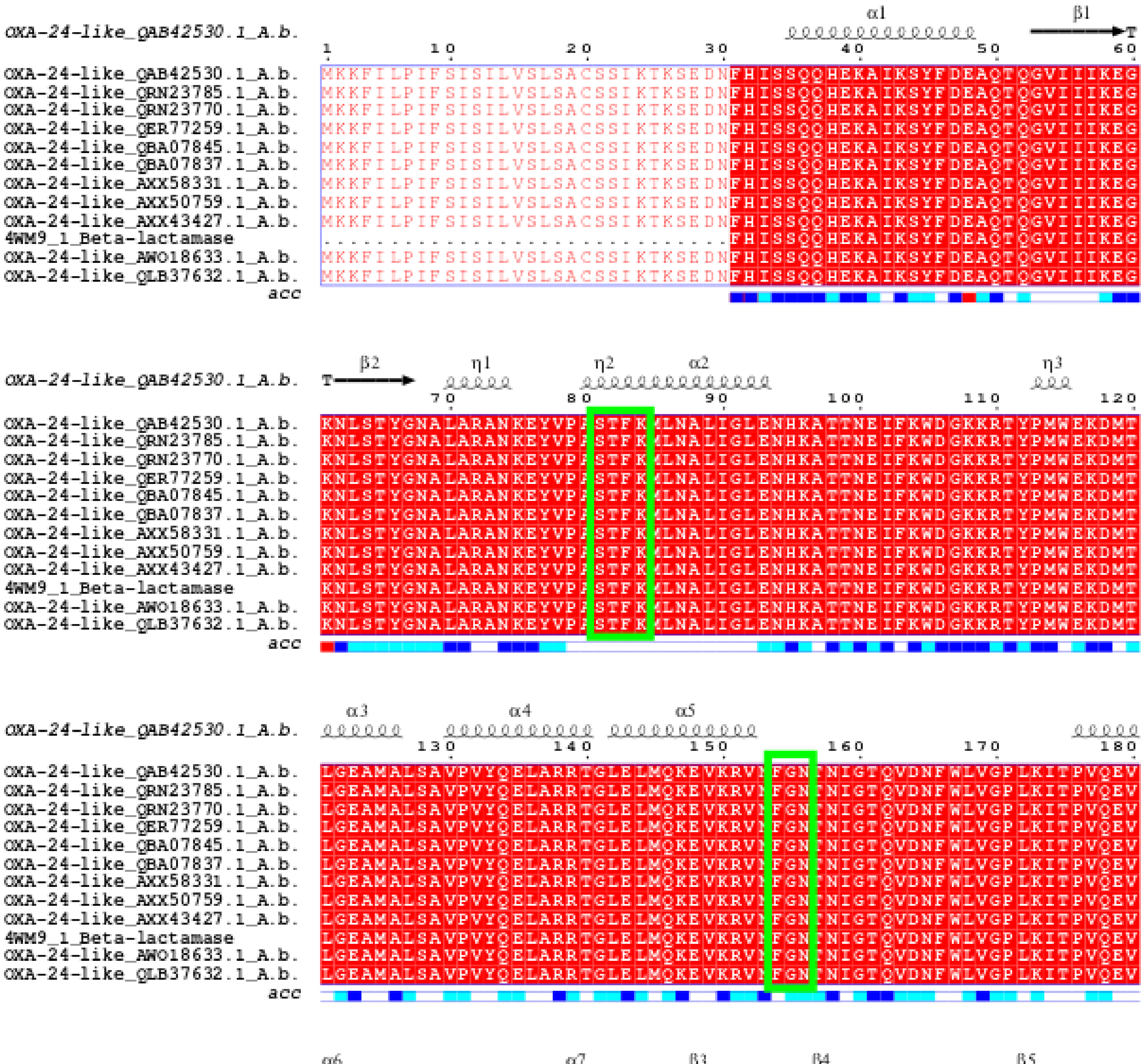

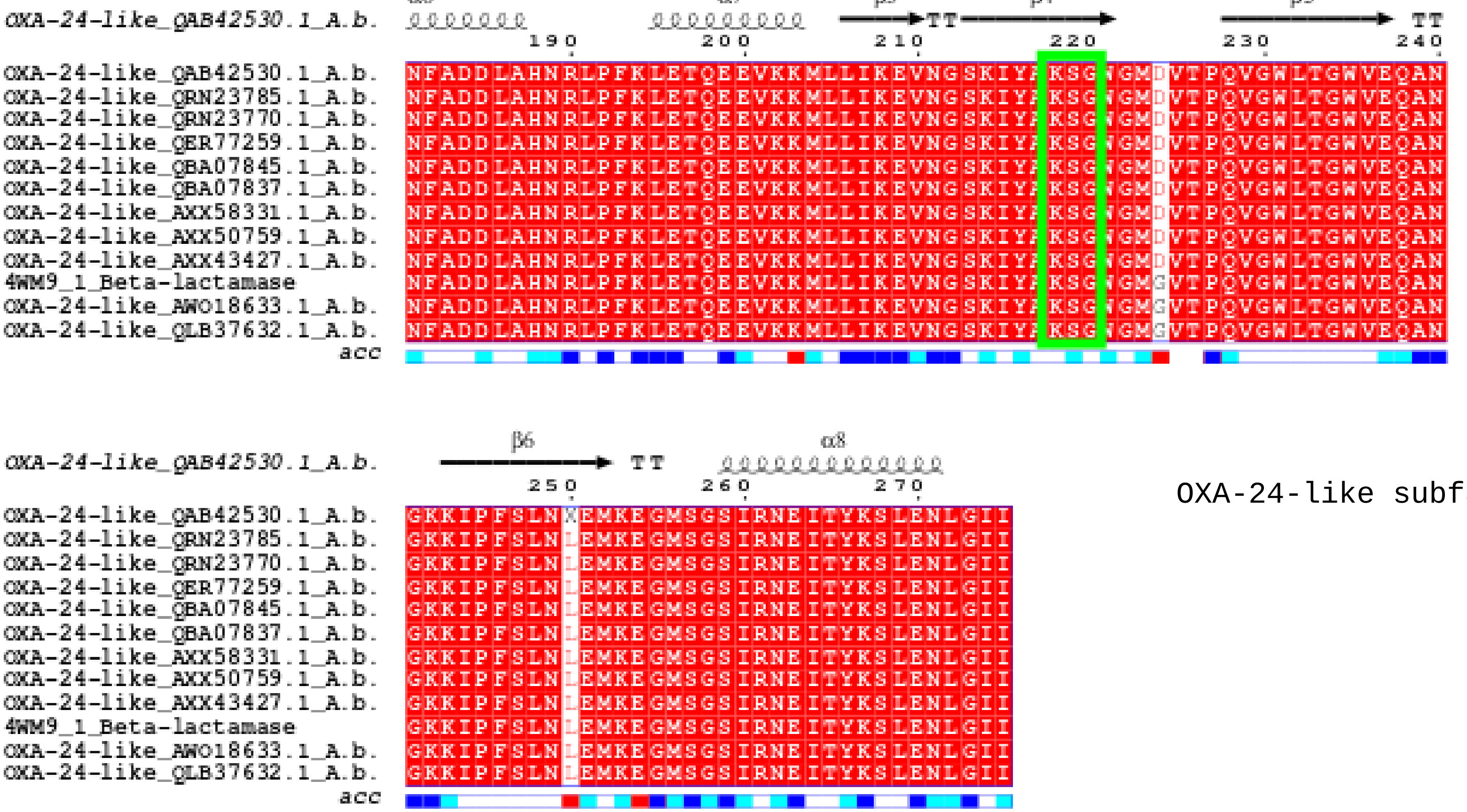

OXA-24-like subfamily

OXA-33-like subfamily

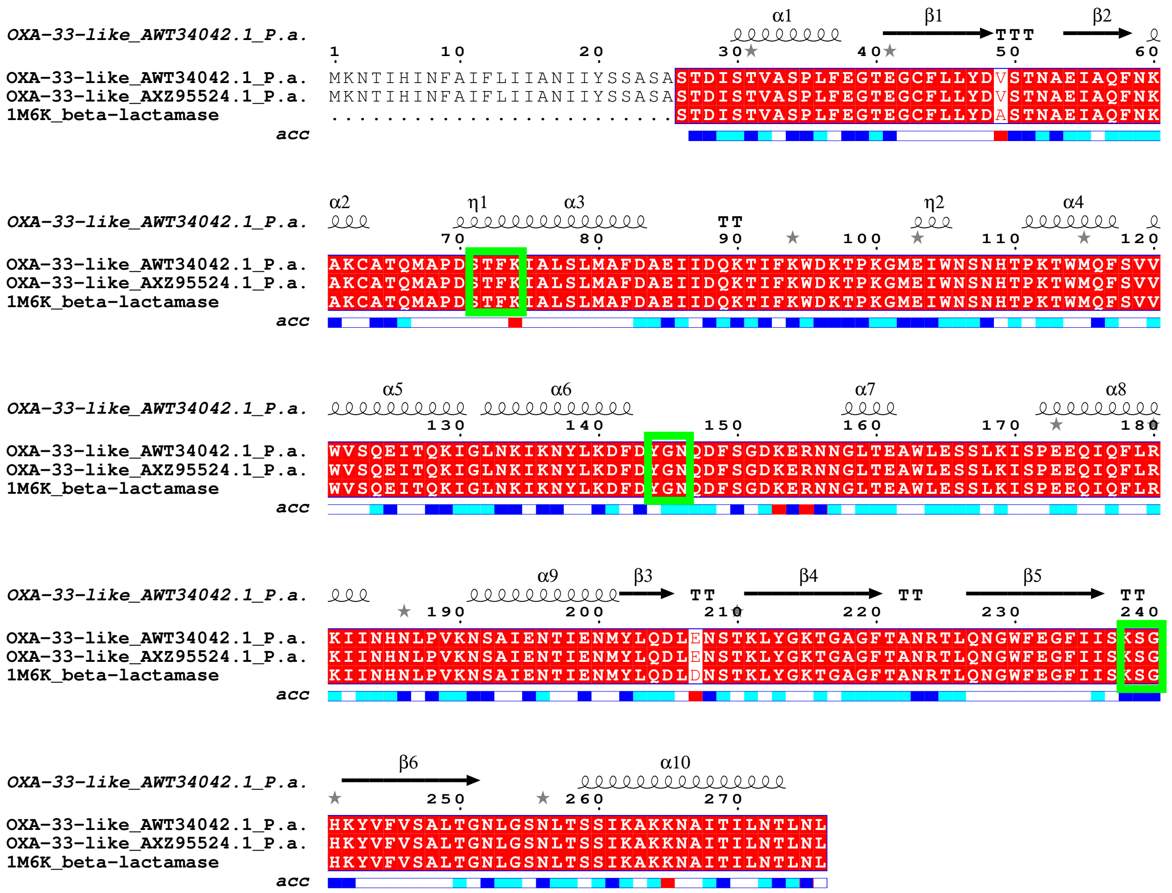

OXA-50-like subfamily

```
                                             η1        α1             β1         β2      α2
                         TTT  TTT
                         1         10        20        30        40        50
OXA-50-like_QQW22444.1_P.a.   MRPLLFSALLLL.SGHAQASEWNDSRAVDKLFGAAGVKGTFVLYDVQRQRYVGHDRERAE
OXA-50-like_QGQ00241.1_P.a.   MRPLLFSALLLL.SGHAQASEWNDSRAVDKLFGAAGVKGTFVLYDVQRQRYVGHDRERAE
OXA-50-like_QQW19268.1_P.a.   MRPLLFSALLLL.SGHAQASEWNDSRAVDKLFGAAGVKGTFVLYDVQRQRYVGHDRERAE
OXA-50-like_QQW06309.1_P.a.   MRPLLFSALLLL.SGHAQASEWNDSRAVDKLFGAAGVKGTFVLYDVQRQRYVGHDRERAE
OXA-50-like_QQV61463.1_P.a.   MRPLLFSALLLL.SGHAQASEWNDSRAVDKLFGAAGVKGTFVLYDVQRQRYVGHDRERAE
OXA-50-like_QPP39331.1_P.a.   MRPLLFSALLLL.SGHAQASEWNDSRAVDKLFGAAGVKGTFVLYDVQRQRYVGHDRERAE
OXA-50-like_QNE62162.1_P.a.   MRPLLFSALLLL.SGHAQASEWNDSRAVDKLFGAAGVKGTFVLYDVQRQRYVGHDRERAE
OXA-50-like_QLF44164.1_P.a.   MRPLLFSALLLL.SGHAQASEWNDSRAVDKLFGAAGVKGTFVLYDVQRQRYVGHDRERAE
OXA-50-like_QLF18710.1_P.a.   MRPLLFSALLLL.SGHAQASEWNDSRAVDKLFGAAGVKGTFVLYDVQRQRYVGHDRERAE
OXA-50-like_QEK46239.1_P.a.   MRPLLFSALLLL.SGHAQASEWNDSRAVDKLFGAAGVKGTFVLYDVQRQRYVGHDRERAE
OXA-50-like_QJA41232.1_P.a.   MRPLLFSALLLL.SGHAQASEWNDSRAVDKLFGAAGVKGTFVLYDVQRQRYVGHDRERAE
OXA-50-like_AYZ58090.1_P.a.   MRPLLFSALLLL.SGHAQASEWNDSRAVDKLFGAAGVKGTFVLYDVQRQRYVGHDRERAE
OXA-50-like_QDR20381.1_P.a.   MRPLLFSALLLL.SGHAQASEWNDSRAVDKLFGAAGVKGTFVLYDVQRQRYVGHDRERAE
OXA-50-like_AYW69566.1_P.a.   MRPLLFSALLLL.SGHAQASEWNDSRAVDKLFGAAGVKGTFVLYDVQRQRYVGHDRERAE
OXA-50-like_AYZ45913.1_P.a.   MRPLLFSALLLL.SGHAQASEWNDSRAVDKLFGAAGVKGTFVLYDVQRQRYVGHDRERAE
OXA-50-like_AYK26584.1_P.a.   MRPLLFSALLLL.SGHAQASEWNDSRAVDKLFGAAGVKGTFVLYDVQRQRYVGHDRERAE
OXA-50-like_ASM88695.1_P.a.   MRPLLFSALLLL.SGHAQASEWNDSRAVDKLFGAAGVKGTFVLYDVQRQRYVGHDRERAE
OXA-50-like_AUA80490.1_P.a.   MRPLLFSALLLL.SGHAQASEWNDSRAVDKLFGAAGVKGTFVLYDVQRQRYVGHDRERAE
OXA-50-like_AUB05120.1_P.a.   MRPLLFSALLLL.SGHAQASEWNDSRAVDKLFGAAGVKGTFVLYDVQRQRYVGHDRERAE
OXA-50-like_AVR86641.1_P.a.   MRPLLFSALLLL.SGHAQASEWNDSRAVDKLFGAAGVKGTFVLYDVQRQRYVGHDRERAE
OXA-50-like_AXS75147.1_P.a.   MRPLLFSALLLL.SGHAQASEWNDSRAVDKLFGAAGVKGTFVLYDVQRQRYVGHDRERAE
OXA-50-like_ALS13221.1_P.a.   MRPLLFSALLLL.SGHAQASEWNDSRAVDKLFGAAGVKGTFVLYDVQRQRYVGHDRERAE
OXA-50-like_ALY73377.1_P.a.   MRPLLFSALLLL.SGHAQASEWNDSRAVDKLFGAAGVKGTFVLYDVQRQRYVGHDRERAE
OXA-50-like_ANA70339.1_P.a.   MRPLLFSALLLL.SGHAQASEWNDSRAVDKLFGAAGVKGTFVLYDVQRQRYVGHDRERAE
OXA-50-like_APB68390.1_P.a.   MRPLLFSALLLL.SGHAQASEWNDSRAVDKLFGAAGVKGTFVLYDVQRQRYVGHDRERAE
OXA-50-like_ARI94830.1_P.a.   MRPLLFSALLLL.SGHAQASEWNDSRAVDKLFGAAGVKGTFVLYDVQRQRYVGHDRERAE
OXA-50-like_ARJ01367.1_P.a.   MRPLLFSALLLL.SGHAQASEWNDSRAVDKLFGAAGVKGTFVLYDVQRQRYVGHDRERAE
OXA-50-like_ASA19039.1_P.a.   MRPLLFSALLLL.SGHAQASEWNDSRAVDKLFGAAGVKGTFVLYDVQRQRYVGHDRERAE
OXA-50-like_AUA74232.1_P.a.   MRPLLFSALLLL.SGHAQASEWNNSRAVDKLFGAAGVKGTFVLYDVQRQRYVGHDRERAE
OXA-50-like_QCC69374.1_P.a.   MRPLLFSALLLL.SGHAQASEWNDSRAVDKLFGAAGVKGTFVLYDVQRQRYVGHDRERAE
OXA-50-like_ATH06157.1_P.a.   MRPLLFSALLLL.SGHAQASEWNDSQAVDKLFGAAGVKGTFVLYDVQRQRYVGHDRERAE
OXA-50-like_AZZ14888.1_P.a.   MRPLLFSALLLL.SGHAQASEWNDSQAVDKLFGAAGVKGTFVLYDVQRQRYVGHDRERAE
OXA-50-like_VDK95206.1_P.a.   MRPLLFSALLLL.SGHAQASEWNDSQAVDKLFGAAGVKGTFVLYDVQRQRYVGHDRERAE
OXA-50-like_VDL11569.1_P.a.   MRPLLFSALLLL.SGHAQASEWNDSQAVDKLFGAAGVKGTFVLYDVQRQRYVGHDRERAE
OXA-50-like_VDL54838.1_P.a.   MRPLLFSALLLL.SGHAQASEWNDSQAVDKLFGAAGVKGTFVLYDVQRQRYVGHDRERAE
OXA-50-like_VDL56340.1_P.a.   MRPLLFSALLLL.SGHAQASEWNDSQAVDKLFGAAGVKGTFVLYDVQRQRYVGHDRERAE
OXA-50-like_VDL57163.1_P.a.   MRPLLFSALLLL.SGHAQASEWNDSQAVDKLFGAAGVKGTFVLYDVQRQRYVGHDRERAE
OXA-50-like_ALZ29437.1_P.a.   MRPLLFSALLLL.SGHAQASEWNDSQAVDKLFGAAGVKGTFVLYDVQRQRYVGHDRERAE
OXA-50-like_AON10100.1_P.a.   MRPLLFSALLLL.SGHAQASEWNDSQAVDKLFGAAGVKGTFVLYDVQRQRYVGHDRERAE
OXA-50-like_AON16083.1_P.a.   MRPLLFSALLLL.SGHAQASEWNDSQAVDKLFGAAGVKGTFVLYDVQRQRYVGHDRERAE
OXA-50-like_AON22092.1_P.a.   MRPLLFSALLLL.SGHAQASEWNDSQAVDKLFGAAGVKGTFVLYDVQRQRYVGHDRERAE
OXA-50-like_AON28079.1_P.a.   MRPLLFSALLLL.SGHAQASEWNDSQAVDKLFGAAGVKGTFVLYDVQRQRYVGHDRERAE
OXA-50-like_AON34087.1_P.a.   MRPLLFSALLLL.SGHAQASEWNDSQAVDKLFGAAGVKGTFVLYDVQRQRYVGHDRERAE
OXA-50-like_AON40072.1_P.a.   MRPLLFSALLLL.SGHAQASEWNDSQAVDKLFGAAGVKGTFVLYDVQRQRYVGHDRERAE
OXA-50-like_AON46078.1_P.a.   MRPLLFSALLLL.SGHAQASEWNDSQAVDKLFGAAGVKGTFVLYDVQRQRYVGHDRERAE
OXA-50-like_AON52080.1_P.a.   MRPLLFSALLLL.SGHAQASEWNDSQAVDKLFGAAGVKGTFVLYDVQRQRYVGHDRERAE
OXA-50-like_ARG47835.1_P.a.   MRPLLFSALLLL.SGHAQASEWNDSQAVDKLFGAAGVKGTFVLYDVQRQRYVGHDRERAE
OXA-50-like_ASP05026.1_P.a.   MRPLLFSALLLL.SGHAQASEWNDSQAVDKLFGAAGVKGTFVLYDVQRQRYVGHDRERAE
OXA-50-like_ASP13070.1_P.a.   MRPLLFSALLLL.SGHAQASEWNDSQAVDKLFGAAGVKGTFVLYDVQRQRYVGHDRERAE
OXA-50-like_AVE36468.1_P.a.   MRPLLFSALLLL.SGHAQASEWNDSQAVDKLFGAAGVKGTFVLYDVQRQRYVGHDRERAE
OXA-50-like_AXL80491.1_P.a.   MRPLLFSALLLL.SGHAQASEWNDSQAVDKLFGAAGVKGTFVLYDVQRQRYVGHDRERAE
OXA-50-like_QDR05681.1_P.a.   MRPLLFSALLLL.SGHAQASEWNDSQAVDKLFGAAGVKGTFVLYDVQRQRYVGHDRERAE
OXA-50-like_QGJ36847.1_P.a.   MRPLLFSALLLL.SGHAQASEWNDSQAVDKLFGAAGVKGTFVLYDVQRQRYVGHDRERAE
OXA-50-like_QKG35875.1_P.a.   MRPLLFSALLLL.SGHAQASEWNDSQAVDKLFGAAGVKGTFVLYDVQRQRYVGHDRERAE
OXA-50-like_QKR24763.1_P.a.   MRPLLFSALLLL.SGHAQASEWNDSQAVDKLFGAAGVKGTFVLYDVQRQRYVGHDRERAE
OXA-50-like_QOQ64022.1_P.a.   MRPLLFSALLLL.SGHAQASEWNDSQAVDKLFGAAGVKGTFVLYDVQRQRYVGHDRERAE
OXA-50-like_QQD36200.1_P.a.   MRPLLFSALLLL.SGHAQASEWNDSQAVDKLFGAAGVKGTFVLYDVQRQRYVGHDRERAE
OXA-50-like_AON75377.1_P.a.   MRPLLFSALLLL.SGHAQASEWNDSQAVDKLFGAAGVKGTFVLYDVQRQRYVGHDRERAE
OXA-50-like_QDY03768.1_P.a.   MRPLLFSALLLL.SGHAQASEWNDSQAVDKLFGAAGVKGTFVLYDVQRQRYVGHDRERAE
OXA-50-like_QPP46609.1_P.a.   MRPLLFSALLLL.SGHAQASEWNDSQAVDKLFGAAGVKGTFVLYDVQRQRYVGHDRERAE
OXA-50-like_QIZ29678.1_P.a.   MRPLFFSALLLLFSGHAQASEWNDSQAVDKLFGAAGVKGTFVLYDVQRQRYVGHDRGRAE
OXA-50-like_QKR77815.1_P.a.   MRPLLFSALLLL.SGHAQASEWNDSQAVDKLFGAAGVKGTFVLYDVQRQRYVGHDRERAE
OXA-50-like_AMA40693.1_P.a.   MRPLLFSALLLL.SGHAQASEWNDSQAVDKLFGAAGVKGTFVLYDVQRQRYVGHDRERAE
OXA-50-like_QKE99620.1_P.a.   MRPLLFSALLLL.SGHAQASEWNDSQAVDKLFGAAGVKGTFVLYDVQRQRYVGHDRERAE
OXA-50-like_AVZ37799.1_P.a.   MRPLLFSALLLL.SGHAQASEWNDSQAVDKLFGAAGVKGTFVLYDVQRQRYVGHDRERAE
OXA-50-like_ASA32790.1_P.a.   MRPLLFSTLLLL.SGHAQASEWNDSQAVDKLFGAAGVKGTFVLYDVQRQRYVGHDRERAE
OXA-50-like_ASD07216.1_P.a.   MRPLLFSTLLLL.SGHAQASEWNDSQAVDKLFGAAGVKGTFVLYDVQRQRYVGHDRERAE
OXA-50-like_VDL31009.1_P.a.   MRPLLFSTLLLL.SGHAQASEWNDSQAVDKLFGAAGVKGTFVLYDVQRQRYVGHDRERAE
OXA-50-like_VDL53067.1_P.a.   MRPLLFSTLLLL.SGHAQASEWNDSQAVDKLFGAAGVKGTFVLYDVQRQRYVGHDRERAE
OXA-50-like_AYF72086.1_P.a.   MRPLLFSALLLL.SGHAQASEWNDSQAVDKLFGAAGVKGTFVLYDVQRQRYVGHDRERAE
OXA-50-like_AYQ83944.1_P.a.   MRPLLFSALLLL.SGHAQASEWNDSQAVDKLFGAAGVKGTFVLYDVQRQRYVGHDRERAE
OXA-50-like_AYR15974.1_P.a.   MRPLLFSALLLL.SGHAQASEWNDSQAVDKLFGAAGVKGTFVLYDVQRQRYVGHDRERAE
OXA-50-like_AZN03541.1_P.a.   MRPLLFSALLLL.SGHAQASEWNDSQAVDKLFGAAGVKGTFVLYDVQRQRYVGHDRERAE
OXA-50-like_AZN09267.1_P.a.   MRPLLFSALLLL.SGHAQASEWNDSQAVDKLFGAAGVKGTFVLYDVQRQRYVGHDRERAE
OXA-50-like_AZN16304.1_P.a.   MRPLLFSALLLL.SGHAQASEWNDSQAVDKLFGAAGVKGTFVLYDVQRQRYVGHDRERAE
OXA-50-like_AZN49449.1_P.a.   MRPLLFSALLLL.SGHAQASEWNDSQAVDKLFGAAGVKGTFVLYDVQRQRYVGHDRERAE
OXA-50-like_AZN54830.1_P.a.   MRPLLFSALLLL.SGHAQASEWNDSQAVDKLFGAAGVKGTFVLYDVQRQRYVGHDRERAE
OXA-50-like_AZN61319.1_P.a.   MRPLLFSALLLL.SGHAQASEWNDSQAVDKLFGAAGVKGTFVLYDVQRQRYVGHDRERAE
OXA-50-like_QPZ71933.1_P.a.   MRPLLFSALLLL.SGHAQASEWNDSQAVDKLFGAAGVKGTFVLYDVQRQRYVGHDRERAE
OXA-50-like_QQM09528.1_P.a.   MRPLLFSALLLL.SGHAQASEWNDSQAVDKLFGAAGVKGTFVLYDVQRQRYVGHDRERAE
OXA-50-like_AVN44779.1_P.a.   MRPLLFSALLLL.SGHAQASEWNDSQAVDKLFGAAGVKGTFVLYDVQRQRYVGHDRERAE
OXA-50-like_QKR65469.1_P.a.   MRPLLFSALLLL.SGHAQASEWNDSQAVDKLFGAAGVKGTFVLYDVQRQRYVGHDRERAE
OXA-50-like_AYW63307.1_P.a.   MRPLLFSALLLL.SGHAQASEWNDSQAVDKLFGAAGVKGTFVLYDVQRQRYVGHDRERAE
OXA-50-like_QPZ65977.1_P.a.   MRPLLFSALLLL.SGHAQASEWNDSQAVDKLFGAAGVKGTFVLYDVQRQLYVGHDRERAE
OXA-50-like_QHF92313.1_P.a.   MRPLLFSALLLL.SGHTQASEWNDSQAVDKLFGAAGVKGTFVLYDVQRQRYVGHDRERAE
OXA-50-like_AZM83163.1_P.a.   MRPLLFSALLLL.SGHTQASEWNDSQAVDKLFGAAGVKGTFVLYDVQRQRYVGHDRERAE
OXA-50-like_ALY58484.1_P.a.   MRPLLFSALLLL.SGHTQASEWNDSQAVDKLFGAAGVKGTFVLYDVQRQRYVGHDRERAE
OXA-50-like_AHA19368.1_P.a.   MRPLLFSALLLL.SGHTQASEWNDSQAVDKLFGAAGVKGTFVLYDVQRQRYVGHDRERAE
OXA-50-like_ALE51523.1_P.a.   MRPLLFSALLLL.SGHTQASEWNDSQAVDKLFGAAGVKGTFVLYDVQRQRYVGHDRERAE
OXA-50-like_ALY50692.1_P.a.   MRPLLFSALLLL.SGHTQASEWNDSQAVDKLFGAAGVKGTFVLYDVQRQRYVGHDRERAE
OXA-50-like_ALY86245.1_P.a.   MRPLLFSALLLL.SGHTQASEWNDSQAVDKLFGAAGVKGTFVLYDVQRQRYVGHDRERAE
OXA-50-like_ALZ22498.1_P.a.   MRPLLFSALLLL.SGHTQASEWNDSQAVDKLFGAAGVKGTFVLYDVQRQRYVGHDRERAE
OXA-50-like_AOP60986.1_P.a.   MRPLLFSALLLL.SGHTQASEWNDSQAVDKLFGAAGVKGTFVLYDVQRQRYVGHDRERAE
OXA-50-like_ASD00726.1_P.a.   MRPLLFSALLLL.SGHTQASEWNDSQAVDKLFGAAGVKGTFVLYDVQRQRYVGHDRERAE
OXA-50-like_AXR31931.1_P.a.   MRPLLFSALLLL.SGHTQASEWNDSQAVDKLFGAAGVKGTFVLYDVQRQRYVGHDRERAE
OXA-50-like_AYW76172.1_P.a.   MRPLLFSALLLL.SGHTQASEWNDSQAVDKLFGAAGVKGTFVLYDVQRQRYVGHDRERAE
OXA-50-like_QAS76324.1_P.a.   MRPLLFSALLLL.SGHTQASEWNDSQAVDKLFGAAGVKGTFVLYDVQRQRYVGHDRERAE
OXA-50-like_QDD38928.1_P.a.   MRPLLFSALLLL.SGHTQASEWNDSQAVDKLFGAAGVKGTFVLYDVQRQRYVGHDRERAE
OXA-50-like_QES85928.1_P.a.   MRPLLFSALLLL.SGHTQASEWNDSQAVDKLFGAAGVKGTFVLYDVQRQRYVGHDRERAE
OXA-50-like_QJH86618.1_P.a.   MRPLLFSALLLL.SGHTQASEWNDSQAVDKLFGAAGVKGTFVLYDVQRQRYVGHDRERAE
OXA-50-like_QKK88642.1_P.a.   MRPLLFSALLLL.SGHTQASEWNDSQAVDKLFGAAGVKGTFVLYDVQRQRYVGHDRERAE
OXA-50-like_QKR30875.1_P.a.   MRPLLFSALLLL.SGHTQASEWNDSQAVDKLFGAAGVKGTFVLYDVQRQRYVGHDRERAE
OXA-50-like_QKR36250.1_P.a.   MRPLLFSALLLL.SGHTQASEWNDSQAVDKLFGAAGVKGTFVLYDVQRQRYVGHDRERAE
```

OXA-50-like subfamily

```
OXA-50-like_QKS42441.1_P.a.   MRPLLFSALLLL.SGHTQASEWNDSQAVDKLFGAAGVKGTFVLYDVQRQRYVGHDRERAE
OXA-50-like_QPP27676.1_P.a.   MRPLLFSALLLL.SGHTQASEWNDSQAVDKLFGAAGVKGTFVLYDVQRQRYVGHDRERAE
OXA-50-like_QPV54155.1_P.a.   MRPLLFSALLLL.SGHTQASEWNDSQAVDKLFGAAGVKGTFVLYDVQRQRYVGHDRERAE
OXA-50-like_QPZ84995.1_P.a.   MRPLLFSALLLL.SGHTQASEWNDSQAVDKLFGAAGVKGTFVLYDVQRQRYVGHDRERAE
OXA-50-like_ANP59968.1_P.a.   MRPLLFSALLLL.SGHTQASEWNDSQAVDKLFGAAGVKGTFVLYDVQRQRYVGHDRERAE
OXA-50-like_QGQ06905.1_P.a.   MRPLLFSALLLL.SGHTQASEWNDSQAVDKLFGAAGVKGTFVLYDVQRQRYVGHDRERAE
OXA-50-like_AXR14368.1_P.a.   MRPLLFSALLLL.SGHTQASEWNDSQAVDKLFGAAGVKGTFVLYDVQRQRYVGHDRERAE
OXA-50-like_ALP57413.1_P.a.   MRPLLFSALLLL.SGHTQASEWNDSQAVDKLFGAAGVKGTFVLYDVQRQCYVGHDRERAE
OXA-50-like_QKR41777.1_P.a.   MRPLLFSALLLL.SGHTQASEWNDSQAVDKLFGAAGVKGTFVLYDVQRQCYVGHDRERAE
OXA-50-like_ALY36156.1_P.a.   MRPLLFSALLLL.SGHTQASEWNDSQAVDKLFGAAGVKGTFVLYDVQRQCYVGHDRERAE
OXA-50-like_ALY52550.1_P.a.   MRPLLFSALLLL.SGHTQASEWNDSQAVDKLFGAAGVKGTFVLYDVQRQCYVGHDRERAE
OXA-50-like_ANI07822.1_P.a.   MRPLLFSALLLL.SGHTQASEWNDSQAVDKLFGAAGVKGTFVLYDVQRQCYVGHDRERAE
OXA-50-like_AWS88163.1_P.a.   MRPLLFSALLLL.SGHTQASEWNDSQAVDKLFGAAGVKGTFVLYDVQRQCYVGHDRERAE
OXA-50-like_AXN29478.1_P.a.   MRPLLFSALLLL.SGHTQASEWNDSQAVDKLFGAAGVKGTFVLYDVQRQCYVGHDRERAE
OXA-50-like_QBC08854.1_P.a.   MRPLLFSALLLL.SGHTQASEWNDSQAVDKLFGAAGVKGTFVLYDVQRQCYVGHDRERAE
OXA-50-like_QDD49249.1_P.a.   MRPLLFSALLLL.SGHTQASEWNDSQAVDKLFGAAGVKGTFVLYDVQRQCYVGHDRERAE
OXA-50-like_QDR11871.1_P.a.   MRPLLFSALLLL.SGHTQASEWNDSQAVDKLFGAAGVKGTFVLYDVQRQCYVGHDRERAE
OXA-50-like_QKP84324.1_P.a.   MRPLLFSALLLL.SGHTQASEWNDSQAVDKLFGAAGVKGTFVLYDVQRQCYVGHDRERAE
OXA-50-like_QKR71283.1_P.a.   MRPLLFSALLLL.SGHTQASEWNDSQAVDKLFGAAGVKGTFVLYDVQRQCYVGHDRERAE
OXA-50-like_QLA53973.1_P.a.   MRPLLFSALLLL.SGHTQASEWNDSQAVDKLFGAAGVKGTFVLYDVQRQCYVGHDRERAE
OXA-50-like_QLD71620.1_P.a.   MRPLLFSALLLL.SGHTQASEWNDSQAVDKLFGAAGVKGTFVLYDVQRQCYVGHDRERAE
OXA-50-like_QLD73935.1_P.a.   MRPLLFSALLLL.SGHTQASEWNDSQAVDKLFGAAGVKGTFVLYDVQRQCYVGHDRERAE
OXA-50-like_QLD78684.1_P.a.   MRPLLFSALLLL.SGHTQASEWNDSQAVDKLFGAAGVKGTFVLYDVQRQCYVGHDRERAE
OXA-50-like_QLF37576.1_P.a.   MRPLLFSALLLL.SGHTQASEWNDSQAVDKLFGAAGVKGTFVLYDVQRQCYVGHDRERAE
OXA-50-like_AWT32452.1_P.a.   MRPLLFSALLLL.SGHTQASEWNDSQAVDKLFGAAGVKGTFVLYDVQRQCYVGHDRERAE
OXA-50-like_AXZ92060.1_P.a.   MRPLLFSALLLL.SGHTQASEWNDSQAVDKLFGAAGVKGTFVLYDVQRQCYVGHDRERAE
OXA-50-like_QII97759.1_P.a.   MRPLLFSALLLL.SGHTQASEWNDSQAVDKLFGAAGVKGTFVLYDVQRQCYVGHDRERAE
OXA-50-like_QOE13519.1_P.a.   MRPLLFSALLLL.SGHTQASEWNDSQAVDKLFGAAGVKGTFVLYDVQRQCYVGHDRERAE
OXA-50-like_QPN23057.1_P.a.   MRPLLFSALLLL.SGHTQASEWNDSQAVDKLFGAAGVKGTFVLYDVQRQCYVGHDRERAE
OXA-50-like_QDR14302.1_P.a.   MRPLLFSALLLL.SGHTQASEWNDSQAVDKLFGAAGVKGTFVLYDVQRQCYVGHDRERAE
OXA-50-like_ALZ16475.1_P.a.   MRPLLFSALLLL.SGHTQASEWNDSQAVDKLFGAAGVKGTFVLYDVQRQCYVGHDRERAE
OXA-50-like_QKF04533.1_P.a.   MRPLLFSALLLL.SGHTQASEWNDSQAVDKLFGAAGVKGTFVLYDVQRQCYVGHDRERAE
OXA-50-like_QNO22317.1_P.a.   MRPLLFSALLLL.SGHTQASEWNDSQAVDKLFGAAGVKGTFVLYDVQRQCYVGHDRERAE
OXA-50-like_QQW12925.1_P.a.   MRPLLFSALLLL.SGHTQASEWNDSQAVDKLFGAAGVKGTFVLYDVQRQCYVGHDRERAE
OXA-50-like_AYZ81447.1_P.a.   MRPLLFSALLLL.SGHTQASEWNDSQAVDKLFGAAGVKGTFVLYDVQRQCYVGHDRERAE
OXA-50-like_QKL16662.1_P.a.   MRPLLFSALLLL.SGHTQASEWNDSQAVDKLFGAAGVKGTFVLYDVQRQCYVGHDRERAE
OXA-50-like_QQW00026.1_P.a.   MRPLLFSALLLL.SGHTQASEWNDSQAVDKLFGAAGVKGTFVLYDVQRQCYVGHDRERAE
OXA-50-like_ALV75159.1_P.a.   MRPLLLSALLLL.SGHTQASEWNDSQAVDKLFGAAGVKGTFVLYDVQRQRYVGHDRERAE
OXA-50-like_ALY89963.1_P.a.   MRPLLLSALLLL.SGHTQASEWNDSQAVDKLFGAAGVKGTFVLYDVQRQRYVGHDRERAE
OXA-50-like_ALY96872.1_P.a.   MRPLLLSALLLL.SGHTQASEWNDSQAVDKLFGAAGVKGTFVLYDVQRQRYVGHDRERAE
OXA-50-like_AMX88486.1_P.a.   MRPLLLSALLLL.SGHTQASEWNDSQAVDKLFGAAGVKGTFVLYDVQRQRYVGHDRERAE
OXA-50-like_ARC82831.1_P.a.   MRPLLLSALLLL.SGHTQASEWNDSQAVDKLFGAAGVKGTFVLYDVQRQRYVGHDRERAE
OXA-50-like_ASD14027.1_P.a.   MRPLLLSALLLL.SGHTQASEWNDSQAVDKLFGAAGVKGTFVLYDVQRQRYVGHDRERAE
OXA-50-like_AYZ75988.1_P.a.   MRPLLLSALLLL.SGHTQASEWNDSQAVDKLFGAAGVKGTFVLYDVQRQRYVGHDRERAE
OXA-50-like_QFZ61919.1_P.a.   MRPLLLSALLLL.SGHTQASEWNDSQAVDKLFGAAGVKGTFVLYDVQRQRYVGHDRERAE
OXA-50-like_QKP90385.1_P.a.   MRPLLLSALLLL.SGHTQASEWNDSQAVDKLFGAAGVKGTFVLYDVQRQRYVGHDRERAE
OXA-50-like_QKR47436.1_P.a.   MRPLLLSALLLL.SGHTQASEWNDSQAVDKLFGAAGVKGTFVLYDVQRQRYVGHDRERAE
OXA-50-like_QKR53224.1_P.a.   MRPLLLSALLLL.SGHTQASEWNDSQAVDKLFGAAGVKGTFVLYDVQRQRYVGHDRERAE
OXA-50-like_QKR59118.1_P.a.   MRPLLLSALLLL.SGHTQASEWNDSQAVDKLFGAAGVKGTFVLYDVQRQRYVGHDRERAE
OXA-50-like_QLF25267.1_P.a.   MRPLLLSALLLL.SGHTQASEWNDSQAVDKLFGAAGVKGTFVLYDVQRQRYVGHDRERAE
OXA-50-like_QPP21637.1_P.a.   MRPLLLSALLLL.SGHTQASEWNDSQAVDKLFGAAGVKGTFVLYDVQRQRYVGHDRERAE
OXA-50-like_QQA85373.1_P.a.   MRPLLLSALLLL.SGHTQASEWNDSQAVDKLFGAAGVKGTFVLYDVQRQRYVGHDRERAE
OXA-50-like_QRG72678.1_P.a.   MRPLLLSALLLL.SGHTQASEWNDSQAVDKLFGAAGVKGTFVLYDVQRQRYVGHDRERAE
OXA-50-like_AYY40220.1_P.a.   MRPLLLSALLLL.SGHTQASEWNDSQAVDKLFGAAGVKGTFVLYDVQRQRYVGHDRERAE
OXA-50-like_ALZ28576.1_P.a.   MRPLLFSALLLL.SGHTQASEWNDSQAVDKLFGAAGVKGTFVLYDVQRQRYVGHDRERAE
OXA-50-like_QNQ04301.1_P.a.   MRPLLFSALLLL.SGHTQASEWNDSQAVDKLFGAAGVKGTFVLYDVQRQRYVGHDRERAE
OXA-50-like_ALZ94285.1_P.a.   MRPLLFSALLLL.SGHTQASEWNDSQAVDKLFGAAGVKGTFVLYDVQRQRYVGHDRERAE
OXA-50-like_AMU04106.1_P.a.   MRPLLFSALLLL.SGHTQASEWNDSQAVDKLFGAAGVKGTFVLYDVQRQRYVGHDRERAE
OXA-50-like_AOT37986.1_P.a.   MRPLLFSALLLL.SGHTQASEWNDSQAVDKLFGAAGVKGTFVLYDVQRQRYVGHDRERAE
OXA-50-like_ASD20357.1_P.a.   MRPLLFSALLLL.SGHTQASEWNDSQAVDKLFGAAGVKGTFVLYDVQRQRYVGHDRERAE
OXA-50-like_AVZ22774.1_P.a.   MRPLLFSALLLL.SGHTQASEWNDSQAVDKLFGAAGVKGTFVLYDVQRQRYVGHDRERAE
OXA-50-like_AXC20842.1_P.a.   MRPLLFSALLLL.SGHTQASEWNDSQAVDKLFGAAGVKGTFVLYDVQRQRYVGHDRERAE
OXA-50-like_AYN84711.1_P.a.   MRPLLFSALLLL.SGHTQASEWNDSQAVDKLFGAAGVKGTFVLYDVQRQRYVGHDRERAE
OXA-50-like_CRZ33166.1_P.a.   MRPLLFSALLLL.SGHTQASEWNDSQAVDKLFGAAGVKGTFVLYDVQRQRYVGHDRERAE
OXA-50-like_QBF65101.1_P.a.   MRPLLFSALLLL.SGHTQASEWNDSQAVDKLFGAAGVKGTFVLYDVQRQRYVGHDRERAE
OXA-50-like_QEF81401.1_P.a.   MRPLLFSALLLL.SGHTQASEWNDSQAVDKLFGAAGVKGTFVLYDVQRQRYVGHDRERAE
OXA-50-like_QEF87414.1_P.a.   MRPLLFSALLLL.SGHTQASEWNDSQAVDKLFGAAGVKGTFVLYDVQRQRYVGHDRERAE
OXA-50-like_QEF93418.1_P.a.   MRPLLFSALLLL.SGHTQASEWNDSQAVDKLFGAAGVKGTFVLYDVQRQRYVGHDRERAE
OXA-50-like_QGM44151.1_P.a.   MRPLLFSALLLL.SGHTQASEWNDSQAVDKLFGAAGVKGTFVLYDVQRQRYVGHDRERAE
OXA-50-like_QHI63098.1_P.a.   MRPLLFSALLLL.SGHTQASEWNDSQAVDKLFGAAGVKGTFVLYDVQRQRYVGHDRERAE
OXA-50-like_QIB85902.1_P.a.   MRPLLFSALLLL.SGHTQASEWNDSQAVDKLFGAAGVKGTFVLYDVQRQRYVGHDRERAE
OXA-50-like_QKR06746.1_P.a.   MRPLLFSALLLL.SGHTQASEWNDSQAVDKLFGAAGVKGTFVLYDVQRQRYVGHDRERAE
OXA-50-like_QKR13035.1_P.a.   MRPLLFSALLLL.SGHTQASEWNDSQAVDKLFGAAGVKGTFVLYDVQRQRYVGHDRERAE
OXA-50-like_QKR18855.1_P.a.   MRPLLFSALLLL.SGHTQASEWNDSQAVDKLFGAAGVKGTFVLYDVQRQRYVGHDRERAE
OXA-50-like_QKR83750.1_P.a.   MRPLLFSALLLL.SGHTQASEWNDSQAVDKLFGAAGVKGTFVLYDVQRQRYVGHDRERAE
OXA-50-like_QLF31591.1_P.a.   MRPLLFSALLLL.SGHTQASEWNDSQAVDKLFGAAGVKGTFVLYDVQRQRYVGHDRERAE
OXA-50-like_VDL04843.1_P.a.   MRPLLFSALLLL.SGHTQASEWNDSQAVDKLFGAAGVKGTFVLYDVQRQRYVGHDRERAE
OXA-50-like_QCP80115.1_P.a.   MRPLLFSALLLL.SGHTQASEWNDSQAVDKLFGAAGVKGTFVLYDVQRQRYVGHDRERAE
OXA-50-like_QEK88934.1_P.a.   MRPLLFSALLLL.SGHTQASEWNDSQAVDKLFGAAGVKGTFVLYDVQRQRYVGHDRERAE
OXA-50-like_ALU47404.1_P.a.   MRPLLFSALLLL.SGHTQASEWNDSQAVDKLFGAAGVKGTFVLYDVQRQRYVGHDRERAE
OXA-50-like_APC75958.1_P.a.   MRPLLFSALLLL.SGHTQASEWNDSQAVDKLFGAAGVKGTFVLYDVQRQRYVGHDRERAE
OXA-50-like_QPZ59753.1_P.a.   MRPLLFSALLLL.SGHTQASEWNDSQAVDKLFGAAGVKGTFVLYDVQRQRYVGHDRERAE
OXA-50-like_QFZ03142.1_P.a.   MRPLLFSALLLL.SGHTQASEWNDSQAVDKLFGAAGVKGTFVLYDVQRQRYVGHDRERAE
OXA-50-like_QGG01915.1_P.a.   MRPLLFNALLLL.SGHTQASEWNDSQAVDKLFGAAGVKGTFVLYDVQRQRYVGHDRERAE
OXA-50-like_AUA86524.1_P.a.   MRPLLFSALLLL.SGHTQASEWNDSQAVDKLFGAAGVKGTFVLYDVQRQRYVGHDRERAE
OXA-50-like_AUA92615.1_P.a.   MRPLLFSALLLL.SGHTQASEWNDSQAVDKLFGAAGVKGTFVLYDVQRQRYVGHDRERAE
4JF4_Beta-lactamase           ..............QIVQGHN.QVIHQYFDEKNTSGVLVIQTDKKINLYGNALSRAN
                     acc
```

OXA-50-like subfamily

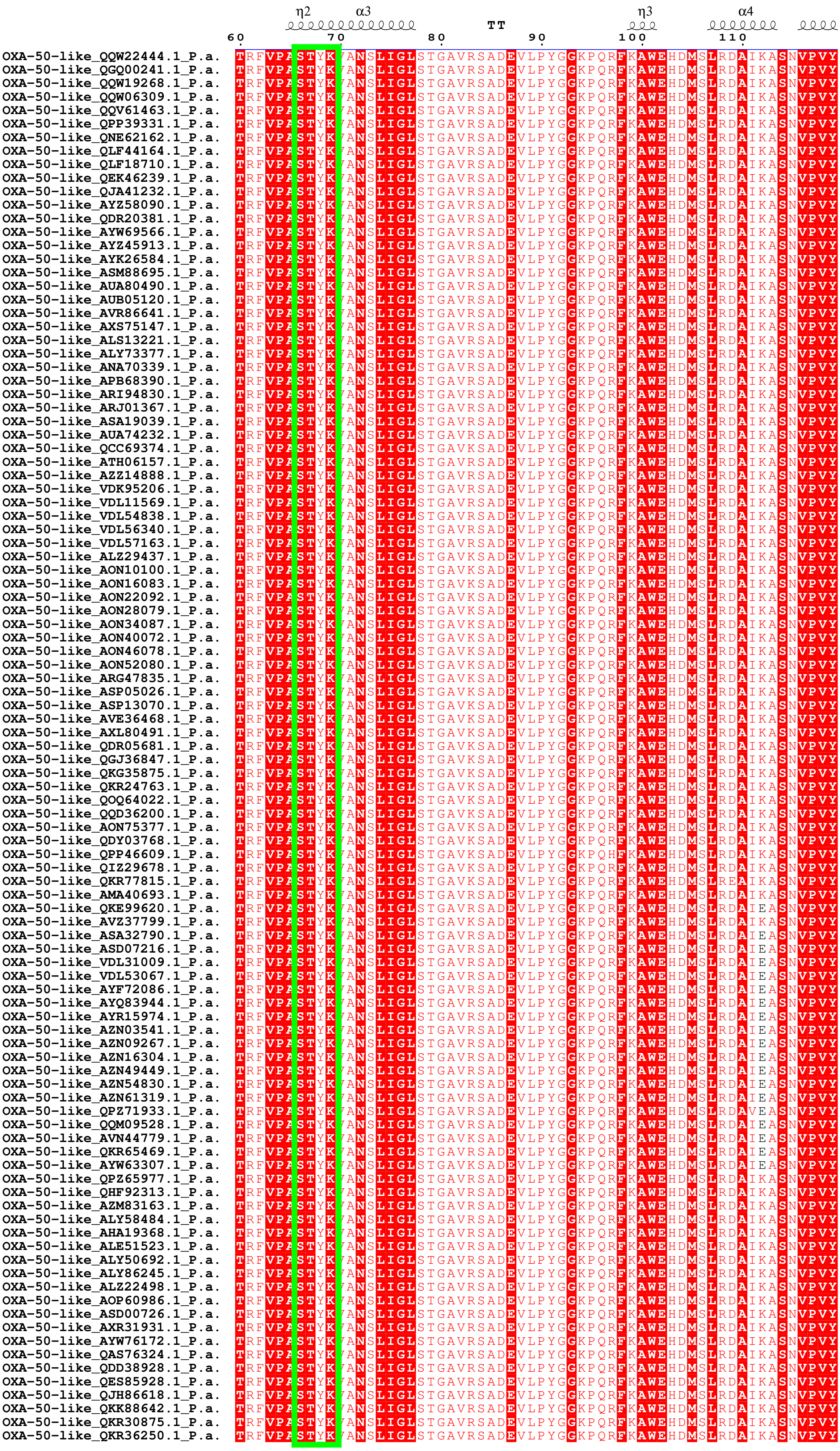

OXA-50-like subfamily

```
OXA-50-like_QKS42441.1_P.a.  TRFVPASTYKVANSLIGLSTGAVRSADEVLPYGGKPQRFKAWEHDMSLRDAIKASNVPVY
OXA-50-like_QPP27676.1_P.a.  TRFVPASTYKVANSLIGLSTGAVRSADEVLPYGGKPQRFKAWEHDMSLRDAIKASNVPVY
OXA-50-like_QPV54155.1_P.a.  TRFVPASTYKVANSLIGLSTGAVRSADEVLPYGGKPQRFKAWEHDMSLRDAIKASNVPVY
OXA-50-like_QPZ84995.1_P.a.  TRFVPASTYKVANSLIGLSTGAVRSADEVLPYGGKPQRFKAWEHDMSLRDAIKASNVPVY
OXA-50-like_ANP59968.1_P.a.  TRFVPASTYKVANSLIGLSTGAVRSADEVLPYGGKPQRFKAWEHDMSLRDAIKASNVPVY
OXA-50-like_QGQ06905.1_P.a.  TRFVPASTYKVANSLIGLSTGAVRSADEVLPYGGKPQRFKAWEHDMSLRDAIKASNVPVY
OXA-50-like_AXR14368.1_P.a.  TRFVPASTYKVANSLIGLSTGAVRSADEVLPYGGKPQRFKAWEHDMSLRDAIKASNVPVY
OXA-50-like_ALP57413.1_P.a.  TRFVPASTYKVANSLIGLSTGAVRSADEVLPYGGKPQRFKAWEHDMSLREAIKASNVPVY
OXA-50-like_QKR41777.1_P.a.  TRFVPASTYKVANSLIGLSTGAVRSADEVLPYGGKPQRFKAWEHDMSLREAIKASNVPVY
OXA-50-like_ALY36156.1_P.a.  TRFVPASTYKVANSLIGLSTGAVRSADEVLPYGGKPQRFKAWEHDMSLREAIKASNVPVY
OXA-50-like_ALY52550.1_P.a.  TRFVPASTYKVANSLIGLSTGAVRSADEVLPYGGKPQRFKAWEHDMSLREAIKASNVPVY
OXA-50-like_ANI07822.1_P.a.  TRFVPASTYKVANSLIGLSTGAVRSADEVLPYGGKPQRFKAWEHDMSLREAIKASNVPVY
OXA-50-like_AWS88163.1_P.a.  TRFVPASTYKVANSLIGLSTGAVRSADEVLPYGGKPQRFKAWEHDMSLREAIKASNVPVY
OXA-50-like_AXN29478.1_P.a.  TRFVPASTYKVANSLIGLSTGAVRSADEVLPYGGKPQRFKAWEHDMSLREAIKASNVPVY
OXA-50-like_QBC08854.1_P.a.  TRFVPASTYKVANSLIGLSTGAVRSADEVLPYGGKPQRFKAWEHDMSLREAIKASNVPVY
OXA-50-like_QDD49249.1_P.a.  TRFVPASTYKVANSLIGLSTGAVRSADEVLPYGGKPQRFKAWEHDMSLREAIKASNVPVY
OXA-50-like_QDR11871.1_P.a.  TRFVPASTYKVANSLIGLSTGAVRSADEVLPYGGKPQRFKAWEHDMSLREAIKASNVPVY
OXA-50-like_QKP84324.1_P.a.  TRFVPASTYKVANSLIGLSTGAVRSADEVLPYGGKPQRFKAWEHDMSLREAIKASNVPVY
OXA-50-like_QKR71283.1_P.a.  TRFVPASTYKVANSLIGLSTGAVRSADEVLPYGGKPQRFKAWEHDMSLREAIKASNVPVY
OXA-50-like_QLA53973.1_P.a.  TRFVPASTYKVANSLIGLSTGAVRSADEVLPYGGKPQRFKAWEHDMSLREAIKASNVPVY
OXA-50-like_QLD71620.1_P.a.  TRFVPASTYKVANSLIGLSTGAVRSADEVLPYGGKPQRFKAWEHDMSLREAIKASNVPVY
OXA-50-like_QLD73935.1_P.a.  TRFVPASTYKVANSLIGLSTGAVRSADEVLPYGGKPQRFKAWEHDMSLREAIKASNVPVY
OXA-50-like_QLD78684.1_P.a.  TRFVPASTYKVANSLIGLSTGAVRSADEVLPYGGKPQRFKAWEHDMSLREAIKASNVPVY
OXA-50-like_QLF37576.1_P.a.  TRFVPASTYKVANSLIGLSTGAVRSADEVLPYGGKPQRFKAWEHDMSLREAIKASNVPVY
OXA-50-like_AWT32452.1_P.a.  TRFVPASTYKVANSLIGLSTGAVRSADEVLPYGGKPQRFKAWEHDMSLREAIKASNVPVY
OXA-50-like_AXZ92060.1_P.a.  TRFVPASTYKVANSLIGLSTGAVRSADEVLPYGGKPQRFKAWEHDMSLREAIKASNVPVY
OXA-50-like_QII97759.1_P.a.  TRFVPASTYKVANSLIGLSTGAVRSADEVLPYGGKPQRFKAWEHDMSLREAIKASNVPVY
OXA-50-like_QOE13519.1_P.a.  TRFVPASTYKVANSLIGLSTGAVRSADEVLPYGGKPQRFKAWEHDMSLREAIKASNVPVY
OXA-50-like_QPN23057.1_P.a.  TRFVPASTYKVANSLIGLSTGAVRSADEVLPYGGKPQRFKAWEHDMSLREAIKASNVPVY
OXA-50-like_QDR14302.1_P.a.  TRFVPASTYKVANSLIGLSTGAVRSADEVLPYGGKPQRFKAWEHDMSLREAIKASNVPVY
OXA-50-like_ALZ16475.1_P.a.  TRFVPASTYKVANSLIGLSTGAVRSADEVLPYGGKPQRFKAWEHDMSLREAIKASNVPVY
OXA-50-like_QKF04533.1_P.a.  TRFVPASTYKVANSLIGLSTGAVRSADEVLPYGGKPQRFKAWEHDMSLREAIKASNVPVY
OXA-50-like_QNO22317.1_P.a.  TRFVPASTYKVANSLIGLSTGAVRSADEVLPYGGKPQRFKAWEHDMSLREAIKASNVPVY
OXA-50-like_QQW12925.1_P.a.  TRFVPASTYKVANSLIGLSTGAVRSADEVLPYGGKPQRFKAWEHDMSLREAIKASNVPVY
OXA-50-like_AYZ81447.1_P.a.  TRFVPASTYKVANSLIGLSTGAVRSADEVLPYGGKPQRFKAWEHDMSLREAIKASNVPVY
OXA-50-like_QKL16662.1_P.a.  TRFVPASTYKVANSLIGLSTGAVRSADEVLPYGGKPQRFKAWEHDMSLREAIKASNVPVY
OXA-50-like_QQW00026.1_P.a.  TRFVPASTYKVANSLIGLSTGAVRSADEVLPYGGKPQRFKAWEHDMSLREAIKASNVPVY
OXA-50-like_ALV75159.1_P.a.  TRFVPASTYKVANSLIGLSTGAVRSADEVLPYGGKPQRFKAWEHDMSLREAIKASNVPVY
OXA-50-like_ALY89963.1_P.a.  TRFVPASTYKVANSLIGLSTGAVRSADEVLPYGGKPQRFKAWEHDMSLREAIKASNVPVY
OXA-50-like_ALY96872.1_P.a.  TRFVPASTYKVANSLIGLSTGAVRSADEVLPYGGKPQRFKAWEHDMSLREAIKASNVPVY
OXA-50-like_AMX88486.1_P.a.  TRFVPASTYKVANSLIGLSTGAVRSADEVLPYGGKPQRFKAWEHDMSLREAIKASNVPVY
OXA-50-like_ARC82831.1_P.a.  TRFVPASTYKVANSLIGLSTGAVRSADEVLPYGGKPQRFKAWEHDMSLREAIKASNVPVY
OXA-50-like_ASD14027.1_P.a.  TRFVPASTYKVANSLIGLSTGAVRSADEVLPYGGKPQRFKAWEHDMSLREAIKASNVPVY
OXA-50-like_AYZ75988.1_P.a.  TRFVPASTYKVANSLIGLSTGAVRSADEVLPYGGKPQRFKAWEHDMSLREAIKASNVPVY
OXA-50-like_QFZ61919.1_P.a.  TRFVPASTYKVANSLIGLSTGAVRSADEVLPYGGKPQRFKAWEHDMSLREAIKASNVPVY
OXA-50-like_QKP90385.1_P.a.  TRFVPASTYKVANSLIGLSTGAVRSADEVLPYGGKPQRFKAWEHDMSLREAIKASNVPVY
OXA-50-like_QKR47436.1_P.a.  TRFVPASTYKVANSLIGLSTGAVRSADEVLPYGGKPQRFKAWEHDMSLREAIKASNVPVY
OXA-50-like_QKR53224.1_P.a.  TRFVPASTYKVANSLIGLSTGAVRSADEVLPYGGKPQRFKAWEHDMSLREAIKASNVPVY
OXA-50-like_QKR59118.1_P.a.  TRFVPASTYKVANSLIGLSTGAVRSADEVLPYGGKPQRFKAWEHDMSLREAIKASNVPVY
OXA-50-like_QLF25267.1_P.a.  TRFVPASTYKVANSLIGLSTGAVRSADEVLPYGGKPQRFKAWEHDMSLREAIKASNVPVY
OXA-50-like_QPP21637.1_P.a.  TRFVPASTYKVANSLIGLSTGAVRSADEVLPYGGKPQRFKAWEHDMSLREAIKASNVPVY
OXA-50-like_QQA85373.1_P.a.  TRFVPASTYKVANSLIGLSTGAVRSADEVLPYGGKPQRFKAWEHDMSLREAIKASNVPVY
OXA-50-like_QRG72678.1_P.a.  TRFVPASTYKVANSLIGLSTGAVRSADEVLPYGGKPQRFKAWEHDMSLREAIKASNVPVY
OXA-50-like_AYY40220.1_P.a.  TRFVPASTYKVANSLIGLSTGAVRSADEVLPYGGKPQRFKAWEHDMSLREAIKASNVPVY
OXA-50-like_ALZ28576.1_P.a.  TRFVPASTYKVANSLIGLSTGAVRSADEVLPYGGKPQRFKAWEHDMSLREAIKASNVPVY
OXA-50-like_QNQ04301.1_P.a.  TRFVPASTYKVANSLIGLSTGAVRSADEVLPYGGKPQRFKAWEHVMSLREAIKASNVPVY
OXA-50-like_ALZ94285.1_P.a.  TRFVPASTYKVANSLIGLSTGAVRSADEVLPYGGKPQRFKAWEHDMSLREAIKASNVPVY
OXA-50-like_AMU04106.1_P.a.  TRFVPASTYKVANSLIGLSTGAVRSADEVLPYGGKPQRFKAWEHDMSLREAIKASNVPVY
OXA-50-like_AOT37986.1_P.a.  TRFVPASTYKVANSLIGLSTGAVRSADEVLPYGGKPQRFKAWEHDMSLREAIKASNVPVY
OXA-50-like_ASD20357.1_P.a.  TRFVPASTYKVANSLIGLSTGAVRSADEVLPYGGKPQRFKAWEHDMSLREAIKASNVPVY
OXA-50-like_AVZ22774.1_P.a.  TRFVPASTYKVANSLIGLSTGAVRSADEVLPYGGKPQRFKAWEHDMSLREAIKASNVPVY
OXA-50-like_AXC20842.1_P.a.  TRFVPASTYKVANSLIGLSTGAVRSADEVLPYGGKPQRFKAWEHDMSLREAIKASNVPVY
OXA-50-like_AYN84711.1_P.a.  TRFVPASTYKVANSLIGLSTGAVRSADEVLPYGGKPQRFKAWEHDMSLREAIKASNVPVY
OXA-50-like_CRZ33166.1_P.a.  TRFVPASTYKVANSLIGLSTGAVRSADEVLPYGGKPQRFKAWEHDMSLREAIKASNVPVY
OXA-50-like_QBF65101.1_P.a.  TRFVPASTYKVANSLIGLSTGAVRSADEVLPYGGKPQRFKAWEHDMSLREAIKASNVPVY
OXA-50-like_QEF81401.1_P.a.  TRFVPASTYKVANSLIGLSTGAVRSADEVLPYGGKPQRFKAWEHDMSLREAIKASNVPVY
OXA-50-like_QEF87414.1_P.a.  TRFVPASTYKVANSLIGLSTGAVRSADEVLPYGGKPQRFKAWEHDMSLREAIKASNVPVY
OXA-50-like_QEF93418.1_P.a.  TRFVPASTYKVANSLIGLSTGAVRSADEVLPYGGKPQRFKAWEHDMSLREAIKASNVPVY
OXA-50-like_QGM44151.1_P.a.  TRFVPASTYKVANSLIGLSTGAVRSADEVLPYGGKPQRFKAWEHDMSLREAIKASNVPVY
OXA-50-like_QHI63098.1_P.a.  TRFVPASTYKVANSLIGLSTGAVRSADEVLPYGGKPQRFKAWEHDMSLREAIKASNVPVY
OXA-50-like_QIB85902.1_P.a.  TRFVPASTYKVANSLIGLSTGAVRSADEVLPYGGKPQRFKAWEHDMSLREAIKASNVPVY
OXA-50-like_QKR06746.1_P.a.  TRFVPASTYKVANSLIGLSTGAVRSADEVLPYGGKPQRFKAWEHDMSLREAIKASNVPVY
OXA-50-like_QKR13035.1_P.a.  TRFVPASTYKVANSLIGLSTGAVRSADEVLPYGGKPQRFKAWEHDMSLREAIKASNVPVY
OXA-50-like_QKR18855.1_P.a.  TRFVPASTYKVANSLIGLSTGAVRSADEVLPYGGKPQRFKAWEHDMSLREAIKASNVPVY
OXA-50-like_QKR83750.1_P.a.  TRFVPASTYKVANSLIGLSTGAVRSADEVLPYGGKPQRFKAWEHDMSLREAIKASNVPVY
OXA-50-like_QLF31591.1_P.a.  TRFVPASTYKVANSLIGLSTGAVRSADEVLPYGGKPQRFKAWEHDMSLREAIKASNVPVY
OXA-50-like_VDL04843.1_P.a.  TRFVPASTYKVANSLIGLSTGAVRSADEVLPYGGKPQRFKAWEHDMSLREAIKASNVPVY
OXA-50-like_QCP80115.1_P.a.  TRFVPASTYKVANSLIGLSTGAVRSADEVLPYGGKPQRFKAWEHDMSLREAIKASNVPVY
OXA-50-like_QEK88934.1_P.a.  TRFVPASTYKVANSLIGLSTGAVRSADEVLPYGGKPQRFKAWEHDMSLREAIKASNVPVY
OXA-50-like_ALU47404.1_P.a.  TRFVPASTYKVANSLIGLSTGAVRSADEVLPYGGKPQRFKAWEHDMSLREAIKASNVPVY
OXA-50-like_APC75958.1_P.a.  TRFVPASTYKVANSLIGLSTGAVRSADEVLPYGGKPQRFKAWEHDMSLREAIKASNVPVY
OXA-50-like_QPZ59753.1_P.a.  TRFVPASTYKVANSLIGLSTGAVRSADEVLPYGGKPQRFKAWEHDMSLREAIKASNVPVY
OXA-50-like_QFZ03142.1_P.a.  TRFVPASTYKVANSLIGLSTGAVRSADEVLPYGGKPQRFKAWEHDMSLREAIKASNVPVY
OXA-50-like_QGG01915.1_P.a.  TRFVPASTYKVANSLIGLSTGAVRSADEVLPYGGKPQRFKAWEHDMSLREAIKASNVPVY
OXA-50-like_AUA86524.1_P.a.  TRFVPASTYKVANSLIGLSTGAVRSADEVLPYGGKPQRFKAWEHDMSLRDAIKASNVPVY
OXA-50-like_AUA92615.1_P.a.  TRFVPASTYKVANSLIGLSTGAVRSADEVLPYGGKPQRFKAWEHDMSLRDAIKASNVPVY
4JF4_Beta-lactamase          TEYVPASTFKMLNALIGLEN.QKTDINEIFKWKGEKRSFTAWEKDMTLGEAMKLSAVPVY
                        acc
```

OXA-50-like subfamily

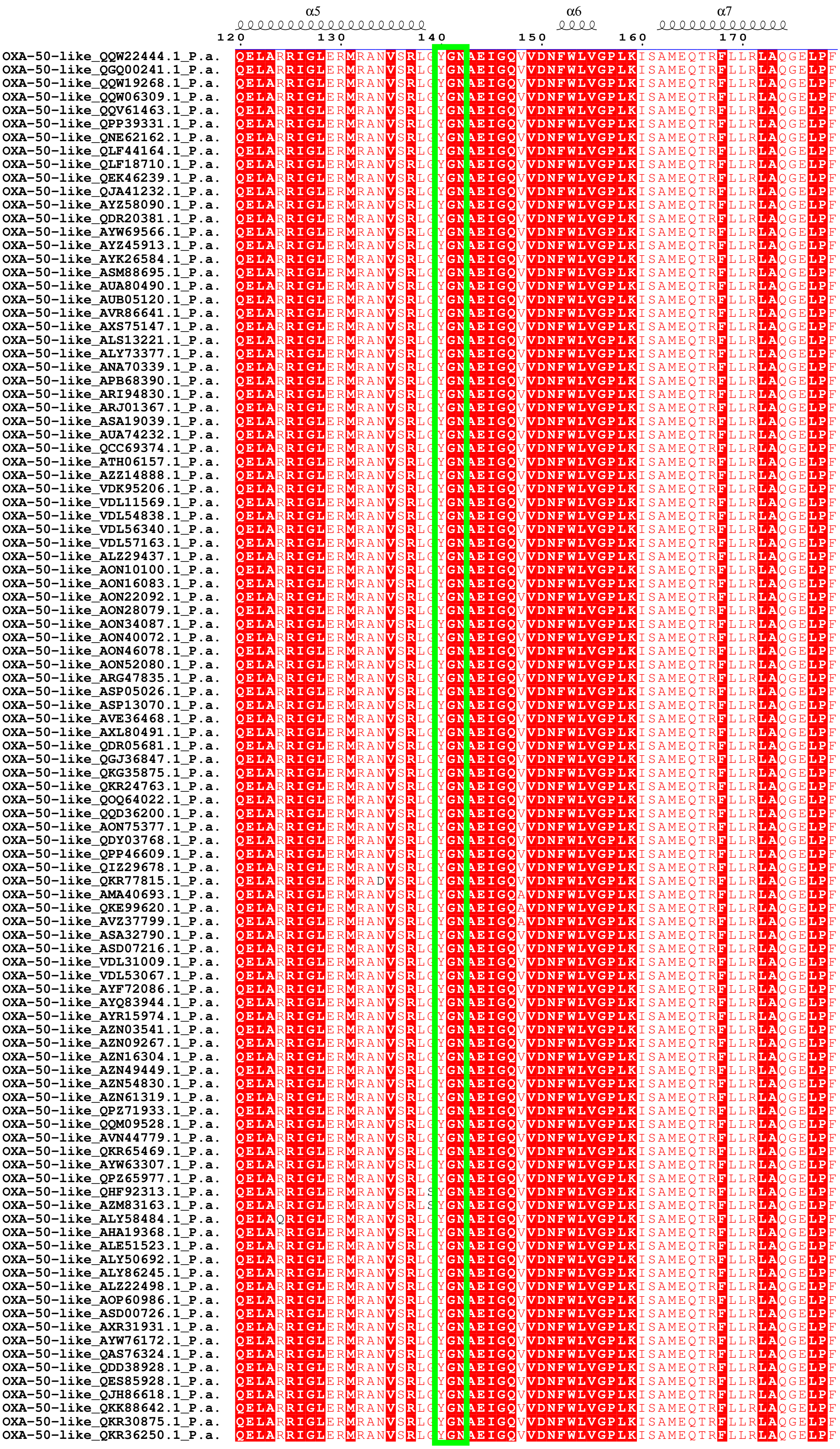

OXA-50-like subfamily

```
OXA-50-like_QKS42441.1_P.a.  QELARRIGLERMRANVSRLGYGNAEIGQVVDNFWLVGPLKISAMEQTRFLLRLAQGELPF
OXA-50-like_QPP27676.1_P.a.  QELARRIGLERMRANVSRLGYGNAEIGQVVDNFWLVGPLKISAMEQTRFLLRLAQGELPF
OXA-50-like_QPV54155.1_P.a.  QELARRIGLERMRANVSRLGYGNAEIGQVVDNFWLVGPLKISAMEQTRFLLRLAQGELPF
OXA-50-like_QPZ84995.1_P.a.  QELARRIGLERMRANVSRLGYGNAEIGQVVDNFWLVGPLKISAMEQTRFLLRLAQGELPF
OXA-50-like_ANP59968.1_P.a.  QELARRIGLERMRANVSRLGYGNAEIGQVVDNFWLVGPLKISAMEQTRFLLRLAQGELPF
OXA-50-like_QGQ06905.1_P.a.  QELARRIGLERMRANVSRLGYGNAEIGQVVDNFWLVGPLKISAMEQTRFLLRLAQGELPF
OXA-50-like_AXR14368.1_P.a.  QELARRIGLERMRANVSRLGYGNAEIGQVVDNFWLVGPLKISAMEQTRFLLRLAQGELPF
OXA-50-like_ALP57413.1_P.a.  QELARRIGLERMRANVSRLGYGNAEIGQVVDNFWLVGPLKISAMEQTRFLLRLAQGELPF
OXA-50-like_QKR41777.1_P.a.  QELARRIGLERMRANVSRLGYGNAEIGQVVDNFWLVGPLKISAMEQTRFLLRLAQGELPL
OXA-50-like_ALY36156.1_P.a.  QELARRIGLERMRANVSRLGYGNAEIGQVVDNFWLVGPLKISAMEQTRFLLRLAQGELPF
OXA-50-like_ALY52550.1_P.a.  QELARRIGLERMRANVSRLGYGNAEIGQVVDNFWLVGPLKISAMEQTRFLLRLAQGELPF
OXA-50-like_ANI07822.1_P.a.  QELARRIGLERMRANVSRLGYGNAEIGQVVDNFWLVGPLKISAMEQTRFLLRLAQGELPF
OXA-50-like_AWS88163.1_P.a.  QELARRIGLERMRANVSRLGYGNAEIGQVVDNFWLVGPLKISAMEQTRFLLRLAQGELPF
OXA-50-like_AXN29478.1_P.a.  QELARRIGLERMRANVSRLGYGNAEIGQVVDNFWLVGPLKISAMEQTRFLLRLAQGELPF
OXA-50-like_QBC08854.1_P.a.  QELARRIGLERMRANVSRLGYGNAEIGQVVDNFWLVGPLKISAMEQTRFLLRLAQGELPF
OXA-50-like_QDD49249.1_P.a.  QELARRIGLERMRANVSRLGYGNAEIGQVVDNFWLVGPLKISAMEQTRFLLRLAQGELPF
OXA-50-like_QDR11871.1_P.a.  QELARRIGLERMRANVSRLGYGNAEIGQVVDNFWLVGPLKISAMEQTRFLLRLAQGELPF
OXA-50-like_QKP84324.1_P.a.  QELARRIGLERMRANVSRLGYGNAEIGQVVDNFWLVGPLKISAMEQTRFLLRLAQGELPF
OXA-50-like_QKR71283.1_P.a.  QELARRIGLERMRANVSRLGYGNAEIGQVVDNFWLVGPLKISAMEQTRFLLRLAQGELPF
OXA-50-like_QLA53973.1_P.a.  QELARRIGLERMRANVSRLGYGNAEIGQVVDNFWLVGPLKISAMEQTRFLLRLAQGELPF
OXA-50-like_QLD71620.1_P.a.  QELARRIGLERMRANVSRLGYGNAEIGQVVDNFWLVGPLKISAMEQTRFLLRLAQGELPF
OXA-50-like_QLD73935.1_P.a.  QELARRIGLERMRANVSRLGYGNAEIGQVVDNFWLVGPLKISAMEQTRFLLRLAQGELPF
OXA-50-like_QLD78684.1_P.a.  QELARRIGLERMRANVSRLGYGNAEIGQVVDNFWLVGPLKISAMEQTRFLLRLAQGELPF
OXA-50-like_QLF37576.1_P.a.  QELARRIGLERMRANVSRLGYGNAEIGQVVDNFWLVGPLKISAMEQTRFLLRLAQGELPF
OXA-50-like_AWT32452.1_P.a.  QELARRIGLERMRANVSRLGYGNAEIGQVVDNFWLVGPLKISAMEQTRFLLRLAQGELPF
OXA-50-like_AXZ92060.1_P.a.  QELARRIGLERMRANVSRLGYGNAEIGQVVDNFWLVGPLKISAMEQTRFLLRLAQGELPF
OXA-50-like_QII97759.1_P.a.  QELARRIGLERMRANVSRLGYGNAEIGQVVDNFWLVGPLKISAMEQTRFLLRLAQGELPF
OXA-50-like_QOE13519.1_P.a.  QELARRIGLERMRANVSRLGYGNAEIGQVVDNFWLVGPLKISAMEQTRFLLRLAQGELPF
OXA-50-like_QPN23057.1_P.a.  QELARRIGLERMRANVSRLGYGNAEIGQVVDNFWLVGPLKISAMEQTRFLLRLAQGELPF
OXA-50-like_QDR14302.1_P.a.  QELARRIGLERMRANVSRLGYGNAEIGQVVDNFWLVGPLKISAMEQTRFLIRLAQGELPF
OXA-50-like_ALZ16475.1_P.a.  QELARRIGLERMRANVSRLGYGNAEIGQVVDNFWLVGPLKISAMEQTHFLLRLAQGELPF
OXA-50-like_QKF04533.1_P.a.  QELARRIGLERMRANVSRLGYGNAEIGQVVDNFWLVGPLKISAMEQTHFLLRLAQGELPF
OXA-50-like_QNO22317.1_P.a.  QELARRIGLERMRANVSRLGYGNAEIGQVVDNFWLVGPLKISAMEQTHFLLRLAQGELPF
OXA-50-like_QQW12925.1_P.a.  QELARRIGLERMRANVSRLGYGNAEIGQVVDNFWLVGPLKISAMEQTHFLLRLAQGELPF
OXA-50-like_AYZ81447.1_P.a.  QELARRIGLERMRANVSRLGYGNAEIGQVVDNFWLVGPLKISAMEQTHFLLRLAQGELPF
OXA-50-like_QKL16662.1_P.a.  QELARRIGLERMRGNVSRLGYGNAEIGQVVDNFWLVGPLKISAMEQTHFLLRLAQGELPF
OXA-50-like_QQW00026.1_P.a.  QELARRIGLERMRGNVSRLGYGNAEIGQVVDNFWLVGPLKISAMEQTHFLLRLAQGELPF
OXA-50-like_ALV75159.1_P.a.  QELARRIGLERMRANVSRLGYGNAEIGQVVDNFWLVGPLKISAMEQTHFLLRLAQGELPF
OXA-50-like_ALY89963.1_P.a.  QELARRIGLERMRANVSRLGYGNAEIGQVVDNFWLVGPLKISAMEQTHFLLRLAQGELPF
OXA-50-like_ALY96872.1_P.a.  QELARRIGLERMRANVSRLGYGNAEIGQVVDNFWLVGPLKISAMEQTHFLLRLAQGELPF
OXA-50-like_AMX88486.1_P.a.  QELARRIGLERMRANVSRLGYGNAEIGQVVDNFWLVGPLKISAMEQTHFLLRLAQGELPF
OXA-50-like_ARC82831.1_P.a.  QELARRIGLERMRANVSRLGYGNAEIGQVVDNFWLVGPLKISAMEQTHFLLRLAQGELPF
OXA-50-like_ASD14027.1_P.a.  QELARRIGLERMRANVSRLGYGNAEIGQVVDNFWLVGPLKISAMEQTHFLLRLAQGELPF
OXA-50-like_AYZ75988.1_P.a.  QELARRIGLERMRANVSRLGYGNAEIGQVVDNFWLVGPLKISAMEQTHFLLRLAQGELPF
OXA-50-like_QFZ61919.1_P.a.  QELARRIGLERMRANVSRLGYGNAEIGQVVDNFWLVGPLKISAMEQTHFLLRLAQGELPF
OXA-50-like_QKP90385.1_P.a.  QELARRIGLERMRANVSRLGYGNAEIGQVVDNFWLVGPLKISAMEQTHFLLRLAQGELPF
OXA-50-like_QKR47436.1_P.a.  QELARRIGLERMRANVSRLGYGNAEIGQVVDNFWLVGPLKISAMEQTHFLLRLAQGELPF
OXA-50-like_QKR53224.1_P.a.  QELARRIGLERMRANVSRLGYGNAEIGQVVDNFWLVGPLKISAMEQTHFLLRLAQGELPF
OXA-50-like_QKR59118.1_P.a.  QELARRIGLERMRANVSRLGYGNAEIGQVVDNFWLVGPLKISAMEQTHFLLRLAQGELPF
OXA-50-like_QLF25267.1_P.a.  QELARRIGLERMRANVSRLGYGNAEIGQVVDNFWLVGPLKISAMEQTHFLLRLAQGELPF
OXA-50-like_QPP21637.1_P.a.  QELARRIGLERMRANVSRLGYGNAEIGQVVDNFWLVGPLKISAMEQTHFLLRLAQGELPF
OXA-50-like_QQA85373.1_P.a.  QELARRIGLERMRANVSRLGYGNAEIGQVVDNFWLVGPLKISAMEQTHFLLRLAQGELPF
OXA-50-like_QRG72678.1_P.a.  QELARRIGLERMRANVSRLGYGNAEIGQVVDNFWLVGPLKISAMEQTHFLLRLAQGELPF
OXA-50-like_AYY40220.1_P.a.  QELARRIGLERMRANVSRLGYGNAEIGQVVDNFWLVGPLKISAMEQTHFLLRLAQGELPF
OXA-50-like_ALZ28576.1_P.a.  QELARRIGLERMRANVSRLGYGNAEIGQVVDNFWLVGPLKISAMEQTHFLLRLAQGELPF
OXA-50-like_QNQ04301.1_P.a.  QELARRIGLERMRANVSRLGYGNAEIGQVVDNFWLVGPLKISAMEQTHFLLRLAQGELPF
OXA-50-like_ALZ94285.1_P.a.  QELARRIGLERMRANVSRLGYGNAEIGQVVDNFWLVGPLKISAMEQTHFLLRLAQGELPF
OXA-50-like_AMU04106.1_P.a.  QELARRIGLERMRANVSRLGYGNAEIGQVVDNFWLVGPLKISAMEQTHFLLRLAQGELPF
OXA-50-like_AOT37986.1_P.a.  QELARRIGLERMRANVSRLGYGNAEIGQVVDNFWLVGPLKISAMEQTHFLLRLAQGELPF
OXA-50-like_ASD20357.1_P.a.  QELARRIGLERMRANVSRLGYGNAEIGQVVDNFWLVGPLKISAMEQTHFLLRLAQGELPF
OXA-50-like_AVZ22774.1_P.a.  QELARRIGLERMRANVSRLGYGNAEIGQVVDNFWLVGPLKISAMEQTHFLLRLAQGELPF
OXA-50-like_AXC20842.1_P.a.  QELARRIGLERMRANVSRLGYGNAEIGQVVDNFWLVGPLKISAMEQTHFLLRLAQGELPF
OXA-50-like_AYN84711.1_P.a.  QELARRIGLERMRANVSRLGYGNAEIGQVVDNFWLVGPLKISAMEQTHFLLRLAQGELPF
OXA-50-like_CRZ33166.1_P.a.  QELARRIGLERMRANVSRLGYGNAEIGQVVDNFWLVGPLKISAMEQTHFLLRLAQGELPF
OXA-50-like_QBF65101.1_P.a.  QELARRIGLERMRANVSRLGYGNAEIGQVVDNFWLVGPLKISAMEQTHFLLRLAQGELPF
OXA-50-like_QEF81401.1_P.a.  QELARRIGLERMRANVSRLGYGNAEIGQVVDNFWLVGPLKISAMEQTHFLLRLAQGELPF
OXA-50-like_QEF87414.1_P.a.  QELARRIGLERMRANVSRLGYGNAEIGQVVDNFWLVGPLKISAMEQTHFLLRLAQGELPF
OXA-50-like_QEF93418.1_P.a.  QELARRIGLERMRANVSRLGYGNAEIGQVVDNFWLVGPLKISAMEQTHFLLRLAQGELPF
OXA-50-like_QGM44151.1_P.a.  QELARRIGLERMRANVSRLGYGNAEIGQVVDNFWLVGPLKISAMEQTHFLLRLAQGELPF
OXA-50-like_QHI63098.1_P.a.  QELARRIGLERMRANVSRLGYGNAEIGQVVDNFWLVGPLKISAMEQTHFLLRLAQGELPF
OXA-50-like_QIB85902.1_P.a.  QELARRIGLERMRANVSRLGYGNAEIGQVVDNFWLVGPLKISAMEQTHFLLRLAQGELPF
OXA-50-like_QKR06746.1_P.a.  QELARRIGLERMRANVSRLGYGNAEIGQVVDNFWLVGPLKISAMEQTHFLLRLAQGELPF
OXA-50-like_QKR13035.1_P.a.  QELARRIGLERMRANVSRLGYGNAEIGQVVDNFWLVGPLKISAMEQTHFLLRLAQGELPF
OXA-50-like_QKR18855.1_P.a.  QELARRIGLERMRANVSRLGYGNAEIGQVVDNFWLVGPLKISAMEQTHFLLRLAQGELPF
OXA-50-like_QKR83750.1_P.a.  QELARRIGLERMRANVSRLGYGNAEIGQVVDNFWLVGPLKISAMEQTHFLLRLAQGELPF
OXA-50-like_QLF31591.1_P.a.  QELARRIGLERMRANVSRLGYGNAEIGQVVDNFWLVGPLKISAMEQTHFLLRLAQGELPF
OXA-50-like_VDL04843.1_P.a.  QELARRIGLERMRANVSRLGYGNAEIGQVVDNFWLVGPLKISAMEQTHFLLRLAQGELPF
OXA-50-like_QCP80115.1_P.a.  QELARRIGLERMRANVSRLGYGNAEIGQVVDNFWLVGPLKISAMEQTHFLLRLAQGELPF
OXA-50-like_QEK88934.1_P.a.  QELARRIGLERMRANVSRLGYGNAEIGQVVDNFWLVGPLKISAMEQTHFLLRLAQGELPF
OXA-50-like_ALU47404.1_P.a.  QELARRIGLERMRANVSRLGYGNAEIGQVVDNFWLVGPLKISAMEQTRFLLRLAQGELPF
OXA-50-like_APC75958.1_P.a.  QELARRIGLERMRANVSRLGYGNAEIGQVVDNFWLVGPLKISAMEQTRFLLRLAQGELPF
OXA-50-like_QPZ59753.1_P.a.  QELARRIGLERMRANVSRLGYGNAEIGQVVDNFWLVGPLKISAMEQTRFLLRLAQGELPF
OXA-50-like_QFZ03142.1_P.a.  QELARRIGLERMRANVSRLGYGNAEIGQVVDNFWLVGPLKISAMEQTRFLLRLAQGELPF
OXA-50-like_QGG01915.1_P.a.  QELARRIGLERMRANVSRLGYGNAEIGQVVDNFWLVGPLKISAMEQTRFLLRLAQGELPF
OXA-50-like_AUA86524.1_P.a.  QELARRIGLERMRANVSRLGYGNAEIGQVVDNFWLVGPLKISAMEQTRFLLRLAQGELPF
OXA-50-like_AUA92615.1_P.a.  QELARRIGLERMRANVSRLGYGNAEIGQVVDNFWLVGPLKISAMEQTRFLLRLAQGELPF
4JF4_Beta-lactamase          QELARRIGLDLMQKEVKRIGFGNAEIGQQVDNFWLVGPLKVTPIQEVEFVSQLAHTQLPF
                        acc
```

OXA-50-like subfamily

```
                                  α8              β3  β4      β5          β6               β7
                           180       190       200       210       220        230
OXA-50-like_QQW22444.1_P.a.  PAPVQSTVRAMTLLESGPGWELHGKTGWCFDCTPELGWWVGWVKRNE.RLYGFALNIDMP
OXA-50-like_QGQ00241.1_P.a.  PAPVQSTVRAMTLLESGPGWELHGKTGWCFDCTPELGWWVGWVKRNE.RLYGFALNIDMP
OXA-50-like_QQW19268.1_P.a.  PAPVQSTVRAMTLLESGPGWELHGKTGWCFDCTPELGWWVGWVKRNE.RLYGFALNIDMP
OXA-50-like_QQW06309.1_P.a.  PAPVQSTVRAMTLLESGPGWELHGKTGWCFDCTPELGWWVGWVKRNE.RLYGFALNIDMP
OXA-50-like_QQV61463.1_P.a.  PAPVQSTVRAMTLLESGPGWELHGKTGWCFDCTPELGWWVGWVKRNE.RLYGFALNIDMP
OXA-50-like_QPP39331.1_P.a.  PAPVQSTVRAMTLLESGPGWELHGKTGWCFDCTPELGWWVGWVKRNE.RLYGFALNIDMP
OXA-50-like_QNE62162.1_P.a.  PAPVQSTVRAMTLLESGPGWELHGKTGWCFDCTPELGWWVGWVKRNE.RLYGFALNIDMP
OXA-50-like_QLF44164.1_P.a.  PAPVQSTVRAMTLLESGPGWELHGKTGWCFDCTPELGWWVGWVKRNE.RLYGFALNIDMP
OXA-50-like_QLF18710.1_P.a.  PAPVQSTVRAMTLLESGPGWELHGKTGWCFDCTPELGWWVGWVKRNE.RLYGFALNIDMP
OXA-50-like_QEK46239.1_P.a.  PAPVQSTVRAMTLLESGPGWELHGKTGWCFDCTPELGWWVGWVKRNE.RLYGFALNIDMP
OXA-50-like_QJA41232.1_P.a.  PAPVQSTVRAMTLLESGPGWELHGKTGWCFDCTPELGWWVGWVKRNE.RLYGFALNIDMP
OXA-50-like_AYZ58090.1_P.a.  PAPVQSTVRAMTLLESGPGWELHGKTGWCFDCTPELGWWVGWVKRNE.RLYGFALNIDMP
OXA-50-like_QDR20381.1_P.a.  PAPVQSTVRAMTLLESGPGWELHGKTGWCFDCTPELGWWVGWVKRNE.RLYGFALNIDMP
OXA-50-like_AYW69566.1_P.a.  PAPVQSTVRAMTLLESGPGWELHGKTGWCFDCTPELGWWVGWVKRNE.RLYGFALNIDMP
OXA-50-like_AYZ45913.1_P.a.  PAPVQSTVRAMTLLESGPGWELHGKTGWCFDCTPELGWWVGWVKRNE.RLYGFALNIDMP
OXA-50-like_AYK26584.1_P.a.  PAPVQSTVRAMTLLESGPGWELHGKTGWCFDCTPELGWWVGWVKRNE.RLYGFALNIDMP
OXA-50-like_ASM88695.1_P.a.  PAPVQSTVRAMTLLESGPGWELHGKTGWCFDCTPELGWWVGWVKRNE.RLYGFALNIDMP
OXA-50-like_AUA80490.1_P.a.  PAPVQSTVRAMTLLESGPGWELHGKTGWCFDCTPELGWWVGWVKRNE.RLYGFALNIDMP
OXA-50-like_AUB05120.1_P.a.  PAPVQSTVRAMTLLESGPGWELHGKTGWCFDCTPELGWWVGWVKRNE.RLYGFALNIDMP
OXA-50-like_AVR86641.1_P.a.  PAPVQSTVRAMTLLESGPGWELHGKTGWCFDCTPELGWWVGWVKRNE.RLYGFALNIDMP
OXA-50-like_AXS75147.1_P.a.  PAPVQSTVRAMTLLESGPGWELHGKTGWCFDCTPELGWWVGWVKRNE.RLYGFALNIDMP
OXA-50-like_ALS13221.1_P.a.  PAPVQSTVRAMTLLESGPGWELHGKTGWCFDCTPELGWWVGWVKRNE.RLYGFALNIDMP
OXA-50-like_ALY73377.1_P.a.  PAPVQSTVRAMTLLESGPGWELHGKTGWCFDCTPELGWWVGWVKRNE.RLYGFALNIDMP
OXA-50-like_ANA70339.1_P.a.  PAPVQSTVRAMTLLESGPGWELHGKTGWCFDCTPELGWWVGWVKRNE.RLYGFALNIDMP
OXA-50-like_APB68390.1_P.a.  PAPVQSTVRAMTLLESGPGWELHGKTGWCFDCTPELGWWVGWVKRNE.RLYGFALNIDMP
OXA-50-like_ARI94830.1_P.a.  PAPVQSTVRAMTLLESGPGWELHGKTGWCFDCTPELGWWVGWVKRNE.RLYGFALNIDMP
OXA-50-like_ARJ01367.1_P.a.  PAPVQSTVRAMTLLESGPGWELHGKTGWCFDCTPELGWWVGWVKRNE.RLYGFALNIDMP
OXA-50-like_ASA19039.1_P.a.  PAPVQSTVRAMTLLESGPGWELHGKTGWCFDCTPELGWWVGWVKRNE.RLYGFALNIDMP
OXA-50-like_AUA74232.1_P.a.  PAPVQSTVRAMTLLESGPGWELHGKTGWCFDCTPELGWWVGWVKRNE.RLYGFALNIDMP
OXA-50-like_QCC69374.1_P.a.  PAPVQSTVRAMTLLESGPGWELHGKTGWCFDCTPELGWWVGWVKRNE.RLYGFALNIDMP
OXA-50-like_ATH06157.1_P.a.  PAPVQSTVRAMTLLESGPGWELHGKTGWCFDCTPELGWWVGWVKRNE.RLYGFALNIDMP
OXA-50-like_AZZ14888.1_P.a.  PAPVQSTVRAMTLLESGPGWELHGKTGWCFDCTPELGWWVGWVKRNE.RLYGFALNIDMP
OXA-50-like_VDK95206.1_P.a.  PAPVQSTVRAMTLLESGPGWELHGKTGWCFDCTPELGWWVGWVKRNE.RLYGFALNIDMP
OXA-50-like_VDL11569.1_P.a.  PAPVQSTVRAMTLLESGPGWELHGKTGWCFDCTPELGWWVGWVKRNE.RLYGFALNIDMP
OXA-50-like_VDL54838.1_P.a.  PAPVQSTVRAMTLLESGPGWELHGKTGWCFDCTPELGWWVGWVKRNE.RLYGFALNIDMP
OXA-50-like_VDL56340.1_P.a.  PAPVQSTVRAMTLLESGPGWELHGKTGWCFDCTPELGWWVGWVKRNE.RLYGFALNIDMP
OXA-50-like_VDL57163.1_P.a.  PAPVQSTVRAMTLLESGPGWELHGKTGWCFDCTPELGWWVGWVKRNE.RLYGFALNIDMP
OXA-50-like_ALZ29437.1_P.a.  PAPVQSTVRAMTLLESGPGWELHGKTGWCFDCTPELGWWVGWVKRNE.RLYGFALNIDMP
OXA-50-like_AON10100.1_P.a.  PAPVQSTVRAMTLLESGPGWELHGKTGWCFDCTPELGWWVGWVKRNE.RLYGFALNIDMP
OXA-50-like_AON16083.1_P.a.  PAPVQSTVRAMTLLESGPGWELHGKTGWCFDCTPELGWWVGWVKRNE.RLYGFALNIDMP
OXA-50-like_AON22092.1_P.a.  PAPVQSTVRAMTLLESGPGWELHGKTGWCFDCTPELGWWVGWVKRNE.RLYGFALNIDMP
OXA-50-like_AON28079.1_P.a.  PAPVQSTVRAMTLLESGPGWELHGKTGWCFDCTPELGWWVGWVKRNE.RLYGFALNIDMP
OXA-50-like_AON34087.1_P.a.  PAPVQSTVRAMTLLESGPGWELHGKTGWCFDCTPELGWWVGWVKRNE.RLYGFALNIDMP
OXA-50-like_AON40072.1_P.a.  PAPVQSTVRAMTLLESGPGWELHGKTGWCFDCTPELGWWVGWVKRNE.RLYGFALNIDMP
OXA-50-like_AON46078.1_P.a.  PAPVQSTVRAMTLLESGPGWELHGKTGWCFDCTPELGWWVGWVKRNE.RLYGFALNIDMP
OXA-50-like_AON52080.1_P.a.  PAPVQSTVRAMTLLESGPGWELHGKTGWCFDCTPELGWWVGWVKRNE.RLYGFALNIDMP
OXA-50-like_ARG47835.1_P.a.  PAPVQSTVRAMTLLESGPGWELHGKTGWCFDCTPELGWWVGWVKRNE.RLYGFALNIDMP
OXA-50-like_ASP05026.1_P.a.  PAPVQSTVRAMTLLESGPGWELHGKTGWCFDCTPELGWWVGWVKRNE.RLYGFALNIDMP
OXA-50-like_ASP13070.1_P.a.  PAPVQSTVRAMTLLESGPGWELHGKTGWCFDCTPELGWWVGWVKRNE.RLYGFALNIDMP
OXA-50-like_AVE36468.1_P.a.  PAPVQSTVRAMTLLESGPGWELHGKTGWCFDCTPELGWWVGWVKRNE.RLYGFALNIDMP
OXA-50-like_AXL80491.1_P.a.  PAPVQSTVRAMTLLESGPGWELHGKTGWCFDCTPELGWWVGWVKRNE.RLYGFALNIDMP
OXA-50-like_QDR05681.1_P.a.  PAPVQSTVRAMTLLESGPGWELHGKTGWCFDCTPELGWWVGWVKRNE.RLYGFALNIDMP
OXA-50-like_QGJ36847.1_P.a.  PAPVQSTVRAMTLLESGPGWELHGKTGWCFDCTPELGWWVGWVKRNE.RLYGFALNIDMP
OXA-50-like_QKG35875.1_P.a.  PAPVQSTVRAMTLLESGPGWELHGKTGWCFDCTPELGWWVGWVKRNE.RLYGFALNIDMP
OXA-50-like_QKR24763.1_P.a.  PAPVQSTVRAMTLLESGPGWELHGKTGWCFDCTPELGWWVGWVKRNE.RLYGFALNIDMP
OXA-50-like_QOQ64022.1_P.a.  PAPVQSTVRAMTLLESGPGWELHGKTGWCFDCTPELGWWVGWVKRNE.RLYGFALNIDMP
OXA-50-like_QQD36200.1_P.a.  PAPVQSTVRAMTLLESGPGWELHGKTGWCFDCTPELGWWVGWVKRNE.RLYGFALNIDMP
OXA-50-like_AON75377.1_P.a.  PAPVQSTVRAMTLLESGPGWELHGKTGWCFDCTPELGWWVGWVKRNE.RLYGFALNIDMP
OXA-50-like_QDY03768.1_P.a.  PAPVQSTVRAMTLLESGPGWELHGKTGWCFDCTPELGWWVGWVKRNE.RLYGFALNIDMP
OXA-50-like_QPP46609.1_P.a.  PAPVQSTVRAMTLLESGPGWELHGKTGWCFDCTPELGWWVGWVKRNE.RLYGFALNIDMP
OXA-50-like_QIZ29678.1_P.a.  PAPVQSTVRAMTLLESGPGWELHGKTGWCFDCTPELGWWVGWVKRNE.RLYGFALNIDMP
OXA-50-like_QKR77815.1_P.a.  PAPVQSTVRAMTLLESGPGWELHGKTGWCFDCTPELGWWVGWVKRNE.RLYGFALNIDMP
OXA-50-like_AMA40693.1_P.a.  PAPVQSTVRAMTLLESGPGWELHGKTGWCFDCTPELGWWVGWVKRNE.RLYGFALNIDMP
OXA-50-like_QKE99620.1_P.a.  PAPVQSTVRAMTLLESGPGWELHGKTGWCFDCTPELGWWVGWVKRNE.RLYGFALNIDMP
OXA-50-like_AVZ37799.1_P.a.  PAPVQSTVRAMTLLESGPGWELHGKTGWCFDCTPELGWWVGWVKRNE.RLYGFALNIDMP
OXA-50-like_ASA32790.1_P.a.  PAPVQSTVRAMTLLESGPGWELHGKTGWCFDCTPELGWWVGWVKRNE.RLYGFALNIDMP
OXA-50-like_ASD07216.1_P.a.  PAPVQSTVRAMTLLESGPGWELHGKTGWCFDCTPELGWWVGWVKRNE.RLYGFALNIDMP
OXA-50-like_VDL31009.1_P.a.  PAPVQSTVRAMTLLESGPGWELHGKTGWCFDCTPELGWWVGWVKRNE.RLYGFALNIDMP
OXA-50-like_VDL53067.1_P.a.  PAPVQSTVRAMTLLESGPGWELHGKTGWCFDCTPELGWWVGWVKRNE.RLYGFALNIDMP
OXA-50-like_AYF72086.1_P.a.  PAPVQSTVRAMTLLESGPGWELHGKTGWCFDCTPELGWWVGWVKRNE.RLYGFALNIDMP
OXA-50-like_AYQ83944.1_P.a.  PAPVQSTVRAMTLLESGPGWELHGKTGWCFDCTPELGWWVGWVKRNE.RLYGFALNIDMP
OXA-50-like_AYR15974.1_P.a.  PAPVQSTVRAMTLLESGPGWELHGKTGWCFDCTPELGWWVGWVKRNE.RLYGFALNIDMP
OXA-50-like_AZN03541.1_P.a.  PAPVQSTVRAMTLLESGPGWELHGKTGWCFDCTPELGWWVGWVKRNE.RLYGFALNIDMP
OXA-50-like_AZN09267.1_P.a.  PAPVQSTVRAMTLLESGPGWELHGKTGWCFDCTPELGWWVGWVKRNE.RLYGFALNIDMP
OXA-50-like_AZN16304.1_P.a.  PAPVQSTVRAMTLLESGPGWELHGKTGWCFDCTPELGWWVGWVKRNE.RLYGFALNIDMP
OXA-50-like_AZN49449.1_P.a.  PAPVQSTVRAMTLLESGPGWELHGKTGWCFDCTPELGWWVGWVKRNE.RLYGFALNIDMP
OXA-50-like_AZN54830.1_P.a.  PAPVQSTVRAMTLLESGPGWELHGKTGWCFDCTPELGWWVGWVKRNE.RLYGFALNIDMP
OXA-50-like_AZN61319.1_P.a.  PAPVQSTVRAMTLLESGPGWELHGKTGWCFDCTPELGWWVGWVKRNE.RLYGFALNIDMP
OXA-50-like_QPZ71933.1_P.a.  PAPVQSTVRAMTLLESGPGWELHGKTGWCFDCTPELGWWVGWVKRNE.RLYGFALNIDMP
OXA-50-like_QQM09528.1_P.a.  PAPVQSTVRTMTLLESGPGWELHGKTGWCFDCTPELGWWVGWVKRNE.RLYGFALNIDMP
OXA-50-like_AVN44779.1_P.a.  PAPVQSTVRAMTLLESGQGWELHGKTGWCFDCTPELGWWVGWVKRNE.RLYGFALNIDMP
OXA-50-like_QKR65469.1_P.a.  PAPVQSTVRAMTLLESGQGWELHGKTGWCFDCTPELGWWVGWVKRNE.RLYGFALNIDMP
OXA-50-like_AYW63307.1_P.a.  PAPVQSTVRAMTLLESGPGWELHGKTGWCFDCTPELGWWVGWVKRNE.RLYGFALNIDMP
OXA-50-like_QPZ65977.1_P.a.  PAPVQSTVRAMTLLESGPGWELHGKTGWCFDCTPELGWWVGWVKRNE.RLYGFALNIDMP
OXA-50-like_QHF92313.1_P.a.  PAPVQSTVRAMTLLESGPGWELHGKTGWCFDCTPELGWWVGWVKRNE.RLYGFALNIDMP
OXA-50-like_AZM83163.1_P.a.  PAPVQSTVRAMTLLESGPGWELHGKTGWCFDCTPELGWWVGWVKRNE.RLYGFALNIDMP
OXA-50-like_ALY58484.1_P.a.  PAPVQSTVRAMTLLESGPGWELHGKTGWCFDCTPELGWWVGWVKRNE.RLYGFALNIDMP
OXA-50-like_AHA19368.1_P.a.  PAPVQSTVRAMTLLESGPGWELHGKTGWCFDCTPELGWWVGWVKRNE.RLYGFALNIDMP
OXA-50-like_ALE51523.1_P.a.  PAPVQSTVRAMTLLESGPGWELHGKTGWCFDCTPELGWWVGWVKRNE.RLYGFALNIDMP
OXA-50-like_ALY50692.1_P.a.  PAPVQSTVRAMTLLESGPGWELHGKTGWCFDCTPELGWWVGWVKRNE.RLYGFALNIDMP
OXA-50-like_ALY86245.1_P.a.  PAPVQSTVRAMTLLESGPGWELHGKTGWCFDCTPELGWWVGWVKRNE.RLYGFALNIDMP
OXA-50-like_ALZ22498.1_P.a.  PAPVQSTVRAMTLLESGPGWELHGKTGWCFDCTPELGWWVGWVKRNE.RLYGFALNIDMP
OXA-50-like_AOP60986.1_P.a.  PAPVQSTVRAMTLLESGPGWELHGKTGWCFDCTPELGWWVGWVKRNE.RLYGFALNIDMP
OXA-50-like_ASD00726.1_P.a.  PAPVQSTVRAMTLLESGPGWELHGKTGWCFDCTPELGWWVGWVKRNE.RLYGFALNIDMP
OXA-50-like_AXR31931.1_P.a.  PAPVQSTVRAMTLLESGPGWELHGKTGWCFDCTPELGWWVGWVKRNE.RLYGFALNIDMP
OXA-50-like_AYW76172.1_P.a.  PAPVQSTVRAMTLLESGPGWELHGKTGWCFDCTPELGWWVGWVKRNE.RLYGFALNIDMP
OXA-50-like_QAS76324.1_P.a.  PAPVQSTVRAMTLLESGPGWELHGKTGWCFDCTPELGWWVGWVKRNE.RLYGFALNIDMP
OXA-50-like_QDD38928.1_P.a.  PAPVQSTVRAMTLLESGPGWELHGKTGWCFDCTPELGWWVGWVKRNE.RLYGFALNIDMP
OXA-50-like_QES85928.1_P.a.  PAPVQSTVRAMTLLESGPGWELHGKTGWCFDCTPELGWWVGWVKRNE.RLYGFALNIDMP
OXA-50-like_QJH86618.1_P.a.  PAPVQSTVRAMTLLESGPGWELHGKTGWCFDCTPELGWWVGWVKRNE.RLYGFALNIDMP
OXA-50-like_QKK88642.1_P.a.  PAPVQSTVRAMTLLESGPGWELHGKTGWCFDCTPELGWWVGWVKRNE.RLYGFALNIDMP
OXA-50-like_QKR30875.1_P.a.  PAPVQSTVRAMTLLESGPGWELHGKTGWCFDCTPELGWWVGWVKRNE.RLYGFALNIDMP
OXA-50-like_QKR36250.1_P.a.  PAPVQSTVRAMTLLESGPGWELHGKTGWCFDCTPELGWWVGWVKRNE.RLYGFALNIDMP
```

OXA-50-like subfamily

```
OXA-50-like_QKS42441.1_P.a.  PAPVQSTVRAMTLLESGPGWELHGKTGWCFDCTPELGWWVGWVKRNE.RLYGFALNIDMP
OXA-50-like_QPP27676.1_P.a.  PAPVQSTVRAMTLLESGPGWELHGKTGWCFDCTPELGWWVGWVKRNE.RLYGFALNIDMP
OXA-50-like_QPV54155.1_P.a.  PAPVQSTVRAMTLLESGPGWELHGKTGWCFDCTPELGWWVGWVKRNE.RLYGFALNIDMP
OXA-50-like_QPZ84995.1_P.a.  PAPVQSTVRAMTLLESGPGWELHGKTGWCFDCTPELGWWVGWVKRNE.RLYGFALNIDMP
OXA-50-like_ANP59968.1_P.a.  PAPVQSTVRAMTLLESGPGWELHGKTGWCFDCTPELGWWVGWVKRNE.RLYGFALNIDMP
OXA-50-like_QGQ06905.1_P.a.  PAPVQSTVRAMTLLESGPGWELHGKTGWCFDCTPELGWWVGWVKRNE.RLYGFALNIDMP
OXA-50-like_AXR14368.1_P.a.  PAPVQSTVRAMTLLESGPGWELHGKTGWCFDCTPELGWWVGWVKRNE.RLYGFALNIDMP
OXA-50-like_ALP57413.1_P.a.  PAPVQSTVRAMTLLESGPGWELHGKTGWCFDCTPELGWWVGWVKRNE.RLYGFALNIDMP
OXA-50-like_QKR41777.1_P.a.  PAPVQSTVRAMTLLESGPGWELHGKTGWCFDCTPELGWWVGWVKRNE.RLYGFALNIDMP
OXA-50-like_ALY36156.1_P.a.  PAPVQSTVRAMTLLESGPGWELHGKTGWCFDCTPELGWWVGWVKRNE.RLYGFALNIDMP
OXA-50-like_ALY52550.1_P.a.  PAPVQSTVRAMTLLESGPGWELHGKTGWCFDCTPELGWWVGWVKRNE.RLYGFALNIDMP
OXA-50-like_ANI07822.1_P.a.  PAPVQSTVRAMTLLESGPGWELHGKTGWCFDCTPELGWWVGWVKRNE.RLYGFALNIDMP
OXA-50-like_AWS88163.1_P.a.  PAPVQSTVRAMTLLESGPGWELHGKTGWCFDCTPELGWWVGWVKRNE.RLYGFALNIDMP
OXA-50-like_AXN29478.1_P.a.  PAPVQSTVRAMTLLESGPGWELHGKTGWCFDCTPELGWWVGWVKRNE.RLYGFALNIDMP
OXA-50-like_QBC08854.1_P.a.  PAPVQSTVRAMTLLESGPGWELHGKTGWCFDCTPELGWWVGWVKRNE.RLYGFALNIDMP
OXA-50-like_QDD49249.1_P.a.  PAPVQSTVRAMTLLESGPGWELHGKTGWCFDCTPELGWWVGWVKRNE.RLYGFALNIDMP
OXA-50-like_QDR11871.1_P.a.  PAPVQSTVRAMTLLESGPGWELHGKTGWCFDCTPELGWWVGWVKRNE.RLYGFALNIDMP
OXA-50-like_QKP84324.1_P.a.  PAPVQSTVRAMTLLESGPGWELHGKTGWCFDCTPELGWWVGWVKRNE.RLYGFALNIDMP
OXA-50-like_QKR71283.1_P.a.  PAPVQSTVRAMTLLESGPGWELHGKTGWCFDCTPELGWWVGWVKRNE.RLYGFALNIDMP
OXA-50-like_QLA53973.1_P.a.  PAPVQSTVRAMTLLESGPGWELHGKTGWCFDCTPELGWWVGWVKRNE.RLYGFALNIDMP
OXA-50-like_QLD71620.1_P.a.  PAPVQSTVRAMTLLESGPGWELHGKTGWCFDCTPELGWWVGWVKRNE.RLYGFALNIDMP
OXA-50-like_QLD73935.1_P.a.  PAPVQSTVRAMTLLESGPGWELHGKTGWCFDCTPELGWWVGWVKRNE.RLYGFALNIDMP
OXA-50-like_QLD78684.1_P.a.  PAPVQSTVRAMTLLESGPGWELHGKTGWCFDCTPELGWWVGWVKRNE.RLYGFALNIDMP
OXA-50-like_QLF37576.1_P.a.  PAPVQSTVRAMTLLESGPGWELHGKTGWCFDCTPELGWWVGWVKRNE.RLYGFALNIDMP
OXA-50-like_AWT32452.1_P.a.  PAPVQSTVRAMTLLESGPGWELHGKTGWCFDCTPELGWWVGWVKRNE.RLYGFALNIDMP
OXA-50-like_AXZ92060.1_P.a.  PAPVQSTVRAMTLLESGPGWELHGKTGWCFDCTPELGWWVGWVKRNE.RLYGFALNIDMP
OXA-50-like_QII97759.1_P.a.  PAPVQSTVRAMTLLESGPGWELHGKTGWCFDCTPELGWWVGWVKRNE.RLYGFALNIDMP
OXA-50-like_QOE13519.1_P.a.  PAPVQSTVRAMTLLESGPGWELHGKTGWCFDCTPELGWWVGWVKRNE.RLYGFALNIDMP
OXA-50-like_QPN23057.1_P.a.  PAPVQSTVRAMTLLESGPGWELHGKTGWCFDCTPELGWWVGWVKRNE.RLYGFALNIDMP
OXA-50-like_QDR14302.1_P.a.  PAPVQSTVRAMTLLESGPGWELHGKTGWCFDCTPELGWWVGWVKRNE.RLYGFALNIDMP
OXA-50-like_ALZ16475.1_P.a.  PAPVQSTVRAMTLLESGPGWELHGKTGWCFDCTPELGWWVGWVKRNE.RLYGFALNIDMP
OXA-50-like_QKF04533.1_P.a.  PAPVQSTVRAMTLLESGPGWELHGKTGWCFDCTPELGWWVGWVKRNE.RLYGFALNIDMP
OXA-50-like_QNO22317.1_P.a.  PAPVQSTVRAMTLLESGPGWELHGKTGWCFDCTPELGWWVGWVKRNE.RLYGFALNIDMP
OXA-50-like_QQW12925.1_P.a.  PAPVQSTVRAMTLLESGPGWELHGKTGWCFDCTPELGWWVGWVKRNE.RLYGFALNIDMP
OXA-50-like_AYZ81447.1_P.a.  PAPVQSTVRAMTLLESGPGWELHGKTGWCFDCTPELGWWVGWVKRNE.RLYGFALNIDMP
OXA-50-like_QKL16662.1_P.a.  PTPVQSTVRAMTLLESGPGWELHGKTGWCFDCTPELGWWVGWVKRNE.RLYGFALNIDMP
OXA-50-like_QQW00026.1_P.a.  PTPVQSTVRAMTLLESGPGWELHGKTGWCFDCTPELGWWVGWVKRNE.RLYGFALNIDMP
OXA-50-like_ALV75159.1_P.a.  PAPVQSTVRAMTLLESGPGWELHGKTGWCFDCTPELGWWVGWVKRNE.RLYGFALNIDMP
OXA-50-like_ALY89963.1_P.a.  PAPVQSTVRAMTLLESGPGWELHGKTGWCFDCTPELGWWVGWVKRNE.RLYGFALNIDMP
OXA-50-like_ALY96872.1_P.a.  PAPVQSTVRAMTLLESGPGWELHGKTGWCFDCTPELGWWVGWVKRNE.RLYGFALNIDMP
OXA-50-like_AMX88486.1_P.a.  PAPVQSTVRAMTLLESGPGWELHGKTGWCFDCTPELGWWVGWVKRNE.RLYGFALNIDMP
OXA-50-like_ARC82831.1_P.a.  PAPVQSTVRAMTLLESGPGWELHGKTGWCFDCTPELGWWVGWVKRNE.RLYGFALNIDMP
OXA-50-like_ASD14027.1_P.a.  PAPVQSTVRAMTLLESGPGWELHGKTGWCFDCTPELGWWVGWVKRNE.RLYGFALNIDMP
OXA-50-like_AYZ75988.1_P.a.  PAPVQSTVRAMTLLESGPGWELHGKTGWCFDCTPELGWWVGWVKRNE.RLYGFALNIDMP
OXA-50-like_QFZ61919.1_P.a.  PAPVQSTVRAMTLLESGPGWELHGKTGWCFDCTPELGWWVGWVKRNE.RLYGFALNIDMP
OXA-50-like_QKP90385.1_P.a.  PAPVQSTVRAMTLLESGPGWELHGKTGWCFDCTPELGWWVGWVKRNE.RLYGFALNIDMP
OXA-50-like_QKR47436.1_P.a.  PAPVQSTVRAMTLLESGPGWELHGKTGWCFDCTPELGWWVGWVKRNE.RLYGFALNIDMP
OXA-50-like_QKR53224.1_P.a.  PAPVQSTVRAMTLLESGPGWELHGKTGWCFDCTPELGWWVGWVKRNE.RLYGFALNIDMP
OXA-50-like_QKR59118.1_P.a.  PAPVQSTVRAMTLLESGPGWELHGKTGWCFDCTPELGWWVGWVKRNE.RLYGFALNIDMP
OXA-50-like_QLF25267.1_P.a.  PAPVQSTVRAMTLLESGPGWELHGKTGWCFDCTPELGWWVGWVKRNE.RLYGFALNIDMP
OXA-50-like_QPP21637.1_P.a.  PAPVQSTVRAMTLLESGPGWELHGKTGWCFDCTPELGWWVGWVKRNE.RLYGFALNIDMP
OXA-50-like_QQA85373.1_P.a.  PAPVQSTVRAMTLLESGPGWELHGKTGWCFDCTPELGWWVGWVKRNE.RLYGFALNIDMP
OXA-50-like_QRG72678.1_P.a.  PAPVQSTVRAMTLLESGPGWELHGKTGWCFDCTPELGWWVGWVKRNE.RLYGFALNIDMP
OXA-50-like_AYY40220.1_P.a.  PAPVQSTVRAMTLLESGPGWELHGKTGWCFDCTPELGWWVGWVKRNE.RLYGFALNIDMP
OXA-50-like_ALZ28576.1_P.a.  PAPVQSTVRAMTLLESGPGWELHGKTGWCFDCTPELGWWVGWVKRNE.RLYGFALNIDMP
OXA-50-like_QNQ04301.1_P.a.  PAPVQSTVRAMTLLESGPGWELHGKTGWCFDCTPELGWWVGWVKRNE.RLYGFALNIDMP
OXA-50-like_ALZ94285.1_P.a.  PAPVQSTVRAMTLLESGPGWELHGKTGWCFDCTPELGWWVGWVKRNE.RLYGFALNIDMP
OXA-50-like_AMU04106.1_P.a.  PAPVQSTVRAMTLLESGPGWELHGKTGWCFDCTPELGWWVGWVKRNE.RLYGFALNIDMP
OXA-50-like_AOT37986.1_P.a.  PAPVQSTVRAMTLLESGPGWELHGKTGWCFDCTPELGWWVGWVKRNE.RLYGFALNIDMP
OXA-50-like_ASD20357.1_P.a.  PAPVQSTVRAMTLLESGPGWELHGKTGWCFDCTPELGWWVGWVKRNE.RLYGFALNIDMP
OXA-50-like_AVZ22774.1_P.a.  PAPVQSTVRAMTLLESGPGWELHGKTGWCFDCTPELGWWVGWVKRNE.RLYGFALNIDMP
OXA-50-like_AXC20842.1_P.a.  PAPVQSTVRAMTLLESGPGWELHGKTGWCFDCTPELGWWVGWVKRNE.RLYGFALNIDMP
OXA-50-like_AYN84711.1_P.a.  PAPVQSTVRAMTLLESGPGWELHGKTGWCFDCTPELGWWVGWVKRNE.RLYGFALNIDMP
OXA-50-like_CRZ33166.1_P.a.  PAPVQSTVRAMTLLESGPGWELHGKTGWCFDCTPELGWWVGWVKRNE.RLYGFALNIDMP
OXA-50-like_QBF65101.1_P.a.  PAPVQSTVRAMTLLESGPGWELHGKTGWCFDCTPELGWWVGWVKRNE.RLYGFALNIDMP
OXA-50-like_QEF81401.1_P.a.  PAPVQSTVRAMTLLESGPGWELHGKTGWCFDCTPELGWWVGWVKRNE.RLYGFALNIDMP
OXA-50-like_QEF87414.1_P.a.  PAPVQSTVRAMTLLESGPGWELHGKTGWCFDCTPELGWWVGWVKRNE.RLYGFALNIDMP
OXA-50-like_QEF93418.1_P.a.  PAPVQSTVRAMTLLESGPGWELHGKTGWCFDCTPELGWWVGWVKRNE.RLYGFALNIDMP
OXA-50-like_QGM44151.1_P.a.  PAPVQSTVRAMTLLESGPGWELHGKTGWCFDCTPELGWWVGWVKRNE.RLYGFALNIDMP
OXA-50-like_QHI63098.1_P.a.  PAPVQSTVRAMTLLESGPGWELHGKTGWCFDCTPELGWWVGWVKRNE.RLYGFALNIDMP
OXA-50-like_QIB85902.1_P.a.  PAPVQSTVRAMTLLESGPGWELHGKTGWCFDCTPELGWWVGWVKRNE.RLYGFALNIDMP
OXA-50-like_QKR06746.1_P.a.  PAPVQSTVRAMTLLESGPGWELHGKTGWCFDCTPELGWWVGWVKRNE.RLYGFALNIDMP
OXA-50-like_QKR13035.1_P.a.  PAPVQSTVRAMTLLESGPGWELHGKTGWCFDCTPELGWWVGWVKRNE.RLYGFALNIDMP
OXA-50-like_QKR18855.1_P.a.  PAPVQSTVRAMTLLESGPGWELHGKTGWCFDCTPELGWWVGWVKRNE.RLYGFALNIDMP
OXA-50-like_QKR83750.1_P.a.  PAPVQSTVRAMTLLESGPGWELHGKTGWCFDCTPELGWWVGWVKRNE.RLYGFALNIDMP
OXA-50-like_QLF31591.1_P.a.  PAPVQSTVRAMTLLESGPGWELHGKTGWCFDCTPELGWWVGWVKRNE.RLYGFALNIDMP
OXA-50-like_VDL04843.1_P.a.  PAPVQSTVRAMTLLESGPGWELHGKTGWCFDCTPELGWWVGWVKRNE.RLYGFALNIDMP
OXA-50-like_QCP80115.1_P.a.  PAPVQSTVRAMTLLESGPGWELHGKTGWCFDCTPELGWWVGWVKRNE.RLYGFALNIDMP
OXA-50-like_QEK88934.1_P.a.  PAPVQSTVRAMTLLESGPGWELHGKTGWCFDCTPELGWWVGWVKRNE.RLYGFALNIDMP
OXA-50-like_ALU47404.1_P.a.  PAPVQSTVRAMTLLESGPGWELHGKTGWCFDCTPELGWWVGWVKRNE.RLYGFALNIDMP
OXA-50-like_APC75958.1_P.a.  PAPVQSTVRAMTLLESGPGWELHGKTGWCFDCTPELGWWVGWVKRNE.RLYGFALNIDMP
OXA-50-like_QPZ59753.1_P.a.  PAPVQSTVRAMTLLESGPGWELHGKTGWCFDCTPELGWWVGWVKRNE.RLYGFALNIDMP
OXA-50-like_QFZ03142.1_P.a.  PAPVQSTVRAMTLLESGPGWELHGKTGWCFDCTPELGWWVGWVKRNE.RLYGFALNIDMP
OXA-50-like_QGG01915.1_P.a.  PAPVQSTVRAMTLLESGPGWELHGKTGWCFDCTPELGWWVGWVKRNE.RLYGFALNIDMP
OXA-50-like_AUA86524.1_P.a.  PAPVQSTVRAMTLLESGPGWELHGKTGWCFDCTPELGWWVGWVKRNE.RLYGFALNIDMP
OXA-50-like_AUA92615.1_P.a.  PAPVQSTVRAMTLLESGPGWELHGKTGWCFDCTPELGWWVGWVKRNE.RLYGFALNIDMP
4JF4_Beta-lactamase          SEKVQANVKNMLLLEESNGYKIFGKTGWAMDIKPQVGWLTGWVEQPDGKIVAFALNMEMR
                        acc
```

OXA-50-like subfamily

```
                                      α9
                              240        250       260
OXA-50-like_QQW22444.1_P.a.   GGEADIGKRVELGKASLKALGILP
OXA-50-like_QGQ00241.1_P.a.   GGEADIGKRVELGKASLKALGILP
OXA-50-like_QQW19268.1_P.a.   GGEADIGKRVELGKASLKALGILP
OXA-50-like_QQW06309.1_P.a.   GGEADIGKRVELGKASLKALGILP
OXA-50-like_QQV61463.1_P.a.   GGEADIGKRVELGKASLKALGILP
OXA-50-like_QPP39331.1_P.a.   GGEADIGKRVELGKASLKALGILP
OXA-50-like_QNE62162.1_P.a.   GGEADIGKRVELGKASLKALGILP
OXA-50-like_QLF44164.1_P.a.   GGEADIGKRVELGKASLKALGILP
OXA-50-like_QLF18710.1_P.a.   GGEADIGKRVELGKASLKALGILP
OXA-50-like_QEK46239.1_P.a.   GGEADIGKRVELGKASLKALGILP
OXA-50-like_QJA41232.1_P.a.   GGEADIGKRVELGKASLKALGILP
OXA-50-like_AYZ58090.1_P.a.   GGEADIGKRVELGKASLKALGILP
OXA-50-like_QDR20381.1_P.a.   GGEADIGKRVELGKASLKALGILP
OXA-50-like_AYW69566.1_P.a.   GGEADIGKRVELGKASLKALGILP
OXA-50-like_AYZ45913.1_P.a.   GGEADIGKRVELGKASLKALGILP
OXA-50-like_AYK26584.1_P.a.   GGEADIGKRVELGKASLKALGILP
OXA-50-like_ASM88695.1_P.a.   GGEADIGKRVELGKASLKALGILP
OXA-50-like_AUA80490.1_P.a.   GGEADIGKRVELGKASLKALGILP
OXA-50-like_AUB05120.1_P.a.   GGEADIGKRVELGKASLKALGILP
OXA-50-like_AVR86641.1_P.a.   GGEADIGKRVELGKASLKALGILP
OXA-50-like_AXS75147.1_P.a.   GGEADIGKRVELGKASLKALGILP
OXA-50-like_ALS13221.1_P.a.   GGEADIGKRVELGKASLKALGILP
OXA-50-like_ALY73377.1_P.a.   GGEADIGKRVELGKASLKALGILP
OXA-50-like_ANA70339.1_P.a.   GGEADIGKRVELGKASLKALGILP
OXA-50-like_APB68390.1_P.a.   GGEADIGKRVELGKASLKALGILP
OXA-50-like_ARI94830.1_P.a.   GGEADIGKRVELGKASLKALGILP
OXA-50-like_ARJ01367.1_P.a.   GGEADIGKRVELGKASLKALGILP
OXA-50-like_ASA19039.1_P.a.   GGEADIGKRVELGKASLKALGILP
OXA-50-like_AUA74232.1_P.a.   GGEADIGKRVELGKASLKALGILP
OXA-50-like_QCC69374.1_P.a.   GGEADIGKRVELGKASLKALGILP
OXA-50-like_ATH06157.1_P.a.   GGEADIGKRVELGKASLKALGILP
OXA-50-like_AZZ14888.1_P.a.   GGEADIGKRVELGKASLKALGILP
OXA-50-like_VDK95206.1_P.a.   GGEADIGKRVELGKASLKALGILP
OXA-50-like_VDL11569.1_P.a.   GGEADIGKRVELGKASLKALGILP
OXA-50-like_VDL54838.1_P.a.   GGEADIGKRVELGKASLKALGILP
OXA-50-like_VDL56340.1_P.a.   GGEADIGKRVELGKASLKALGILP
OXA-50-like_VDL57163.1_P.a.   GGEADIGKRVELGKASLKALGILP
OXA-50-like_ALZ29437.1_P.a.   GGEADIGKRVELGKASLKALGILP
OXA-50-like_AON10100.1_P.a.   GGEADIGKRVELGKASLKALGILP
OXA-50-like_AON16083.1_P.a.   GGEADIGKRVELGKASLKALGILP
OXA-50-like_AON22092.1_P.a.   GGEADIGKRVELGKASLKALGILP
OXA-50-like_AON28079.1_P.a.   GGEADIGKRVELGKASLKALGILP
OXA-50-like_AON34087.1_P.a.   GGEADIGKRVELGKASLKALGILP
OXA-50-like_AON40072.1_P.a.   GGEADIGKRVELGKASLKALGILP
OXA-50-like_AON46078.1_P.a.   GGEADIGKRVELGKASLKALGILP
OXA-50-like_AON52080.1_P.a.   GGEADIGKRVELGKASLKALGILP
OXA-50-like_ARG47835.1_P.a.   GGEADIGKRVELGKASLKALGILP
OXA-50-like_ASP05026.1_P.a.   GGEADIGKRVELGKASLKALGILP
OXA-50-like_ASP13070.1_P.a.   GGEADIGKRVELGKASLKALGILP
OXA-50-like_AVE36468.1_P.a.   GGEADIGKRVELGKASLKALGILP
OXA-50-like_AXL80491.1_P.a.   GGEADIGKRVELGKASLKALGILP
OXA-50-like_QDR05681.1_P.a.   GGEADIGKRVELGKASLKALGILP
OXA-50-like_QGJ36847.1_P.a.   GGEADIGKRVELGKASLKALGILP
OXA-50-like_QKG35875.1_P.a.   GGEADIGKRVELGKASLKALGILP
OXA-50-like_QKR24763.1_P.a.   GGEADIGKRVELGKASLKALGILP
OXA-50-like_QOQ64022.1_P.a.   GGEADIGKRVELGKASLKALGILP
OXA-50-like_QQD36200.1_P.a.   GGEADIGKRVELGKASLKALGILP
OXA-50-like_AON75377.1_P.a.   GGEADIGKRVELGKASLKALGILP
OXA-50-like_QDY03768.1_P.a.   GGEADIGKRVELGKASLKALGILP
OXA-50-like_QPP46609.1_P.a.   GGEADIGKRVELGKASLKALGILP
OXA-50-like_QIZ29678.1_P.a.   GGEADIGKRVELGKASLTALGILP
OXA-50-like_QKR77815.1_P.a.   GGEADIGKRVELGKASLKALGILP
OXA-50-like_AMA40693.1_P.a.   GGEADIGKRVELGKASLKALGILP
OXA-50-like_QKE99620.1_P.a.   GGEADIGKRVELGKASLKALGILP
OXA-50-like_AVZ37799.1_P.a.   GGEADIGKRVELGKASLKALGILP
OXA-50-like_ASA32790.1_P.a.   GGEADIGKRVELGKASLKALGILP
OXA-50-like_ASD07216.1_P.a.   GGEADIGKRVELGKASLKALGILP
OXA-50-like_VDL31009.1_P.a.   GGEADIGKRVELGKASLKALGILP
OXA-50-like_VDL53067.1_P.a.   GGEADIGKRVELGKASLKALGILP
OXA-50-like_AYF72086.1_P.a.   GGEADIGKRVELGKASLKALGILP
OXA-50-like_AYQ83944.1_P.a.   GGEADIGKRVELGKASLKALGILP
OXA-50-like_AYR15974.1_P.a.   GGEADIGKRVELGKASLKALGILP
OXA-50-like_AZN03541.1_P.a.   GGEADIGKRVELGKASLKALGILP
OXA-50-like_AZN09267.1_P.a.   GGEADIGKRVELGKASLKALGILP
OXA-50-like_AZN16304.1_P.a.   GGEADIGKRVELGKASLKALGILP
OXA-50-like_AZN49449.1_P.a.   GGEADIGKRVELGKASLKALGILP
OXA-50-like_AZN54830.1_P.a.   GGEADIGKRVELGKASLKALGILP
OXA-50-like_AZN61319.1_P.a.   GGEADIGKRVELGKASLKALGILP
OXA-50-like_QPZ71933.1_P.a.   GGEADIGKRVELGKASLKALGILP
OXA-50-like_QQM09528.1_P.a.   GGEADIGKRVELGKASLKALGILP
OXA-50-like_AVN44779.1_P.a.   GGEADIGKRVELGKASLKALGILP
OXA-50-like_QKR65469.1_P.a.   GGEADIGKRVELGKASLKALGILP
OXA-50-like_AYW63307.1_P.a.   GGEADIGKRVELGKASLKALGILP
OXA-50-like_QPZ65977.1_P.a.   GGEADIGKRVELGKASLKALGILP
OXA-50-like_QHF92313.1_P.a.   GGEADIGKRVELGKASLKALGILP
OXA-50-like_AZM83163.1_P.a.   GGEADIGKRVELGKASLKALGILP
OXA-50-like_ALY58484.1_P.a.   GGEADIGKRVELGKASLKALGILP
OXA-50-like_AHA19368.1_P.a.   GGEADIGKRVELGKASLKALGILP
OXA-50-like_ALE51523.1_P.a.   GGEADIGKRVELGKASLKALGILP
OXA-50-like_ALY50692.1_P.a.   GGEADIGKRVELGKASLKALGILP
OXA-50-like_ALY86245.1_P.a.   GGEADIGKRVELGKASLKALGILP
OXA-50-like_ALZ22498.1_P.a.   GGEADIGKRVELGKASLKALGILP
OXA-50-like_AOP60986.1_P.a.   GGEADIGKRVELGKASLKALGILP
OXA-50-like_ASD00726.1_P.a.   GGEADIGKRVELGKASLKALGILP
OXA-50-like_AXR31931.1_P.a.   GGEADIGKRVELGKASLKALGILP
OXA-50-like_AYW76172.1_P.a.   GGEADIGKRVELGKASLKALGILP
OXA-50-like_QAS76324.1_P.a.   GGEADIGKRVELGKASLKALGILP
OXA-50-like_QDD38928.1_P.a.   GGEADIGKRVELGKASLKALGILP
OXA-50-like_QES85928.1_P.a.   GGEADIGKRVELGKASLKALGILP
OXA-50-like_QJH86618.1_P.a.   GGEADIGKRVELGKASLKALGILP
OXA-50-like_QKK88642.1_P.a.   GGEADIGKRVELGKASLKALGILP
OXA-50-like_QKR30875.1_P.a.   GGEADIGKRVELGKASLKALGILP
OXA-50-like_QKR36250.1_P.a.   GGEADIGKRVELGKASLKALGILP
```

OXA-50-like subfamily

```
OXA-50-like_QKS42441.1_P.a.  GGEADIGKRVELGKASLKALGILP
OXA-50-like_QPP27676.1_P.a.  GGEADIGKRVELGKASLKALGILP
OXA-50-like_QPV54155.1_P.a.  GGEADIGKRVELGKASLKALGILP
OXA-50-like_QPZ84995.1_P.a.  GGEADIGKRVELGKASLKALGILP
OXA-50-like_ANP59968.1_P.a.  GGEADIGKRVELGKASLKALGILP
OXA-50-like_QGQ06905.1_P.a.  GGEADIGKRVELGKASLKALGILP
OXA-50-like_AXR14368.1_P.a.  GGEADIGKRVELGKASLKALGILP
OXA-50-like_ALP57413.1_P.a.  GGEADIGKRVELGKASLKALGILP
OXA-50-like_QKR41777.1_P.a.  GGEADIGKRVELGKASLKALGILP
OXA-50-like_ALY36156.1_P.a.  GGEADIGKRVELGKASLKALGILP
OXA-50-like_ALY52550.1_P.a.  GGEADIGKRVELGKASLKALGILP
OXA-50-like_ANI07822.1_P.a.  GGEADIGKRVELGKASLKALGILP
OXA-50-like_AWS88163.1_P.a.  GGEADIGKRVELGKASLKALGILP
OXA-50-like_AXN29478.1_P.a.  GGEADIGKRVELGKASLKALGILP
OXA-50-like_QBC08854.1_P.a.  GGEADIGKRVELGKASLKALGILP
OXA-50-like_QDD49249.1_P.a.  GGEADIGKRVELGKASLKALGILP
OXA-50-like_QDR11871.1_P.a.  GGEADIGKRVELGKASLKALGILP
OXA-50-like_QKP84324.1_P.a.  GGEADIGKRVELGKASLKALGILP
OXA-50-like_QKR71283.1_P.a.  GGEADIGKRVELGKASLKALGILP
OXA-50-like_QLA53973.1_P.a.  GGEADIGKRVELGKASLKALGILP
OXA-50-like_QLD71620.1_P.a.  GGEADIGKRVELGKASLKALGILP
OXA-50-like_QLD73935.1_P.a.  GGEADIGKRVELGKASLKALGILP
OXA-50-like_QLD78684.1_P.a.  GGEADIGKRVELGKASLKALGILP
OXA-50-like_QLF37576.1_P.a.  GGEADIGKRVELGKASLKALGILP
OXA-50-like_AWT32452.1_P.a.  GGEADIGKRVELGKASLKALGILP
OXA-50-like_AXZ92060.1_P.a.  GGEADIGKRVELGKASLKALGILP
OXA-50-like_QII97759.1_P.a.  GGEADIGKRVELGKASLKALGILP
OXA-50-like_QOE13519.1_P.a.  GGEADIGKRVELGKASLKALGILP
OXA-50-like_QPN23057.1_P.a.  GGEADIGKRVELGKASLKALGILP
OXA-50-like_QDR14302.1_P.a.  GGEADIGKRVELGKASLKALGILP
OXA-50-like_ALZ16475.1_P.a.  GGEADIGKRVELGKASLKALGILP
OXA-50-like_QKF04533.1_P.a.  GGEADIGKRVELGKASLKALGILP
OXA-50-like_QNO22317.1_P.a.  GGEADIGKRVELGKASLKALGILP
OXA-50-like_QQW12925.1_P.a.  GGEADIGKRVELGKASLKALGILP
OXA-50-like_AYZ81447.1_P.a.  GGEADIGKRVELGKASLKALGILP
OXA-50-like_QKL16662.1_P.a.  GGEADIGKRVELGKASLKALGILP
OXA-50-like_QQW00026.1_P.a.  GGEADIGKRVELGKASLKALGILP
OXA-50-like_ALV75159.1_P.a.  GGEADIGKRVELGKASLKALGILP
OXA-50-like_ALY89963.1_P.a.  GGEADIGKRVELGKASLKALGILP
OXA-50-like_ALY96872.1_P.a.  GGEADIGKRVELGKASLKALGILP
OXA-50-like_AMX88486.1_P.a.  GGEADIGKRVELGKASLKALGILP
OXA-50-like_ARC82831.1_P.a.  GGEADIGKRVELGKASLKALGILP
OXA-50-like_ASD14027.1_P.a.  GGEADIGKRVELGKASLKALGILP
OXA-50-like_AYZ75988.1_P.a.  GGEADIGKRVELGKASLKALGILP
OXA-50-like_QFZ61919.1_P.a.  GGEADIGKRVELGKASLKALGILP
OXA-50-like_QKP90385.1_P.a.  GGEADIGKRVELGKASLKALGILP
OXA-50-like_QKR47436.1_P.a.  GGEADIGKRVELGKASLKALGILP
OXA-50-like_QKR53224.1_P.a.  GGEADIGKRVELGKASLKALGILP
OXA-50-like_QKR59118.1_P.a.  GGEADIGKRVELGKASLKALGILP
OXA-50-like_QLF25267.1_P.a.  GGEADIGKRVELGKASLKALGILP
OXA-50-like_QPP21637.1_P.a.  GGEADIGKRVELGKASLKALGILP
OXA-50-like_QQA85373.1_P.a.  GGEADIGKRVELGKASLKALGILP
OXA-50-like_QRG72678.1_P.a.  GGEADIGKRVELGKASLKALGILP
OXA-50-like_AYY40220.1_P.a.  GGEADIGKRVELGKASLKALGILP
OXA-50-like_ALZ28576.1_P.a.  GGEADIGKRVELGKASLKALGILP
OXA-50-like_QNQ04301.1_P.a.  GGEADIGKRVELGKASLKALGILP
OXA-50-like_ALZ94285.1_P.a.  GGEADIGKRVELGKASLKALGILP
OXA-50-like_AMU04106.1_P.a.  GGEADIGKRVELGKASLKALGILP
OXA-50-like_AOT37986.1_P.a.  GGEADIGKRVELGKASLKALGILP
OXA-50-like_ASD20357.1_P.a.  GGEADIGKRVELGKASLKALGILP
OXA-50-like_AVZ22774.1_P.a.  GGEADIGKRVELGKASLKALGILP
OXA-50-like_AXC20842.1_P.a.  GGEADIGKRVELGKASLKALGILP
OXA-50-like_AYN84711.1_P.a.  GGEADIGKRVELGKASLKALGILP
OXA-50-like_CRZ33166.1_P.a.  GGEADIGKRVELGKASLKALGILP
OXA-50-like_QBF65101.1_P.a.  GGEADIGKRVELGKASLKALGILP
OXA-50-like_QEF81401.1_P.a.  GGEADIGKRVELGKASLKALGILP
OXA-50-like_QEF87414.1_P.a.  GGEADIGKRVELGKASLKALGILP
OXA-50-like_QEF93418.1_P.a.  GGEADIGKRVELGKASLKALGILP
OXA-50-like_QGM44151.1_P.a.  GGEADIGKRVELGKASLKALGILP
OXA-50-like_QHI63098.1_P.a.  GGEADIGKRVELGKASLKALGILP
OXA-50-like_QIB85902.1_P.a.  GGEADIGKRVELGKASLKALGILP
OXA-50-like_QKR06746.1_P.a.  GGEADIGKRVELGKASLKALGILP
OXA-50-like_QKR13035.1_P.a.  GGEADIGKRVELGKASLKALGILP
OXA-50-like_QKR18855.1_P.a.  GGEADIGKRVELGKASLKALGILP
OXA-50-like_QKR83750.1_P.a.  GGEADIGKRVELGKASLKALGILP
OXA-50-like_QLF31591.1_P.a.  GGEADIGKRVELGKASLKALGILP
OXA-50-like_VDL04843.1_P.a.  GGEADIGKRVELGKASLKALGILP
OXA-50-like_QCP80115.1_P.a.  GGEADIGKRVELGKASLKALGILP
OXA-50-like_QEK88934.1_P.a.  GGEADIGKRVELGKASLKALGILP
OXA-50-like_ALU47404.1_P.a.  GGEADIGKRVELGKASLKALGILP
OXA-50-like_APC75958.1_P.a.  GGEADIGKRVELGKASLKALGILP
OXA-50-like_QPZ59753.1_P.a.  GGEADIGKRVELGKASLKALGILP
OXA-50-like_QFZ03142.1_P.a.  GGEADIGKRVELGKASLKALGILP
OXA-50-like_QGG01915.1_P.a.  GGEADIGKRVELGKASLKALGILP
OXA-50-like_AUA86524.1_P.a.  GGEADIGKRVELSKASLKALGILP
OXA-50-like_AUA92615.1_P.a.  GGEADIGKRVELSKASLKALGILP
4JF4_Beta-lactamase          .SEMPASIRNELLMKSLKQLNII.
                        acc
```

OXA-51-like subfamily

```
                                                                  α1            β1
4ZDX_Beta-lactamase                                     QQQQQQQQQQQQQ     ──────►TT
                             1        10        20        30        40        50        60
4ZDX_Beta-lactamase          MNIKTLLLITSAIFISACSPYIVTANPNHSASKSDEKAEKIKNLFNEVHTTGVLVIQQGQ
OXA-51-like_QLB35675.1_A.b.  MNIKTLLLITSAIFISACSPYIVTANPNHSASKSDEKAEKIKNLFNEVHTTGVLVIQQGQ
OXA-51-like_AUT37227.1_A.b.  MNIKTLLLITSAIFISACSPYIVTANPNHSASKSDEKAEKIKNLFNEVHTTGVLVIQQGQ
OXA-51-like_QJF39460.1_A.b.  MNIKTLLLITSAIFISACSPYIVTANPNHSASKSDEKAEKIKNLFNEVHTTGVLVIQQGQ
OXA-51-like_QLI40100.1_A.b.  MNIKTLLLITSAIFISACSPYIVTANPNHSASKSDEKAEKIKNLFNEVHTTGVLVIQQGQ
OXA-51-like_ARG36573.1_A.b.  MNIKALLLITSAIFISACSPYIVTANPNHSASKSDEKGEKIKNLFNEAHTTGVLVIQQGQ
OXA-51-like_ASO71619.1_A.b.  MNIKALLLITSAIFISACSPYIVTANPNHSASKSDEKGEKIKNLFNEAHTTGVLVIQQGQ
OXA-51-like_AVP34890.1_A.b.  MNIKALLLITSAIFISACSPYIVTANPNHSASKSDEKGEKIKNLFNEAHTTGVLVIQQGQ
OXA-51-like_QCR89674.1_A.b.  MNIKALLLITSAIFISACSPYIVTANPNHSASKSDEKGEKIKNLFNEAHTTGVLVIQQGQ
OXA-51-like_QJH20957.1_A.b.  MNIKALLLITSAIFISACSPYIVTANPNHSASKSDEKGEKIKNLFNEAHTTGVLVIQQGQ
OXA-51-like_QMS82274.1_A.b.  MNIKALLLITSAIFISACSPYIVTANPNHSASKSDEKGEKIKNLFNEAHTTGVLVIQQGQ
OXA-51-like_QCT16238.1_A.b.  MNIKALLLITSAIFISACSPYIVTANPNHSASKSDEKAEKIKNLFNEAHTTGVLVIQQGQ
OXA-51-like_AVF07049.1_A.b.  MNIKALLLITSAIFISACSPYIVTANPNHSASKSDEKAEKIKNLFNEAHTTGVLVIQQGQ
OXA-51-like_AVF08644.1_A.b.  MNIKALLLITSAIFISACSPYIVTANPNHSASKSDEKAEKIKNLFNEAHTTGVLVIQQGQ
OXA-51-like_QBR77846.1_A.b.  MNIKALLLITSTIFISACSPYIVTANPNHSTSKSDEKAEKIKNLFNEAHTTGVLVIQQGQ
OXA-51-like_QBR79997.1_A.b.  MNIKALLLITSTIFISACSPYIVTANPNHSTSKSDEKAEKIKNLFNEAHTTGVLVIQQGQ
OXA-51-like_ABO11944.2_A.b.  MNIKALLLITSAIFISACSPYIVTANPNHSASKSDEKAEKIKNLFNEAHTTGVLVIQQGQ
OXA-51-like_AGH35408.1_A.b.  MNIKALLLITSAIFISACSPYIVTANPNHSASKSDEKAEKIKNLFNEAHTTGVLVIQQGQ
OXA-51-like_BBT49379.1_A.b.  MNIKALLLITSAIFISACSPYIVTANPNHSASKSDEKAEKIKNLFNEAHTTGVLVIQQGQ
OXA-51-like_QAB40708.1_A.b.  MNIKALLLITSAIFISACSPYIVTANPNHSASKSDEKAEKIKNLFNEAHTTGVLVIQQGQ
OXA-51-like_QDQ60454.1_A.b.  MNIKALLLITSAIFISACSPYIVTANPNHSASKSDEKAEKIKNLFNEAHTTGVLVIQQGQ
OXA-51-like_QKY23678.1_A.b.  MNIKALLLITSAIFISACSPYIVTANPNHSASKSDEKAEKIKNLFNEAHTTGVLVIQQGQ
OXA-51-like_QLI36478.1_A.b.  MNIKALLLITSAIFISACSPYIVTANPNHSASKSDEKAEKIKNLFNEAHTTGVLVIQQGQ
OXA-51-like_QNT82744.1_A.b.  MNIKALLLITSAIFISACSPYIVTANPNHSASKSDEKAEKIKNLFNEAHTTGVLVIQQGQ
OXA-51-like_APX50552.1_A.b.  MNIKALLLITSAIFISACSPYIVTANPNHSASKSDEKAEKIKNLFNEAHTTGVLVIQQGQ
OXA-51-like_QER37219.1_A.b.  MNIKALLLITSAIFISACSPYIVTANPNHSASKSDEKAEKIKNLFNEAHTTGVLVIQQGQ
OXA-51-like_QLF06531.1_A.b.  MNIKALLLITSAIFISACSPYIVTANPNHSASKSDKKAEKIKNLFNEAHTTGVLVIQQDQ
OXA-51-like_AGQ06390.1_A.b.  MNIKALLLITSAIFISACSPYIVSANPNHSASKSDEKAEKIKNLFNEAHTTGVLVIQQGQ
OXA-51-like_QCP28577.1_A.b.  MNIKALLLITSAIFISACSPYIVSANPNHSASKSDEKAEKIKNLFNEAHTTGVLVIQQGQ
OXA-51-like_AIY37715.2_A.b.  MNIKALLLITSAIFISACSPYIVSANPNHSASKSDEKAEKIKNLFNEAHTTGVLVIQQGQ
OXA-51-like_APO58838.1_A.b.  MNIKALLLITSAIFISACSPYIVSANPNHSASKSDEKAEKIKNLFNEAHTTGVLVIQQGQ
OXA-51-like_AXX43643.1_A.b.  MNIKALLLITSAIFISACSPYIVSANPNHSASKSDEKAEKIKNLFNEAHTTGVLVIQQGQ
OXA-51-like_QBY14110.1_A.b.  MNIKALLLITSAIFISACSPYIVSANPNHSASKSDEKAEKIKNLFNEAHTTGVLVIQQGQ
OXA-51-like_QCP23393.1_A.b.  MNIKALLLITSAIFISACSPYIVSANPNHSASKSDEKAEKIKNLFNEAHTTGVLVIQQGQ
OXA-51-like_QJG76604.1_A.b.  MNIKALLLITSAIFISACSPYIVSANPNHSASKSDEKAEKIKNLFNEAHTTGVLVIQQGQ
OXA-51-like_QJG80512.1_A.b.  MNIKALLLITSAIFISACSPYIVSANPNHSASKSDEKAEKIKNLFNEAHTTGVLVIQQGQ
OXA-51-like_QJG83258.1_A.b.  MNIKALLLITSAIFISACSPYIVSANPNHSASKSDEKAEKIKNLFNEAHTTGVLVIQQGQ
OXA-51-like_QJP37521.1_A.b.  MNIKALLLITSAIFISACSPYIVSANPNHSASKSDEKAEKIKNLFNEAHTTGVLVIQQGQ
OXA-51-like_ARG37991.1_A.b.  MNIKALLLITSAIFISACSPYIVSANPNHSASKSDEKAEKIKNLFNEAHTTGVLVIQQGQ
OXA-51-like_AMN01331.1_A.b.  MNIKALLLITSAIFISACSPYIVSANPNHSASKSDEKAEKIKNLFNEAHTTGVLVIQQGQ
OXA-51-like_ARN31023.1_A.b.  MNIKALLLITSAIFISACSPYIVSANPNHSASKSDEKAEKIKNLFNEAHTTGVLVIQQGQ
OXA-51-like_QDM66466.1_A.b.  MNIKALLLITSAIFISACSPYIVSANPNHSASKSDEKAEKIKNLFNEAHTTGVLVIQQGQ
OXA-51-like_QEY04089.1_A.b.  MNIKALLLITSAIFISACSPYIVSANPNHSASKSDEKAEKIKNLFNEAHTTGVLVIQQGQ
OXA-51-like_QFQ05250.1_A.b.  MNIKALLLITSAIFISACSPYIVSANPNHSASKSDEKAEKIKNLFNEAHTTGVLVIQQGQ
OXA-51-like_QGX51318.1_A.b.  MNIKALLLITSAIFISACSPYIVSANPNHSASKSDEKAEKIKNLFNEAHTTGVLVIQQGQ
OXA-51-like_QPP12630.1_A.b.  MNIKALLLITSAIFISACSPYIVSANPNHSASKSDEKAEKIKNLFNEAHTTGVLVIQQGQ
OXA-51-like_QNT83403.1_A.b.  MNIKALLLITSAIFISACSPYIVSANPNHSASKSDEKAEKIKNLFNEAHTTGVLVIQQGQ
OXA-51-like_QPF14787.1_A.b.  MNIKALLLITSAIFISACSPYIVSANPNHSASKSDEKAEKIKNLFNEAHTTGVLVIQQGQ
OXA-51-like_QJF32440.1_A.b.  MNIKALLLITSAIFISACSPYIVTANPNHSASKSDKKAEKIKNLFNEAHTTGVLVIQQGQ
OXA-51-like_QJF35790.1_A.b.  MNIKALLLITSAIFISACSPYIVTANPNHSASKSDKKAEKIKNLFNEAHTTGVLVIQQGQ
OXA-51-like_QLF10281.1_A.b.  MNIKALLLITSAIFISACSPYIVTANPNHSASKSDKKAEKIKNLFNEAHTTGVLVIQQGQ
OXA-51-like_AYY52683.1_A.b.  MNIKALLLITSAIFISACSPYIVTANPNHSASKSDKKAEKIKNLFNEAHTTGVLVIQQGQ
OXA-51-like_SCD15761.1_A.b.  MNIKALLLITSAIFISACSPYIVSANPNHSASKSDEKAEKIKNLFNEAHTTGVLVIQQGQ
OXA-51-like_BAP66909.1_A.b.  MNIKALLLITSAIFISACSPYIVTANPNHSASKSDEKAEKIKNLFNEAHTTGVLVIQQGQ
OXA-51-like_QCP30880.1_A.b.  MNIKALLLITSAIFISACSPYIVTANPNHSASKSDEKAEKIKNLFNEAHTTGVLVIQQGQ
OXA-51-like_QER75560.1_A.b.  MNIKALLLITSAIFISACSPYIVTANPNHSASKSDEKAEKIKNLFNEAHTTGVLVIQQGQ
OXA-51-like_QCO80718.1_A.b.  MNIKALLLITSAIFISACSPYIVTANPNHSASKSDEKAEKIKNLFNEAHTTGVLVIQQGQ
OXA-51-like_QCD18717.1_A.b.  MNIKALLLITSAIFISACSPYIVTANPNHSASKSDEKAEKIKNLFNEAHTTGVLVIQQGQ
OXA-51-like_QCD22484.1_A.b.  MNIKALLLITSAIFISACSPYIVTANPNHSASKSDEKAEKIKNLFNEAHTTGVLVIQQGQ
OXA-51-like_QRN22730.1_A.b.  MNIKALLLITSAIFISACSPYIVTANPNHSASKSDEKAEKIKNLFNEAHTTGVLVIQQGQ
OXA-51-like_QRN17816.1_A.b.  MNIKALLLITSAIFISACSPYIVTANPNHSASKSDEKAEKIKNLFNEAHTTGVLVIQQGQ
OXA-51-like_QRN09142.1_A.b.  MNIKALLLITSAIFISACSPYIVTANPNHSASKSDEKAEKIKNLFNEAHTTGVLVIQQGQ
OXA-51-like_QRN14361.1_A.b.  MNIKALLLITSAIFISACSPYIVTANPNHSASKSDEKAEKIKNLFNEAHTTGVLVIQQGQ
OXA-51-like_AHB91645.1_A.b.  MNIKALLLITSAIFISACSPYIVTTNPNHSASKSDEKAEKIKNLFNEAHTTGVLVIQQGQ
OXA-51-like_QEI76099.1_A.b.  MNIKALLLITSAIFISACSPYIVTTNPNHSASKSDEKAEKIKNLFNEAHTTGVLVIQQGQ
OXA-51-like_QBY88347.1_A.b.  MNIQALLLITSAIFISACSPYIVSANPNHSASKSDEKAEKIKNLFNEAHTTGVLVIQQGQ
OXA-51-like_ANC35117.1_A.b.  MNIKALLLITSAIFISACSPYIVTANPNHSASKSDEKAEKIKNLFNEAHTTGVLVIQQGQ
OXA-51-like_APJ19826.1_A.b.  MNIKALLLITSAIFISACSPYIVTANPNHSASKSDEKAEKIKNLFNEAHTTGVLVIQQGQ
OXA-51-like_AVE55122.1_A.b.  MNIKALLLITSAIFISACSPYIVTANPNHSASKSDEKAEKIKNLFNEAHTTGVLVIQQGQ
OXA-51-like_AVO92126.1_A.b.  MNIKALLLITSAIFISACSPYIVTANPNHSASKSDEKAEKIKNLFNEAHTTGVLVIQQGQ
OXA-51-like_AXG84775.1_A.b.  MNIKALLLITSAIFISACSPYIVTANPNHSASKSDEKAEKIKNLFNEAHTTGVLVIQQGQ
OXA-51-like_AXX40472.1_A.b.  MNIKALLLITSAIFISACSPYIVTANPNHSASKSDEKAEKIKNLFNEAHTTGVLVIQQGQ
OXA-51-like_AYY89494.1_A.b.  MNIKALLLITSAIFISACSPYIVTANPNHSASKSDEKAEKIKNLFNEAHTTGVLVIQQGQ
OXA-51-like_QFH45392.1_A.b.  MNIKALLLITSAIFISACSPYIVTANPNHSASKSDEKAEKIKNLFNEAHTTGVLVIQQGQ
OXA-51-like_QFY69235.1_A.b.  MNIKALLLITSAIFISACSPYIVTANPNHSASKSDEKAEKIKNLFNEAHTTGVLVIQQGQ
OXA-51-like_QJF27678.1_A.b.  MNIKALLLITSAIFISACSPYIVTANPNHSASKSDEKAEKIKNLFNEAHTTGVLVIQQGQ
OXA-51-like_AML74598.1_A.b.  MNIKALLLITSAIFISACSPYIVTANPNHSASKSDVKAEKIKNLFNEAHTTGVLVIQQGQ
OXA-51-like_AOP62964.1_A.b.  MNIKALLLITSAIFISACSPYIVTANPNHSASKSDVKAEKIKNLFNEAHTTGVLVIQQGQ
OXA-51-like_AFU38004.1_A.b.  MNIKALLLITSAIFISACSPYIVTANPNHSASKSDVKAEKIKNLFNEAHTTGVLVIQQGQ
OXA-51-like_AML67359.1_A.b.  MNIKALLLITSAIFISACSPYIVTANPNHSASKSDVKAEKIKNLFNEAHTTGVLVIQQGQ
OXA-51-like_AML71041.1_A.b.  MNIKALLLITSAIFISACSPYIVTANPNHSASKSDVKAEKIKNLFNEAHTTGVLVIQQGQ
OXA-51-like_AML63873.1_A.b.  MNIKALLLITSAIFISACSPYIVTANPNHSASKSDVKAEKIKNLFNEAHTTGVLVIQQGQ
OXA-51-like_QKK74898.1_A.b.  MNIKALLLITSAIFISACSPYIVTANPNHSASKSDVKAEKIKNLFNEAHTTGVLVIQQGQ
OXA-51-like_QFX70271.1_A.b.  MNIKALLLITSAIFISACSPYIVTANPNHSASKSDVKAEKIKNLFNEAHTTGVLVIQQGQ
OXA-51-like_QEK67468.1_A.b.  MNIKALLLITSAIFISACSPYIVTANPNHSASKSDVKAEKIKNLFNEAHTTGVLVIQQGQ
OXA-51-like_QEK74707.1_A.b.  MNIKALLLITSAIFISACSPYIVTANPNHSASKSDVKAEKIKNLFNEAHTTGVLVIQQGQ
OXA-51-like_QEK71081.1_A.b.  MNIKALLLITSAIFISACSPYIVTANPNHSASKSDVKAEKIKNLFNEAHTTGVLVIQQGQ
OXA-51-like_QFZ58831.1_A.b.  MNIKALLLITSAIFISACSPYIVTANPNHSASKSDVKAEKIKNLFNEAHTTGVLVIQQGQ
OXA-51-like_QAS47153.1_A.b.  MNIKALLLITSAIFISACSPYIVTANPNHSASKSDVKAEKIKNLFNEAHTTGVLVIQQGQ
OXA-51-like_QAS43533.1_A.b.  MNIKALLLITSAIFISACSPYIVTANPNHSASKSDVKAEKIKNLFNEAHTTGVLVIQQGQ
OXA-51-like_QNV16375.1_A.b.  MNIKALLLITSAIFISACSPYIVTANPNHSASKSDVKAEKIKNLFNEAHTTGVLVIQQGQ
OXA-51-like_QNV20321.1_A.b.  MNIKALLLITSAIFISACSPYIVTANPNHSASKSDVKAEKIKNLFNEAHTTGVLVIQQGQ
OXA-51-like_QNV24005.1_A.b.  MNIKALLLITSAIFISACSPYIVTANPNHSASKSDVKAEKIKNLFNEAHTTGVLVIQQGQ
OXA-51-like_QNV27835.1_A.b.  MNIKALLLITSAIFISACSPYIVTANPNHSASKSDVKAEKIKNLFNEAHTTGVLVIQQGQ
OXA-51-like_QNV31585.1_A.b.  MNIKALLLITSAIFISACSPYIVTANPNHSASKSDVKAEKIKNLFNEAHTTGVLVIQQGQ
OXA-51-like_QPN98293.1_A.b.  MNIKALLLITSAIFISACSPYIVTANPNHSASKSDVKAEKIKNLFNEAHTTGVLVIQQGQ
OXA-51-like_QPO01798.1_A.b.  MNIKALLLITSAIFISACSPYIVTANPNHSASKSDVKAEKIKNLFNEAHTTGVLVIQQGQ
OXA-51-like_QQD93954.1_A.b.  MNIKALLLITSAIFISACSPYIVTANPNHSASKSDVKAEKIKNLFNEAHTTGVLVIQQGQ
OXA-51-like_QQD94685.1_A.b.  MNIKALLLITSAIFISACSPYIVTANPNHSASKSDVKAEKIKNLFNEAHTTGVLVIQQGQ
OXA-51-like_QQD98482.1_A.b.  MNIKALLLITSAIFISACSPYIVTANPNHSASKSDVKAEKIKNLFNEAHTTGVLVIQQGQ
OXA-51-like_QQE02276.1_A.b.  MNIKALLLITSAIFISACSPYIVTANPNHSASKSDVKAEKIKNLFNEAHTTGVLVIQQGQ
OXA-51-like_SBS22256.1_A.b.  MNIKALLLITSAIFISACSPYIVTANPNHSASKSDVKAEKIKNLFNEAHTTGVLVIQQGQ
OXA-51-like_AEP06182.1_A.b.  MNIKALLLITSAIFISACSPYIVTANPNHSASKSDVKAEKIKNLFNEAHTTGVLVIQQGQ
```

OXA-51-like subfamily

```
OXA-51-like_ARG02295.1_A.b.  MNIKALLLITSAIFISACSPYIVTANPNHSASKSDVKAEKIKNLFNEAHTTGVLVIQQGQ
OXA-51-like_ARG25110.1_A.b.  MNIKALLLITSAIFISACSPYIVTANPNHSASKSDVKAEKIKNLFNEAHTTGVLVIQQGQ
OXA-51-like_AYC01674.1_A.b.  MNIKALLLITSAIFISACSPYIVTANPNHSASKSDVKAEKIKNLFNEAHTTGVLVIQQGQ
OXA-51-like_ATU49292.1_A.b.  MNIKALLLITSAIFISACSPYIVTANPNHSASKSDVKAEKIKNLFNEAHTTGVLVIQQGQ
OXA-51-like_ATU52990.1_A.b.  MNIKALLLITSAIFISACSPYIVTANPNHSASKSDVKAEKIKNLFNEAHTTGVLVIQQGQ
OXA-51-like_ATU56628.1_A.b.  MNIKALLLITSAIFISACSPYIVTANPNHSASKSDVKAEKIKNLFNEAHTTGVLVIQQGQ
OXA-51-like_AZK38077.1_A.b.  MNIKALLLITSAIFISACSPYIVTANPNHSASKSDVKAEKIKNLFNEAHTTGVLVIQQGQ
OXA-51-like_AZK41733.1_A.b.  MNIKALLLITSAIFISACSPYIVTANPNHSASKSDVKAEKIKNLFNEAHTTGVLVIQQGQ
OXA-51-like_QAS39909.1_A.b.  MNIKALLLITSAIFISACSPYIVTANPNHSASKSDVKAEKIKNLFNEAHTTGVLVIQQGQ
OXA-51-like_QAS33473.1_A.b.  MNIKALLLITSAIFISACSPYIVTANPNHSASKSDVKAEKIKNLFNEAHTTGVLVIQQGQ
OXA-51-like_ACC56872.1_A.b.  MNIKALLLITSAIFISACSPYIVTANPNHSASKSDVKAEKIKNLFNEAHTTGVLVIQQGQ
OXA-51-like_ADX91666.1_A.b.  MNIKALLLITSAIFISACSPYIVTANPNHSASKSDVKAEKIKNLFNEAHTTGVLVIQQGQ
OXA-51-like_ADX92178.1_A.b.  MNIKALLLITSAIFISACSPYIVTANPNHSASKSDVKAEKIKNLFNEAHTTGVLVIQQGQ
OXA-51-like_AGQ10228.1_A.b.  MNIKALLLITSAIFISACSPYIVTANPNHSASKSDVKAEKIKNLFNEAHTTGVLVIQQGQ
OXA-51-like_ALX99780.1_A.b.  MNIKALLLITSAIFISACSPYIVTANPNHSASKSDVKAEKIKNLFNEAHTTGVLVIQQGQ
OXA-51-like_AMC15222.1_A.b.  MNIKALLLITSAIFISACSPYIVTANPNHSASKSDVKAEKIKNLFNEAHTTGVLVIQQGQ
OXA-51-like_ANB88975.1_A.b.  MNIKALLLITSAIFISACSPYIVTANPNHSASKSDVKAEKIKNLFNEAHTTGVLVIQQGQ
OXA-51-like_AOX69607.1_A.b.  MNIKALLLITSAIFISACSPYIVTANPNHSASKSDVKAEKIKNLFNEAHTTGVLVIQQGQ
OXA-51-like_AOX73369.1_A.b.  MNIKALLLITSAIFISACSPYIVTANPNHSASKSDVKAEKIKNLFNEAHTTGVLVIQQGQ
OXA-51-like_AOX77482.1_A.b.  MNIKALLLITSAIFISACSPYIVTANPNHSASKSDVKAEKIKNLFNEAHTTGVLVIQQGQ
OXA-51-like_AOX81661.1_A.b.  MNIKALLLITSAIFISACSPYIVTANPNHSASKSDVKAEKIKNLFNEAHTTGVLVIQQGQ
OXA-51-like_AOX85074.1_A.b.  MNIKALLLITSAIFISACSPYIVTANPNHSASKSDVKAEKIKNLFNEAHTTGVLVIQQGQ
OXA-51-like_AOX88942.1_A.b.  MNIKALLLITSAIFISACSPYIVTANPNHSASKSDVKAEKIKNLFNEAHTTGVLVIQQGQ
OXA-51-like_AOX92779.1_A.b.  MNIKALLLITSAIFISACSPYIVTANPNHSASKSDVKAEKIKNLFNEAHTTGVLVIQQGQ
OXA-51-like_AOX96637.1_A.b.  MNIKALLLITSAIFISACSPYIVTANPNHSASKSDVKAEKIKNLFNEAHTTGVLVIQQGQ
OXA-51-like_APF43961.1_A.b.  MNIKALLLITSAIFISACSPYIVTANPNHSASKSDVKAEKIKNLFNEAHTTGVLVIQQGQ
OXA-51-like_APJ23527.1_A.b.  MNIKALLLITSAIFISACSPYIVTANPNHSASKSDVKAEKIKNLFNEAHTTGVLVIQQGQ
OXA-51-like_APM48724.1_A.b.  MNIKALLLITSAIFISACSPYIVTANPNHSASKSDVKAEKIKNLFNEAHTTGVLVIQQGQ
OXA-51-like_APQ85553.1_A.b.  MNIKALLLITSAIFISACSPYIVTANPNHSASKSDVKAEKIKNLFNEAHTTGVLVIQQGQ
OXA-51-like_APQ93188.1_A.b.  MNIKALLLITSAIFISACSPYIVTANPNHSASKSDVKAEKIKNLFNEAHTTGVLVIQQGQ
OXA-51-like_AQU55455.1_A.b.  MNIKALLLITSAIFISACSPYIVTANPNHSASKSDVKAEKIKNLFNEAHTTGVLVIQQGQ
OXA-51-like_ARF91818.1_A.b.  MNIKALLLITSAIFISACSPYIVTANPNHSASKSDVKAEKIKNLFNEAHTTGVLVIQQGQ
OXA-51-like_ARF95229.1_A.b.  MNIKALLLITSAIFISACSPYIVTANPNHSASKSDVKAEKIKNLFNEAHTTGVLVIQQGQ
OXA-51-like_ARG04169.1_A.b.  MNIKALLLITSAIFISACSPYIVTANPNHSASKSDVKAEKIKNLFNEAHTTGVLVIQQGQ
OXA-51-like_ARG09260.1_A.b.  MNIKALLLITSAIFISACSPYIVTANPNHSASKSDVKAEKIKNLFNEAHTTGVLVIQQGQ
OXA-51-like_ARG14122.1_A.b.  MNIKALLLITSAIFISACSPYIVTANPNHSASKSDVKAEKIKNLFNEAHTTGVLVIQQGQ
OXA-51-like_ARG20812.1_A.b.  MNIKALLLITSAIFISACSPYIVTANPNHSASKSDVKAEKIKNLFNEAHTTGVLVIQQGQ
OXA-51-like_ARG26815.1_A.b.  MNIKALLLITSAIFISACSPYIVTANPNHSASKSDVKAEKIKNLFNEAHTTGVLVIQQGQ
OXA-51-like_ATR88137.1_A.b.  MNIKALLLITSAIFISACSPYIVTANPNHSASKSDVKAEKIKNLFNEAHTTGVLVIQQGQ
OXA-51-like_ATU22923.1_A.b.  MNIKALLLITSAIFISACSPYIVTANPNHSASKSDVKAEKIKNLFNEAHTTGVLVIQQGQ
OXA-51-like_AUG11400.1_A.b.  MNIKALLLITSAIFISACSPYIVTANPNHSASKSDVKAEKIKNLFNEAHTTGVLVIQQGQ
OXA-51-like_AVE45033.1_A.b.  MNIKALLLITSAIFISACSPYIVTANPNHSASKSDVKAEKIKNLFNEAHTTGVLVIQQGQ
OXA-51-like_AVE90526.1_A.b.  MNIKALLLITSAIFISACSPYIVTANPNHSASKSDVKAEKIKNLFNEAHTTGVLVIQQGQ
OXA-51-like_AVO88307.1_A.b.  MNIKALLLITSAIFISACSPYIVTANPNHSASKSDVKAEKIKNLFNEAHTTGVLVIQQGQ
OXA-51-like_AWO15631.1_A.b.  MNIKALLLITSAIFISACSPYIVTANPNHSASKSDVKAEKIKNLFNEAHTTGVLVIQQGQ
OXA-51-like_AWW77770.1_A.b.  MNIKALLLITSAIFISACSPYIVTANPNHSASKSDVKAEKIKNLFNEAHTTGVLVIQQGQ
OXA-51-like_AWW85353.1_A.b.  MNIKALLLITSAIFISACSPYIVTANPNHSASKSDVKAEKIKNLFNEAHTTGVLVIQQGQ
OXA-51-like_AXW91373.1_A.b.  MNIKALLLITSAIFISACSPYIVTANPNHSASKSDVKAEKIKNLFNEAHTTGVLVIQQGQ
OXA-51-like_AXX48901.1_A.b.  MNIKALLLITSAIFISACSPYIVTANPNHSASKSDVKAEKIKNLFNEAHTTGVLVIQQGQ
OXA-51-like_AXX55862.1_A.b.  MNIKALLLITSAIFISACSPYIVTANPNHSASKSDVKAEKIKNLFNEAHTTGVLVIQQGQ
OXA-51-like_QBA07478.1_A.b.  MNIKALLLITSAIFISACSPYIVTANPNHSASKSDVKAEKIKNLFNEAHTTGVLVIQQGQ
OXA-51-like_QBB77223.1_A.b.  MNIKALLLITSAIFISACSPYIVTANPNHSASKSDVKAEKIKNLFNEAHTTGVLVIQQGQ
OXA-51-like_QCH33275.1_A.b.  MNIKALLLITSAIFISACSPYIVTANPNHSASKSDVKAEKIKNLFNEAHTTGVLVIQQGQ
OXA-51-like_QCP15970.1_A.b.  MNIKALLLITSAIFISACSPYIVTANPNHSASKSDVKAEKIKNLFNEAHTTGVLVIQQGQ
OXA-51-like_QCP19079.1_A.b.  MNIKALLLITSAIFISACSPYIVTANPNHSASKSDVKAEKIKNLFNEAHTTGVLVIQQGQ
OXA-51-like_QCP45479.1_A.b.  MNIKALLLITSAIFISACSPYIVTANPNHSASKSDVKAEKIKNLFNEAHTTGVLVIQQGQ
OXA-51-like_QDR93660.1_A.b.  MNIKALLLITSAIFISACSPYIVTANPNHSASKSDVKAEKIKNLFNEAHTTGVLVIQQGQ
OXA-51-like_QEY27958.1_A.b.  MNIKALLLITSAIFISACSPYIVTANPNHSASKSDVKAEKIKNLFNEAHTTGVLVIQQGQ
OXA-51-like_QIX30833.1_A.b.  MNIKALLLITSAIFISACSPYIVTANPNHSASKSDVKAEKIKNLFNEAHTTGVLVIQQGQ
OXA-51-like_QIX34674.1_A.b.  MNIKALLLITSAIFISACSPYIVTANPNHSASKSDVKAEKIKNLFNEAHTTGVLVIQQGQ
OXA-51-like_QIX38631.1_A.b.  MNIKALLLITSAIFISACSPYIVTANPNHSASKSDVKAEKIKNLFNEAHTTGVLVIQQGQ
OXA-51-like_QIX42205.1_A.b.  MNIKALLLITSAIFISACSPYIVTANPNHSASKSDVKAEKIKNLFNEAHTTGVLVIQQGQ
OXA-51-like_QIX46166.1_A.b.  MNIKALLLITSAIFISACSPYIVTANPNHSASKSDVKAEKIKNLFNEAHTTGVLVIQQGQ
OXA-51-like_QJG69125.1_A.b.  MNIKALLLITSAIFISACSPYIVTANPNHSASKSDVKAEKIKNLFNEAHTTGVLVIQQGQ
OXA-51-like_QJG72958.1_A.b.  MNIKALLLITSAIFISACSPYIVTANPNHSASKSDVKAEKIKNLFNEAHTTGVLVIQQGQ
OXA-51-like_QJG88231.1_A.b.  MNIKALLLITSAIFISACSPYIVTANPNHSASKSDVKAEKIKNLFNEAHTTGVLVIQQGQ
OXA-51-like_QJG91989.1_A.b.  MNIKALLLITSAIFISACSPYIVTANPNHSASKSDVKAEKIKNLFNEAHTTGVLVIQQGQ
OXA-51-like_QJG95593.1_A.b.  MNIKALLLITSAIFISACSPYIVTANPNHSASKSDVKAEKIKNLFNEAHTTGVLVIQQGQ
OXA-51-like_QJG99403.1_A.b.  MNIKALLLITSAIFISACSPYIVTANPNHSASKSDVKAEKIKNLFNEAHTTGVLVIQQGQ
OXA-51-like_QJH07093.1_A.b.  MNIKALLLITSAIFISACSPYIVTANPNHSASKSDVKAEKIKNLFNEAHTTGVLVIQQGQ
OXA-51-like_QJH10219.1_A.b.  MNIKALLLITSAIFISACSPYIVTANPNHSASKSDVKAEKIKNLFNEAHTTGVLVIQQGQ
OXA-51-like_QJH14483.1_A.b.  MNIKALLLITSAIFISACSPYIVTANPNHSASKSDVKAEKIKNLFNEAHTTGVLVIQQGQ
OXA-51-like_QJH17776.1_A.b.  MNIKALLLITSAIFISACSPYIVTANPNHSASKSDVKAEKIKNLFNEAHTTGVLVIQQGQ
OXA-51-like_QJP31173.1_A.b.  MNIKALLLITSAIFISACSPYIVTANPNHSASKSDVKAEKIKNLFNEAHTTGVLVIQQGQ
OXA-51-like_QLA73304.1_A.b.  MNIKALLLITSAIFISACSPYIVTANPNHSASKSDVKAEKIKNLFNEAHTTGVLVIQQGQ
OXA-51-like_QNV12531.1_A.b.  MNIKALLLITSAIFISACSPYIVTANPNHSASKSDVKAEKIKNLFNEAHTTGVLVIQQGQ
OXA-51-like_QAS93855.1_A.b.  MNIKALLLITSAIFISACSPYIVTANPNHSASKSDVKAEKIKNLFNEAHTTGVLVIQQGQ
OXA-51-like_QAT01315.1_A.b.  MNIKALLLITSAIFISACSPYIVTANPNHSASKSDVKAEKIKNLFNEAHTTGVLVIQQGQ
OXA-51-like_QAS97806.1_A.b.  MNIKALLLITSAIFISACSPYIVTANPNHSASKSDVKAEKIKNLFNEAHTTGVLVIQQGQ
OXA-51-like_QAT04873.1_A.b.  MNIKALLLITSAIFISACSPYIVTANPNHSASKSDVKAEKIKNLFNEAHTTGVLVIQQGQ
OXA-51-like_QEE57116.1_A.b.  MNIKALLLITSAIFISACSPYIVTANPNHSASKSDVKAEKIKNLFNEAHTTGVLVIQQGQ
OXA-51-like_ADX03652.1_A.b.  MNIKALLLITSAIFISACSPYIVTANPNHSASKSDVKAEKIKNLFNEAHTTGVLVIQQGQ
OXA-51-like_QCO82814.1_A.b.  MNIKALLLITSAIFISACSPYIVTANPNHSASKSDVKAEKIKNLFNEAHTTGVLVIQQGQ
OXA-51-like_AJF81571.1_A.b.  MNIKALLLITSAIFISACSPYIVTANPNHSASKSDDKAEKIKNLFNEAHTTGVLVIHQGQ
OXA-51-like_ATP87403.1_A.b.  MNIKALLLITSAIFISACSPYIVTANPNHSASKSDDKAEKIKNLFNEAHTTGVLVIHQGQ
OXA-51-like_AKA32028.1_A.b.  MNIKALLLITSAIFISACSPYIVTANPNHSASKSDDKAEKIKNLFNEAHTTGVLVIHQGQ
OXA-51-like_ALJ88113.1_A.b.  MNIKALLLITSAIFISACSPYIVTANPNHSASKSDDKAEKIKNLFNEAHTTGVLVIHQGQ
OXA-51-like_ARG31555.1_A.b.  MNIKALLLITSAIFISACSPYIVTANPNHSASKSDDKAEKIKNLFNEAHTTGVLVIHQGQ
OXA-51-like_ASF77094.1_A.b.  MNIKALLLITSAIFISACSPYIVTANPNHSASKSDDKAEKIKNLFNEAHTTGVLVIHQGQ
OXA-51-like_AVN14185.1_A.b.  MNIKALLLITSAIFISACSPYIVTANPNHSASKSDDKAEKIKNLFNEAHTTGVLVIHQGQ
OXA-51-like_AVN25165.1_A.b.  MNIKALLLITSAIFISACSPYIVTANPNHSASKSDDKAEKIKNLFNEAHTTGVLVIHQGQ
OXA-51-like_AXB14467.1_A.b.  MNIKALLLITSAIFISACSPYIVTANPNHSASKSDDKAEKIKNLFNEAHTTGVLVIHQGQ
OXA-51-like_QLG79253.1_A.b.  MNIKALLLITSAIFISACSPYIVTANPNHSASKSDDKAEKIKNLFNEAHTTGVLVIHQGQ
OXA-51-like_QQB68144.1_A.b.  MNIKALLLITSAIFISACSPYIVTANPNHSASKSDDKAEKIKNLFNEAHTTGVLVIHQGQ
OXA-51-like_SQI55469.1_A.b.  MNIKALLLITSAIFISACSPYIVTANPNHSASKSDDKAEKIKNLFNEAHTTGVLVIHQGQ
OXA-51-like_QJH03197.1_A.b.  MNIKALLLITSAIFISACSPYIVTANPNHSASKSDDKAEKIKNLFNEAHTTGVLVIHQGQ
OXA-51-like_QHB90233.1_A.b.  MNIKALLLITSAIFISACSPYIVTANPNHSASKSDDKAEKIKNLFNEAHTTGVLVIHQGQ
OXA-51-like_BBR73296.1_A.b.  MNIKALLLITSAIFISACSPYIVTANPNHSASKSDDKAEKIKNLFNEAHTTGVLVIHQGQ
                        acc
```

OXA-51-like subfamily

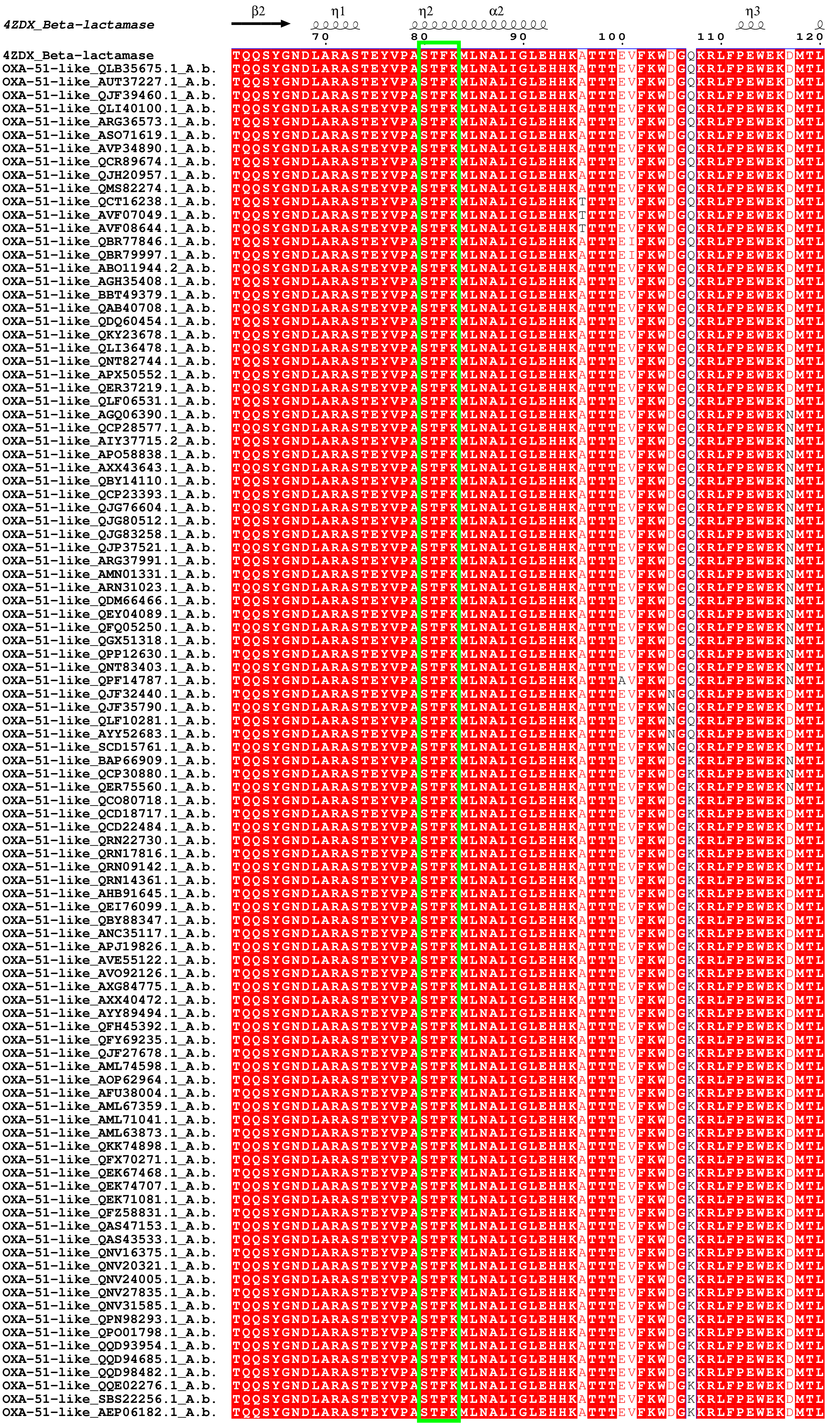

OXA-51-like subfamily

```
OXA-51-like_ARG02295.1_A.b. TQQSYGNDLARASTEYVPASTFKMLNALIGLEHHKATTTEVFKWDGKKRLFPEWEKDMTL
OXA-51-like_ARG25110.1_A.b. TQQSYGNDLARASTEYVPASTFKMLNALIGLEHHKATTTEVFKWDGKKRLFPEWEKDMTL
OXA-51-like_AYC01674.1_A.b. TQQSYGNDLARASTEYVPASTFKMLNALIGLEHHKATTTEVFKWDGKKRLFPEWEKDMTL
OXA-51-like_ATU49292.1_A.b. TQQSYGNDLARASTEYVPASTFKMLNALIGLEHHKATTTEVFKWDGKKRLFPEWEKDMTL
OXA-51-like_ATU52990.1_A.b. TQQSYGNDLARASTEYVPASTFKMLNALIGLEHHKATTTEVFKWDGKKRLFPEWEKDMTL
OXA-51-like_ATU56628.1_A.b. TQQSYGNDLARASTEYVPASTFKMLNALIGLEHHKATTTEVFKWDGKKRLFPEWEKDMTL
OXA-51-like_AZK38077.1_A.b. TQQSYGNDLARASTEYVPASTFKMLNALIGLEHHKATTTEVFKWDGKKRLFPEWEKDMTL
OXA-51-like_AZK41733.1_A.b. TQQSYGNDLARASTEYVPASTFKMLNALIGLEHHKATTTEVFKWDGKKRLFPEWEKDMTL
OXA-51-like_QAS39909.1_A.b. TQQSYGNDLARASTEYVPASTFKMLNALIGLEHHKATTTEVFKWDGKKRLFPEWEKDMTL
OXA-51-like_QAS33473.1_A.b. TQQSYGNDLARASTEYVPASTFKMLNALIGLEHHKATTTEVFKWDGKKRLFPEWEKDMTL
OXA-51-like_ACC56872.1_A.b. TQQSYGNDLARASTEYVPASTFKMLNALIGLEHHKATTTEVFKWDGKKRLFPEWEKDMTL
OXA-51-like_ADX91666.1_A.b. TQQSYGNDLARASTEYVPASTFKMLNALIGLEHHKATTTEVFKWDGKKRLFPEWEKDMTL
OXA-51-like_ADX92178.1_A.b. TQQSYGNDLARASTEYVPASTFKMLNALIGLEHHKATTTEVFKWDGKKRLFPEWEKDMTL
OXA-51-like_AGQ10228.1_A.b. TQQSYGNDLARASTEYVPASTFKMLNALIGLEHHKATTTEVFKWDGKKRLFPEWEKDMTL
OXA-51-like_ALX99780.1_A.b. TQQSYGNDLARASTEYVPASTFKMLNALIGLEHHKATTTEVFKWDGKKRLFPEWEKDMTL
OXA-51-like_AMC15222.1_A.b. TQQSYGNDLARASTEYVPASTFKMLNALIGLEHHKATTTEVFKWDGKKRLFPEWEKDMTL
OXA-51-like_ANB88975.1_A.b. TQQSYGNDLARASTEYVPASTFKMLNALIGLEHHKATTTEVFKWDGKKRLFPEWEKDMTL
OXA-51-like_AOX69607.1_A.b. TQQSYGNDLARASTEYVPASTFKMLNALIGLEHHKATTTEVFKWDGKKRLFPEWEKDMTL
OXA-51-like_AOX73369.1_A.b. TQQSYGNDLARASTEYVPASTFKMLNALIGLEHHKATTTEVFKWDGKKRLFPEWEKDMTL
OXA-51-like_AOX77482.1_A.b. TQQSYGNDLARASTEYVPASTFKMLNALIGLEHHKATTTEVFKWDGKKRLFPEWEKDMTL
OXA-51-like_AOX81661.1_A.b. TQQSYGNDLARASTEYVPASTFKMLNALIGLEHHKATTTEVFKWDGKKRLFPEWEKDMTL
OXA-51-like_AOX85074.1_A.b. TQQSYGNDLARASTEYVPASTFKMLNALIGLEHHKATTTEVFKWDGKKRLFPEWEKDMTL
OXA-51-like_AOX88942.1_A.b. TQQSYGNDLARASTEYVPASTFKMLNALIGLEHHKATTTEVFKWDGKKRLFPEWEKDMTL
OXA-51-like_AOX92779.1_A.b. TQQSYGNDLARASTEYVPASTFKMLNALIGLEHHKATTTEVFKWDGKKRLFPEWEKDMTL
OXA-51-like_AOX96637.1_A.b. TQQSYGNDLARASTEYVPASTFKMLNALIGLEHHKATTTEVFKWDGKKRLFPEWEKDMTL
OXA-51-like_APF43961.1_A.b. TQQSYGNDLARASTEYVPASTFKMLNALIGLEHHKATTTEVFKWDGKKRLFPEWEKDMTL
OXA-51-like_APJ23527.1_A.b. TQQSYGNDLARASTEYVPASTFKMLNALIGLEHHKATTTEVFKWDGKKRLFPEWEKDMTL
OXA-51-like_APM48724.1_A.b. TQQSYGNDLARASTEYVPASTFKMLNALIGLEHHKATTTEVFKWDGKKRLFPEWEKDMTL
OXA-51-like_APQ85553.1_A.b. TQQSYGNDLARASTEYVPASTFKMLNALIGLEHHKATTTEVFKWDGKKRLFPEWEKDMTL
OXA-51-like_APQ93188.1_A.b. TQQSYGNDLARASTEYVPASTFKMLNALIGLEHHKATTTEVFKWDGKKRLFPEWEKDMTL
OXA-51-like_AQU55455.1_A.b. TQQSYGNDLARASTEYVPASTFKMLNALIGLEHHKATTTEVFKWDGKKRLFPEWEKDMTL
OXA-51-like_ARF91818.1_A.b. TQQSYGNDLARASTEYVPASTFKMLNALIGLEHHKATTTEVFKWDGKKRLFPEWEKDMTL
OXA-51-like_ARF95229.1_A.b. TQQSYGNDLARASTEYVPASTFKMLNALIGLEHHKATTTEVFKWDGKKRLFPEWEKDMTL
OXA-51-like_ARG04169.1_A.b. TQQSYGNDLARASTEYVPASTFKMLNALIGLEHHKATTTEVFKWDGKKRLFPEWEKDMTL
OXA-51-like_ARG09260.1_A.b. TQQSYGNDLARASTEYVPASTFKMLNALIGLEHHKATTTEVFKWDGKKRLFPEWEKDMTL
OXA-51-like_ARG14122.1_A.b. TQQSYGNDLARASTEYVPASTFKMLNALIGLEHHKATTTEVFKWDGKKRLFPEWEKDMTL
OXA-51-like_ARG20812.1_A.b. TQQSYGNDLARASTEYVPASTFKMLNALIGLEHHKATTTEVFKWDGKKRLFPEWEKDMTL
OXA-51-like_ARG26815.1_A.b. TQQSYGNDLARASTEYVPASTFKMLNALIGLEHHKATTTEVFKWDGKKRLFPEWEKDMTL
OXA-51-like_ATR88137.1_A.b. TQQSYGNDLARASTEYVPASTFKMLNALIGLEHHKATTTEVFKWDGKKRLFPEWEKDMTL
OXA-51-like_ATU22923.1_A.b. TQQSYGNDLARASTEYVPASTFKMLNALIGLEHHKATTTEVFKWDGKKRLFPEWEKDMTL
OXA-51-like_AUG11400.1_A.b. TQQSYGNDLARASTEYVPASTFKMLNALIGLEHHKATTTEVFKWDGKKRLFPEWEKDMTL
OXA-51-like_AVE45033.1_A.b. TQQSYGNDLARASTEYVPASTFKMLNALIGLEHHKATTTEVFKWDGKKRLFPEWEKDMTL
OXA-51-like_AVE90526.1_A.b. TQQSYGNDLARASTEYVPASTFKMLNALIGLEHHKATTTEVFKWDGKKRLFPEWEKDMTL
OXA-51-like_AVO88307.1_A.b. TQQSYGNDLARASTEYVPASTFKMLNALIGLEHHKATTTEVFKWDGKKRLFPEWEKDMTL
OXA-51-like_AWO15631.1_A.b. TQQSYGNDLARASTEYVPASTFKMLNALIGLEHHKATTTEVFKWDGKKRLFPEWEKDMTL
OXA-51-like_AWW77770.1_A.b. TQQSYGNDLARASTEYVPASTFKMLNALIGLEHHKATTTEVFKWDGKKRLFPEWEKDMTL
OXA-51-like_AWW85353.1_A.b. TQQSYGNDLARASTEYVPASTFKMLNALIGLEHHKATTTEVFKWDGKKRLFPEWEKDMTL
OXA-51-like_AXW91373.1_A.b. TQQSYGNDLARASTEYVPASTFKMLNALIGLEHHKATTTEVFKWDGKKRLFPEWEKDMTL
OXA-51-like_AXX48901.1_A.b. TQQSYGNDLARASTEYVPASTFKMLNALIGLEHHKATTTEVFKWDGKKRLFPEWEKDMTL
OXA-51-like_AXX55862.1_A.b. TQQSYGNDLARASTEYVPASTFKMLNALIGLEHHKATTTEVFKWDGKKRLFPEWEKDMTL
OXA-51-like_QBA07478.1_A.b. TQQSYGNDLARASTEYVPASTFKMLNALIGLEHHKATTTEVFKWDGKKRLFPEWEKDMTL
OXA-51-like_QBB77223.1_A.b. TQQSYGNDLARASTEYVPASTFKMLNALIGLEHHKATTTEVFKWDGKKRLFPEWEKDMTL
OXA-51-like_QCH33275.1_A.b. TQQSYGNDLARASTEYVPASTFKMLNALIGLEHHKATTTEVFKWDGKKRLFPEWEKDMTL
OXA-51-like_QCP15970.1_A.b. TQQSYGNDLARASTEYVPASTFKMLNALIGLEHHKATTTEVFKWDGKKRLFPEWEKDMTL
OXA-51-like_QCP19079.1_A.b. TQQSYGNDLARASTEYVPASTFKMLNALIGLEHHKATTTEVFKWDGKKRLFPEWEKDMTL
OXA-51-like_QCP45479.1_A.b. TQQSYGNDLARASTEYVPASTFKMLNALIGLEHHKATTTEVFKWDGKKRLFPEWEKDMTL
OXA-51-like_QDR93660.1_A.b. TQQSYGNDLARASTEYVPASTFKMLNALIGLEHHKATTTEVFKWDGKKRLFPEWEKDMTL
OXA-51-like_QEY27958.1_A.b. TQQSYGNDLARASTEYVPASTFKMLNALIGLEHHKATTTEVFKWDGKKRLFPEWEKDMTL
OXA-51-like_QIX30833.1_A.b. TQQSYGNDLARASTEYVPASTFKMLNALIGLEHHKATTTEVFKWDGKKRLFPEWEKDMTL
OXA-51-like_QIX34674.1_A.b. TQQSYGNDLARASTEYVPASTFKMLNALIGLEHHKATTTEVFKWDGKKRLFPEWEKDMTL
OXA-51-like_QIX38631.1_A.b. TQQSYGNDLARASTEYVPASTFKMLNALIGLEHHKATTTEVFKWDGKKRLFPEWEKDMTL
OXA-51-like_QIX42205.1_A.b. TQQSYGNDLARASTEYVPASTFKMLNALIGLEHHKATTTEVFKWDGKKRLFPEWEKDMTL
OXA-51-like_QIX46166.1_A.b. TQQSYGNDLARASTEYVPASTFKMLNALIGLEHHKATTTEVFKWDGKKRLFPEWEKDMTL
OXA-51-like_QJG69125.1_A.b. TQQSYGNDLARASTEYVPASTFKMLNALIGLEHHKATTTEVFKWDGKKRLFPEWEKDMTL
OXA-51-like_QJG72958.1_A.b. TQQSYGNDLARASTEYVPASTFKMLNALIGLEHHKATTTEVFKWDGKKRLFPEWEKDMTL
OXA-51-like_QJG88231.1_A.b. TQQSYGNDLARASTEYVPASTFKMLNALIGLEHHKATTTEVFKWDGKKRLFPEWEKDMTL
OXA-51-like_QJG91989.1_A.b. TQQSYGNDLARASTEYVPASTFKMLNALIGLEHHKATTTEVFKWDGKKRLFPEWEKDMTL
OXA-51-like_QJG95593.1_A.b. TQQSYGNDLARASTEYVPASTFKMLNALIGLEHHKATTTEVFKWDGKKRLFPEWEKDMTL
OXA-51-like_QJG99403.1_A.b. TQQSYGNDLARASTEYVPASTFKMLNALIGLEHHKATTTEVFKWDGKKRLFPEWEKDMTL
OXA-51-like_QJH07093.1_A.b. TQQSYGNDLARASTEYVPASTFKMLNALIGLEHHKATTTEVFKWDGKKRLFPEWEKDMTL
OXA-51-like_QJH10219.1_A.b. TQQSYGNDLARASTEYVPASTFKMLNALIGLEHHKATTTEVFKWDGKKRLFPEWEKDMTL
OXA-51-like_QJH14483.1_A.b. TQQSYGNDLARASTEYVPASTFKMLNALIGLEHHKATTTEVFKWDGKKRLFPEWEKDMTL
OXA-51-like_QJH17776.1_A.b. TQQSYGNDLARASTEYVPASTFKMLNALIGLEHHKATTTEVFKWDGKKRLFPEWEKDMTL
OXA-51-like_QJP31173.1_A.b. TQQSYGNDLARASTEYVPASTFKMLNALIGLEHHKATTTEVFKWDGKKRLFPEWEKDMTL
OXA-51-like_QLA73304.1_A.b. TQQSYGNDLARASTEYVPASTFKMLNALIGLEHHKATTTEVFKWDGKKRLFPEWEKDMTL
OXA-51-like_QNV12531.1_A.b. TQQSYGNDLARASTEYVPASTFKMLNALIGLEHHKATTTEVFKWDGKKRLFPEWEKDMTL
OXA-51-like_QAS93855.1_A.b. TQQSYGNDLARASTEYVPASTFKMLNALIGLEHHKATTTEVFKWDGKKRLFPEWEKDMTL
OXA-51-like_QAT01315.1_A.b. TQQSYGNDLARASTEYVPASTFKMLNALIGLEHHKATTTEVFKWDGKKRLFPEWEKDMTL
OXA-51-like_QAS97806.1_A.b. TQQSYGNDLARASTEYVPASTFKMLNALIGLEHHKATTTEVFKWDGKKRLFPEWEKDMTL
OXA-51-like_QAT04873.1_A.b. TQQSYGNDLARASTEYVPASTFKMLNALIGLEHHKATTTEVFKWDGKKRLFPEWEKDMTL
OXA-51-like_QEE57116.1_A.b. TQQSYGNDLARASTEYVPASTFKMLNALIGLEHHKATTTEVFKWDGKKRLFPEWEKDMTL
OXA-51-like_ADX03652.1_A.b. TQQSYGNDLARASTEYVPASTFKMLNALIGLEHHKATTTEVFKWDGKKRLFPEWEKDMTL
OXA-51-like_QCO82814.1_A.b. TQQSYGNDLARASTEYVPASTFKMLNALIGLEHHKATTTEVFKWDGKKRLFPEWEKDMTL
OXA-51-like_AJF81571.1_A.b. TQQSYGNDLARASTEYVPASTFKMLNALIGLEHHKATTTEVFKWDGEKRLFPEWEKNMTL
OXA-51-like_ATP87403.1_A.b. TQQSYGNDLARASTEYVPASTFKMLNALIGLEHHKATTTEVFKWDGEKRLFPEWEKNMTL
OXA-51-like_AKA32028.1_A.b. TQQSYGNDLARASTEYVPASTFKMLNALIGLEHHKATTTEVFKWDGEKRLFPEWEKNMTL
OXA-51-like_ALJ88113.1_A.b. TQQSYGNDLARASTEYVPASTFKMLNALIGLEHHKATTTEVFKWDGEKRLFPEWEKNMTL
OXA-51-like_ARG31555.1_A.b. TQQSYGNDLARASTEYVPASTFKMLNALIGLEHHKATTTEVFKWDGEKRLFPEWEKNMTL
OXA-51-like_ASF77094.1_A.b. TQQSYGNDLARASTEYVPASTFKMLNALIGLEHHKATTTEVFKWDGEKRLFPEWEKNMTL
OXA-51-like_AVN14185.1_A.b. TQQSYGNDLARASTEYVPASTFKMLNALIGLEHHKATTTEVFKWDGEKRLFPEWEKNMTL
OXA-51-like_AVN25165.1_A.b. TQQSYGNDLARASTEYVPASTFKMLNALIGLEHHKATTTEVFKWDGEKRLFPEWEKNMTL
OXA-51-like_AXB14467.1_A.b. TQQSYGNDLARASTEYVPASTFKMLNALIGLEHHKATTTEVFKWDGEKRLFPEWEKNMTL
OXA-51-like_QLG79253.1_A.b. TQQSYGNDLARASTEYVPASTFKMLNALIGLEHHKATTTEVFKWDGEKRLFPEWEKNMTL
OXA-51-like_QQB68144.1_A.b. TQQSYGNDLARASTEYVPASTFKMLNALIGLEHHKATTTEVFKWDGEKRLFPEWEKNMTL
OXA-51-like_SQI55469.1_A.b. TQQSYGNDLARASTEYVPASTFKMLNALIGLEHHKATTTEVFKWDGEKRLFPEWEKNMTL
OXA-51-like_QJH03197.1_A.b. TQQSYGNDLARASTEYVPASTFKMLNALIGLEHHKATTTEVFKWDGEKRLFPEWEKNMTL
OXA-51-like_QHB90233.1_A.b. TQQSYGNDLARASTEYVPASTFKMLNALIGLEHHKATTTEVFKWDGQKRLFPEWEKNMTL
OXA-51-like_BBR73296.1_A.b. TQQSYGNDLARASTEYVPASTFKMLNALIGLEHHKATTTEIFKWDGQKRLFPEWEKDMTL
                       acc
```

OXA-51-like subfamily

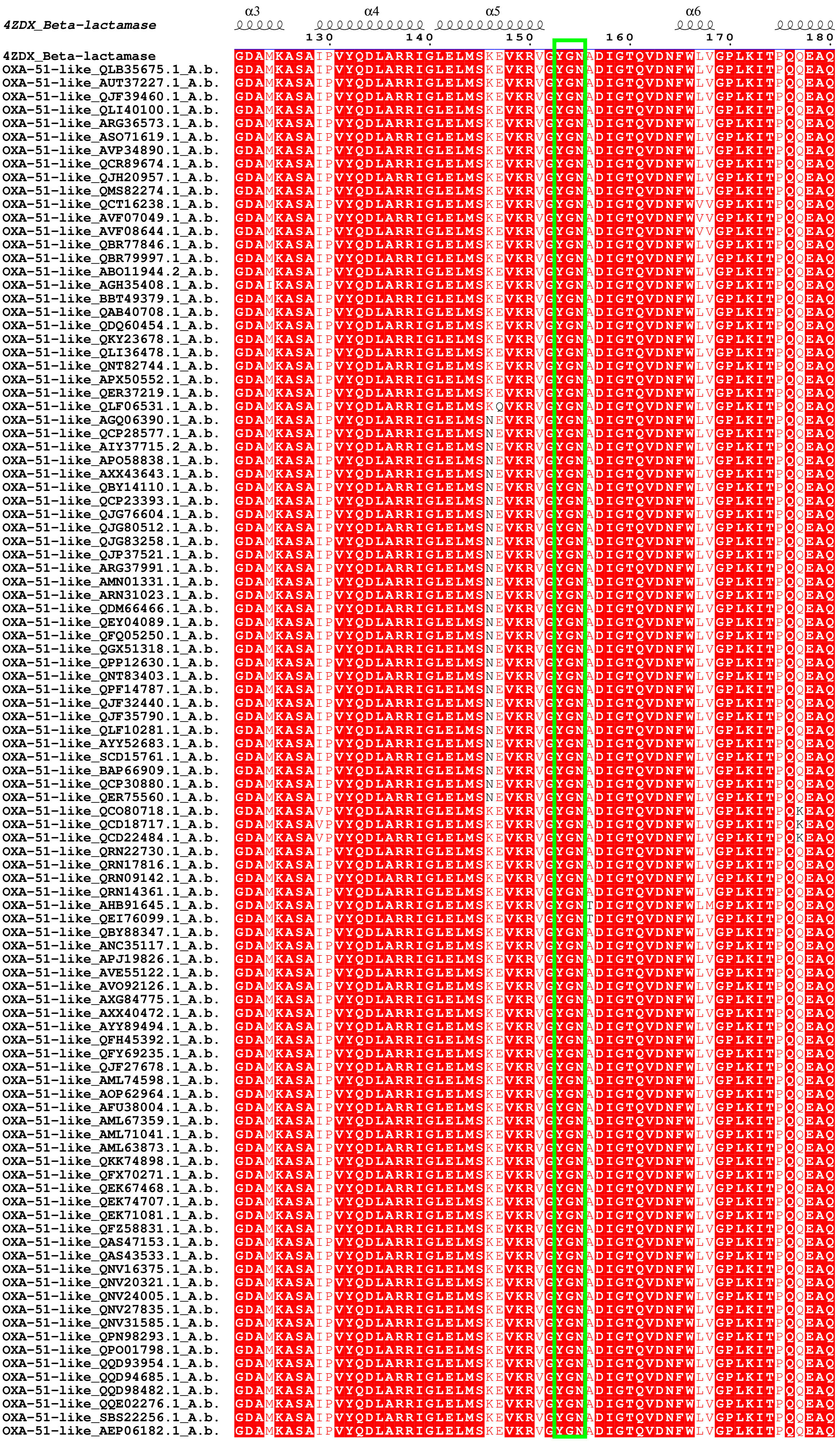

OXA-51-like subfamily

```
OXA-51-like_ARG02295.1_A.b. GDAMKASAIPVYQDLARRIGLELMSKEVKRVGYGNADIGTQVDNFWLVGPLKITPQQEAQ
OXA-51-like_ARG25110.1_A.b. GDAMKASAIPVYQDLARRIGLELMSKEVKRVGYGNADIGTQVDNFWLVGPLKITPQQEAQ
OXA-51-like_AYC01674.1_A.b. GDAMKASAIPVYQDLARRIGLELMSKEVKRVGYGNADIGTQVDNFWLVGPLKITPQQEAQ
OXA-51-like_ATU49292.1_A.b. GDAMKASAIPVYQDLARRIGLELMSKEVKRVGYGNADIGTQVDNFWLVGPLKITPQQEAQ
OXA-51-like_ATU52990.1_A.b. GDAMKASAIPVYQDLARRIGLELMSKEVKRVGYGNADIGTQVDNFWLVGPLKITPQQEAQ
OXA-51-like_ATU56628.1_A.b. GDAMKASAIPVYQDLARRIGLELMSKEVKRVGYGNADIGTQVDNFWLVGPLKITPQQEAQ
OXA-51-like_AZK38077.1_A.b. GDAMKASAIPVYQDLARRIGLELMSKEVKRVGYGNADIGTQVDNFWLVGPLKITPQQEAQ
OXA-51-like_AZK41733.1_A.b. GDAMKASAIPVYQDLARRIGLELMSKEVKRVGYGNADIGTQVDNFWLVGPLKITPQQEAQ
OXA-51-like_QAS39909.1_A.b. GDAMKASAIPVYQDLARRIGLELMSKEVKRVGYGNADIGTQVDNFWLVGPLKITPQQEAQ
OXA-51-like_QAS33473.1_A.b. GDAMKASAIPVYQDLARRIGLELMSKEVKRVGYGNADIGTQVDNFWLVGPLKITPQQEAQ
OXA-51-like_ACC56872.1_A.b. GDAMKASAIPVYQDLARRIGLELMSKEVKRVGYGNADIGTQVDNFWLVGPLKITPQQEAQ
OXA-51-like_ADX91666.1_A.b. GDAMKASAIPVYQDLARRIGLELMSKEVKRVGYGNADIGTQVDNFWLVGPLKITPQQEAQ
OXA-51-like_ADX92178.1_A.b. GDAMKASAIPVYQDLARRIGLELMSKEVKRVGYGNADIGTQVDNFWLVGPLKITPQQEAQ
OXA-51-like_AGQ10228.1_A.b. GDAMKASAIPVYQDLARRIGLELMSKEVKRVGYGNADIGTQVDNFWLVGPLKITPQQEAQ
OXA-51-like_ALX99780.1_A.b. GDAMKASAIPVYQDLARRIGLELMSKEVKRVGYGNADIGTQVDNFWLVGPLKITPQQEAQ
OXA-51-like_AMC15222.1_A.b. GDAMKASAIPVYQDLARRIGLELMSKEVKRVGYGNADIGTQVDNFWLVGPLKITPQQEAQ
OXA-51-like_ANB88975.1_A.b. GDAMKASAIPVYQDLARRIGLELMSKEVKRVGYGNADIGTQVDNFWLVGPLKITPQQEAQ
OXA-51-like_AOX69607.1_A.b. GDAMKASAIPVYQDLARRIGLELMSKEVKRVGYGNADIGTQVDNFWLVGPLKITPQQEAQ
OXA-51-like_AOX73369.1_A.b. GDAMKASAIPVYQDLARRIGLELMSKEVKRVGYGNADIGTQVDNFWLVGPLKITPQQEAQ
OXA-51-like_AOX77482.1_A.b. GDAMKASAIPVYQDLARRIGLELMSKEVKRVGYGNADIGTQVDNFWLVGPLKITPQQEAQ
OXA-51-like_AOX81661.1_A.b. GDAMKASAIPVYQDLARRIGLELMSKEVKRVGYGNADIGTQVDNFWLVGPLKITPQQEAQ
OXA-51-like_AOX85074.1_A.b. GDAMKASAIPVYQDLARRIGLELMSKEVKRVGYGNADIGTQVDNFWLVGPLKITPQQEAQ
OXA-51-like_AOX88942.1_A.b. GDAMKASAIPVYQDLARRIGLELMSKEVKRVGYGNADIGTQVDNFWLVGPLKITPQQEAQ
OXA-51-like_AOX92779.1_A.b. GDAMKASAIPVYQDLARRIGLELMSKEVKRVGYGNADIGTQVDNFWLVGPLKITPQQEAQ
OXA-51-like_AOX96637.1_A.b. GDAMKASAIPVYQDLARRIGLELMSKEVKRVGYGNADIGTQVDNFWLVGPLKITPQQEAQ
OXA-51-like_APF43961.1_A.b. GDAMKASAIPVYQDLARRIGLELMSKEVKRVGYGNADIGTQVDNFWLVGPLKITPQQEAQ
OXA-51-like_APJ23527.1_A.b. GDAMKASAIPVYQDLARRIGLELMSKEVKRVGYGNADIGTQVDNFWLVGPLKITPQQEAQ
OXA-51-like_APM48724.1_A.b. GDAMKASAIPVYQDLARRIGLELMSKEVKRVGYGNADIGTQVDNFWLVGPLKITPQQEAQ
OXA-51-like_APQ85553.1_A.b. GDAMKASAIPVYQDLARRIGLELMSKEVKRVGYGNADIGTQVDNFWLVGPLKITPQQEAQ
OXA-51-like_APQ93188.1_A.b. GDAMKASAIPVYQDLARRIGLELMSKEVKRVGYGNADIGTQVDNFWLVGPLKITPQQEAQ
OXA-51-like_AQU55455.1_A.b. GDAMKASAIPVYQDLARRIGLELMSKEVKRVGYGNADIGTQVDNFWLVGPLKITPQQEAQ
OXA-51-like_ARF91818.1_A.b. GDAMKASAIPVYQDLARRIGLELMSKEVKRVGYGNADIGTQVDNFWLVGPLKITPQQEAQ
OXA-51-like_ARF95229.1_A.b. GDAMKASAIPVYQDLARRIGLELMSKEVKRVGYGNADIGTQVDNFWLVGPLKITPQQEAQ
OXA-51-like_ARG04169.1_A.b. GDAMKASAIPVYQDLARRIGLELMSKEVKRVGYGNADIGTQVDNFWLVGPLKITPQQEAQ
OXA-51-like_ARG09260.1_A.b. GDAMKASAIPVYQDLARRIGLELMSKEVKRVGYGNADIGTQVDNFWLVGPLKITPQQEAQ
OXA-51-like_ARG14122.1_A.b. GDAMKASAIPVYQDLARRIGLELMSKEVKRVGYGNADIGTQVDNFWLVGPLKITPQQEAQ
OXA-51-like_ARG20812.1_A.b. GDAMKASAIPVYQDLARRIGLELMSKEVKRVGYGNADIGTQVDNFWLVGPLKITPQQEAQ
OXA-51-like_ARG26815.1_A.b. GDAMKASAIPVYQDLARRIGLELMSKEVKRVGYGNADIGTQVDNFWLVGPLKITPQQEAQ
OXA-51-like_ATR88137.1_A.b. GDAMKASAIPVYQDLARRIGLELMSKEVKRVGYGNADIGTQVDNFWLVGPLKITPQQEAQ
OXA-51-like_ATU22923.1_A.b. GDAMKASAIPVYQDLARRIGLELMSKEVKRVGYGNADIGTQVDNFWLVGPLKITPQQEAQ
OXA-51-like_AUG11400.1_A.b. GDAMKASAIPVYQDLARRIGLELMSKEVKRVGYGNADIGTQVDNFWLVGPLKITPQQEAQ
OXA-51-like_AVE45033.1_A.b. GDAMKASAIPVYQDLARRIGLELMSKEVKRVGYGNADIGTQVDNFWLVGPLKITPQQEAQ
OXA-51-like_AVE90526.1_A.b. GDAMKASAIPVYQDLARRIGLELMSKEVKRVGYGNADIGTQVDNFWLVGPLKITPQQEAQ
OXA-51-like_AVO88307.1_A.b. GDAMKASAIPVYQDLARRIGLELMSKEVKRVGYGNADIGTQVDNFWLVGPLKITPQQEAQ
OXA-51-like_AWO15631.1_A.b. GDAMKASAIPVYQDLARRIGLELMSKEVKRVGYGNADIGTQVDNFWLVGPLKITPQQEAQ
OXA-51-like_AWW77770.1_A.b. GDAMKASAIPVYQDLARRIGLELMSKEVKRVGYGNADIGTQVDNFWLVGPLKITPQQEAQ
OXA-51-like_AWW85353.1_A.b. GDAMKASAIPVYQDLARRIGLELMSKEVKRVGYGNADIGTQVDNFWLVGPLKITPQQEAQ
OXA-51-like_AXW91373.1_A.b. GDAMKASAIPVYQDLARRIGLELMSKEVKRVGYGNADIGTQVDNFWLVGPLKITPQQEAQ
OXA-51-like_AXX48901.1_A.b. GDAMKASAIPVYQDLARRIGLELMSKEVKRVGYGNADIGTQVDNFWLVGPLKITPQQEAQ
OXA-51-like_AXX55862.1_A.b. GDAMKASAIPVYQDLARRIGLELMSKEVKRVGYGNADIGTQVDNFWLVGPLKITPQQEAQ
OXA-51-like_QBA07478.1_A.b. GDAMKASAIPVYQDLARRIGLELMSKEVKRVGYGNADIGTQVDNFWLVGPLKITPQQEAQ
OXA-51-like_QBB77223.1_A.b. GDAMKASAIPVYQDLARRIGLELMSKEVKRVGYGNADIGTQVDNFWLVGPLKITPQQEAQ
OXA-51-like_QCH33275.1_A.b. GDAMKASAIPVYQDLARRIGLELMSKEVKRVGYGNADIGTQVDNFWLVGPLKITPQQEAQ
OXA-51-like_QCP15970.1_A.b. GDAMKASAIPVYQDLARRIGLELMSKEVKRVGYGNADIGTQVDNFWLVGPLKITPQQEAQ
OXA-51-like_QCP19079.1_A.b. GDAMKASAIPVYQDLARRIGLELMSKEVKRVGYGNADIGTQVDNFWLVGPLKITPQQEAQ
OXA-51-like_QCP45479.1_A.b. GDAMKASAIPVYQDLARRIGLELMSKEVKRVGYGNADIGTQVDNFWLVGPLKITPQQEAQ
OXA-51-like_QDR93660.1_A.b. GDAMKASAIPVYQDLARRIGLELMSKEVKRVGYGNADIGTQVDNFWLVGPLKITPQQEAQ
OXA-51-like_QEY27958.1_A.b. GDAMKASAIPVYQDLARRIGLELMSKEVKRVGYGNADIGTQVDNFWLVGPLKITPQQEAQ
OXA-51-like_QIX30833.1_A.b. GDAMKASAIPVYQDLARRIGLELMSKEVKRVGYGNADIGTQVDNFWLVGPLKITPQQEAQ
OXA-51-like_QIX34674.1_A.b. GDAMKASAIPVYQDLARRIGLELMSKEVKRVGYGNADIGTQVDNFWLVGPLKITPQQEAQ
OXA-51-like_QIX38631.1_A.b. GDAMKASAIPVYQDLARRIGLELMSKEVKRVGYGNADIGTQVDNFWLVGPLKITPQQEAQ
OXA-51-like_QIX42205.1_A.b. GDAMKASAIPVYQDLARRIGLELMSKEVKRVGYGNADIGTQVDNFWLVGPLKITPQQEAQ
OXA-51-like_QIX46166.1_A.b. GDAMKASAIPVYQDLARRIGLELMSKEVKRVGYGNADIGTQVDNFWLVGPLKITPQQEAQ
OXA-51-like_QJG69125.1_A.b. GDAMKASAIPVYQDLARRIGLELMSKEVKRVGYGNADIGTQVDNFWLVGPLKITPQQEAQ
OXA-51-like_QJG72958.1_A.b. GDAMKASAIPVYQDLARRIGLELMSKEVKRVGYGNADIGTQVDNFWLVGPLKITPQQEAQ
OXA-51-like_QJG88231.1_A.b. GDAMKASAIPVYQDLARRIGLELMSKEVKRVGYGNADIGTQVDNFWLVGPLKITPQQEAQ
OXA-51-like_QJG91989.1_A.b. GDAMKASAIPVYQDLARRIGLELMSKEVKRVGYGNADIGTQVDNFWLVGPLKITPQQEAQ
OXA-51-like_QJG95593.1_A.b. GDAMKASAIPVYQDLARRIGLELMSKEVKRVGYGNADIGTQVDNFWLVGPLKITPQQEAQ
OXA-51-like_QJG99403.1_A.b. GDAMKASAIPVYQDLARRIGLELMSKEVKRVGYGNADIGTQVDNFWLVGPLKITPQQEAQ
OXA-51-like_QJH07093.1_A.b. GDAMKASAIPVYQDLARRIGLELMSKEVKRVGYGNADIGTQVDNFWLVGPLKITPQQEAQ
OXA-51-like_QJH10219.1_A.b. GDAMKASAIPVYQDLARRIGLELMSKEVKRVGYGNADIGTQVDNFWLVGPLKITPQQEAQ
OXA-51-like_QJH14483.1_A.b. GDAMKASAIPVYQDLARRIGLELMSKEVKRVGYGNADIGTQVDNFWLVGPLKITPQQEAQ
OXA-51-like_QJH17776.1_A.b. GDAMKASAIPVYQDLARRIGLELMSKEVKRVGYGNADIGTQVDNFWLVGPLKITPQQEAQ
OXA-51-like_QJP31173.1_A.b. GDAMKASAIPVYQDLARRIGLELMSKEVKRVGYGNADIGTQVDNFWLVGPLKITPQQEAQ
OXA-51-like_QLA73304.1_A.b. GDAMKASAIPVYQDLARRIGLELMSKEVKRVGYGNADIGTQVDNFWLVGPLKITPQQEAQ
OXA-51-like_QNV12531.1_A.b. GDAMKASAIPVYQDLARRIGLELMSKEVKRVGYGNADIGTQVDNFWLVGPLKITPQQEAQ
OXA-51-like_QAS93855.1_A.b. GDAMKASAIPVYQDLARRIGLELMSKEVKRVGYGNADIGTQVDNFWVVGPLKITPQQEAQ
OXA-51-like_QAT01315.1_A.b. GDAMKASAIPVYQDLARRIGLELMSKEVKRVGYGNADIGTQVDNFWVVGPLKITHQQEAQ
OXA-51-like_QAS97806.1_A.b. GDAMKASAIPVYQDLARRIGLELMSKEVKRVGYGNADIGTQVDNFWVVGPLKITPQQEAQ
OXA-51-like_QAT04873.1_A.b. GDAMKASAIPVYQDLARRIGLELMSKEVKRVGYGNADIGTQVDNFWVVGPLKITPQQEAQ
OXA-51-like_QEE57116.1_A.b. GDAMKASAIPVYQDLARRIGLELMSKEVKRVGYGNADIGTQVDNFWVVGPLKITPQQEAQ
OXA-51-like_ADX03652.1_A.b. GDAMKASAIQVYQDLARRIGLELMSKEVKRVGYGNADIGTQVDNFWLVGPLKITPQQEAQ
OXA-51-like_QCO82814.1_A.b. GDAMKASAIQVYQDLARRIGLELMSKEVKRVGYGNADIGTQVDNFWLVGPLKITPQQEAQ
OXA-51-like_AJF81571.1_A.b. GDAMKASAIPVYQDLARRIGLELMSKEVKRVGYGNADIGTQVDNFWLVGPLKITPQQEAQ
OXA-51-like_ATP87403.1_A.b. GDAMKASAIPVYQDLARRIGLELMSKEVKRVGYGNADIGTQVDNFWLVGPLKITPQQEAQ
OXA-51-like_AKA32028.1_A.b. GDAMKASAIPVYQDLARRIGLELMSKEVKRVGYGNADIGTQVDNFWLVGPLKITPQQEAQ
OXA-51-like_ALJ88113.1_A.b. GDAMKASAIPVYQDLARRIGLELMSKEVKRVGYGNADIGTQVDNFWLVGPLKITPQQEAQ
OXA-51-like_ARG31555.1_A.b. GDAMKASAIPVYQDLARRIGLELMSKEVKRVGYGNADIGTQVDNFWLVGPLKITPQQEAQ
OXA-51-like_ASF77094.1_A.b. GDAMKASAIPVYQDLARRIGLELMSKEVKRVGYGNADIGTQVDNFWLVGPLKITPQQEAQ
OXA-51-like_AVN14185.1_A.b. GDAMKASAIPVYQDLARRIGLELMSKEVKRVGYGNADIGTQVDNFWLVGPLKITPQQEAQ
OXA-51-like_AVN25165.1_A.b. GDAMKASAIPVYQDLARRIGLELMSKEVKRVGYGNADIGTQVDNFWLVGPLKITPQQEAQ
OXA-51-like_AXB14467.1_A.b. GDAMKASAIPVYQDLARRIGLELMSKEVKRVGYGNADIGTQVDNFWLVGPLKITPQQEAQ
OXA-51-like_QLG79253.1_A.b. GDAMKASAIPVYQDLARRIGLELMSKEVKRVGYGNADIGTQVDNFWLVGPLKITPQQEAQ
OXA-51-like_QQB68144.1_A.b. GDAMKASAIPVYQDLARRIGLELMSKEVKRVGYGNADIGTQVDNFWLVGPLKITPQQEAQ
OXA-51-like_SQI55469.1_A.b. GDAMKASAIPVYQDLARRIGLELMSKEVKRVGYGNADIGTQVDNFWLVGPLKITPQQEAQ
OXA-51-like_QJH03197.1_A.b. GDAMKASAIPVYQDLARRIGLELMSKEVKRVGYGNADIGTQVDNFWLVGPLKITPQQEAQ
OXA-51-like_QHB90233.1_A.b. GDAMKASAIPVYQDLARRIGLELMSNEVKRIGYGNADIGTQVDNFWLVGPLKITPQQEAQ
OXA-51-like_BBR73296.1_A.b. GDAMKASAIPVYQDLARRIGLELMSKEVKRVGYGNADIGTQVDNFWLVGPLKITPQQEAQ
                       acc
```

OXA-51-like subfamily

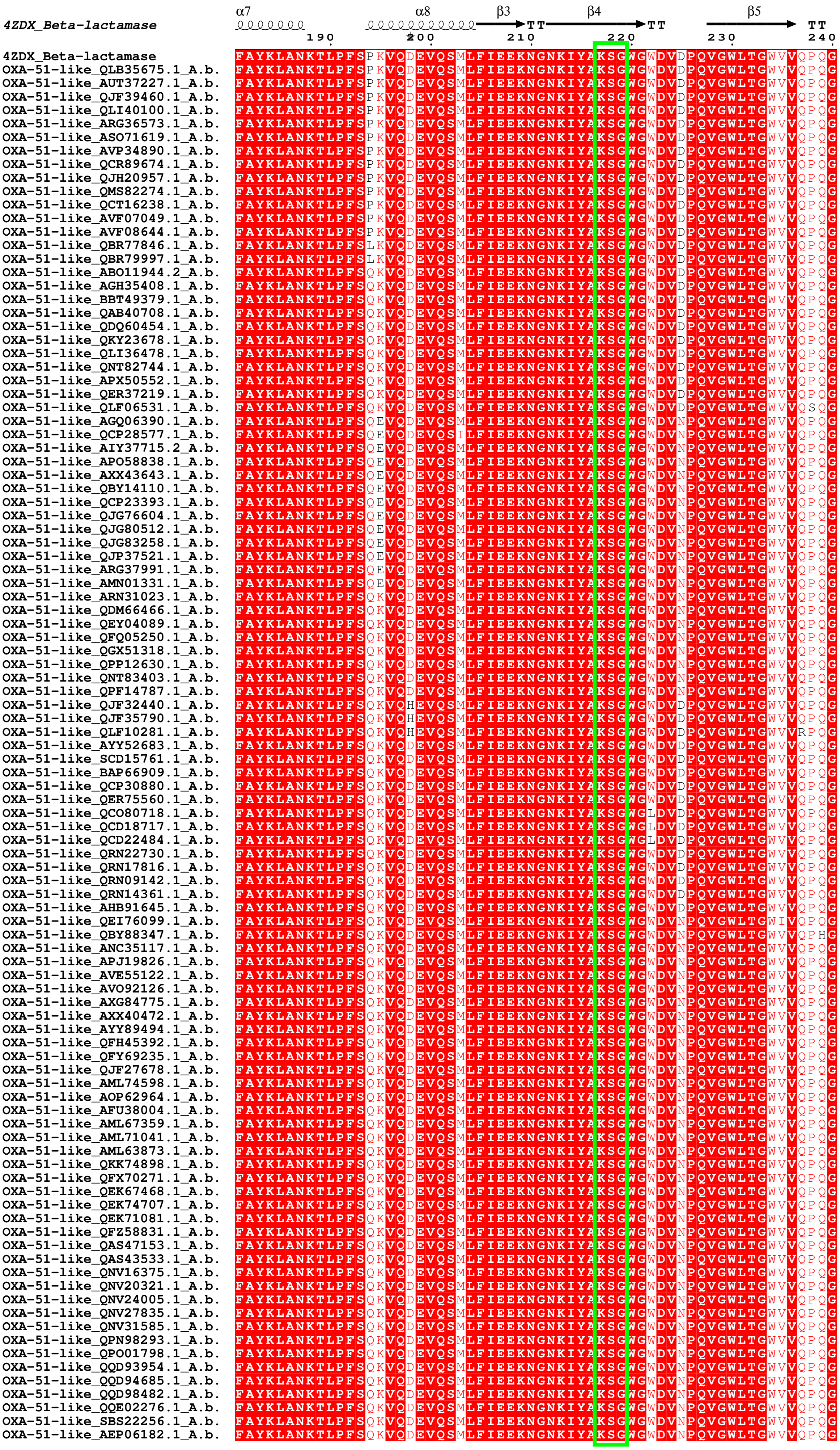

OXA-51-like subfamily

```
OXA-51-like_ARG02295.1_A.b.   FAYKLANKTLPFSQKVQDEVQSMLFIEEKNGNKIYAKSGWGWDVNPQVGWLTGWVVQPQG
OXA-51-like_ARG25110.1_A.b.   FAYKLANKTLPFSQKVQDEVQSMLFIEEKNGNKIYAKSGWGWDVNPQVGWLTGWVVQPQG
OXA-51-like_AYC01674.1_A.b.   FAYKLANKTLPFSQKVQDEVQSMLFIEEKNGNKIYAKSGWGWDVNPQVGWLTGWVVQPQG
OXA-51-like_ATU49292.1_A.b.   FAYKLANKTLPFSQKVQDEVQSMLFIEEKNGNKIYAKSGWGWDVNPQVGWLTGWVVQPQG
OXA-51-like_ATU52990.1_A.b.   FAYKLANKTLPFSQKVQDEVQSMLFIEEKNGNKIYAKSGWGWDVNPQVGWLTGWVVQPQG
OXA-51-like_ATU56628.1_A.b.   FAYKLANKTLPFSQKVQDEVQSMLFIEEKNGNKIYAKSGWGWDVNPQVGWLTGWVVQPQG
OXA-51-like_AZK38077.1_A.b.   FAYKLANKTLPFSQKVQDEVQSMLFIEEKNGNKIYAKSGWGWDVNPQVGWLTGWVVQPQG
OXA-51-like_AZK41733.1_A.b.   FAYKLANKTLPFSQKVQDEVQSMLFIEEKNGNKIYAKSGWGWDVNPQVGWLTGWVVQPQG
OXA-51-like_QAS39909.1_A.b.   FAYKLANKTLPFSQKVQDEVQSMLFIEEKNGNKIYAKSGWGWDVNPQVGWLTGWVVQPQG
OXA-51-like_QAS33473.1_A.b.   FAYKLANKTLPFSQKVQDEVQSMLFIEEKNGNKIYAKSGWGWDVNPQVGWLTGWVVQPQG
OXA-51-like_ACC56872.1_A.b.   FAYKLANKTLPFSQKVQDEVQSMLFIEEKNGNKIYAKSGWGWDVNPQVGWLTGWVVQPQG
OXA-51-like_ADX91666.1_A.b.   FAYKLANKTLPFSQKVQDEVQSMLFIEEKNGNKIYAKSGWGWDVNPQVGWLTGWVVQPQG
OXA-51-like_ADX92178.1_A.b.   FAYKLANKTLPFSQKVQDEVQSMLFIEEKNGNKIYAKSGWGWDVNPQVGWLTGWVVQPQG
OXA-51-like_AGQ10228.1_A.b.   FAYKLANKTLPFSQKVQDEVQSMLFIEEKNGNKIYAKSGWGWDVNPQVGWLTGWVVQPQG
OXA-51-like_ALX99780.1_A.b.   FAYKLANKTLPFSQKVQDEVQSMLFIEEKNGNKIYAKSGWGWDVNPQVGWLTGWVVQPQG
OXA-51-like_AMC15222.1_A.b.   FAYKLANKTLPFSQKVQDEVQSMLFIEEKNGNKIYAKSGWGWDVNPQVGWLTGWVVQPQG
OXA-51-like_ANB88975.1_A.b.   FAYKLANKTLPFSQKVQDEVQSMLFIEEKNGNKIYAKSGWGWDVNPQVGWLTGWVVQPQG
OXA-51-like_AOX69607.1_A.b.   FAYKLANKTLPFSQKVQDEVQSMLFIEEKNGNKIYAKSGWGWDVNPQVGWLTGWVVQPQG
OXA-51-like_AOX73369.1_A.b.   FAYKLANKTLPFSQKVQDEVQSMLFIEEKNGNKIYAKSGWGWDVNPQVGWLTGWVVQPQG
OXA-51-like_AOX77482.1_A.b.   FAYKLANKTLPFSQKVQDEVQSMLFIEEKNGNKIYAKSGWGWDVNPQVGWLTGWVVQPQG
OXA-51-like_AOX81661.1_A.b.   FAYKLANKTLPFSQKVQDEVQSMLFIEEKNGNKIYAKSGWGWDVNPQVGWLTGWVVQPQG
OXA-51-like_AOX85074.1_A.b.   FAYKLANKTLPFSQKVQDEVQSMLFIEEKNGNKIYAKSGWGWDVNPQVGWLTGWVVQPQG
OXA-51-like_AOX88942.1_A.b.   FAYKLANKTLPFSQKVQDEVQSMLFIEEKNGNKIYAKSGWGWDVNPQVGWLTGWVVQPQG
OXA-51-like_AOX92779.1_A.b.   FAYKLANKTLPFSQKVQDEVQSMLFIEEKNGNKIYAKSGWGWDVNPQVGWLTGWVVQPQG
OXA-51-like_AOX96637.1_A.b.   FAYKLANKTLPFSQKVQDEVQSMLFIEEKNGNKIYAKSGWGWDVNPQVGWLTGWVVQPQG
OXA-51-like_APF43961.1_A.b.   FAYKLANKTLPFSQKVQDEVQSMLFIEEKNGNKIYAKSGWGWDVNPQVGWLTGWVVQPQG
OXA-51-like_APJ23527.1_A.b.   FAYKLANKTLPFSQKVQDEVQSMLFIEEKNGNKIYAKSGWGWDVNPQVGWLTGWVVQPQG
OXA-51-like_APM48724.1_A.b.   FAYKLANKTLPFSQKVQDEVQSMLFIEEKNGNKIYAKSGWGWDVNPQVGWLTGWVVQPQG
OXA-51-like_APQ85553.1_A.b.   FAYKLANKTLPFSQKVQDEVQSMLFIEEKNGNKIYAKSGWGWDVNPQVGWLTGWVVQPQG
OXA-51-like_APQ93188.1_A.b.   FAYKLANKTLPFSQKVQDEVQSMLFIEEKNGNKIYAKSGWGWDVNPQVGWLTGWVVQPQG
OXA-51-like_AQU55455.1_A.b.   FAYKLANKTLPFSQKVQDEVQSMLFIEEKNGNKIYAKSGWGWDVNPQVGWLTGWVVQPQG
OXA-51-like_ARF91818.1_A.b.   FAYKLANKTLPFSQKVQDEVQSMLFIEEKNGNKIYAKSGWGWDVNPQVGWLTGWVVQPQG
OXA-51-like_ARF95229.1_A.b.   FAYKLANKTLPFSQKVQDEVQSMLFIEEKNGNKIYAKSGWGWDVNPQVGWLTGWVVQPQG
OXA-51-like_ARG04169.1_A.b.   FAYKLANKTLPFSQKVQDEVQSMLFIEEKNGNKIYAKSGWGWDVNPQVGWLTGWVVQPQG
OXA-51-like_ARG09260.1_A.b.   FAYKLANKTLPFSQKVQDEVQSMLFIEEKNGNKIYAKSGWGWDVNPQVGWLTGWVVQPQG
OXA-51-like_ARG14122.1_A.b.   FAYKLANKTLPFSQKVQDEVQSMLFIEEKNGNKIYAKSGWGWDVNPQVGWLTGWVVQPQG
OXA-51-like_ARG20812.1_A.b.   FAYKLANKTLPFSQKVQDEVQSMLFIEEKNGNKIYAKSGWGWDVNPQVGWLTGWVVQPQG
OXA-51-like_ARG26815.1_A.b.   FAYKLANKTLPFSQKVQDEVQSMLFIEEKNGNKIYAKSGWGWDVNPQVGWLTGWVVQPQG
OXA-51-like_ATR88137.1_A.b.   FAYKLANKTLPFSQKVQDEVQSMLFIEEKNGNKIYAKSGWGWDVNPQVGWLTGWVVQPQG
OXA-51-like_ATU22923.1_A.b.   FAYKLANKTLPFSQKVQDEVQSMLFIEEKNGNKIYAKSGWGWDVNPQVGWLTGWVVQPQG
OXA-51-like_AUG11400.1_A.b.   FAYKLANKTLPFSQKVQDEVQSMLFIEEKNGNKIYAKSGWGWDVNPQVGWLTGWVVQPQG
OXA-51-like_AVE45033.1_A.b.   FAYKLANKTLPFSQKVQDEVQSMLFIEEKNGNKIYAKSGWGWDVNPQVGWLTGWVVQPQG
OXA-51-like_AVE90526.1_A.b.   FAYKLANKTLPFSQKVQDEVQSMLFIEEKNGNKIYAKSGWGWDVNPQVGWLTGWVVQPQG
OXA-51-like_AVO88307.1_A.b.   FAYKLANKTLPFSQKVQDEVQSMLFIEEKNGNKIYAKSGWGWDVNPQVGWLTGWVVQPQG
OXA-51-like_AWO15631.1_A.b.   FAYKLANKTLPFSQKVQDEVQSMLFIEEKNGNKIYAKSGWGWDVNPQVGWLTGWVVQPQG
OXA-51-like_AWW77770.1_A.b.   FAYKLANKTLPFSQKVQDEVQSMLFIEEKNGNKIYAKSGWGWDVNPQVGWLTGWVVQPQG
OXA-51-like_AWW85353.1_A.b.   FAYKLANKTLPFSQKVQDEVQSMLFIEEKNGNKIYAKSGWGWDVNPQVGWLTGWVVQPQG
OXA-51-like_AXW91373.1_A.b.   FAYKLANKTLPFSQKVQDEVQSMLFIEEKNGNKIYAKSGWGWDVNPQVGWLTGWVVQPQG
OXA-51-like_AXX48901.1_A.b.   FAYKLANKTLPFSQKVQDEVQSMLFIEEKNGNKIYAKSGWGWDVNPQVGWLTGWVVQPQG
OXA-51-like_AXX55862.1_A.b.   FAYKLANKTLPFSQKVQDEVQSMLFIEEKNGNKIYAKSGWGWDVNPQVGWLTGWVVQPQG
OXA-51-like_QBA07478.1_A.b.   FAYKLANKTLPFSQKVQDEVQSMLFIEEKNGNKIYAKSGWGWDVNPQVGWLTGWVVQPQG
OXA-51-like_QBB77223.1_A.b.   FAYKLANKTLPFSQKVQDEVQSMLFIEEKNGNKIYAKSGWGWDVNPQVGWLTGWVVQPQG
OXA-51-like_QCH33275.1_A.b.   FAYKLANKTLPFSQKVQDEVQSMLFIEEKNGNKIYAKSGWGWDVNPQVGWLTGWVVQPQG
OXA-51-like_QCP15970.1_A.b.   FAYKLANKTLPFSQKVQDEVQSMLFIEEKNGNKIYAKSGWGWDVNPQVGWLTGWVVQPQG
OXA-51-like_QCP19079.1_A.b.   FAYKLANKTLPFSQKVQDEVQSMLFIEEKNGNKIYAKSGWGWDVNPQVGWLTGWVVQPQG
OXA-51-like_QCP45479.1_A.b.   FAYKLANKTLPFSQKVQDEVQSMLFIEEKNGNKIYAKSGWGWDVNPQVGWLTGWVVQPQG
OXA-51-like_QDR93660.1_A.b.   FAYKLANKTLPFSQKVQDEVQSMLFIEEKNGNKIYAKSGWGWDVNPQVGWLTGWVVQPQG
OXA-51-like_QEY27958.1_A.b.   FAYKLANKTLPFSQKVQDEVQSMLFIEEKNGNKIYAKSGWGWDVNPQVGWLTGWVVQPQG
OXA-51-like_QIX30833.1_A.b.   FAYKLANKTLPFSQKVQDEVQSMLFIEEKNGNKIYAKSGWGWDVNPQVGWLTGWVVQPQG
OXA-51-like_QIX34674.1_A.b.   FAYKLANKTLPFSQKVQDEVQSMLFIEEKNGNKIYAKSGWGWDVNPQVGWLTGWVVQPQG
OXA-51-like_QIX38631.1_A.b.   FAYKLANKTLPFSQKVQDEVQSMLFIEEKNGNKIYAKSGWGWDVNPQVGWLTGWVVQPQG
OXA-51-like_QIX42205.1_A.b.   FAYKLANKTLPFSQKVQDEVQSMLFIEEKNGNKIYAKSGWGWDVNPQVGWLTGWVVQPQG
OXA-51-like_QIX46166.1_A.b.   FAYKLANKTLPFSQKVQDEVQSMLFIEEKNGNKIYAKSGWGWDVNPQVGWLTGWVVQPQG
OXA-51-like_QJG69125.1_A.b.   FAYKLANKTLPFSQKVQDEVQSMLFIEEKNGNKIYAKSGWGWDVNPQVGWLTGWVVQPQG
OXA-51-like_QJG72958.1_A.b.   FAYKLANKTLPFSQKVQDEVQSMLFIEEKNGNKIYAKSGWGWDVNPQVGWLTGWVVQPQG
OXA-51-like_QJG88231.1_A.b.   FAYKLANKTLPFSQKVQDEVQSMLFIEEKNGNKIYAKSGWGWDVNPQVGWLTGWVVQPQG
OXA-51-like_QJG91989.1_A.b.   FAYKLANKTLPFSQKVQDEVQSMLFIEEKNGNKIYAKSGWGWDVNPQVGWLTGWVVQPQG
OXA-51-like_QJG95593.1_A.b.   FAYKLANKTLPFSQKVQDEVQSMLFIEEKNGNKIYAKSGWGWDVNPQVGWLTGWVVQPQG
OXA-51-like_QJG99403.1_A.b.   FAYKLANKTLPFSQKVQDEVQSMLFIEEKNGNKIYAKSGWGWDVNPQVGWLTGWVVQPQG
OXA-51-like_QJH07093.1_A.b.   FAYKLANKTLPFSQKVQDEVQSMLFIEEKNGNKIYAKSGWGWDVNPQVGWLTGWVVQPQG
OXA-51-like_QJH10219.1_A.b.   FAYKLANKTLPFSQKVQDEVQSMLFIEEKNGNKIYAKSGWGWDVNPQVGWLTGWVVQPQG
OXA-51-like_QJH14483.1_A.b.   FAYKLANKTLPFSQKVQDEVQSMLFIEEKNGNKIYAKSGWGWDVNPQVGWLTGWVVQPQG
OXA-51-like_QJH17776.1_A.b.   FAYKLANKTLPFSQKVQDEVQSMLFIEEKNGNKIYAKSGWGWDVNPQVGWLTGWVVQPQG
OXA-51-like_QJP31173.1_A.b.   FAYKLANKTLPFSQKVQDEVQSMLFIEEKNGNKIYAKSGWGWDVNPQVGWLTGWVVQPQG
OXA-51-like_QLA73304.1_A.b.   FAYKLANKTLPFSQKVQDEVQSMLFIEEKNGNKIYAKSGWGWDVNPQVGWLTGWVVQPQG
OXA-51-like_QNV12531.1_A.b.   FAYKLANKTLPFSQKVQDEVQSMLFIEEKNGNKIYAKSGWGWDVNPQVGWLTGWVVQPQG
OXA-51-like_QAS93855.1_A.b.   FAYKLANKTLPFSQKVQDEVQSMLFIEEKNGNKIYAKSGWGWDVNPQVGWLTGWVVQPQG
OXA-51-like_QAT01315.1_A.b.   FAYKLANKTLPFSQKVQDEVQSMLFIEEKNGNKIYAKSGWGWDVNPQVGWLTGWVVQPQG
OXA-51-like_QAS97806.1_A.b.   FAYKLANKTLPFSQKVQDEVQSMLFIEEKNGNKIYAKSGWGWDVNPQVGWLTGWVVQPQG
OXA-51-like_QAT04873.1_A.b.   FAYKLANKTLPFSQKVQDEVQSMLFIEEKNGNKIYAKSGWGWDVNPQVGWLTGWVVQPQG
OXA-51-like_QEE57116.1_A.b.   FAYKLANKTLPFSQKVQDEVQSMLFIEEKNGNKIYAKSGWGWDVNPQVGWLTGWVVQPQG
OXA-51-like_ADX03652.1_A.b.   FAYKLANKTLPFSQKVQDEVQSMLFIEEKNGNKIYAKSGWGWDVNPQVGWLTGWVVQPQG
OXA-51-like_QCO82814.1_A.b.   FAYKLANKTLPFSQKVQDEVQSMLFIEEKNGNKIYAKSGWGLDVNPQVGWLTGWVVQPQG
OXA-51-like_AJF81571.1_A.b.   FAYKLANKTLPFSQKVQDEVQSMLFIEEKNGNKIYAKSGWGWDVNPQVGWLTGWVVQPQG
OXA-51-like_ATP87403.1_A.b.   FAYKLANKTLPFSQKVQDEVQSMLFIEEKNGNKIYAKSGWGWDVNPQVGWLTGSVVQPQG
OXA-51-like_AKA32028.1_A.b.   FAYKLANKTLPFSQKVQDEVQSMLFIEEKNGNKIYAKSGWGWDVNPQVGWLTGWVVQPQG
OXA-51-like_ALJ88113.1_A.b.   FAYKLANKTLPFSQKVQDEVQSMLFIEEKNGNKIYAKSGWGWDVNPQVGWLTGWVVQPQG
OXA-51-like_ARG31555.1_A.b.   FAYKLANKTLPFSQKVQDEVQSMLFIEEKNGNKIYAKSGWGWDVNPQVGWLTGWVVQPQG
OXA-51-like_ASF77094.1_A.b.   FAYKLANKTLPFSQKVQDEVQSMLFIEEKNGNKIYAKSGWGWDVNPQVGWLTGWVVQPQG
OXA-51-like_AVN14185.1_A.b.   FAYKLANKTLPFSQKVQDEVQSMLFIEEKNGNKIYAKSGWGWDVNPQVGWLTGWVVQPQG
OXA-51-like_AVN25165.1_A.b.   FAYKLANKTLPFSQKVQDEVQSMLFIEEKNGNKIYAKSGWGWDVNPQVGWLTGWVVQPQG
OXA-51-like_AXB14467.1_A.b.   FAYKLANKTLPFSQKVQDEVQSMLFIEEKNGNKIYAKSGWGWDVNPQVGWLTGWVVQPQG
OXA-51-like_QLG79253.1_A.b.   FAYKLANKTLPFSQKVQDEVQSMLFIEEKNGNKIYAKSGWGWDVNPQVGWLTGWVVQPQG
OXA-51-like_QQB68144.1_A.b.   FAYKLANKTLPFSQKVQDEVQSMLFIEEKNGNKIYAKSGWGWDVNPQVGWLTGWVVQPQG
OXA-51-like_SQI55469.1_A.b.   FAYKLANKTLPFSQKVQDEVQSMLFIEEKNGNKIYAKSGWGWDVNPQVGWLTGWVVQPQG
OXA-51-like_QJH03197.1_A.b.   FAYKLANKTLPFSQKVQDEVQSMLFIEEKNGNKIYAKSGWGWDVNPQVGWLTGWVVKPQG
OXA-51-like_QHB90233.1_A.b.   FAYKLANKTLPFSQKVQDEVQSMLFIEEKNGNKIYAKSGWGWDVNPQVGWLTGWVVQPQG
OXA-51-like_BBR73296.1_A.b.   FAYKLANKTLPFSQKVQDEVQSMLFIEEKNGNKIYAKSGWGWDVNPQVGWLTGWVVQPQG
                        acc
```

OXA-51-like subfamily

```
                                      β6                    α9
4ZDX_Beta-lactamase               ----------->   TT   ℓℓℓℓℓℓℓℓℓℓℓℓℓ
                                             250       260       270
4ZDX_Beta-lactamase               NIVAFSLNLEMKKGIPSSVRKEITYKSLEQLGIL
OXA-51-like_QLB35675.1_A.b.       NIVAFSLNLEMKKGIPSSVRKEMTYKSLEQLGIL
OXA-51-like_AUT37227.1_A.b.       NIVAFSLNLEMKKGIPSSVRKEITYKSLEQLGIL
OXA-51-like_QJF39460.1_A.b.       NIVAFSLNLEMKKGIPSSVRKEITYKSLEQLGIL
OXA-51-like_QLI40100.1_A.b.       NIVAFSLNLEMKKGIPSSVRKEITYKSLEQLGIL
OXA-51-like_ARG36573.1_A.b.       NIVAFSLNLEMKKGIPSSVRKEITYKSLEQLGIL
OXA-51-like_ASO71619.1_A.b.       NIVAFSLNLEMKKGIPSSVRKEITYKSLEQLGIL
OXA-51-like_AVP34890.1_A.b.       NIVAFSLNLEMKKGIPSSVRKEITYKSLEQLGIL
OXA-51-like_QCR89674.1_A.b.       NIVAFSLNLEMKKGIPSSVRKEITYKSLEQLGIL
OXA-51-like_QJH20957.1_A.b.       NIVAFSLNLEMKKGIPSSVRKEITYKSLEQLGIL
OXA-51-like_QMS82274.1_A.b.       NIVAFSLNLEMKKGIPSSVRKEITYKSLEQLGIL
OXA-51-like_QCT16238.1_A.b.       NIVAFSLNLEMKKGIPSSVRKEITYKSLEQLGIL
OXA-51-like_AVF07049.1_A.b.       NIVAFSLNLEMKKGIPSSVRKEITYKSLEQLGIL
OXA-51-like_AVF08644.1_A.b.       NIVAFSLNLEMKKGIPSSVRKEITYKSLEQLGIL
OXA-51-like_QBR77846.1_A.b.       NIVAFSLNLEMKKGIPSSVRKEITYKSLEQLGIL
OXA-51-like_QBR79997.1_A.b.       NIVAFSLNLEMKKGIPSSVRKEITYKSLEQLGIL
OXA-51-like_ABO11944.2_A.b.       NIVAFSLNLEMKKGIPSSVRKEITYKSLEQLGIL
OXA-51-like_AGH35408.1_A.b.       NIVAFSLNLEMKKGIPSSVRKEITYKSLEQLGIL
OXA-51-like_BBT49379.1_A.b.       NIVAFSLNLEMKKGIPSSVRKEITYKSLEQLGIL
OXA-51-like_QAB40708.1_A.b.       NIVAFSLNLEMKKGIPSSVRKEITYKSLEQLGIL
OXA-51-like_QDQ60454.1_A.b.       NIVAFSLNLEMKKGIPSSVRKEITYKSLEQLGIL
OXA-51-like_QKY23678.1_A.b.       NIVAFSLNLEMKKGIPSSVRKEITYKSLEQLGIL
OXA-51-like_QLI36478.1_A.b.       NIVAFSLNLEMKKGIPSSVRKEITYKSLEQLGIL
OXA-51-like_QNT82744.1_A.b.       NIVAFSLNLEMKKGIPSSVRKEITYKSLEQLGIL
OXA-51-like_APX50552.1_A.b.       NIVAFSLNLEMKKGIPSSVRKEITYKSLEQLGIL
OXA-51-like_QER37219.1_A.b.       NIVAFSLNLEMKKGIPSSVRKEITYKSLEQLGIL
OXA-51-like_QLF06531.1_A.b.       NIVAFSLNLEMKKGIPSSVRKEITYKSLEQLGIL
OXA-51-like_AGQ06390.1_A.b.       NIVAFSLNLEMKKGIPSSVRKEITYKSLEQLGIL
OXA-51-like_QCP28577.1_A.b.       NIVAFSLNLEMKKGIPSSVRKEITYKSLEQLGIL
OXA-51-like_AIY37715.2_A.b.       NIVAFSLNLEMKKGIPSSVRKEITYKSLEQLGIL
OXA-51-like_APO58838.1_A.b.       NIVAFSLNLEMKKGIPSSVRKEITYKSLEQLGIL
OXA-51-like_AXX43643.1_A.b.       NIVAFSLNLEMKKGIPSSVRKEITYKSLEQLGIL
OXA-51-like_QBY14110.1_A.b.       NIVAFSLNLEMKKGIPSSVRKEITYKSLEQLGIL
OXA-51-like_QCP23393.1_A.b.       NIVAFSLNLEMKKGIPSSVRKEITYKSLEQLGIL
OXA-51-like_QJG76604.1_A.b.       NIVAFSLNLEMKKGIPSSVRKEITYKSLEQLGIL
OXA-51-like_QJG80512.1_A.b.       NIVAFSLNLEMKKGIPSSVRKEITYKSLEQLGIL
OXA-51-like_QJG83258.1_A.b.       NIVAFSLNLEMKKGIPSSVRKEITYKSLEQLGIL
OXA-51-like_QJP37521.1_A.b.       NIVAFSLNLEMKKGIPSSVRKEITYKSLEQLGIL
OXA-51-like_ARG37991.1_A.b.       NIVAFSLNLEMKKGIPSSVRKEITYKSLEQLGIL
OXA-51-like_AMN01331.1_A.b.       NIVAFSLNLEMKKGIPSSVRKEITYKSLEQLGIL
OXA-51-like_ARN31023.1_A.b.       NIVAFSLNLEMKKGIPSSVRKEITYKSLEQLGIL
OXA-51-like_QDM66466.1_A.b.       NIVAFSLNLEMKKGIPSSVRKEITYKSLEQLGIL
OXA-51-like_QEY04089.1_A.b.       NIVAFSLNLEMKKGIPSSVRKEITYKSLEQLGIL
OXA-51-like_QFQ05250.1_A.b.       NIVAFSLNLEMKKGIPSSVRKEITYKSLEQLGIL
OXA-51-like_QGX51318.1_A.b.       NIVAFSLNLEMKKGIPSSVRKEITYKSLEQLGIL
OXA-51-like_QPP12630.1_A.b.       NIVAFSLNLEMKKGIPSSVRKEITYKSLEQLGIL
OXA-51-like_QNT83403.1_A.b.       NIVAFSLNLEMKKGIPSSVRKEITYKSLEQLGIL
OXA-51-like_QPF14787.1_A.b.       NIVAFSLNLEMKKGTPSSVRKEITYKSLEQLGIL
OXA-51-like_QJF32440.1_A.b.       NIVAFSLNLEMKKGIPSSVRKEITYKSLEQLGIL
OXA-51-like_QJF35790.1_A.b.       NIVAFSLNLEMKKGIPSSVRKEITYKSLEQLGIL
OXA-51-like_QLF10281.1_A.b.       NIVAFSLNLEMKKGIPSSVRKEITYKSLEQLGIL
OXA-51-like_AYY52683.1_A.b.       NIVAFSLNLEMKKGIPSSVRKEITYKSLEQLGIL
OXA-51-like_SCD15761.1_A.b.       NIVAFSLNLEMKKGIPSSVRKEITYKSLEQLGIL
OXA-51-like_BAP66909.1_A.b.       NIVAFSLNLEMKKGIPSSVRKEITYKSLEQLGIL
OXA-51-like_QCP30880.1_A.b.       NIVAFSLNLEMKKGIPSSVRKEITYKSLEQLGIL
OXA-51-like_QER75560.1_A.b.       NIVAFSLNLEMKKGIPSSVRKEITYKSLEQLGIL
OXA-51-like_QCO80718.1_A.b.       NIVAFSLNLEMKKGIPSSVRKEITYKSLEQLGIL
OXA-51-like_QCD18717.1_A.b.       NIVAFSLNLEMKKGIPSSVRKEITYKSLEQLGIL
OXA-51-like_QCD22484.1_A.b.       NIVAFSLNLEMKKGIPSSVRKEITYKSLEQLGIL
OXA-51-like_QRN22730.1_A.b.       NIVAFSLNLEMKKGIPSSVRKEITYKSLEQLGIL
OXA-51-like_QRN17816.1_A.b.       NIVAFSLNLEMKKGIPSSVRKEITYKSLEQLGIL
OXA-51-like_QRN09142.1_A.b.       NIVAFSLNLEMKKGIPSSVRKEITYKSLEQLGIL
OXA-51-like_QRN14361.1_A.b.       NIVAFSLNLEMKKGIPSSVRKEITYKSLEQLGIL
OXA-51-like_AHB91645.1_A.b.       NIVAFSLNLEMKKGIPSSVRKEITYKSLEQLGIL
OXA-51-like_QEI76099.1_A.b.       NIVAFSLNLEMKKGIPSSVRKEITYKSLEQLGIL
OXA-51-like_QBY88347.1_A.b.       NIVAFSLNLEMKKGIPSSVRKEITYKSLEQLGIL
OXA-51-like_ANC35117.1_A.b.       NIVAFSLNLEMKKGIPSSVRKEITYKSLEQLGIL
OXA-51-like_APJ19826.1_A.b.       NIVAFSLNLEMKKGIPSSVRKEITYKSLEQLGIL
OXA-51-like_AVE55122.1_A.b.       NIVAFSLNLEMKKGIPSSVRKEITYKSLEQLGIL
OXA-51-like_AVO92126.1_A.b.       NIVAFSLNLEMKKGIPSSVRKEITYKSLEQLGIL
OXA-51-like_AXG84775.1_A.b.       NIVAFSLNLEMKKGIPSSVRKEITYKSLEQLGIL
OXA-51-like_AXX40472.1_A.b.       NIVAFSLNLEMKKGIPSSVRKEITYKSLEQLGIL
OXA-51-like_AYY89494.1_A.b.       NIVAFSLNLEMKKGIPSSVRKEITYKSLEQLGIL
OXA-51-like_QFH45392.1_A.b.       NIVAFSLNLEMKKGIPSSVRKEITYKSLEQLGIL
OXA-51-like_QFY69235.1_A.b.       NIVAFSLNLEMKKGIPSSVRKEITYKSLEQLGIL
OXA-51-like_QJF27678.1_A.b.       NIVAFSLNLEMKKGIPSSVRKEITYKSLEQLGIL
OXA-51-like_AML74598.1_A.b.       NIVAFSLNLEMKKGIPSSVRKEITYKSLEQLGIL
OXA-51-like_AOP62964.1_A.b.       NIVAFSLNLEMKKGIPSSVRKEITYKSLEQLGIL
OXA-51-like_AFU38004.1_A.b.       NIVAFSLNLEMKKGIPSSVRKEITYKSLEQLGIL
OXA-51-like_AML67359.1_A.b.       NIVAFSLNLEMKKGIPSSVRKEITYKSLEQLGIL
OXA-51-like_AML71041.1_A.b.       NIVAFSLNLEMKKGIPSSVRKEITYKSLEQLGIL
OXA-51-like_AML63873.1_A.b.       NIVAFSLNLEMKKGIPSSVRKEITYKSLEQLGIL
OXA-51-like_QKK74898.1_A.b.       NIVAFSLNLEMKKGIPSSVRKEITYKSLEQLGIL
OXA-51-like_QFX70271.1_A.b.       NIVAFSLNLEMKKGIPSSVRKEITYKSLEQLGIL
OXA-51-like_QEK67468.1_A.b.       NIVAFSLNLEMKKGIPSSVRKEITYKSLEQLGIL
OXA-51-like_QEK74707.1_A.b.       NIVAFSLNLEMKKGIPSSVRKEITYKSLEQLGIL
OXA-51-like_QEK71081.1_A.b.       NIVAFSLNLEMKKGIPSSVRKEITYKSLEQLGIL
OXA-51-like_QFZ58831.1_A.b.       NIVAFSLNLEMKKGIPSSVRKEITYKSLEQLGIL
OXA-51-like_QAS47153.1_A.b.       NIVAFSLNLEMKKGIPSSVRKEITYKSLEQLGIL
OXA-51-like_QAS43533.1_A.b.       NIVAFSLNLEMKKGIPSSVRKEITYKSLEQLGIL
OXA-51-like_QNV16375.1_A.b.       NIVAFSLNLEMKKGIPSSVRKEITYKSLEQLGIL
OXA-51-like_QNV20321.1_A.b.       NIVAFSLNLEMKKGIPSSVRKEITYKSLEQLGIL
OXA-51-like_QNV24005.1_A.b.       NIVAFSLNLEMKKGIPSSVRKEITYKSLEQLGIL
OXA-51-like_QNV27835.1_A.b.       NIVAFSLNLEMKKGIPSSVRKEITYKSLEQLGIL
OXA-51-like_QNV31585.1_A.b.       NIVAFSLNLEMKKGIPSSVRKEITYKSLEQLGIL
OXA-51-like_QPN98293.1_A.b.       NIVAFSLNLEMKKGIPSSVRKEITYKSLEQLGIL
OXA-51-like_QPO01798.1_A.b.       NIVAFSLNLEMKKGIPSSVRKEITYKSLEQLGIL
OXA-51-like_QQD93954.1_A.b.       NIVAFSLNLEMKKGIPSSVRKEITYKSLEQLGIL
OXA-51-like_QQD94685.1_A.b.       NIVAFSLNLEMKKGIPSSVRKEITYKSLEQLGIL
OXA-51-like_QQD98482.1_A.b.       NIVAFSLNLEMKKGIPSSVRKEITYKSLEQLGIL
OXA-51-like_QQE02276.1_A.b.       NIVAFSLNLEMKKGIPSSVRKEITYKSLEQLGIL
OXA-51-like_SBS22256.1_A.b.       NIVAFSLNLEMKKGIPSSVRKEITYKSLEQLGIL
OXA-51-like_AEP06182.1_A.b.       NIVAFSLNLEMKKGIPSSVRKEITYKSLEQLGIL
```

OXA-51-like subfamily

```
OXA-51-like_ARG02295.1_A.b.  NIVAFSLNLEMKKGIPSSVRKEITYKSLEQLGIL
OXA-51-like_ARG25110.1_A.b.  NIVAFSLNLEMKKGIPSSVRKEITYKSLEQLGIL
OXA-51-like_AYC01674.1_A.b.  NIVAFSLNLEMKKGIPSSVRKEITYKSLEQLGIL
OXA-51-like_ATU49292.1_A.b.  NIVAFSLNLEMKKGIPSSVRKEITYKSLEQLGIL
OXA-51-like_ATU52990.1_A.b.  NIVAFSLNLEMKKGIPSSVRKEITYKSLEQLGIL
OXA-51-like_ATU56628.1_A.b.  NIVAFSLNLEMKKGIPSSVRKEITYKSLEQLGIL
OXA-51-like_AZK38077.1_A.b.  NIVAFSLNLEMKKGIPSSVRKEITYKSLEQLGIL
OXA-51-like_AZK41733.1_A.b.  NIVAFSLNLEMKKGIPSSVRKEITYKSLEQLGIL
OXA-51-like_QAS39909.1_A.b.  NIVAFSLNLEMKKGIPSSVRKEITYKSLEQLGIL
OXA-51-like_QAS33473.1_A.b.  NIVAFSLNLEMKKGIPSSVRKEITYKSLEQLGIL
OXA-51-like_ACC56872.1_A.b.  NIVAFSLNLEMKKGIPSSVRKEITYKSLEQLGIL
OXA-51-like_ADX91666.1_A.b.  NIVAFSLNLEMKKGIPSSVRKEITYKSLEQLGIL
OXA-51-like_ADX92178.1_A.b.  NIVAFSLNLEMKKGIPSSVRKEITYKSLEQLGIL
OXA-51-like_AGQ10228.1_A.b.  NIVAFSLNLEMKKGIPSSVRKEITYKSLEQLGIL
OXA-51-like_ALX99780.1_A.b.  NIVAFSLNLEMKKGIPSSVRKEITYKSLEQLGIL
OXA-51-like_AMC15222.1_A.b.  NIVAFSLNLEMKKGIPSSVRKEITYKSLEQLGIL
OXA-51-like_ANB88975.1_A.b.  NIVAFSLNLEMKKGIPSSVRKEITYKSLEQLGIL
OXA-51-like_AOX69607.1_A.b.  NIVAFSLNLEMKKGIPSSVRKEITYKSLEQLGIL
OXA-51-like_AOX73369.1_A.b.  NIVAFSLNLEMKKGIPSSVRKEITYKSLEQLGIL
OXA-51-like_AOX77482.1_A.b.  NIVAFSLNLEMKKGIPSSVRKEITYKSLEQLGIL
OXA-51-like_AOX81661.1_A.b.  NIVAFSLNLEMKKGIPSSVRKEITYKSLEQLGIL
OXA-51-like_AOX85074.1_A.b.  NIVAFSLNLEMKKGIPSSVRKEITYKSLEQLGIL
OXA-51-like_AOX88942.1_A.b.  NIVAFSLNLEMKKGIPSSVRKEITYKSLEQLGIL
OXA-51-like_AOX92779.1_A.b.  NIVAFSLNLEMKKGIPSSVRKEITYKSLEQLGIL
OXA-51-like_AOX96637.1_A.b.  NIVAFSLNLEMKKGIPSSVRKEITYKSLEQLGIL
OXA-51-like_APF43961.1_A.b.  NIVAFSLNLEMKKGIPSSVRKEITYKSLEQLGIL
OXA-51-like_APJ23527.1_A.b.  NIVAFSLNLEMKKGIPSSVRKEITYKSLEQLGIL
OXA-51-like_APM48724.1_A.b.  NIVAFSLNLEMKKGIPSSVRKEITYKSLEQLGIL
OXA-51-like_APQ85553.1_A.b.  NIVAFSLNLEMKKGIPSSVRKEITYKSLEQLGIL
OXA-51-like_APQ93188.1_A.b.  NIVAFSLNLEMKKGIPSSVRKEITYKSLEQLGIL
OXA-51-like_AQU55455.1_A.b.  NIVAFSLNLEMKKGIPSSVRKEITYKSLEQLGIL
OXA-51-like_ARF91818.1_A.b.  NIVAFSLNLEMKKGIPSSVRKEITYKSLEQLGIL
OXA-51-like_ARF95229.1_A.b.  NIVAFSLNLEMKKGIPSSVRKEITYKSLEQLGIL
OXA-51-like_ARG04169.1_A.b.  NIVAFSLNLEMKKGIPSSVRKEITYKSLEQLGIL
OXA-51-like_ARG09260.1_A.b.  NIVAFSLNLEMKKGIPSSVRKEITYKSLEQLGIL
OXA-51-like_ARG14122.1_A.b.  NIVAFSLNLEMKKGIPSSVRKEITYKSLEQLGIL
OXA-51-like_ARG20812.1_A.b.  NIVAFSLNLEMKKGIPSSVRKEITYKSLEQLGIL
OXA-51-like_ARG26815.1_A.b.  NIVAFSLNLEMKKGIPSSVRKEITYKSLEQLGIL
OXA-51-like_ATR88137.1_A.b.  NIVAFSLNLEMKKGIPSSVRKEITYKSLEQLGIL
OXA-51-like_ATU22923.1_A.b.  NIVAFSLNLEMKKGIPSSVRKEITYKSLEQLGIL
OXA-51-like_AUG11400.1_A.b.  NIVAFSLNLEMKKGIPSSVRKEITYKSLEQLGIL
OXA-51-like_AVE45033.1_A.b.  NIVAFSLNLEMKKGIPSSVRKEITYKSLEQLGIL
OXA-51-like_AVE90526.1_A.b.  NIVAFSLNLEMKKGIPSSVRKEITYKSLEQLGIL
OXA-51-like_AVO88307.1_A.b.  NIVAFSLNLEMKKGIPSSVRKEITYKSLEQLGIL
OXA-51-like_AWO15631.1_A.b.  NIVAFSLNLEMKKGIPSSVRKEITYKSLEQLGIL
OXA-51-like_AWW77770.1_A.b.  NIVAFSLNLEMKKGIPSSVRKEITYKSLEQLGIL
OXA-51-like_AWW85353.1_A.b.  NIVAFSLNLEMKKGIPSSVRKEITYKSLEQLGIL
OXA-51-like_AXW91373.1_A.b.  NIVAFSLNLEMKKGIPSSVRKEITYKSLEQLGIL
OXA-51-like_AXX48901.1_A.b.  NIVAFSLNLEMKKGIPSSVRKEITYKSLEQLGIL
OXA-51-like_AXX55862.1_A.b.  NIVAFSLNLEMKKGIPSSVRKEITYKSLEQLGIL
OXA-51-like_QBA07478.1_A.b.  NIVAFSLNLEMKKGIPSSVRKEITYKSLEQLGIL
OXA-51-like_QBB77223.1_A.b.  NIVAFSLNLEMKKGIPSSVRKEITYKSLEQLGIL
OXA-51-like_QCH33275.1_A.b.  NIVAFSLNLEMKKGIPSSVRKEITYKSLEQLGIL
OXA-51-like_QCP15970.1_A.b.  NIVAFSLNLEMKKGIPSSVRKEITYKSLEQLGIL
OXA-51-like_QCP19079.1_A.b.  NIVAFSLNLEMKKGIPSSVRKEITYKSLEQLGIL
OXA-51-like_QCP45479.1_A.b.  NIVAFSLNLEMKKGIPSSVRKEITYKSLEQLGIL
OXA-51-like_QDR93660.1_A.b.  NIVAFSLNLEMKKGIPSSVRKEITYKSLEQLGIL
OXA-51-like_QEY27958.1_A.b.  NIVAFSLNLEMKKGIPSSVRKEITYKSLEQLGIL
OXA-51-like_QIX30833.1_A.b.  NIVAFSLNLEMKKGIPSSVRKEITYKSLEQLGIL
OXA-51-like_QIX34674.1_A.b.  NIVAFSLNLEMKKGIPSSVRKEITYKSLEQLGIL
OXA-51-like_QIX38631.1_A.b.  NIVAFSLNLEMKKGIPSSVRKEITYKSLEQLGIL
OXA-51-like_QIX42205.1_A.b.  NIVAFSLNLEMKKGIPSSVRKEITYKSLEQLGIL
OXA-51-like_QIX46166.1_A.b.  NIVAFSLNLEMKKGIPSSVRKEITYKSLEQLGIL
OXA-51-like_QJG69125.1_A.b.  NIVAFSLNLEMKKGIPSSVRKEITYKSLEQLGIL
OXA-51-like_QJG72958.1_A.b.  NIVAFSLNLEMKKGIPSSVRKEITYKSLEQLGIL
OXA-51-like_QJG88231.1_A.b.  NIVAFSLNLEMKKGIPSSVRKEITYKSLEQLGIL
OXA-51-like_QJG91989.1_A.b.  NIVAFSLNLEMKKGIPSSVRKEITYKSLEQLGIL
OXA-51-like_QJG95593.1_A.b.  NIVAFSLNLEMKKGIPSSVRKEITYKSLEQLGIL
OXA-51-like_QJG99403.1_A.b.  NIVAFSLNLEMKKGIPSSVRKEITYKSLEQLGIL
OXA-51-like_QJH07093.1_A.b.  NIVAFSLNLEMKKGIPSSVRKEITYKSLEQLGIL
OXA-51-like_QJH10219.1_A.b.  NIVAFSLNLEMKKGIPSSVRKEITYKSLEQLGIL
OXA-51-like_QJH14483.1_A.b.  NIVAFSLNLEMKKGIPSSVRKEITYKSLEQLGIL
OXA-51-like_QJH17776.1_A.b.  NIVAFSLNLEMKKGIPSSVRKEITYKSLEQLGIL
OXA-51-like_QJP31173.1_A.b.  NIVAFSLNLEMKKGIPSSVRKEITYKSLEQLGIL
OXA-51-like_QLA73304.1_A.b.  NIVAFSLNLEMKKGIPSSVRKEITYKSLEQLGIL
OXA-51-like_QNV12531.1_A.b.  NIVAFSLNLEMKKGIPSSVRKEITYKSLEQLGIL
OXA-51-like_QAS93855.1_A.b.  NIVAFSLNLEMKKGIPSSVRKEITYKSLEQLGIL
OXA-51-like_QAT01315.1_A.b.  NIVAFSLNLEMKKGIPSSVRKEITYKSLEQLGIL
OXA-51-like_QAS97806.1_A.b.  NIVAFSLNLEMKKGIPSSVRKEITYKSLEQLGIL
OXA-51-like_QAT04873.1_A.b.  NIVAFSLNLEMKKGIPSSVRKEITYKSLEQLGIL
OXA-51-like_QEE57116.1_A.b.  NIVAFSLNLEMKKGIPSSVRKEITYKSLEQLGIL
OXA-51-like_ADX03652.1_A.b.  NIVAFSLNLEMKKGIPSSVRKEITYKSLEQLGIL
OXA-51-like_QCO82814.1_A.b.  NIVAFSLNLEMKKGIPSSVRKEITYKSLEQLGIL
OXA-51-like_AJF81571.1_A.b.  NIVAFSLNLEMKKGIPSSVRKEITYKSLEQLGIL
OXA-51-like_ATP87403.1_A.b.  NIVAFSLNLEMKKGIPSSVRKEITYKSLEQLGIL
OXA-51-like_AKA32028.1_A.b.  NIVAFSLNLEMKKGIPSSVRKEITYKSLEQLGIL
OXA-51-like_ALJ88113.1_A.b.  NIVAFSLNLEMKKGIPSSVRKEITYKSLEQLGIL
OXA-51-like_ARG31555.1_A.b.  NIVAFSLNLEMKKGIPSSVRKEITYKSLEQLGIL
OXA-51-like_ASF77094.1_A.b.  NIVAFSLNLEMKKGIPSSVRKEITYKSLEQLGIL
OXA-51-like_AVN14185.1_A.b.  NIVAFSLNLEMKKGIPSSVRKEITYKSLEQLGIL
OXA-51-like_AVN25165.1_A.b.  NIVAFSLNLEMKKGIPSSVRKEITYKSLEQLGIL
OXA-51-like_AXB14467.1_A.b.  NIVAFSLNLEMKKGIPSSVRKEITYKSLEQLGIL
OXA-51-like_QLG79253.1_A.b.  NIVAFSLNLEMKKGIPSSVRKEITYKSLEQLGIL
OXA-51-like_QQB68144.1_A.b.  NIVAFSLNLEMKKGIPSSVRKEITYKSLEQLGIL
OXA-51-like_SQI55469.1_A.b.  NIVAFSLNLEMKKGIPSSVRKEITYKSLEQLGIL
OXA-51-like_QJH03197.1_A.b.  NIVAFSLNLEMKKGIPSSVRKEITYKSLEQLGIL
OXA-51-like_QHB90233.1_A.b.  NIVAFSLNLEMKKGISSSVRKEITYRSLEQLGIL
OXA-51-like_BBR73296.1_A.b.  NIVAFSLNLEMKKGIPSSVRKEITYKSLEQLGIL
                        acc
```

OXA-58-like subfamily

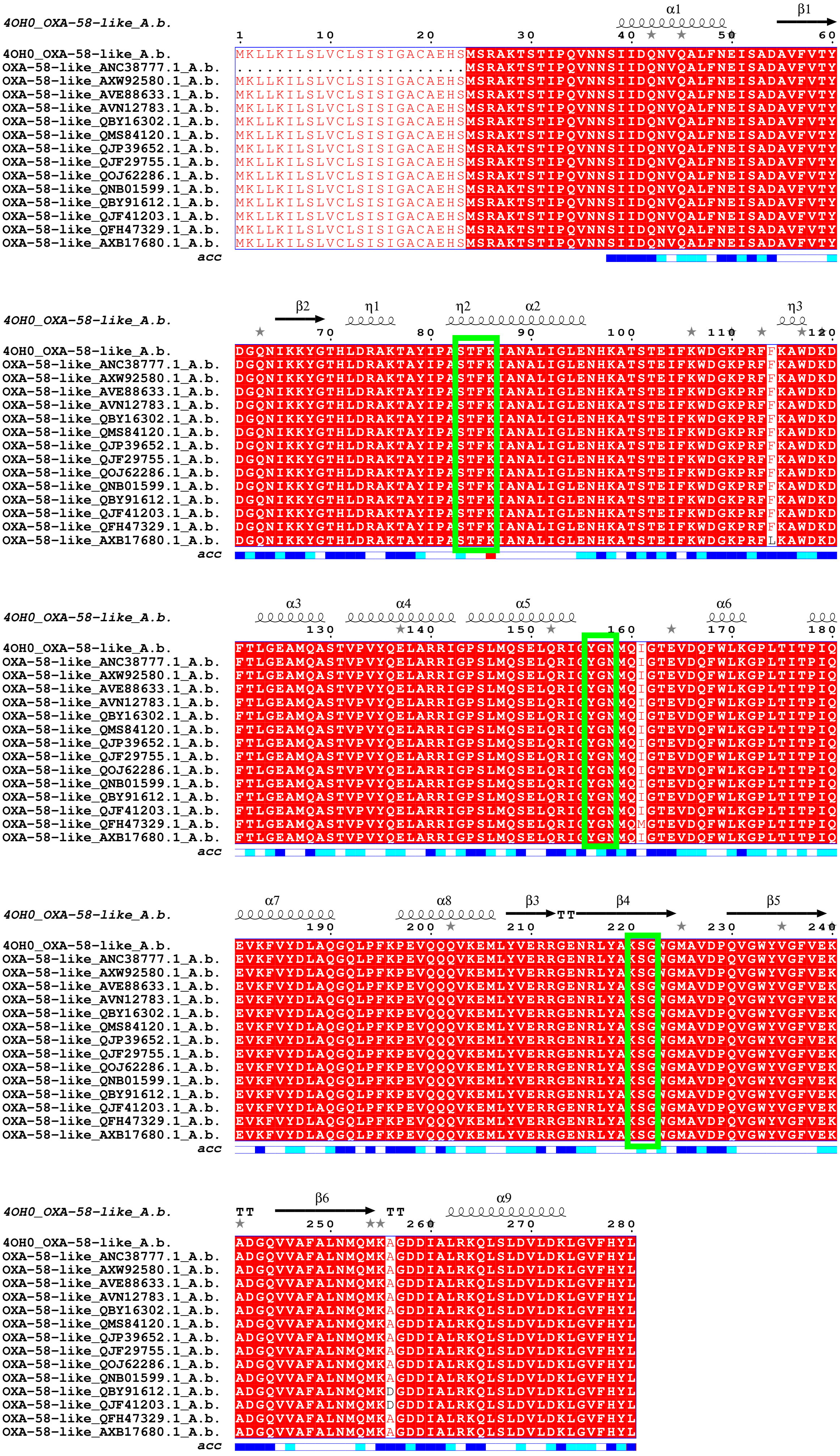

OXA-134-like subfamily

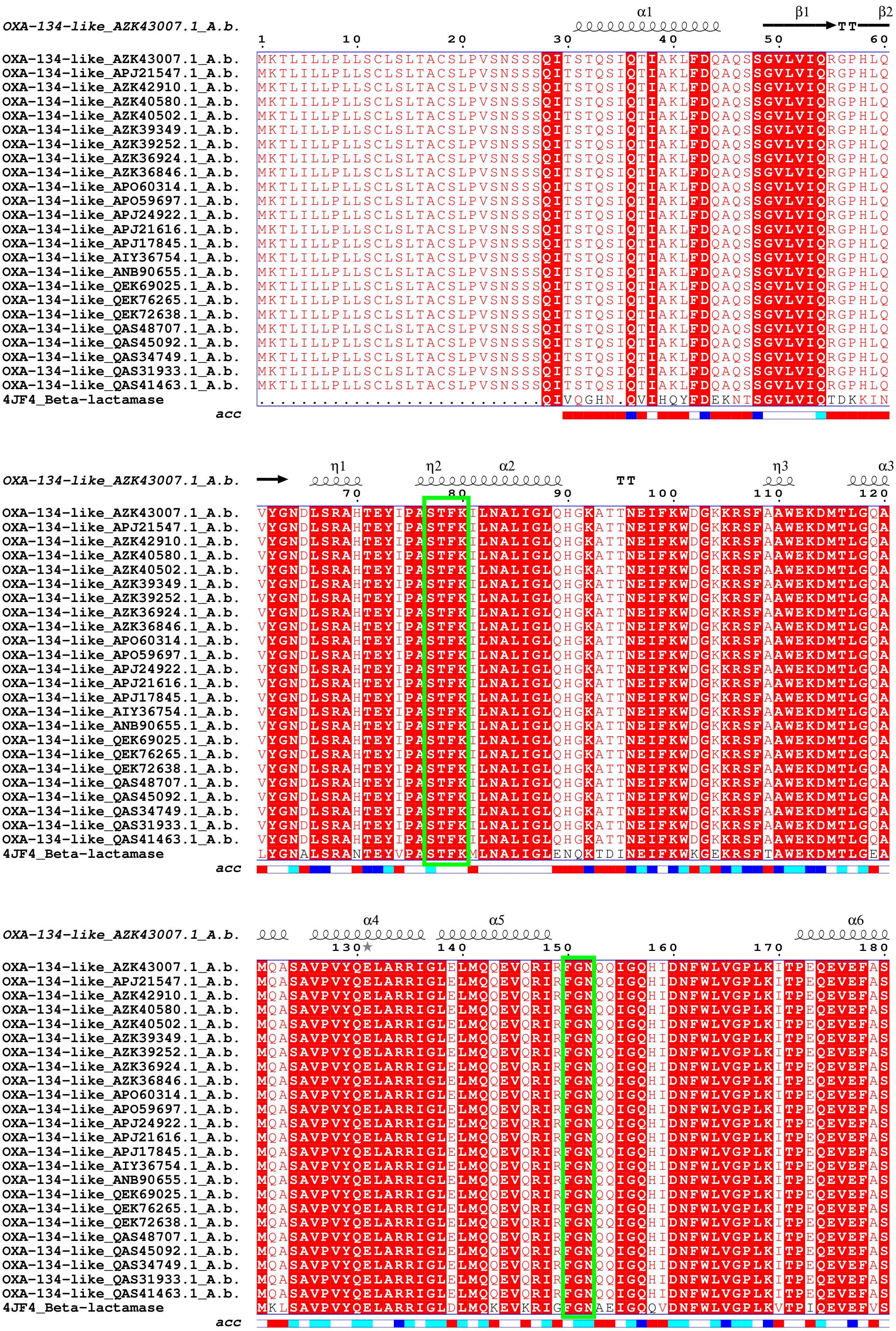

OXA-134-like subfamily

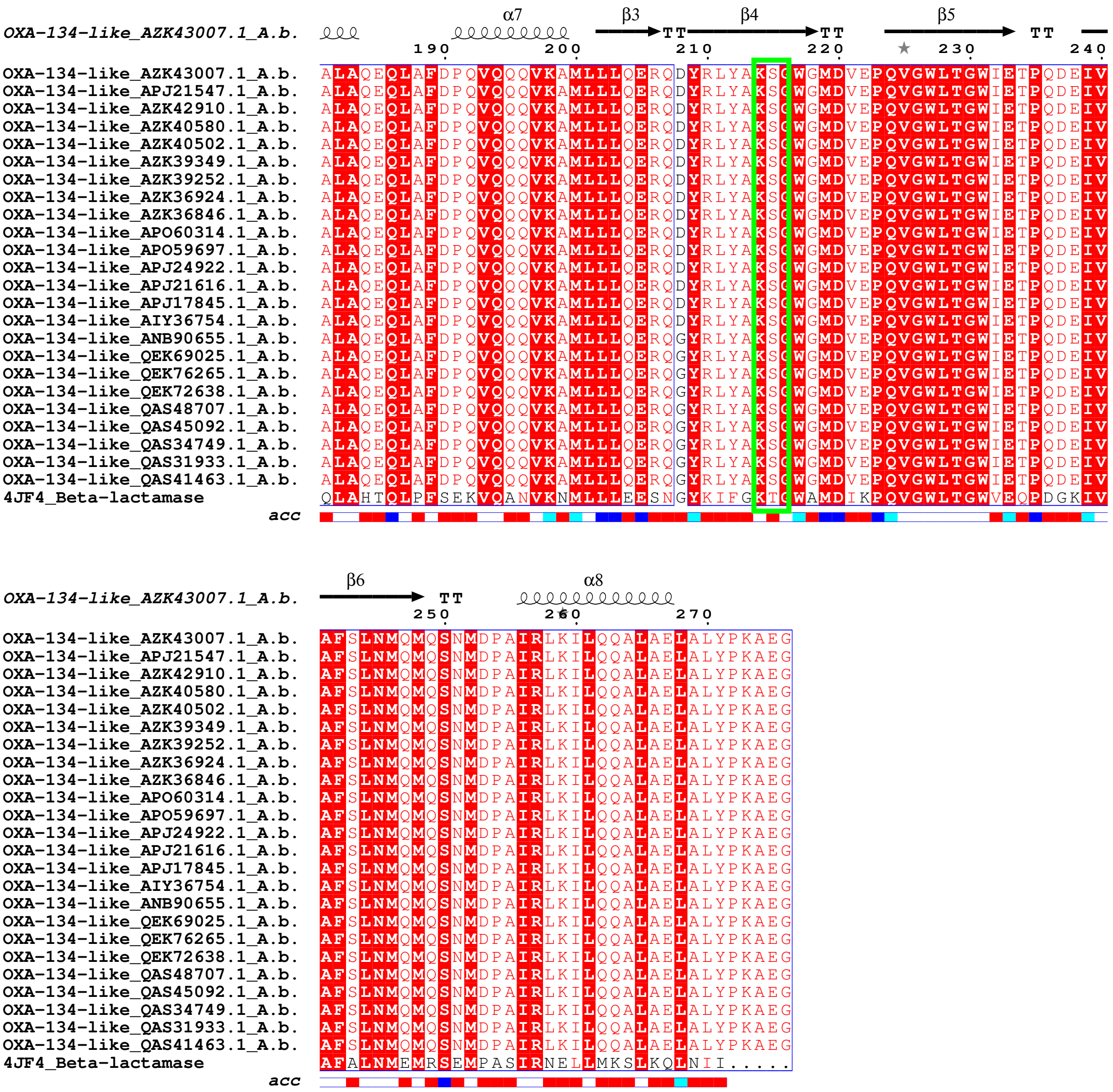

OXA-213-like subfamily

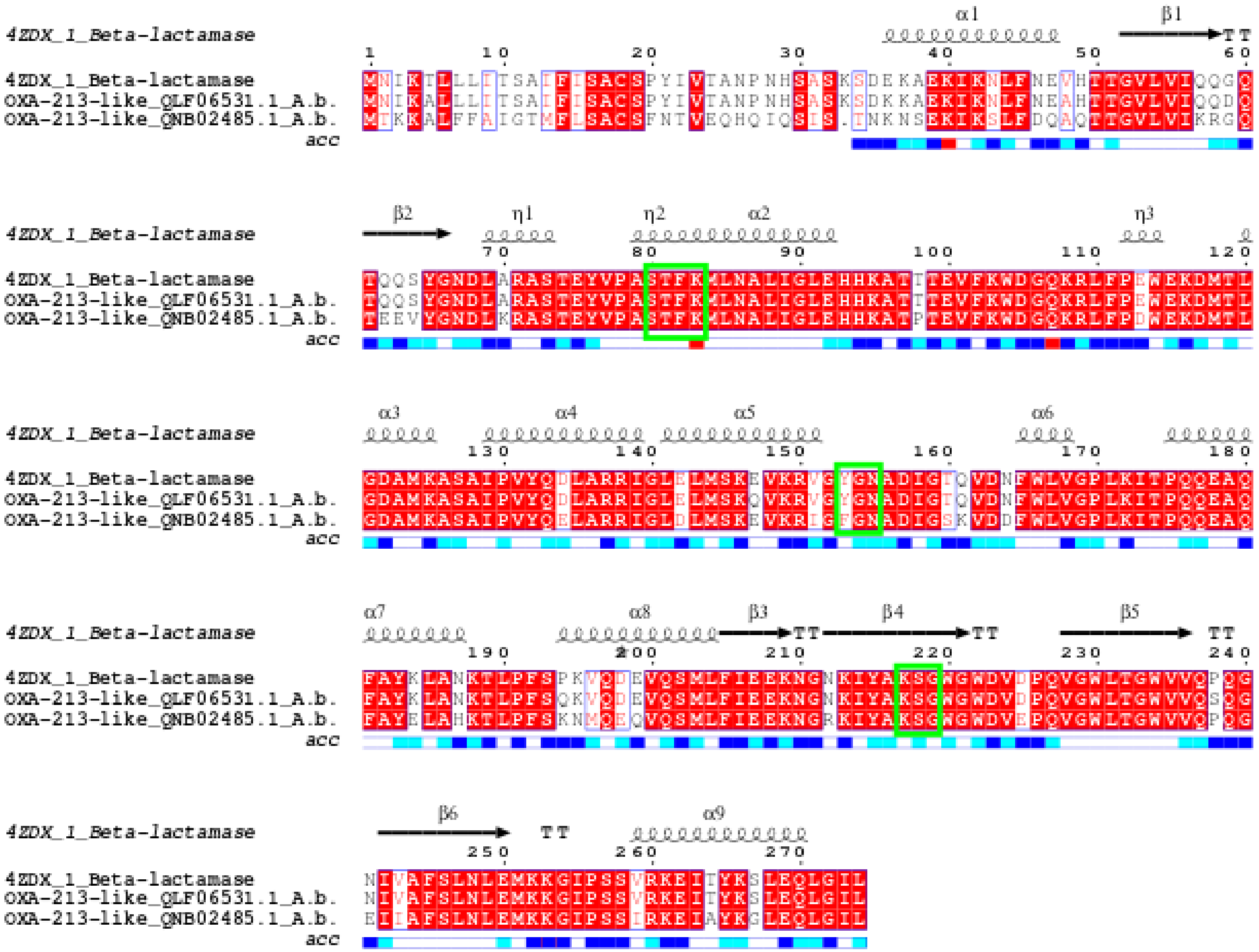

OXA-48-like subfamily

```
                                                                 β1     η1   α1          β2          β3      α2
*OXA-48-like_QRC22072.1_K.p.*
                                1         10        20        30        40        50        60
OXA-48-like_QRC22072.1_K.p.     MRVLALSAVFLVASIIGMPAVAKEWQENKSWNAHFTEHKSQGVVVLWNENKQQGFTNNLK
OXA-48-like_ASG42064.1_E.spp.   MRVLALSAVFLVASIIGMPAVAKEWQENKSWNAHFTEHKSQGVVVLWNENKQQGFTNNLK
OXA-48-like_QRC13711.1_K.p.     MRVLALSAVFLVASIIGMPAVAKEWQENKSWNAHFTEHKSQGVVVLWNENKQQGFTNNLK
OXA-48-like_VAY01821.1_K.p.     MRVLALSAVFLVASIIGMPAVAKEWQENKSWNAHFTEHKSQGVVVLWNENKQQGFTNNLK
OXA-48-like_VAX91950.1_K.p.     MRVLALSAVFLVASIIGMPAVAKEWQENKSWNAHFTEHKSQGVVVLWNENKQQGFTNNLK
OXA-48-like_VAX91959.1_K.p.     MRVLALSAVFLVASIIGMPAVAKEWQENKSWNAHFTEHKSQGVVVLWNENKQQGFTNNLK
OXA-48-like_QPP62592.1_K.p.     MRVLALSAVFLVASIIGMPAVAKEWQENKSWNAHFTEHKSQGVVVLWNENKQQGFTNNLK
OXA-48-like_QOU38957.1_K.p.     MRVLALSAVFLVASIIGMPAVAKEWQENKSWNAHFTEHKSQGVVVLWNENKQQGFTNNLK
OXA-48-like_QOT97232.1_K.p.     MRVLALSAVFLVASIIGMPAVAKEWQENKSWNAHFTEHKSQGVVVLWNENKQQGFTNNLK
OXA-48-like_QLI33314.1_K.p.     MRVLALSAVFLVASIIGMPAVAKEWQENKSWNAHFTEHKSQGVVVLWNENKQQGFTNNLK
OXA-48-like_QLI27956.1_K.p.     MRVLALSAVFLVASIIGMPAVAKEWQENKSWNAHFTEHKSQGVVVLWNENKQQGFTNNLK
OXA-48-like_QLI22474.1_K.p.     MRVLALSAVFLVASIIGMPAVAKEWQENKSWNAHFTEHKSQGVVVLWNENKQQGFTNNLK
OXA-48-like_QLC16065.1_K.p.     MRVLALSAVFLVASIIGMPAVAKEWQENKSWNAHFTEHKSQGVVVLWNENKQQGFTNNLK
OXA-48-like_QLC05384.1_K.p.     MRVLALSAVFLVASIIGMPAVAKEWQENKSWNAHFTEHKSQGVVVLWNENKQQGFTNNLK
OXA-48-like_QLC00099.1_K.p.     MRVLALSAVFLVASIIGMPAVAKEWQENKSWNAHFTEHKSQGVVVLWNENKQQGFTNNLK
OXA-48-like_QLB94822.1_K.p.     MRVLALSAVFLVASIIGMPAVAKEWQENKSWNAHFTEHKSQGVVVLWNENKQQGFTNNLK
OXA-48-like_QLB89481.1_K.p.     MRVLALSAVFLVASIIGMPAVAKEWQENKSWNAHFTEHKSQGVVVLWNENKQQGFTNNLK
OXA-48-like_QLB84144.1_K.p.     MRVLALSAVFLVASIIGMPAVAKEWQENKSWNAHFTEHKSQGVVVLWNENKQQGFTNNLK
OXA-48-like_QLB78805.1_K.p.     MRVLALSAVFLVASIIGMPAVAKEWQENKSWNAHFTEHKSQGVVVLWNENKQQGFTNNLK
OXA-48-like_QLB73478.1_K.p.     MRVLALSAVFLVASIIGMPAVAKEWQENKSWNAHFTEHKSQGVVVLWNENKQQGFTNNLK
OXA-48-like_QIJ23388.1_K.p.     MRVLALSAVFLVASIIGMPAVAKEWQENKSWNAHFTEHKSQGVVVLWNENKQQGFTNNLK
OXA-48-like_QIJ17785.1_K.p.     MRVLALSAVFLVASIIGMPAVAKEWQENKSWNAHFTEHKSQGVVVLWNENKQQGFTNNLK
OXA-48-like_QND07059.1_K.p.     MRVLALSAVFLVASIIGMPAVAKEWQENKSWNAHFTEHKSQGVVVLWNENKQQGFTNNLK
OXA-48-like_QET12150.1_K.p.     MRVLALSAVFLVASIIGMPAVAKEWQENKSWNAHFTEHKSQGVVVLWNENKQQGFTNNLK
OXA-48-like_QCS89961.1_K.p.     MRVLALSAVFLVASIIGMPAVAKEWQENKSWNAHFTEHKSQGVVVLWNENKQQGFTNNLK
OXA-48-like_QCT00720.1_K.p.     MRVLALSAVFLVASIIGMPAVAKEWQENKSWNAHFTEHKSQGVVVLWNENKQQGFTNNLK
OXA-48-like_QDF35925.1_K.p.     MRVLALSAVFLVASIIGMPAVAKEWQENKSWNAHFTEHKSQGVVVLWNENKQQGFTNNLK
OXA-48-like_AZJ02288.1_K.p.     MRVLALSAVFLVASIIGMPAVAKEWQENKSWNAHFTEHKSQGVVVLWNENKQQGFTNNLK
OXA-48-like_AZI07598.1_K.p.     MRVLALSAVFLVASIIGMPAVAKEWQENKSWNAHFTEHKSQGVVVLWNENKQQGFTNNLK
OXA-48-like_AXZ24690.1_K.p.     MRVLALSAVFLVASIIGMPAVAKEWQENKSWNAHFTEHKSQGVVVLWNENKQQGFTNNLK
OXA-48-like_AXS22834.1_K.p.     MRVLALSAVFLVASIIGMPAVAKEWQENKSWNAHFTEHKSQGVVVLWNENKQQGFTNNLK
OXA-48-like_AXR54472.1_K.p.     MRVLALSAVFLVASIIGMPAVAKEWQENKSWNAHFTEHKSQGVVVLWNENKQQGFTNNLK
OXA-48-like_AWR57773.1_K.p.     MRVLALSAVFLVASIIGMPAVAKEWQENKSWNAHFTEHKSQGVVVLWNENKQQGFTNNLK
OXA-48-like_ART03415.1_K.p.     MRVLALSAVFLVASIIGMPAVAKEWQENKSWNAHFTEHKSQGVVVLWNENKQQGFTNNLK
OXA-48-like_ART03346.1_K.p.     MRVLALSAVFLVASIIGMPAVAKEWQENKSWNAHFTEHKSQGVVVLWNENKQQGFTNNLK
OXA-48-like_APQ28988.1_K.p.     MRVLALSAVFLVASIIGMPAVAKEWQENKSWNAHFTEHKSQGVVVLWNENKQQGFTNNLK
OXA-48-like_APP73786.1_K.p.     MRVLALSAVFLVASIIGMPAVAKEWQENKSWNAHFTEHKSQGVVVLWNENKQQGFTNNLK
OXA-48-like_APP67917.1_K.p.     MRVLALSAVFLVASIIGMPAVAKEWQENKSWNAHFTEHKSQGVVVLWNENKQQGFTNNLK
OXA-48-like_APP56266.1_K.p.     MRVLALSAVFLVASIIGMPAVAKEWQENKSWNAHFTEHKSQGVVVLWNENKQQGFTNNLK
OXA-48-like_APP50376.1_K.p.     MRVLALSAVFLVASIIGMPAVAKEWQENKSWNAHFTEHKSQGVVVLWNENKQQGFTNNLK
OXA-48-like_APP44504.1_K.p.     MRVLALSAVFLVASIIGMPAVAKEWQENKSWNAHFTEHKSQGVVVLWNENKQQGFTNNLK
OXA-48-like_APP38728.1_K.p.     MRVLALSAVFLVASIIGMPAVAKEWQENKSWNAHFTEHKSQGVVVLWNENKQQGFTNNLK
OXA-48-like_APM79240.1_K.p.     MRVLALSAVFLVASIIGMPAVAKEWQENKSWNAHFTEHKSQGVVVLWNENKQQGFTNNLK
OXA-48-like_APM73588.1_K.p.     MRVLALSAVFLVASIIGMPAVAKEWQENKSWNAHFTEHKSQGVVVLWNENKQQGFTNNLK
OXA-48-like_APM67987.1_K.p.     MRVLALSAVFLVASIIGMPAVAKEWQENKSWNAHFTEHKSQGVVVLWNENKQQGFTNNLK
OXA-48-like_APM62354.1_K.p.     MRVLALSAVFLVASIIGMPAVAKEWQENKSWNAHFTEHKSQGVVVLWNENKQQGFTNNLK
OXA-48-like_APM47124.1_K.p.     MRVLALSAVFLVASIIGMPAVAKEWQENKSWNAHFTEHKSQGVVVLWNENKQQGFTNNLK
OXA-48-like_APG80363.1_K.p.     MRVLALSAVFLVASIIGMPAVAKEWQENKSWNAHFTEHKSQGVVVLWNENKQQGFTNNLK
OXA-48-like_AOF13070.1_K.p.     MRVLALSAVFLVASIIGMPAVAKEWQENKSWNAHFTEHKSQGVVVLWNENKQQGFTNNLK
OXA-48-like_QPQ02212.1_K.p.     MRVLALSAVFLVASIIGMPAVAKEWQENKSWNAHFTEHKSQGVVVLWNENKQQGFTNNLK
OXA-48-like_QPQ02194.1_K.p.     MRVLALSAVFLVASIIGMPAVAKEWQENKSWNAHFTEHKSQGVVVLWNENKQQGFTNNLK
OXA-48-like_QIJ34285.1_K.p.     MRVLALSAVFLVASIIGMPAVAKEWQENKSWNAHFTEHKSQGVVVLWNENKQQGFTNNLK
OXA-48-like_QIJ09605.1_K.p.     MRVLALSAVFLVASIIGMPAVAKEWQENKSWNAHFTEHKSQGVVVLWNENKQQGFTNNLK
OXA-48-like_QIR58822.1_K.p.     MRVLALSAVFLVASIIGMPAVAKEWQENKSWNAHFTEHKSQGVVVLWNENKQQGFTNNLK
5OE0_1_Beta-lactamase           MRVLALSAVFLVASIIGMPAVAKEWQENKSWNAHFTEHKSQGVVVLWNENKQQGFTNNLK
OXA-48-like_ANE69785.1_K.p.     MRVLALSAVFLVASIIGMPAVAKEWQENKSWNAHFTEHKSQGVVVLWNENKQQGFTNNLK
OXA-48-like_ANE71804.1_K.p.     MRVLALSAVFLVASIIGMPAVAKEWQENKSWNAHFTEHKSQGVVVLWNENKQQGFTNNLK
OXA-48-like_ANE72730.1_K.p.     MRVLALSAVFLVASIIGMPAVAKEWQENKSWNAHFTEHKSQGVVVLWNENKQQGFTNNLK
OXA-48-like_AOA98603.1_K.p.     MRVLALSAVFLVASIIGMPAVAKEWQENKSWNAHFTEHKSQGVVVLWNENKQQGFTNNLK
OXA-48-like_CEP69754.1_K.p.     MRVLALSAVFLVASIIGMPAVAKEWQENKSWNAHFTEHKSQGVVVLWNENKQQGFTNNLK
OXA-48-like_CEP70686.1_K.p.     MRVLALSAVFLVASIIGMPAVAKEWQENKSWNAHFTEHKSQGVVVLWNENKQQGFTNNLK
OXA-48-like_CEP72746.1_K.p.     MRVLALSAVFLVASIIGMPAVAKEWQENKSWNAHFTEHKSQGVVVLWNENKQQGFTNNLK
OXA-48-like_ATX28768.1_K.p.     MRVLALSAVFLVASIIGMPAVAKEWQENKSWNAHFTEHKSQGVVVLWNENKQQGFTNNLK
OXA-48-like_ATX29640.1_K.p.     MRVLALSAVFLVASIIGMPAVAKEWQENKSWNAHFTEHKSQGVVVLWNENKQQGFTNNLK
OXA-48-like_AVO14768.1_K.p.     MRVLALSAVFLVASIIGMPAVAKEWQENKSWNAHFTEHKSQGVVVLWNENKQQGFTNNLK
OXA-48-like_AVO15618.1_K.p.     MRVLALSAVFLVASIIGMPAVAKEWQENKSWNAHFTEHKSQGVVVLWNENKQQGFTNNLK
OXA-48-like_AWD00385.1_K.p.     MRVLALSAVFLVASIIGMPAVAKEWQENKSWNAHFTEHKSQGVVVLWNENKQQGFTNNLK
OXA-48-like_AWD98100.1_K.p.     MRVLALSAVFLVASIIGMPAVAKEWQENKSWNAHFTEHKSQGVVVLWNENKQQGFTNNLK
OXA-48-like_AWS85277.1_K.p.     MRVLALSAVFLVASIIGMPAVAKEWQENKSWNAHFTEHKSQGVVVLWNENKQQGFTNNLK
OXA-48-like_QEG84876.1_K.p.     MRVLALSAVFLVASIIGMPAVAKEWQENKSWNAHFTEHKSQGVVVLWNENKQQGFTNNLK
OXA-48-like_QIR47572.1_K.p.     MRVLALSAVFLVASIIGMPAVAKEWQENKSWNAHFTEHKSQGVVVLWNENKQQGFTNNLK
OXA-48-like_QIR53202.1_K.p.     MRVLALSAVFLVASIIGMPAVAKEWQENKSWNAHFTEHKSQGVVVLWNENKQQGFTNNLK
OXA-48-like_QOQ50577.1_K.p.     MRVLALSAVFLVASIIGMPAVAKEWQENKSWNAHFTEHKSQGVVVLWNENKQQGFTNNLK
OXA-48-like_BBO98227.1_K.p.     MRVLALSAVFLVASIIGMPAVAKEWQENKSWNAHFTEHKSQGVVVLWNENKQQGFTNNLK
OXA-48-like_BBO99129.1_K.p.     MRVLALSAVFLVASIIGMPAVAKEWQENKSWNAHFTEHKSQGVVVLWNENKQQGFTNNLK
OXA-48-like_QDE47101.1_E.spp.   MRVLALSAVFLVASIIGMPAVAKEWQENKSWNAHFTEHKSQGVVVLWNENKQQGFTNNLK
OXA-48-like_AIG86783.1_K.p.     MRVLALSAVFLVASIIGMPAVAKEWQENKSWNAHFTEHKSQGVVVLWNENKQQGFTNNLK
OXA-48-like_ALH88756.1_K.p.     MRVLALSAVFLVASIIGMPAVAKEWQENKSWNAHFTEHKSQGVVVLWNENKQQGFTNNLK
OXA-48-like_AOF02466.1_K.p.     MRVLALSAVFLVASIIGMPAVAKEWQENKSWNAHFTEHKSQGVVVLWNENKQQGFTNNLK
OXA-48-like_ATO26616.1_K.p.     MRVLALSAVFLVASIIGMPAVAKEWQENKSWNAHFTEHKSQGVVVLWNENKQQGFTNNLK
OXA-48-like_AZB79861.1_K.p.     MRVLALSAVFLVASIIGMPAVAKEWQENKSWNAHFTEHKSQGVVVLWNENKQQGFTNNLK
OXA-48-like_AZV17592.1_K.p.     MRVLALSAVFLVASIIGMPAVAKEWQENKSWNAHFTEHKSQGVVVLWNENKQQGFTNNLK
OXA-48-like_QBG05392.1_K.p.     MRVLALSAVFLVASIIGMPAVAKEWQENKSWNAHFTEHKSQGVVVLWNENKQQGFTNNLK
OXA-48-like_QBH46740.1_K.p.     MRVLALSAVFLVASIIGMPAVAKEWQENKSWNAHFTEHKSQGVVVLWNENKQQGFTNNLK
OXA-48-like_QBH61269.1_K.p.     MRVLALSAVFLVASIIGMPAVAKEWQENKSWNAHFTEHKSQGVVVLWNENKQQGFTNNLK
OXA-48-like_QLF61798.1_K.p.     MRVLALSAVFLVASIIGMPAVAKEWQENKSWNAHFTEHKSQGVVVLWNENKQQGFTNNLK
OXA-48-like_QLF61822.1_K.p.     MRVLALSAVFLVASIIGMPAVAKEWQENKSWNAHFTEHKSQGVVVLWNENKQQGFTNNLK
                          acc
```

OXA-48-like subfamily

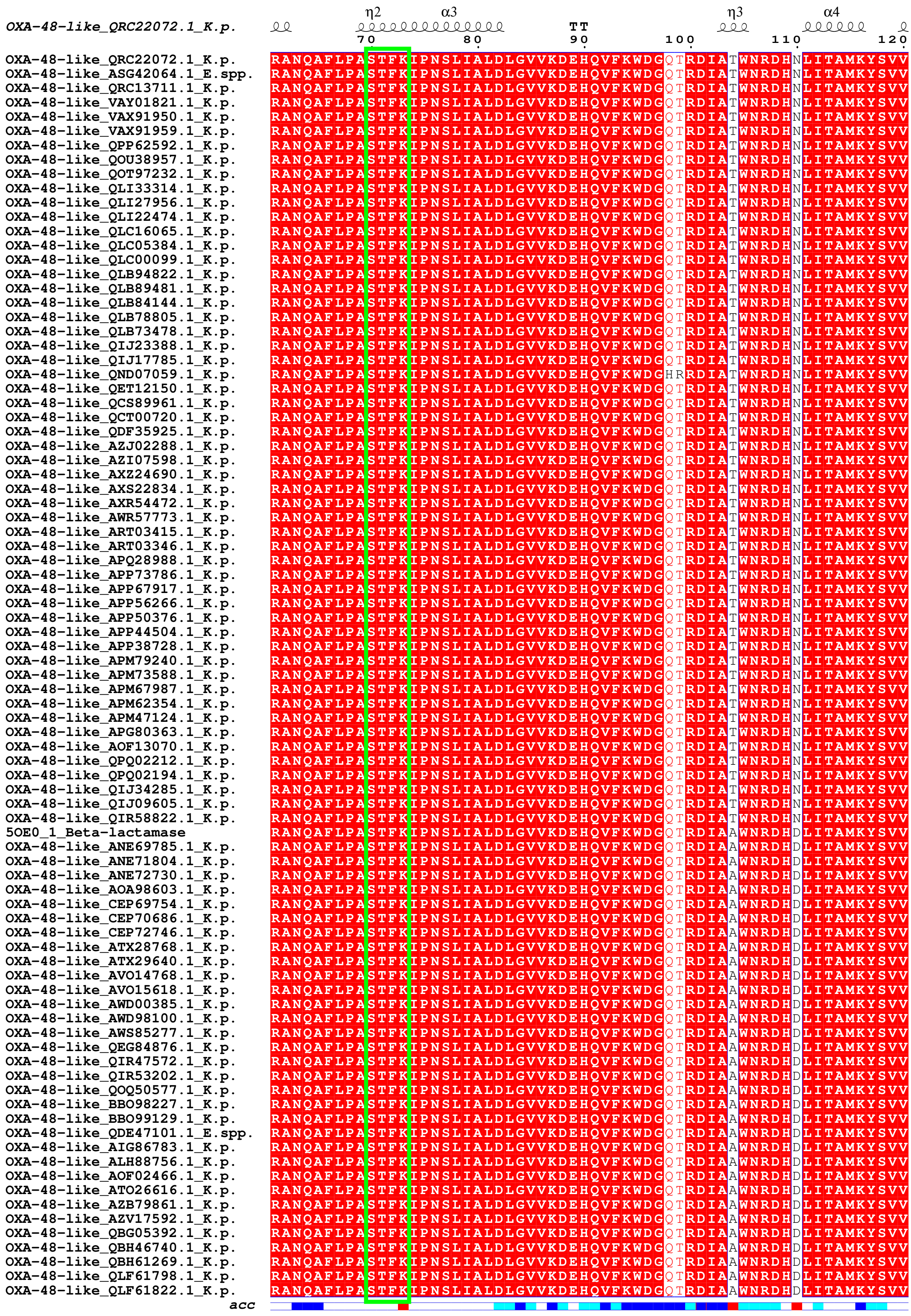

OXA-198-like subfamily

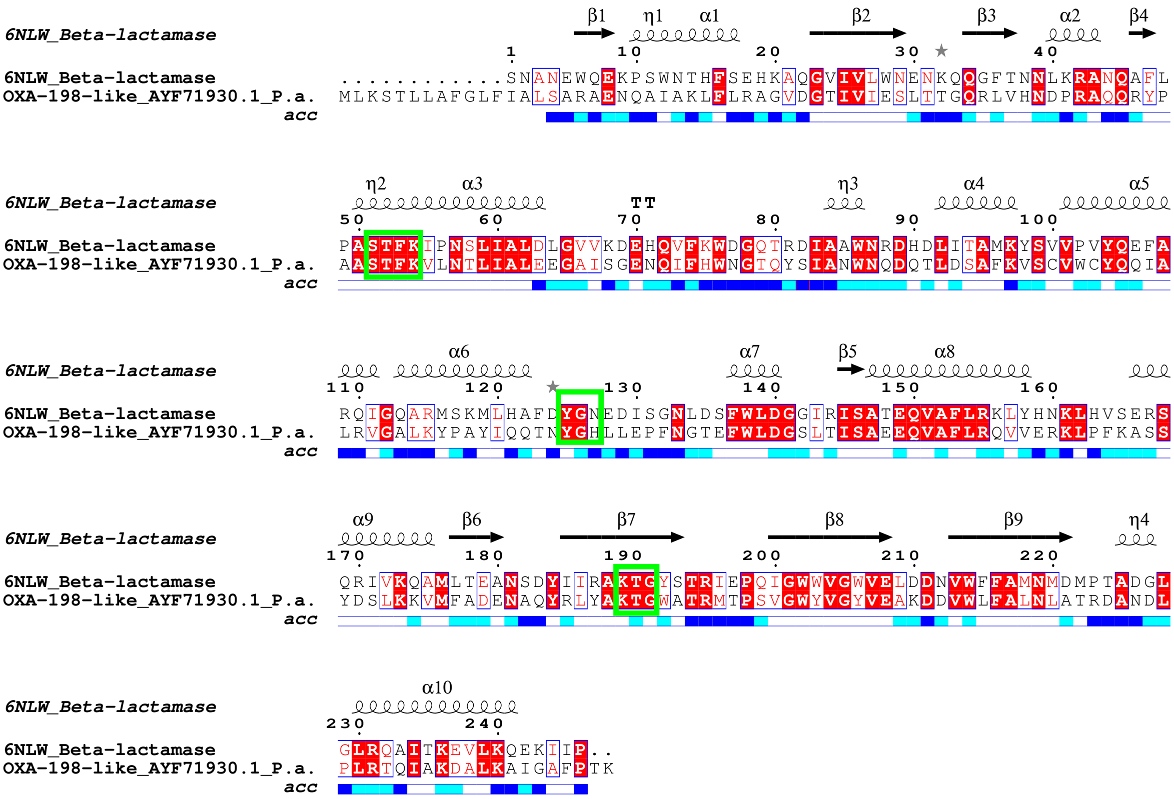

OXA-48-like subfamily

```
                                   α5           α6                    α7            α8
OXA-48-like_QRC22072.1_K.p.
                                         130       140       150       160       170       180
OXA-48-like_QRC22072.1_K.p.     PVYQEFARQIGEARMSKMLHAFDYGNEDISGNVDSFWLDGGIRISATEQISFLRKLYHNK
OXA-48-like_ASG42064.1_E.spp.   PVYQEFARQIGEARMSKMLHAFDYGNEDISGNVDSFWLDGGIRISATEQISFLRKLYHNK
OXA-48-like_QRC13711.1_K.p.     PVYQEFARQIGEARMSKMLHAFDYGNEDISGNVDSFWLDGGIRISATEQISFLRKLYHNK
OXA-48-like_VAY01821.1_K.p.     PVYQEFARQIGEARMSKMLHAFDYGNEDISGNVDSFWLDGGIRISATEQISFLRKLYHNK
OXA-48-like_VAX91950.1_K.p.     PVYQEFARQIGEARMSKMLHAFDYGNEDISGNVDSFWLDGGIRISATEQISFLRKLYHNK
OXA-48-like_VAX91959.1_K.p.     PVYQEFARQIGEARMSKMLHAFDYGNEDISGNVDSFWLDGGIRISATEQISFLRKLYHNK
OXA-48-like_QPP62592.1_K.p.     PVYQEFARQIGEARMSKMLHAFDYGNEDISGNVDSFWLDGGIRISATEQISFLRKLYHNK
OXA-48-like_QOU38957.1_K.p.     PVYQEFARQIGEARMSKMLHAFDYGNEDISGNVDSFWLDGGIRISATEQISFLRKLYHNK
OXA-48-like_QOT97232.1_K.p.     PVYQEFARQIGEARMSKMLHAFDYGNEDISGNVDSFWLDGGIRISATEQISFLRKLYHNK
OXA-48-like_QLI33314.1_K.p.     PVYQEFARQIGEARMSKMLHAFDYGNEDISGNVDSFWLDGGIRISATEQISFLRKLYHNK
OXA-48-like_QLI27956.1_K.p.     PVYQEFARQIGEARMSKMLHAFDYGNEDISGNVDSFWLDGGIRISATEQISFLRKLYHNK
OXA-48-like_QLI22474.1_K.p.     PVYQEFARQIGEARMSKMLHAFDYGNEDISGNVDSFWLDGGIRISATEQISFLRKLYHNK
OXA-48-like_QLC16065.1_K.p.     PVYQEFARQIGEARMSKMLHAFDYGNEDISGNVDSFWLDGGIRISATEQISFLRKLYHNK
OXA-48-like_QLC05384.1_K.p.     PVYQEFARQIGEARMSKMLHAFDYGNEDISGNVDSFWLDGGIRISATEQISFLRKLYHNK
OXA-48-like_QLC00099.1_K.p.     PVYQEFARQIGEARMSKMLHAFDYGNEDISGNVDSFWLDGGIRISATEQISFLRKLYHNK
OXA-48-like_QLB94822.1_K.p.     PVYQEFARQIGEARMSKMLHAFDYGNEDISGNVDSFWLDGGIRISATEQISFLRKLYHNK
OXA-48-like_QLB89481.1_K.p.     PVYQEFARQIGEARMSKMLHAFDYGNEDISGNVDSFWLDGGIRISATEQISFLRKLYHNK
OXA-48-like_QLB84144.1_K.p.     PVYQEFARQIGEARMSKMLHAFDYGNEDISGNVDSFWLDGGIRISATEQISFLRKLYHNK
OXA-48-like_QLB78805.1_K.p.     PVYQEFARQIGEARMSKMLHAFDYGNEDISGNVDSFWLDGGIRISATEQISFLRKLYHNK
OXA-48-like_QLB73478.1_K.p.     PVYQEFARQIGEARMSKMLHAFDYGNEDISGNVDSFWLDGGIRISATEQISFLRKLYHNK
OXA-48-like_QIJ23388.1_K.p.     PVYQEFARQIGEARMSKMLHAFDYGNEDISGNVDSFWLDGGIRISATEQISFLRKLYHNK
OXA-48-like_QIJ17785.1_K.p.     PVYQEFARQIGEARMSKMLHAFDYGNEDISGNVDSFWLDGGIRISATEQISFLRKLYHNK
OXA-48-like_QND07059.1_K.p.     PVYQEFARQIGEARMSKMLHAFDYGNEDISGNVDSFWLDGGIRISATEQISFLRKLYHNK
OXA-48-like_QET12150.1_K.p.     PVYQEFARQIGEARMSKMLHAFDYGNEDISGNVDSFWLDGGIRISATEQISFLRKLYHNK
OXA-48-like_QCS89961.1_K.p.     PVYQEFARQIGEARMSKMLHAFDYGNEDISGNVDSFWLDGGIRISATEQISFLRKLYHNK
OXA-48-like_QCT00720.1_K.p.     PVYQEFARQIGEARMSKMLHAFDYGNEDISGNVDSFWLDGGIRISATEQISFLRKLYHNK
OXA-48-like_QDF35925.1_K.p.     PVYQEFARQIGEARMSKMLHAFDYGNEDISGNVDSFWLDGGIRISATEQISFLRKLYHNK
OXA-48-like_AZJ02288.1_K.p.     PVYQEFARQIGEARMSKMLHAFDYGNEDISGNVDSFWLDGGIRISATEQISFLRKLYHNK
OXA-48-like_AZI07598.1_K.p.     PVYQEFARQIGEARMSKMLHAFDYGNEDISGNVDSFWLDGGIRISATEQISFLRKLYHNK
OXA-48-like_AXZ24690.1_K.p.     PVYQEFARQIGEARMSKMLHAFDYGNEDISGNVDSFWLDGGIRISATEQISFLRKLYHNK
OXA-48-like_AXS22834.1_K.p.     PVYQEFARQIGEARMSKMLHAFDYGNEDISGNVDSFWLDGGIRISATEQISFLRKLYHNK
OXA-48-like_AXR54472.1_K.p.     PVYQEFARQIGEARMSKMLHAFDYGNEDISGNVDSFWLDGGIRISATEQISFLRKLYHNK
OXA-48-like_AWR57773.1_K.p.     PVYQEFARQIGEARMSKMLHAFDYGNEDISGNVDSFWLDGGIRISATEQISFLRKLYHNK
OXA-48-like_ART03415.1_K.p.     PVYQEFARQIGEARMSKMLHAFDYGNEDISGNVDSFWLDGGIRISATEQISFLRKLYHNK
OXA-48-like_ART03346.1_K.p.     PVYQEFARQIGEARMSKMLHAFDYGNEDISGNVDSFWLDGGIRISATEQISFLRKLYHNK
OXA-48-like_APQ28988.1_K.p.     PVYQEFARQIGEARMSKMLHAFDYGNEDISGNVDSFWLDGGIRISATEQISFLRKLYHNK
OXA-48-like_APP73786.1_K.p.     PVYQEFARQIGEARMSKMLHAFDYGNEDISGNVDSFWLDGGIRISATEQISFLRKLYHNK
OXA-48-like_APP67917.1_K.p.     PVYQEFARQIGEARMSKMLHAFDYGNEDISGNVDSFWLDGGIRISATEQISFLRKLYHNK
OXA-48-like_APP56266.1_K.p.     PVYQEFARQIGEARMSKMLHAFDYGNEDISGNVDSFWLDGGIRISATEQISFLRKLYHNK
OXA-48-like_APP50376.1_K.p.     PVYQEFARQIGEARMSKMLHAFDYGNEDISGNVDSFWLDGGIRISATEQISFLRKLYHNK
OXA-48-like_APP44504.1_K.p.     PVYQEFARQIGEARMSKMLHAFDYGNEDISGNVDSFWLDGGIRISATEQISFLRKLYHNK
OXA-48-like_APP38728.1_K.p.     PVYQEFARQIGEARMSKMLHAFDYGNEDISGNVDSFWLDGGIRISATEQISFLRKLYHNK
OXA-48-like_APM79240.1_K.p.     PVYQEFARQIGEARMSKMLHAFDYGNEDISGNVDSFWLDGGIRISATEQISFLRKLYHNK
OXA-48-like_APM73588.1_K.p.     PVYQEFARQIGEARMSKMLHAFDYGNEDISGNVDSFWLDGGIRISATEQISFLRKLYHNK
OXA-48-like_APM67987.1_K.p.     PVYQEFARQIGEARMSKMLHAFDYGNEDISGNVDSFWLDGGIRISATEQISFLRKLYHNK
OXA-48-like_APM62354.1_K.p.     PVYQEFARQIGEARMSKMLHAFDYGNEDISGNVDSFWLDGGIRISATEQISFLRKLYHNK
OXA-48-like_APM47124.1_K.p.     PVYQEFARQIGEARMSKMLHAFDYGNEDISGNVDSFWLDGGIRISATEQISFLRKLYHNK
OXA-48-like_APG80363.1_K.p.     PVYQEFARQIGEARMSKMLHAFDYGNEDISGNVDSFWLDGGIRISATEQISFLRKLYHNK
OXA-48-like_AOF13070.1_K.p.     PVYQEFARQIGEARMSKMLHAFDYGNEDISGNVDSFWLDGGIRISATEQISFLRKLYHNK
OXA-48-like_QPQ02212.1_K.p.     PVYQEFARQIGEARMSKMLHAFDYGNEDISGNVDSFWLDGGIRISATEQISFLRKLYHNK
OXA-48-like_QPQ02194.1_K.p.     PVYQEFARQIGEARMSKMLHAFDYGNEDISGNVDSFWLDGGIRISATEQISFLRKLYHNK
OXA-48-like_QIJ34285.1_K.p.     PVYQEFARQIGEARMSKMLHAFDYGNEDISGNVDSFWLDGGIRISATEQISFLRKLYHNK
OXA-48-like_QIJ09605.1_K.p.     PVYQEFARQIGEARMSKMLHAFDYGNEDISGNVDSFWLDGGIRISATEQISFLRKLYHNK
OXA-48-like_QIR58822.1_K.p.     PVYQEFARQIGEARMSKMLHAFDYGNEDISGNVDSFWLDGGIRISATEQISFLRKLYHNK
5OE0_1_Beta-lactamase           PVYQEFARQIGEARMSKMLHAFDYGNEDISGNVDSFWLDGGIRISATQQIAFLRKLYHNK
OXA-48-like_ANE69785.1_K.p.     PVYQEFARQIGEARMSKMLHAFDYGNEDISGNVDSFWLDGGIRISATQQIAFLRKLYHNK
OXA-48-like_ANE71804.1_K.p.     PVYQEFARQIGEARMSKMLHAFDYGNEDISGNVDSFWLDGGIRISATQQIAFLRKLYHNK
OXA-48-like_ANE72730.1_K.p.     PVYQEFARQIGEARMSKMLHAFDYGNEDISGNVDSFWLDGGIRISATQQIAFLRKLYHNK
OXA-48-like_AOA98603.1_K.p.     PVYQEFARQIGEARMSKMLHAFDYGNEDISGNVDSFWLDGGIRISATQQIAFLRKLYHNK
OXA-48-like_CEP69754.1_K.p.     PVYQEFARQIGEARMSKMLHAFDYGNEDISGNVDSFWLDGGIRISATQQIAFLRKLYHNK
OXA-48-like_CEP70686.1_K.p.     PVYQEFARQIGEARMSKMLHAFDYGNEDISGNVDSFWLDGGIRISATQQIAFLRKLYHNK
OXA-48-like_CEP72746.1_K.p.     PVYQEFARQIGEARMSKMLHAFDYGNEDISGNVDSFWLDGGIRISATQQIAFLRKLYHNK
OXA-48-like_ATX28768.1_K.p.     PVYQEFARQIGEARMSKMLHAFDYGNEDISGNVDSFWLDGGIRISATQQIAFLRKLYHNK
OXA-48-like_ATX29640.1_K.p.     PVYQEFARQIGEARMSKMLHAFDYGNEDISGNVDSFWLDGGIRISATQQIAFLRKLYHNK
OXA-48-like_AVO14768.1_K.p.     PVYQEFARQIGEARMSKMLHAFDYGNEDISGNVDSFWLDGGIRISATQQIAFLRKLYHNK
OXA-48-like_AVO15618.1_K.p.     PVYQEFARQIGEARMSKMLHAFDYGNEDISGNVDSFWLDGGIRISATQQIAFLRKLYHNK
OXA-48-like_AWD00385.1_K.p.     PVYQEFARQIGEARMSKMLHAFDYGNEDISGNVDSFWLDGGIRISATQQIAFLRKLYHNK
OXA-48-like_AWD98100.1_K.p.     PVYQEFARQIGEARMSKMLHAFDYGNEDISGNVDSFWLDGGIRISATQQIAFLRKLYHNK
OXA-48-like_AWS85277.1_K.p.     PVYQEFARQIGEARMSKMLHAFDYGNEDISGNVDSFWLDGGIRISATQQIAFLRKLYHNK
OXA-48-like_QEG84876.1_K.p.     PVYQEFARQIGEARMSKMLHAFDYGNEDISGNVDSFWLDGGIRISATQQIAFLRKLYHNK
OXA-48-like_QIR47572.1_K.p.     PVYQEFARQIGEARMSKMLHAFDYGNEDISGNVDSFWLDGGIRISATQQIAFLRKLYHNK
OXA-48-like_QIR53202.1_K.p.     PVYQEFARQIGEARMSKMLHAFDYGNEDISGNVDSFWLDGGIRISATQQIAFLRKLYHNK
OXA-48-like_QOQ50577.1_K.p.     PVYQEFARQIGEARMSKMLHAFDYGNEDISGNVDSFWLDGGIRISATQQIAFLRKLYHNK
OXA-48-like_BBO98227.1_K.p.     PVYQEFARQIGEARMSKMLHAFDYGNEDISGNVDSFWLDGGIRISATQQIAFLRKLYHNK
OXA-48-like_BBO99129.1_K.p.     PVYQEFARQIGEARMSKMLHAFDYGNEDISGNVDSFWLDGGIRISATQQIAFLRKLYHNK
OXA-48-like_QDE47101.1_E.spp.   PVYQEFARQIGEARMSKMLHAFDYGNEDISGNVDSFWLDGGIRISATQQIAFLRKLYHNK
OXA-48-like_AIG86783.1_K.p.     PVYQEFARQIGEARMSKMLHAFDYGNEDISGNVDSFWLDGGIRISATQQIAFLRKLYHNK
OXA-48-like_ALH88756.1_K.p.     PVYQEFARQIGEARMSKMLHAFDYGNEDISGNVDSFWLDGGIRISATQQIAFLRKLYHNK
OXA-48-like_AOF02466.1_K.p.     PVYQEFARQIGEARMSKMLHAFDYGNEDISGNVDSFWLDGGIRISATQQIAFLRKLYHNK
OXA-48-like_ATO26616.1_K.p.     PVYQEFARQIGEARMSKMLHAFDYGNEDISGNVDSFWLDGGIRISATQQIAFLRKLYHNK
OXA-48-like_AZB79861.1_K.p.     PVYQEFARQIGEARMSKMLHAFDYGNEDISGNVDSFWLDGGIRISATQQIAFLRKLYHNK
OXA-48-like_AZV17592.1_K.p.     PVYQEFARQIGEARMSKMLHAFDYGNEDISGNVDSFWLDGGIRISATQQIAFLRKLYHNK
OXA-48-like_QBG05392.1_K.p.     PVYQEFARQIGEARMSKMLHAFDYGNEDISGNVDSFWLDGGIRISATQQIAFLRKLYHNK
OXA-48-like_QBH46740.1_K.p.     PVYQEFARQIGEARMSKMLHAFDYGNEDISGNVDSFWLDGGIRISATQQIAFLRKLYHNK
OXA-48-like_QBH61269.1_K.p.     PVYQEFARQIGEARMSKMLHAFDYGNEDISGNVDSFWLDGGIRISATQQIAFLRKLYHNK
OXA-48-like_QLF61798.1_K.p.     PVYQEFARQIGEARMSKMLHAFDYGNEDISGNVDSFWLDGGIRISATQQIAFLRKLYHNK
OXA-48-like_QLF61822.1_K.p.     PVYQEFARQIGEARMSKMLHAFDYGNEDISGNVDSFWLDGGIRISATQQIAFLRKLYHNK
                           acc
```

OXA-48-like subfamily

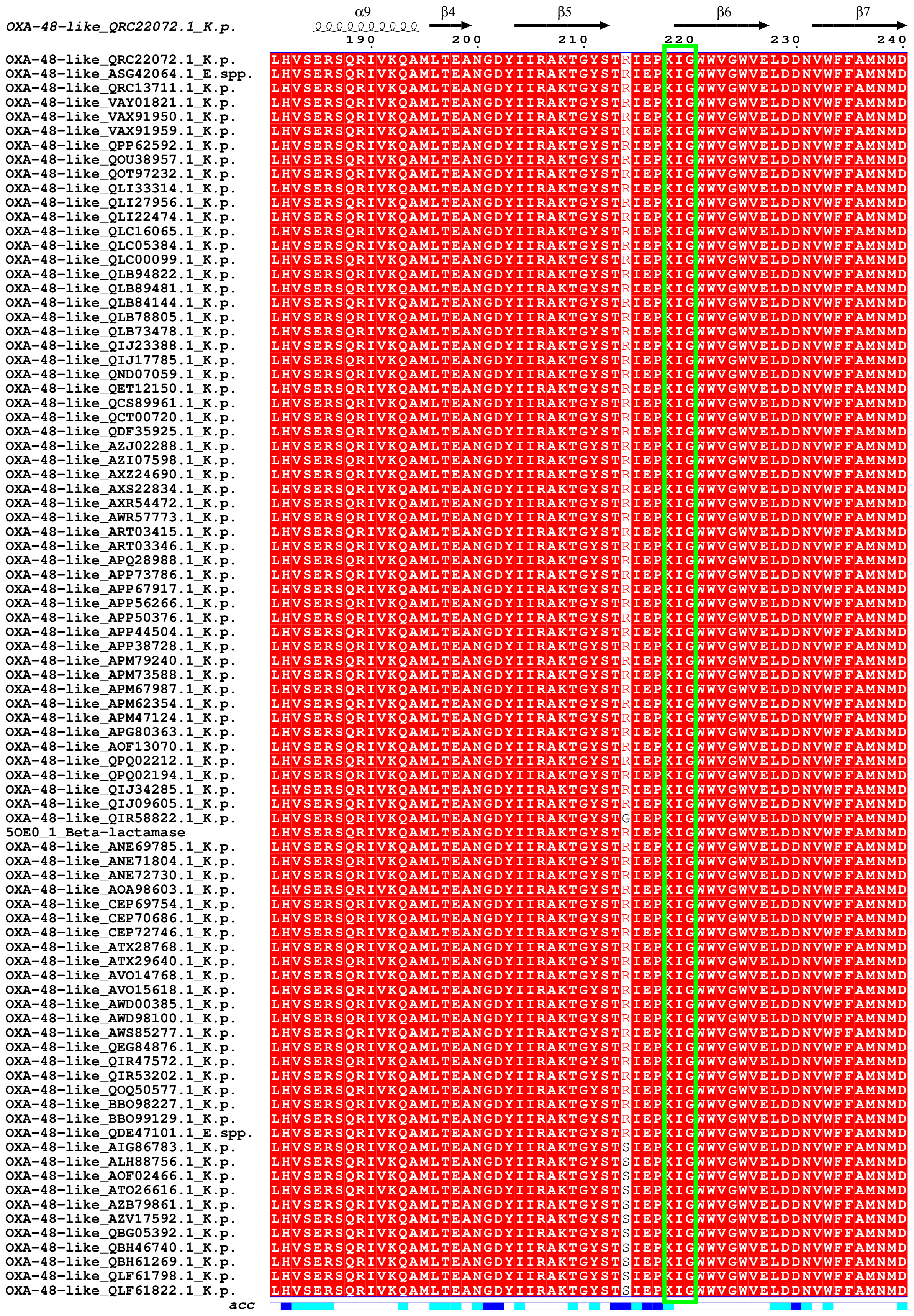

OXA-48-like subfamily

```
                                    η4       α10
OXA-48-like_QRC22072.1_K.p.                250       260
OXA-48-like_QRC22072.1_K.p.     MPTSDGLGLRQAITKEVLKQEKIIP
OXA-48-like_ASG42064.1_E.spp.   MPTSDGLGLRQAITKEVLKQEKIIP
OXA-48-like_QRC13711.1_K.p.     MPTSDGLGLRQAITKEVLKQEKIIP
OXA-48-like_VAY01821.1_K.p.     MPTSDGLGLRQAITKEVLKQEKIIP
OXA-48-like_VAX91950.1_K.p.     MPTSDGLGLRQAITKEVLKQEKIIP
OXA-48-like_VAX91959.1_K.p.     MPTSDGLGLRQAITKEVLKQEKIIP
OXA-48-like_QPP62592.1_K.p.     MPTSDGLGLRQAITKEVLKQEKIIP
OXA-48-like_QOU38957.1_K.p.     MPTSDGLGLRQAITKEVLKQEKIIP
OXA-48-like_QOT97232.1_K.p.     MPTSDGLGLRQAITKEVLKQEKIIP
OXA-48-like_QLI33314.1_K.p.     MPTSDGLGLRQAITKEVLKQEKIIP
OXA-48-like_QLI27956.1_K.p.     MPTSDGLGLRQAITKEVLKQEKIIP
OXA-48-like_QLI22474.1_K.p.     MPTSDGLGLRQAITKEVLKQEKIIP
OXA-48-like_QLC16065.1_K.p.     MPTSDGLGLRQAITKEVLKQEKIIP
OXA-48-like_QLC05384.1_K.p.     MPTSDGLGLRQAITKEVLKQEKIIP
OXA-48-like_QLC00099.1_K.p.     MPTSDGLGLRQAITKEVLKQEKIIP
OXA-48-like_QLB94822.1_K.p.     MPTSDGLGLRQAITKEVLKQEKIIP
OXA-48-like_QLB89481.1_K.p.     MPTSDGLGLRQAITKEVLKQEKIIP
OXA-48-like_QLB84144.1_K.p.     MPTSDGLGLRQAITKEVLKQEKIIP
OXA-48-like_QLB78805.1_K.p.     MPTSDGLGLRQAITKEVLKQEKIIP
OXA-48-like_QLB73478.1_K.p.     MPTSDGLGLRQAITKEVLKQEKIIP
OXA-48-like_QIJ23388.1_K.p.     MPTSDGLGLRQAITKEVLKQEKIIP
OXA-48-like_QIJ17785.1_K.p.     MPTSDGLGLRQAITKEVLKQEKIIP
OXA-48-like_QND07059.1_K.p.     MPTSDGLGLRQAITKEVLKQEKIIP
OXA-48-like_QET12150.1_K.p.     MPTSDGLGLRQAITKEVLKQEKIIP
OXA-48-like_QCS89961.1_K.p.     MPTSDGLGLRQAITKEVLKQEKIIP
OXA-48-like_QCT00720.1_K.p.     MPTSDGLGLRQAITKEVLKQEKIIP
OXA-48-like_QDF35925.1_K.p.     MPTSDGLGLRQAITKEVLKQEKIIP
OXA-48-like_AZJ02288.1_K.p.     MPTSDGLGLRQAITKEVLKQEKIIP
OXA-48-like_AZI07598.1_K.p.     MPTSDGLGLRQAITKEVLKQEKIIP
OXA-48-like_AXZ24690.1_K.p.     MPTSDGLGLRQAITKEVLKQEKIIP
OXA-48-like_AXS22834.1_K.p.     MPTSDGLGLRQAITKEVLKQEKIIP
OXA-48-like_AXR54472.1_K.p.     MPTSDGLGLRQAITKEVLKQEKIIP
OXA-48-like_AWR57773.1_K.p.     MPTSDGLGLRQAITKEVLKQEKIIP
OXA-48-like_ART03415.1_K.p.     MPTSDGLGLRQAITKEVLKQEKIIP
OXA-48-like_ART03346.1_K.p.     MPTSDGLGLRQAITKEVLKQEKIIP
OXA-48-like_APQ28988.1_K.p.     MPTSDGLGLRQAITKEVLKQEKIIP
OXA-48-like_APP73786.1_K.p.     MPTSDGLGLRQAITKEVLKQEKIIP
OXA-48-like_APP67917.1_K.p.     MPTSDGLGLRQAITKEVLKQEKIIP
OXA-48-like_APP56266.1_K.p.     MPTSDGLGLRQAITKEVLKQEKIIP
OXA-48-like_APP50376.1_K.p.     MPTSDGLGLRQAITKEVLKQEKIIP
OXA-48-like_APP44504.1_K.p.     MPTSDGLGLRQAITKEVLKQEKIIP
OXA-48-like_APP38728.1_K.p.     MPTSDGLGLRQAITKEVLKQEKIIP
OXA-48-like_APM79240.1_K.p.     MPTSDGLGLRQAITKEVLKQEKIIP
OXA-48-like_APM73588.1_K.p.     MPTSDGLGLRQAITKEVLKQEKIIP
OXA-48-like_APM67987.1_K.p.     MPTSDGLGLRQAITKEVLKQEKIIP
OXA-48-like_APM62354.1_K.p.     MPTSDGLGLRQAITKEVLKQEKIIP
OXA-48-like_APM47124.1_K.p.     MPTSDGLGLRQAITKEVLKQEKIIP
OXA-48-like_APG80363.1_K.p.     MPTSDGLGLRQAITKEVLKQEKIIP
OXA-48-like_AOF13070.1_K.p.     MPTSDGLGLRQAITKEVLKQEKIIP
OXA-48-like_QPQ02212.1_K.p.     MPTSDGLGLRQAITKEVLKQEKIIP
OXA-48-like_QPQ02194.1_K.p.     MPTSDGLGLRQAITKEVLKQEKIIP
OXA-48-like_QIJ34285.1_K.p.     MPTSDGLGLRQAITKEVLKQEKIIP
OXA-48-like_QIJ09605.1_K.p.     MPTSDGLGLRQAITKEVLKQEKIIP
OXA-48-like_QIR58822.1_K.p.     MPTSDGLGLRQAITKEVLKQEKIIP
5OE0_1_Beta-lactamase           MPTSDGLGLRQAITKEVLKQEKIIP
OXA-48-like_ANE69785.1_K.p.     MPTSDGLGLRQAITKEVLKQEKIIP
OXA-48-like_ANE71804.1_K.p.     MPTSDGLGLRQAITKEVLKQEKIIP
OXA-48-like_ANE72730.1_K.p.     MPTSDGLGLRQAITKEVLKQEKIIP
OXA-48-like_AOA98603.1_K.p.     MPTSDGLGLRQAITKEVLKQEKIIP
OXA-48-like_CEP69754.1_K.p.     MPTSDGLGLRQAITKEVLKQEKIIP
OXA-48-like_CEP70686.1_K.p.     MPTSDGLGLRQAITKEVLKQEKIIP
OXA-48-like_CEP72746.1_K.p.     MPTSDGLGLRQAITKEVLKQEKIIP
OXA-48-like_ATX28768.1_K.p.     MPTSDGLGLRQAITKEVLKQEKIIP
OXA-48-like_ATX29640.1_K.p.     MPTSDGLGLRQAITKEVLKQEKIIP
OXA-48-like_AVO14768.1_K.p.     MPTSDGLGLRQAITKEVLKQEKIIP
OXA-48-like_AVO15618.1_K.p.     MPTSDGLGLRQAITKEVLKQEKIIP
OXA-48-like_AWD00385.1_K.p.     MPTSDGLGLRQAITKEVLKQEKIIP
OXA-48-like_AWD98100.1_K.p.     MPTSDGLGLRQAITKEVLKQEKIIP
OXA-48-like_AWS85277.1_K.p.     MPTSDGLGLRQAITKEVLKQEKIIP
OXA-48-like_QEG84876.1_K.p.     MPTSDGLGLRQAITKEVLKQEKIIP
OXA-48-like_QIR47572.1_K.p.     MPTSDGLGLRQAITKEVLKQEKIIP
OXA-48-like_QIR53202.1_K.p.     MPTSDGLGLRQAITKEVLKQEKIIP
OXA-48-like_QOQ50577.1_K.p.     MPTSDGLGLRQAITKEVLKQEKIIP
OXA-48-like_BBO98227.1_K.p.     MPTSDGLGLRQAITKEVLKQEKIIP
OXA-48-like_BBO99129.1_K.p.     MPTSDGLGLRQAITKEVLKQEKIIP
OXA-48-like_QDE47101.1_E.spp.   MPTSDGLGLRQAITKEVLKQEKIIP
OXA-48-like_AIG86783.1_K.p.     MPTSDGLGLRQAITKEVLKQEKIIP
OXA-48-like_ALH88756.1_K.p.     MPTSDGLGLRQAITKEVLKQEKIIP
OXA-48-like_AOF02466.1_K.p.     MPTSDGLGLRQAITKEVLKQEKIIP
OXA-48-like_ATO26616.1_K.p.     MPTSDGLGLRQAITKEVLKQEKIIP
OXA-48-like_AZB79861.1_K.p.     MPTSDGLGLRQAITKEVLKQEKIIP
OXA-48-like_AZV17592.1_K.p.     MPTSDGLGLRQAITKEVLKQEKIIP
OXA-48-like_QBG05392.1_K.p.     MPTSDGLGLRQAITKEVLKQEKIIP
OXA-48-like_QBH46740.1_K.p.     MPTSDGLGLRQAITKEVLKQEKIIP
OXA-48-like_QBH61269.1_K.p.     MPTSDGLGLRQAITKEVLKQEKIIP
OXA-48-like_QLF61798.1_K.p.     MPTSDGLGLRQAITKEVLKQEKIIP
OXA-48-like_QLF61822.1_K.p.     MPTSDGLGLRQAITKEVLKQEKIIP
                            acc
```
